# Carbene-catalyzed chemoselective reaction of unsymmetric enedials for access to Furo[2,3-b]pyrroles

**DOI:** 10.1038/s41467-023-39988-z

**Published:** 2023-07-15

**Authors:** Guodong Fan, Qingyun Wang, Jun Xu, Pengcheng Zheng, Yonggui Robin Chi

**Affiliations:** 1 State Key Laboratory Breeding Base of Green Pesticide and Agricultural Bioengineering, Key Laboratory of Green Pesticide and Agricultural Bioengineering, Ministry of Education, Guizhou University, 550025 Guiyang, China; 2 Guizhou University of Traditional Chinese Medicine, 550025 Guiyang, China; 3 School of Chemistry, Chemical Engineering, and Biotechnology, Nanyang Technological University, Singapore, 637371 Singapore

**Keywords:** Catalysis, Reaction mechanisms

## Abstract

A carbene-catalyzed chemoselective reaction of unsymmetric enedials is disclosed. The reaction provides a concise access to bicyclic furo[2,3-b]pyrroles derivatives in excellent selectivity. A main challenge in this reaction is chemoselective reaction of the two aldehyde moieties in the enedial substrates. Mechanistic studies via experiments suggest that our chemoselectivity controls are mostly achieved on the reducing properties of different sited Breslow intermediates. Several side reactions processes and the corresponding side adducts are also studied by high resolution mass spectroscopy analysis. Our method allows for efficient assembly of the furo[2,3-b]pyrrole structural moieties and their analogues widely found in natural products and pharmaceuticals.

## Abstract (web summary)

Chemoselective reactions of two or multiple functional groups with similar reactivities remain an ongoing challenge in chemistry. Here the authors disclosed a chemoselective reaction of enedial and observed that the high chemoselectivity is mainly controlled via redox (rather than steric) properties of the substrate/catalyst.

## Introduction

**Fig. 1:**
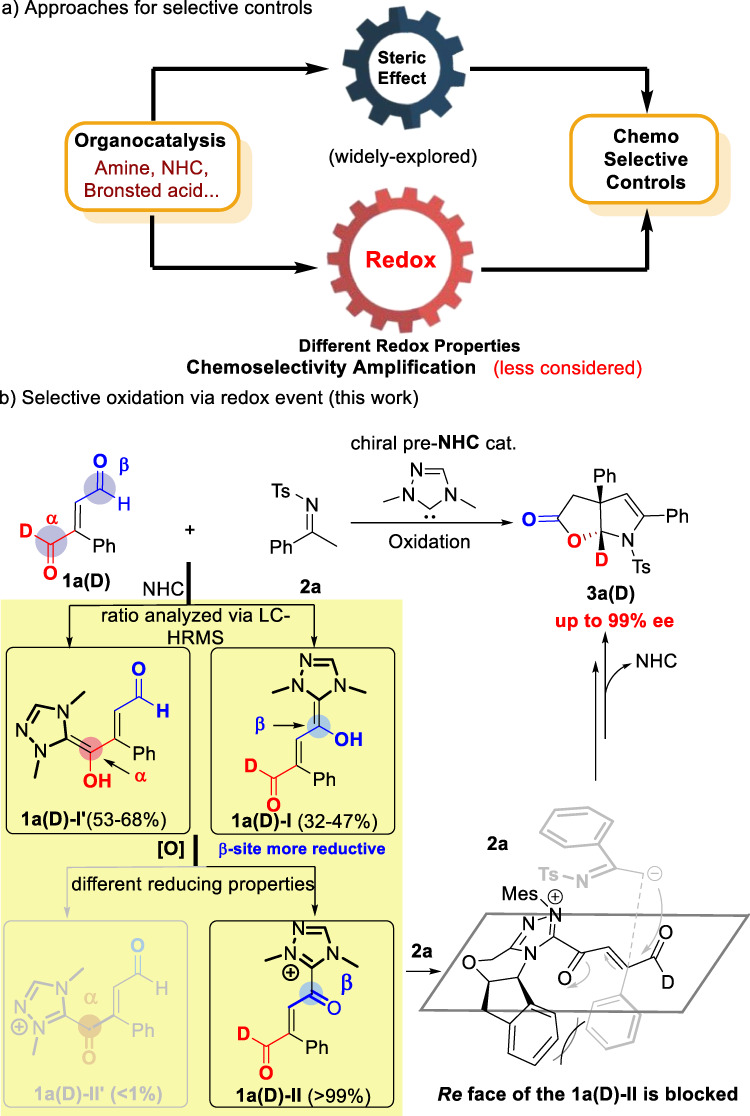
Selective reactions of unsymmetric enedial. **a** Approaches for selective controls. **b** Selective oxidation via redox event.

Chemoselective reactions of two or multiple functional groups with similar reactivities remain an ongoing challenge in chemistry. Some of such selectivity issues may be addressed (in industrial practices) through the use (and recycle) of certain reagents in large excess. On the other hand, it is much more attractive both scientifically and practically to achieve selective reactions in the first places. Organic catalysis has been proven to be a versatile approach for activation and selective controls^1–5^. Much attention have been placed to modulate the steric interactions between the catalysts and substrates to achieve selective controls (Fig. 1a)^6–9^. N-heterocyclic carbene (NHC) catalysts^10–14^ provide unique activation and reaction modes for a diverse set of reactions, and impressive reactions with selectivity modulated mostly by steric interactions have been widely studied^15–19^. Furthermore, it is well explored that the Breslow intermediates can be oxidated by various oxidants^20–31^. However, the high chemoselectivity is mainly controlled via redox (rather than steric) properties of the substrate/catalyst remains undeveloped to date. It appears to us the redox properties brought by both the NHC catalysts and the reacting substrates can be explored to achieve unusual reaction controls (Fig. 1a). Specifically, in such a scenario, turning the redox properties of catalysts and substrates/reagents can likely have profound effects on the reaction outcomes^32–35^.

In this work, we develop a chemo- and enantioselective strategy transforming unsymmetric enedials to furo[2,3-b]pyrrole containing molecules. The overall chemoselectivity observed is due to an oxidation event of the Breslow intermediate which provides a very significant increase to the chemoselectivity. Chemoselective reactions of unsymmetric enedials can be achieved through an oxidative process (Fig. 1b). Mechanistic studies via high-resolution mass spectroscopy (HRMS) suggest that our process is chemoselective during two of the key steps. With imine^36–39^ as the other reaction partner, our chemoselective reactions of unsymmetric enedials afford bicyclic furo[2,3-b]pyrroles derivatives in excellent diastereo- and enantioselectivity with the formation of four new chemical bonds and two chiral centers. Our findings can likely stimulate further explorations for unique reactions by placing more attentions on the redox steps of many organic catalytic reactions.

## Results

### Reaction development

**Fig. 2:**
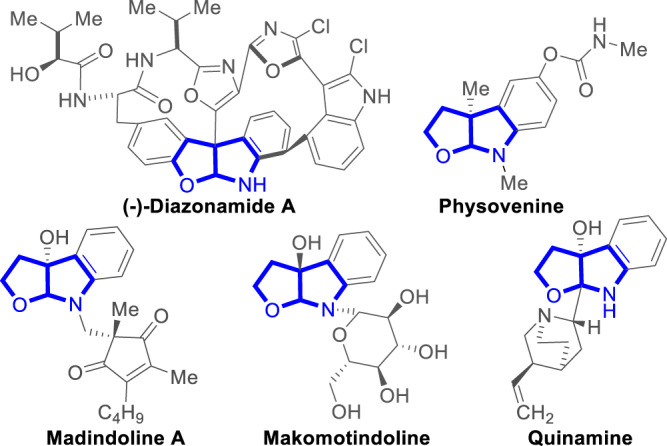
Functional molecules containing furo[2,3-b]pyrrole. Examples of functional chiral molecules.

Our study provides a much less explored strategy via a redox process in achieving otherwise challenging chemoselectivity. Further development via similar logics is expected to bring surprises in reactions controls. In the model reaction, the addition of NHC catalyst to the enedial leads to the formation of Breslow intermediate between the NHC catalyst and the enendial substrate did not provide sufficient differentiations (Fig. 1b). Subsequently, it was the chemoselectivity determining step that the oxidation of the Breslow intermediates to form the α, β-unsaturated acyl azolium intermediates **1a(D)**-**II** and **1a(D)**-**II’**. In the redox step, the two intermediates **1a(D)**-**II** and **1a(D)**-**II’** exhibit excellent chemoselectivity ratio 99:1. Then the acyl azolium intermediate **1a(D)**-**II** reacted with imine to form the final chiral furo[2,3-b]pyrrole containing products with high optical purities. Such furo[2,3-b]pyrrole is a common structural motif widely found in natural products and pharmaceuticals, such as diazonamide, physovenine and quinamine (Fig. 2)^40–50^. Key results of the optimization of the reaction conditions are summarized in Table 1. Enedial **1a** was selected to react with imine **2a** under the catalysis of various pre-NHC catalysts (Table 1, entry 1 to 3, respectively). We found that pre-catalyst A bearing a 2,4,6-trimethylphenyl (Mes) group, gave the product **3a** in a moderate yield and 96:4 er (Table 1, entry 1). The enantioselectivity could be enhanced by switching bases, and **3a** was obtained in 51% yield and 97:3 er by using K_2_CO_3_ (Table 1, entry 5). In addition, the solvents had a strong impact on reactions, switching THF to MeCN gave the 66% yield and the excellent enantioselectivity (98:2 er) with 4 Å molecular sieves (MS) as an additive (Table 1, entry 12). Furthermore, we evaluated different sterically hindered oxidants, it was found no obviously erosion of the er values and the yields when the bulky less oxidants were employed (Table 1, entry 13, 14). The product **3a** formed in this chiral catalytic process was isolated as a single diastereomer.

**Table 1:** Initial studies and reaction optimization^a^


Entry	NHC	base	solvent	yield (%)^b^	er^c^
1	**A**	Na_2_CO_3_	THF	40	96:4
2	**B**	Na_2_CO_3_	THF	10	90:10
3	**C**	Na_2_CO_3_	THF	N.R.	–
4	**A**	Et_3_N	THF	42	99:1
5	**A**	K_2_CO_3_	THF	51	97:3
6	**A**	NaOAc	THF	32	98:2
7	**A**	K_2_CO_3_	EA	56	96:4
8	**A**	K_2_CO_3_	DCE	N.R.	–
9	**A**	K_2_CO_3_	DMSO	N.R.	–
10	**A**	K_2_CO_3_	NMP	N.R.	–
11	**A**	K_2_CO_3_	MTBE	N.R.	–
12	**A**	K_2_CO_3_	MeCN	66	98:2
13^d^	**A**	K_2_CO_3_	MeCN	63	98:2
14^e^	**A**	K_2_CO_3_	MeCN	61	98:2

^a^Unless otherwise specified, the reactions were conducted with **1a** (0.13 mmol), **2a** (0.10 mmol), NHC (0.02 mmol), base (0.02 mmol), DQ A (0.13 mmol), 4 Å MS (150 mg) and solvent (2.0 mL) at 45 °C for 12 h.

^b^Isolated yield of **3a**.

^c^The er values were determined via HPLC on chiral stationary phase.

^d^The reaction uses DQ B as the oxidant.

^e^The reaction uses BQ as the oxidant.

### Substrate scope

**Fig. 3:**
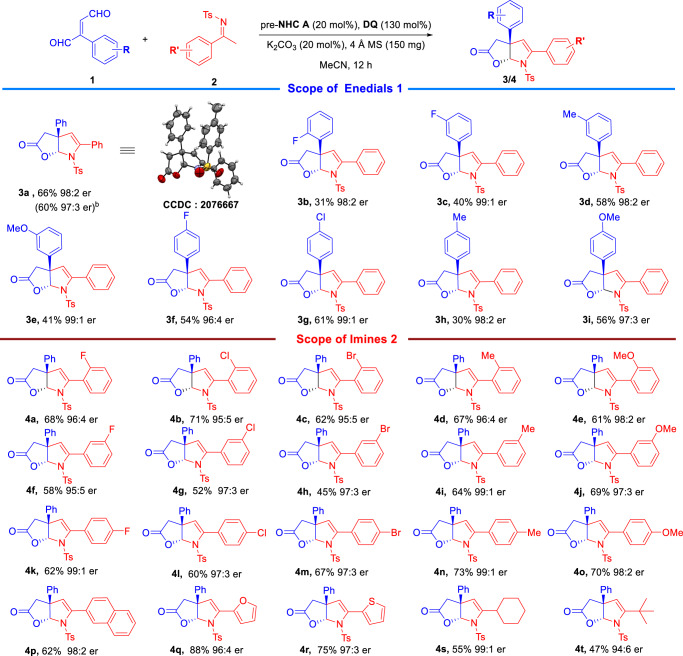
Substrates scope of the reaction^a^. ^a^Reaction conditions as stated in Table 1, entry 12. Yields were isolated yields after purification by column chromatography. Er values were determined via HPLC on chiral stationary phase. ^b^The reaction was carried out at 1.0 mmol scale based on **2a**.

Having established acceptable reaction conditions, we evaluated the generality of the reaction using substrates enedial **1** and imine **2** with different substitutions (Fig. 3). Initially, we studied the reaction scope of enedials **1**. Both electron-withdrawing and electron-donating substituents could be installed on each (para-, meta- or ortho-) position of benzene ring of the enedials substrate **1**, with the corresponding products afforded in moderate to good yields with high optical purities (Fig. 3, **3a**–**3i**). Alkyl enedials substrates were not be synthesized by general procedure, which was mentioned in supporting information. We will further investigate this problem in our further studies of this class of molecules in relevant application-driven projects. The catalytic approach could also be carried out at 1.0 mmol scale with the desired product **3a** afforded in good yield and excellent enantioselectivity (258.9 mg, 60%, 97:3 er). We then moved to examine the reaction scope of imine as substrates **2** with enedial **1a** (Fig. 3). It was found that both electron-withdrawing and electron-donating substituents were well tolerated (**4a**–**4o**), and the desired products were produced in moderate to good yields and excellent enantioselectivities. The benzene ring of the imine could also be replaced with naphthalene group and the desired product **4p** was produced in good yield and high enantioselectivity. The substituted benzene moiety of the imine could be exchanged to heteroaromatic moieties without erosion of enantioselectivities and the yields of corresponding products were enhanced (**4q**–**4r**). It is worth noting that aliphatic imines could also be used as suitable reaction substrates in this reaction, with the desired product **4** **s** and **4t** afforded in moderate yields and excellent enantioselectivities. The er value of the product **4t** decreased slightly when a tert-butyl imine was used as the substrate, this may be caused by the steric hindrance.

### Synthetic transformations

**Fig. 4:**
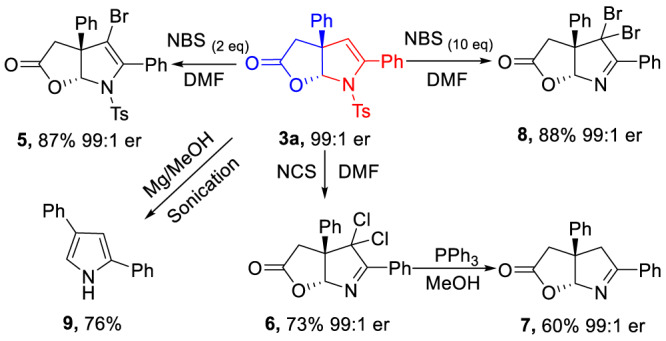
Synthetic transformations of 3a. Derivatization studies on **3a**.

Additionally, the chiral furo[2,3-b]pyrrole containing product **3a** was easily transformed into various derivatives through simple protocols (Fig. 4). For instance, the hydrogen atom of **3a** was employed to react with N-bromo-succinimide (NBS) (2 eq), the brominated product **5** was obtained in 87% yield and 99:1 er. Increasing the loading of NBS (up to 10 eq), the dibromo product **8** could be isolated with high optical purity. Moreover, switching NBS to N-chloro-succinimide (NCS), the dichloride product **6** could be obtained in good yield and excellent er value. In addition, compound **7** could be synthesized when using triphenylphosphine to reduce the dichloride product **6**. The absolute configuration of products was determined by X-ray diffraction analysis. The **3a** could be completely reduced with Mg/MeOH to give the diphenyl substituted pyrrole **9** in 76% yield.

### Mechanistic studies

**Fig. 5:**
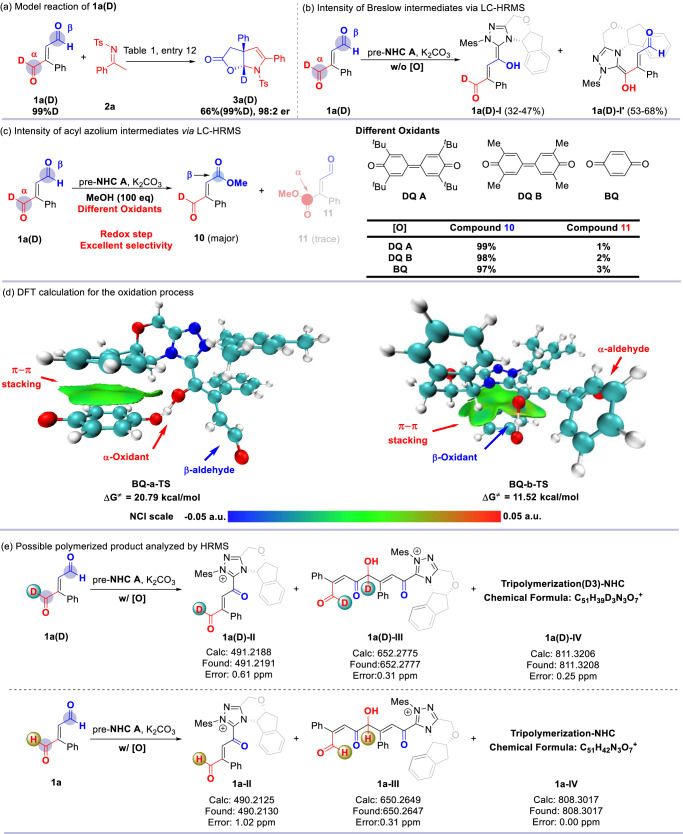
Additional supporting mechanistic experiments. **a** Model reaction of **1a(D)**. **b** Intensity of Breslow intermediates via LC-HRMS. **c** Intensity of acyl azolium intermediates via LC-HRMS. **d** DFT calculation for the oxidation process. **e** Possible polymerized product analyzed by HRMS.

To understand the mechanism of the reaction, some mechanistic studies were performed. The deuterium labeled (abbreviated as D-labeled) α*-*aldehyde moiety substrate **1a(D)** was synthesized, then **1a(D)** was reacted with imine **2a** in the model reaction condition (Table 1, entry 12). Finally, the D-labeled product **3a(D)** was obtained in 66% yield and 98:2 er (Fig. 5a), the reaction results were similar to non-deuterium labeled model reaction. It suggested that the D-labeled **1a(D)** can be used as model substate to study the driving force of chemoselectivity in reactions.

The carbene catalyst addition to one of the two aldehyde moieties of **1a(D)** to form two type Breslow intermediates **1a(D)-I** and **1a(D)-I’** (Fig. 5b), the D-labeled Breslow intermediate **1a(D)-I** was one Dalton greater than the non-labeled Breslow intermediate **1a(D)-I’**, the differentiation of the molecular weight can be determined via in situ liquid chromatography high resolution mass spectroscopy (LC-HRMS), it means that the reacted site can be clearly confirmed. Furthermore, the response intensity of the D-labeled and non-labeled Breslow intermediates was detected and differentiated by LC-HRMS. It was found that the intensity ratio of **1a(D)-I** and **1a(D)-I’** was (32-47%):(53-68%), which means that the β-aldehyde moiety was more favorably reacted with NHC catalyst.

Subsequently, the selective redox of Breslow **1a(D)-I** and **1a(D)-I’** was studied. Base on the model reaction, 3,3’,5,5’-tetra-tert-butyl-[1,1’-bi(cyclohexylidene)]−2,2’,5,5’-tetraene-4,4’-dione (DQ A) was added to oxidize the Breslow intermediate to form the acyl azolium intermediate and MeOH (100 eq) was added to quench acyl azolium intermediates to form the ester products (Fig. 5c). In the redox process, the D-labeled site remained intact. The intensity of ester products **10** and **11** were detected by LC-HRMS, the intensity ratio of them was 99:1. In the same way, we carried out further studies to replace the large sterically hindered oxidant DQ A with bulky less oxidants 3,3’,5,5’-tetramethyl-[1,1’-bi(cyclohexylidene)]−2,2’,5,5’-tetraene-4,4’-dione (DQ B) and benzoquinone (BQ) to obtain the same esterification products (**10** and **11**). The intensity of products **10** and **11** were detected by LC-HRMS under same conditions, the ratios were 98:2 (DQ B as oxidant) and 97:3 (BQ as oxidant), with no obvious negative effects compared to using DQ A as oxidant. Steric hindrance is not an important factor for this chemoselective oxidation reaction. Disparity in redox properties of Breslow intermediates with different reaction sites is more likely to lead to selective oxidation, it suggested that the formation of acyl azolium intermediate **1a(D)-II** was much faster than **1a(D)-II’** (Fig. 5c). Additional density functional theory (DFT) calculations were performed to estimate the Gibbs free energy barriers of the oxidation of Breslow intermediates (Fig. 5d). In the calculation, the less sterically hindered BQ was selected as the oxidant in order to exclude the effect of steric hindrance. It was found that the β-site Breslow intermediate (ΔG^≠^ = 11.52 kcal/mol) was more easily oxidated than the α-site (ΔG^≠^ = 20.79 kcal/mol). Furthermore, the non-covalent interactions (NCI) of the transition structures were analyzed. It was shown that only the π-π stacking interaction were found between BQ and Breslow intermediates in the transition state, and the transition state could be stabled by the weak interactions. Overall, the experimental observations and DFT calculations were consistent with the finding that the reducibility of the β-site Breslow intermediate was higher than that of α-site when steric bulk was not present.

In the model reaction condition (such as Table 1, entry 12), the yield of desired product **3a** didn’t over 70%. Unfortunately, no obvious byproduct could be isolated by flash column chromatography. To well investigate the formation of byproduct in the model reaction, the additional experiments were carried out (Fig. 5e). Comparing with the standard condition (Table 1, entry 12), the D-labeled enedial **1a(D)** was employed in the control experiment, and the reaction was determined by in situ LC-HRMS. Finally, some polymerized products were found in the reaction. It was normal that the D-labeled acyl azolium intermediate **1a(D)-II** was formed in the reaction. However, the remaining aldehyde moiety of acyl azolium intermediate **1a(D)-II** was activated by acyl azolium moiety, and reacted with Breslow intermediate to form dimer intermediate **1a(D)-III**. Furthermore, the **1a(D)-III** continuously reacted with another Breslow intermediate and oxidized to form trimer **1a(D)-IV**. Comparing with non-labeled enedial **1a**, all the D-labeled site remained intact, so that the reacted sites can be clearly identified by HRMS. The fragmentation of **1a-IV** in liquid chromatography high resolution accurate mass spectrometer (LC-HRMS-MS) was investigated and reviewed. In order to further speculate the structure of the reaction byproducts, we found some possible intermediates, including **1a-II**, etc. (see Supplementary Information). The formation of dimer and trimer polymerized products indicated that high reactivity aldehyde moiety of acyl azolium intermediate **1a(D)-II** can easily and indiscriminately react with other nucleophilic substrates or intermediates. Thus, desired product and byproduct were formed, which will lead to decrease the yield of desired products.

This furo[2,3-b]pyrrole is a common structural motif widely found in natural products and drugs, we were interested in preliminary exploration of its bioactivity. The furo[2,3-b]pyrrole derivatives from our approach also exhibited biological activity in our research on novel pesticide development for crop protections. For example, many of our compounds showed good antibacterial activities against *Xanthomonas axonopodis* pv. *citri (Xac)* which can cause citrus canker and result in huge economic losses in the production of lemons, oranges and grapefruits. According to the analysis of Table 2, it was found that substrates with ortho-F (**3b**) and para-methoxy (**3i**) substituents exhibited better activity when the enedials substrates were expanded. In addition, It was observed that when the methyl group was installed at the imine(**4i)** exhibited good activity. Moreover, the derivatives **6, 7** and **8** from **3a**, showed good activity exhibited good activity as well. Compared with the thiodiazole copper and bismerthiazo that have been widely used as commercially available antibacterial agrichemicals, some furo[2,3-b]pyrrole chiral molecules afforded from our method exhibited obviously superior antibacterial activities and can be regarded as promising candidates in the search for new pesticide lead compounds.

**Table 2:** In vitro antibacterial activity of the target compounds against *Xac*^a^

Compounds	*Xanthomonas axonopodis* pv. *citri* (*Xac*) Inhibition rate / %	
**3a**	57.15 ± 1.21 (230 μM/L)	26.77 ± 2.01 (120 μM/L)
**3a(D)**	56.94 ± 1.15 (230 μM/L)	30.27 ± 2.45 (120 μM/L)
**3b**	95.81 ± 4.01 (220 μM/L)	37.96 ± 1.17 (110 μM/L)
**3d**	46.24 ± 0.40 (220 μM/L)	33.23 ± 0.17 (110 μM/L)
**3e**	50.97 ± 0.35 (220 μM/L)	33.28 ± 1.82 (110 μM/L)
**3f**	46.40 ± 0.85 (220 μM/L)	30.65 ± 0.66 (110 μM/L)
**3g**	46.67 ± 7.18 (210 μM/L)	35.91 ± 0.21 (110 μM/L)
**3i**	97.42 ± 0.80 (220 μM/L)	35.70 ± 1.50 (110 μM/L)
**4d**	73.06 ± 1.97 (220 μM/L)	28.01 ± 6.41 (110 μM/L)
**4f**	55.86 ± 4.89 (220 μM/L)	36.45 ± 7.58 (110 μM/L)
**4i**	90.48 ± 2.21 (220 μM/L)	53.66 ± 0.87 (110 μM/L)
**4k**	67.31 ± 0.40 (220 μM/L)	50.86 ± 2.48 (110 μM/L)
**4q**	65.48 ± 0.82 (240 μM/L)	40.27 ± 4.29 (120 μM/L)
**4r**	27.80 ± 1.05 (230 μM/L)	23.98 ± 0.84 (110 μM/L)
**5**	68.92 ± 0.68 (200 μM/L)	36.40 ± 5.95 (100 μM/L)
**6**	89.62 ± 1.32 (290 μM/L)	80.43 ± 5.56 (140 μM/L)
**7**	94.78 ± 0.42 (360 μM/L)	80.16 ± 0.40 (180 μM/L)
**8**	92.31 ± 5.16 (230 μM/L)	39.35 ± 2.61 (110 μM/L)
Thiodiazole copper^b^	57.11 ± 4.48 (300 μM/L)	27.90 ± 2.14 (150 μM/L)
Bismerthiazol^b^	94.52 ± 1.84 (360 μM/L)	55.22 ± 3.44 (180 μM/L)

^a^All data were average data of three replicates.

^b^Commercial bactericide, used as the positive control.

In summary, we have developed a chemo- and enantioselective strategy in transforming unsymmetric enedials to furo[2,3-b]pyrrole containing molecules. Our results suggested that the initial addition steps between the NHC catalyst and the aldehyde moieties of the enedial substate (to form the corresponding Breslow intermediates) do not provide sufficient differentiations on the two aldehyde moieties. Instead, the observed overall chemoselectivity was resulted from the Breslow intermediate oxidation event that offers a very significant chemoselectivity increase. Furthermore, multiple additional experiments were performed for the redox process, it was found that all the bulky or less bulky oxidants could be priority reducted by β-site Breslow intermediate, the observed overall chemoselectivity was resulted from the reducing properties of different sited Breslow intermediates. Multiple possible side reactions were elucidated by analyzing the reaction mixtures via LC-HRMS. The chiral furo[2,3-b]pyrroles derivatives from our reactions can be easily transformed into various functiona molecules. Further studies on the bioactivities of these chiral furo[2,3-b]pyrrole compounds for agricultural applications had been evaluated, the preliminary results suggested that these molecules show encouraging in vitro activities against *Xac*. Our method and its mechanistic implications shall also stimulate further reflect on driving force of chemoselective for the molecules bearing multiple structural units with similar reactivities.

## Methods

### General procedure for the catalytic reactions

To a dry 4.0 mL vial equipped with a magnetic stir bar, was added **1a** (0.13 mmol), **2a** (0.10 mmol), **pre-NHC A** (0.02 mmol), DQ A (0.13 mmol) and base (0.02 mmol). After purges with N_2_ in glove-box, anhydrous MeCN (2.0 mL), and 4 Å MS (150.0 mg) was added and sealed. The reaction mixture was stirred at 45 °C for 12 h. Then the mixture was directly concentrated under reduced pressure to afford a crude product. The crude product was purified via column chromatography on silica gel (petroleum ether/ethyl acetate = 5/1) to afford the desired product **3a**.

### Reporting summary

Further information on research design is available in the Nature Portfolio Reporting Summary linked to this article.

### Supplementary information

Supplementary Information

Description of Additional Supplementary Files

supplementary data 1

Reporting Summary

## Data Availability

The experimental method and data generated in this study are provided in the Supplementary Information file. Geometries of all DFT optimized structures (in. xyz format) are provided as Supplementary Data file. The crystallographic data for structures of **1a**, **3a**, **5**, **6** and **7** have been deposited in the Cambridge Crystallographic Data Centre under accession CCDC code **2156768**, **2076667**, **2156751**, **2156752** and **2156755**, respectively. Copies of the data can be obtained free of charge via www.ccdc.cam.ac.uk/data_request/cif.
