# Author Correction: Carbene-catalyzed chemoselective reaction of unsymmetric enedials for access to Furo[2,3-b]pyrroles

**DOI:** 10.1038/s41467-024-46202-1

**Published:** 2024-02-26

**Authors:** Guodong Fan, Qingyun Wang, Jun Xu, Pengcheng Zheng, Yonggui Robin Chi

**Affiliations:** 1 State Key Laboratory Breeding Base of Green Pesticide and Agricultural Bioengineering, Key Laboratory of Green Pesticide and Agricultural Bioengineering, Ministry of Education, Guizhou University, 550025 Guiyang, China; 2 Guizhou University of Traditional Chinese Medicine, 550025 Guiyang, China; 3 School of Chemistry, Chemical Engineering, and Biotechnology, Nanyang Technological University, Singapore, 637371 Singapore

**Keywords:** Catalysis, Reaction mechanisms

Correction to: *Nature Communications* 10.1038/s41467-023-39988-z, published online 15 July 2023

This Article contains an error in Fig. 1 in which a phenyl substituent on the position of the γ carbon atom in the structure of 2a was omitted. The error has been corrected in the PDF or HTML version of the Article.

