# Supplementary material for: Carbene-catalyzed chemoselective reaction of unsymmetric enedials for access to Furo[2,3-b]pyrroles
Source: Nat Commun. 2023 Jul 15;14:4243. doi: 10.1038/s41467-023-39988-z (PMC10349821; doi:10.1038/s41467-023-39988-z)
Supplement: Supplementary file 1 — Supplementary Information [file 41467_2023_39988_MOESM1_ESM.pdf]

Supplementary Information

Carbene-Catalyzed Chemoselective Reaction of Unsymmetric Enedials for Access to Furo[2,3-b]pyrroles

Guodong Fan,[1] Qingyun Wang,[1] Jun Xu,[2] Pengcheng Zheng[1]* and Yonggui Robin Chi [1,3]*

[1] State Key Laboratory Breeding Base of Green Pesticide and Agricultural Bioengineering, Key Laboratory of Green Pesticide and Agricultural Bioengineering, Ministry of Education, Guizhou University, Guiyang 550025, China

[2] Guizhou University of Traditional Chinese Medicine, Guiyang 550025, China

[3] School of Chemistry, Chemical Engineering, and Biotechnology, Nanyang Technological University, Singapore 637371, Singapore

*Corresponding authors e-mails:

zhengpc1986@163.com

robinchi@ntu.edu.sg

Table of Contents

I. Supplementary Methods 3
General information 3
Preparation of substrates 4
Experimental section: condition optimization for the synthesis of **3a** 6
General procedure for the catalytic reactions 8
DFT Calculations of oxidation selectivity 10
Mechanistic studies ESI-HRMS analysis of the catalyst system 11
Antibacterial activity in Vitro *(Xac)* 26
II. Supplementary Discussions 28
Postulated reaction mechanism 28
III. Supplementary Notes 31
Characterization of substrates and products 31
IV. Supplementary Figures 46
Stereochemistry determination via X-ray crystallographic analysis 46
^{1}H NMR, ^{13}C NMR, ^{19}F NMR and HPLC spectra 48
V. Supplementary References 136

I. Supplementary Methods

General information

Commercially available materials and dry solvents purchased from Energy Chemical and Bidepharm were used as received. Unless otherwise specified, all reactions were prepared using 4 mL vial under N_2 atmosphere in glove-box from Mbraun (UNILAB SP). Proton nuclear magnetic resonance (^{1}H NMR) spectra were recorded on a Bruker (AVANCE III HD 400 MHz) spectrometer. Chemical shifts were recorded in parts per million (ppm, δ) relative to tetramethylsilane (δ 0.00) or chloroform ($\delta = 7.26$, singlet). ^{1}H NMR splitting patterns are designated as singlet (s), doublet (d), triplet (t), quartet (q), dd (doublet of doublets); m (multiplets), and etc. All first-order splitting patterns were assigned on the basis of the appearance of the multiplet. Splitting patterns that could not be easily interpreted are designated as multiplet (m) or broad (br). Carbon nuclear magnetic resonance (^{13}C NMR) spectra were recorded on a Bruker (AVANCE III HD 101 MHz) spectrometer. Fluorine (^{19}F) nuclear magnetic resonance (^{19}F NMR) spectra were recorded on a Bruker (AVANCE III HD 376 MHz) spectrometer. The melting points (m.p.) of the title compounds were determined when left untouched on an XT-4-MP apparatus from Beijing Tech. Instrument Co. (Beijing, China). High resolution mass spectrometer analysis (HRMS) was performed on Waters Xevo G2-S QTof mass spectrometer. The gas chromatography high resolution mass spectrometer (GC-HRMS) analyses were measured on Thermo Scientific TM Exactive GC. Absolute configuration of the products was determined by X-ray crystallography (Bruker d8 quest). HPLC analyses were measured on Waters systems with Empower 3 system controller, Alliance 2695, and 2998 Diode Array Waters 2489 UV/Vis detector. Chiralcel brand chiral columns from Daicel Chemical Industries were used with models IB, in 4.6 × 250 mm size. The racemic products used to determine the er values were synthesized using racemic catalyst. Optical rotations were measured on a Insmark IP-digi Polarimeter in a 1 dm cuvette at 25 ℃. The concentration (*c*) is given in g/100 mL. Analytical thin-layer chromatography (TLC) was carried out pre-coated silica gel plate (0.2 mm thickness). Visualization was performed using a UV lamp.

Preparation of substrates

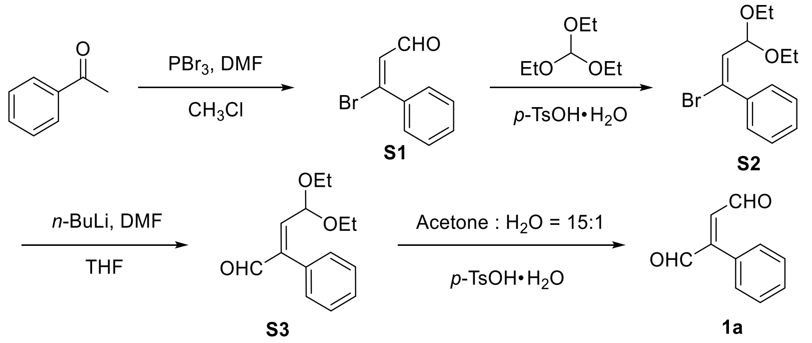

Supplementary Figure 1. Synthesis method of product **1a**.

DMF (2.0 mL, 26.0 mmol) in 25.0 mL dry $CHCl_3$ was added PBr_3 (2.2 mL, 23.0 mmol) dropwise at 0 °C, and white solid precipitated during the addition process. The suspension was stirred for further 30 minutes, and acetophenone (1.1 mL, 10.0 mmol) in 5.0 mL dry $CHCl_3$ was added dropwise at 0 °C. The mixture was allowed to warm to rt and stirred for about 8 hours. After completely consumption of acetophenone determined by TLC, the mixture was poured into ice water, and $NaHCO_3$ were added to neutralize to pH ≈ 6. After extraction with CH_2Cl_2, the organic layer was washed with brine, dried over anhydrous Na_2SO_4, and concentrated under reduced pressure to afford the crude product of β-bromo-enal **S1**. To a solution of β-bromo-enal **S1** in triethyl orthoformate (8.3 mL, 50.0 mmol) was added *p*-TsOH•H_2O (34.0 mg, 2.0 mmol), and the resulting mixture was stirred at rt for 2 hours. The crude reaction mixture was purified by silica gel column chromatography to afford the corresponding product **S2**. To a stirred solution of **S2** in anhydrous THF (50.0 mL), solution of *n*-BuLi in *n*-hexane (4.8 mL, 2.5 M, 12.0 mmol) was added dropwise at -78 °C for 5 minutes. The mixture was stirred at the same temperature for 50 minutes, and then DMF (0.9 mL, 12.0 mmol) was added to the mixture at -78 °C for 10 minutes. The mixture was allowed to warm to ambient temperature, quenched with H_2O (10.0 mL), extracted with EtOAc (3 × 15.0 mL). The combined organic phase was dried over anhydrous Na_2SO_4, purified by silica gel column chromatography to afford the corresponding **S3**. To the solution of **S3** in mixture of acetone and water (15:1, v/v), *p*-TsOH•H_2O (34.0 mg, 2.0 mmol) was added and stirred at 60 °C for 2 hours. The reaction mixture was extracted with EtOAc (3 × 15.0 mL), then the combined organic phase was dried over anhydrous Na_2SO_4, and purified by silica gel column chromatography to afford the corresponding product **1a**.

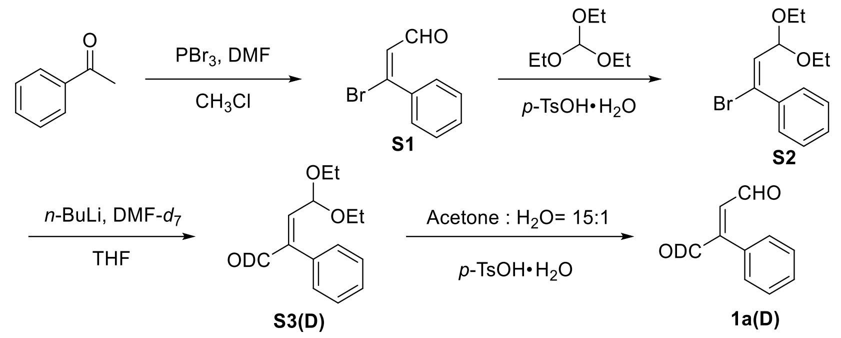

Supplementary Figure 2. Synthesis method of product **1a(D)**.

DMF (2.0 mL, 26.0 mmol) in 25.0 mL dry $CHCl_3$ was added PBr_3 (2.2 mL, 23.0 mmol) dropwise at 0 °C, and white solid precipitated during the addition process. The suspension was stirred for further 30 minutes, and acetophenone (1.1 mL, 10.0 mmol) in 5.0 mL dry $CHCl_3$ was added dropwise at 0 °C. The mixture was allowed to warm to rt and stirred for about 8 hours. After completely consumption of acetophenone determined by TLC, the mixture was poured into ice water, and $NaHCO_3$ were added to neutralize to pH ≈ 6. After extraction with CH_2Cl_2, the organic layer was washed with brine, dried over anhydrous Na_2SO_4, and concentrated under reduced pressure to afford the crude product of β-bromo-enal **S1**. To a solution of β-bromo-enal **S1** in triethyl orthoformate (8.3 mL, 50.0 mmol) was added *p*-TsOH•H_2O (34.0 mg, 2.0 mmol), and the resulting mixture was stirred at rt for 2 hours. The crude reaction mixture was purified by silica gel column chromatography to afford the corresponding product **S2**. To a stirred solution of **S2** in anhydrous THF (50.0 mL), solution of *n*-BuLi in *n*-hexane (4.8 mL, 2.5 M, 12.0 mmol) was added dropwise at -78 °C for 5 minutes. The mixture was stirred at the same temperature for 50 minutes, and then DMF-d_7 (0.9 mL, 12.0 mmol) was added to the mixture at -78 °C for 10 minutes. The mixture was allowed to warm to ambient temperature, quenched with H_2O (10.0 mL), extracted with EtOAc (3 × 15.0 mL). The combined organic phase was dried over anhydrous Na_2SO_4, and purified by silica gel column chromatography to afford the corresponding **S3(D)**. To the solution of **S3(D)** in mixture of acetone and water (15:1, v/v), *p*-TsOH•H_2O (34.0 mg, 2.0 mmol) was added and stirred at 60 °C for 2 hours. The reaction mixture was extracted with EtOAc (3 × 15.0 mL), then the combined organic phase was dried over anhydrous Na_2SO_4, and purified by silica gel column chromatography to afford the corresponding product **1a(D)**.

Experimental section: condition optimization for the synthesis of **3a**

Supplementary Table 1. The effects of catalysts, bases, solvents on the reaction outcome[a].

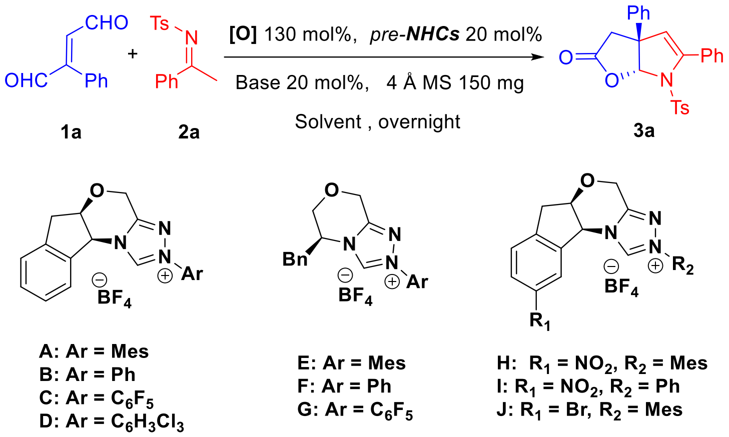

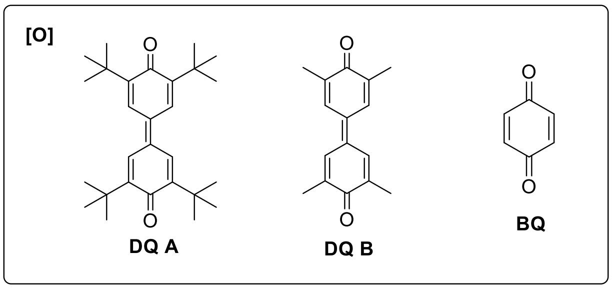

Entry	Cat.	Base	Solvent	Yield (%)[b]	Er[c]
1	**A**	Cs_2CO_3	THF	24	97:3
2	**B**	Cs_2CO_3	THF	9	90:10
3	**C**	Cs_2CO_3	THF	NR	-
4	**D**	Cs_2CO_3	THF	NR	-
5	**E**	Cs_2CO_3	THF	10	80:20
6	**F**	Cs_2CO_3	THF	7	82:18
7	**G**	Cs_2CO_3	THF	NR	-
8	**H**	Cs_2CO_3	THF	10	96:4
9	**I**	Cs_2CO_3	THF	12	89:11
10	**J**	Cs_2CO_3	THF	13	95:5
11	**A**	Na_2CO_3	THF	40	96:4
12	**A**	K_2CO_3	THF	51	98:2
13	**A**	NaOAc	THF	32	98:2
14	**A**	Et_3N	THF	42	99:1
15	**A**	DIPEA	THF	29	92:8
16	**A**	NaOH	THF	38	98:2
17	**A**	K_2CO_3	EA	56	96:4
18	**A**	K_2CO_3	DCE	NR	-
19	**A**	K_2CO_3	DMSO	NR	-
20	**A**	K_2CO_3	NMP	NR	-
21	**A**	K_2CO_3	MTBE	NR	-

22[d]	**A**	K_2CO_3	MeCN	66	98:2
23[e]	**A**	K_2CO_3	MeCN	63	98:2
24[f]	**A**	K_2CO_3	MeCN	61	98:2

[a]General conditions (unless otherwise specified): **1a** (0.13 mmol), **2a** (0.10 mmol), pre-**NHC** (0.02 mmol), DQ A (0.13 mmol), base (0.02 mmol), solvent (2.0 mL), 45 °C, 12 h. [b]Isolated yield of **3a**. [c]The er values were determined *via* HPLC on chiral stationary phase. [d]Using 150 mg 4 Å MS as additive. [e]Using DQ B as oxidant. [f]Using BQ as oxidant. DIPEA = *N*, *N*-Diisopropylethylamine. THF = Tetrahydrofuran. EA = Ethyl acetate. DCE = Dichloroethane. DMSO = Dimethyl sulfoxide. NMP = *N*-Methylpyrrolidone. MTBE = Methyl tert-butyl ether.

General procedure for the catalytic reactions

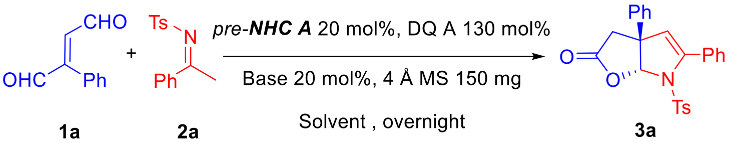

1a **2a** **3a**

Supplementary Figure 3. General procedure for the catalytic reactions of aryl aldehyde **1a** and imine **2a** to synthesize product **3a**.

To a dry 4.0 mL vial equipped with a magnetic stir bar, was added **1a** (0.13 mmol), **2a** (0.10 mmol), pre-NHC **A** (0.02 mmol), DQ A (0.13 mmol) and base (0.02 mmol). After purges with N_2 in glove-box, anhydrous MeCN (2.0 mL), and 4 Å MS (150.0 mg) was added and sealed. The reaction mixture was stirred at 45 °C for 12 hours. Then the mixture was directly concentrated under reduced pressure to afford a crude product. The crude product was purified via column chromatography on silica gel (petroleum ether / ethyl acetate = 5 / 1) to afford the desired product **3a**.

Synthetic transformations of chiral products 3a:

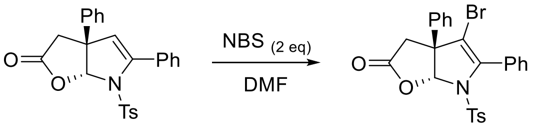

3a 99:1 er **5** 87% 99:1 er

Supplementary Figure 4. Preparation of **5** from product **3a**.

Compound **3a** (42.9 mg, 0.10 mmol) was dissolved in DMF (10.0 mL) and NBS (35.6 mg, 0.20 mmol, 2 eq) was added. The mixture was stirred at room temperature for 4 hours until the consumption of the **3a** (monitored by TLC). The reaction was then quenched by the slow addition of water, extracted with ethyl acetate. The combined organic phases were dried over anhydrous Na_2SO_4, concentrated under reduced pressure to give the crude product. The crude product was purified via SiO_2 flash chromatography (petroleum ether / ethyl acetate = 5:1) to afford product **5** (44.5 mg, 87% yield, 99:1 er) as a white solid.

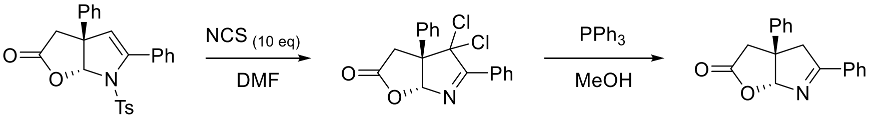

3a 99:1 er **6** 73% 99:1 er **7** 60% 99:1 er

Supplementary Figure 5. Preparation of **6,7** from product **3a**.

Compound **3a** (42.9 mg, 0.10 mmol) was dissolved in DMF (10.0 mL) and NCS (133.5 mg, 1.00 mmol, 10 eq) was added. The mixture was stirred at room temperature for 4 hours until the consumption of the **3a** (monitored by TLC). The reaction was then quenched by the slow addition of water, extracted

with ethyl acetate. The combined organic phases were dried over anhydrous Na_2SO_4, concentrated under reduced pressure. The crude product was purified via SiO_2 flash chromatography (petroleum ether / ethyl acetate = 5:1) to afford product **6** (25.3 mg, 73% yield, 99:1 er) as a white solid.

Compound **6** (34.6 mg, 0.10 mmol) was dissolved in MeOH (10.0 mL) and PPh_3 (262.3 mg, 1.00 mmol) was added. The mixture was stirred at room temperature for 8 hours until the consumption of the **6** (monitored by TLC). The combined organic phases concentrated under reduced pressure. The crude product was purified via SiO_2 flash chromatography (petroleum ether / ethyl acetate = 5:1) to afford product **7** (16.6 mg, 60% yield, 99:1 er) as a white solid.

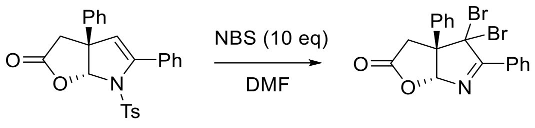

3a 99:1 er   **8** 88% 99:1 er

Supplementary Figure 6. Preparation of **8** from product **3a**.

Compound **3a** (42.9 mg, 0.10 mmol) was dissolved in DMF (10.0 mL) and NBS (177.9 mg, 1.00 mmol) was added. The mixture was stirred at room temperature for 4 hours until the consumption of **3a**. The reaction was then quenched by the slow addition of water, extracted with ethyl acetate. The combined organic phases were dried over anhydrous Na_2SO_4, concentrated under reduced pressure. The crude product was purified via SiO_2 flash chromatography (petroleum ether / ethyl acetate = 5:1) to afford product **8** (38.3 mg, 88% yield, 99:1 er) as a white solid.

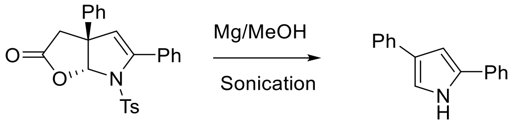

3a 99:1 er   **9** 76% Yield

Supplementary Figure 7. Preparation of **9** from product **3a**.

Under N_2 atmosphere, to an oven-dried Schlenk tube equipped with a magnetic stir bar, was added the compound **3a** (42.9 mg, 0.10 mmol) and Mg-powder (6.0 mg, 0.50 mmol). Absolute MeOH (2.0 mL) was added and the reaction was capped with a septum, then sonicated at room temperature until no more starting material was remained monitored by TLC (around 45 minutes). After cooled to 0 °C, the reaction mixture was slowly quenched with saturated NH_4Cl. The aqueous phases were extracted with DCM (3 × 10.0 mL). The combined organic phases were washed with saturated brine (20.0 mL), then dried over anhydrous Na_2SO_4, concentrated under reduced pressure. The residue was purified by silica gel column chromatography (petroleum ether / ethyl acetate = 5:1) to afford product **9** (16.1 mg, white solid 76% yield).

DFT Calculations of oxidation selectivity

The initial cluster structures were optimized using M06-2X-D3 with def2SVP basis set for all atoms as implemented in Gaussian 16 (rev. A.03)[1]. Single point (SP) corrections were performed using M06-2X-D3 functional and def2-TZVPP basis set for all atoms. Minima and transition structures on the potential energy surface (PES) were confirmed as such by harmonic frequency analysis, showing respectively zero and one imaginary frequency. The implicit IEFPCM continuum solvation model for MeCN solvent was used to account for the effect of solvent on the potential energy surface. The thermal energy terms associated with various thermal motions of the molecule were calculated from the frequency analysis output using Shermo[2], with scale factors[3] applied to adjust for the errors arising from the harmonic oscillation assumption. Noncovalent interactions were analyzed by using Multiwfn[4]. The three-dimensional structures were illustrated by using VMD[5,6] and CYLview20[7].

Mechanistic studies ESI-HRMS analysis of the catalyst system

LC-HRMS semi-quantitative intensity analysis of Breslow intermediate

To a dry Schlenk tube equipped with a magnetic stir bar, was added **1a(D)** (0.10 mmol), pre-NHC **A** (0.02 mmol) and base (0.02 mmol). After purges with N_2 in glove-box, anhydrous MeCN (2.0 mL), and 4 Å MS (150.0 mg) was added and sealed. The reaction mixture was then stirred at 45 °C in oil bath for 4 hours. It was found that pre-NHC directly reacted with **1a(D)** to form product **1a(D)-I**, and **1a(D)-I'** which was determined by liquid chromatography-high resolution mass spectrum (LC-HRMS). These numbers are semi-quantitative, and can provide certain insights on the reaction mechanism.

The intensity of product was detected by the Waters BEH-C_{18} column of length 100.0 mm, 2.1 mm internal diameter and 1.7 μm particle thickness. 10% acetonitrile, 90% water as the initial mobile phase, gradient elution until 100% acetonitrile (from 0.0 min to 35.0 min), and the flow rate of the mobile phase was 300.0 μL/min throughout the gradient. Column temperatures were tested at 40 °C. The samples were taken 5.0 μL from the reaction solution with a micro syringe and diluted to 100.0 μL with pure acetonitrile and analyzed in full scan mode in the mass range of 50 to 1500 m/z. We repeated each experiment three times under the same conditions.

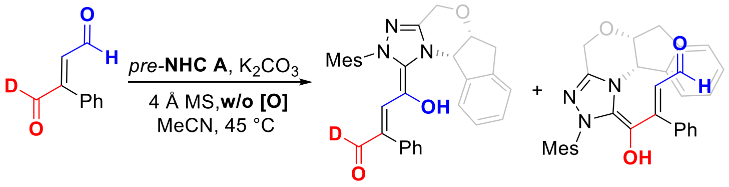

1a(D) **1a(D)-I (32-47%)** **1a(D)-I' (53-68%)**

Supplementary Figure 8. Intensity of Breslow intermediate.

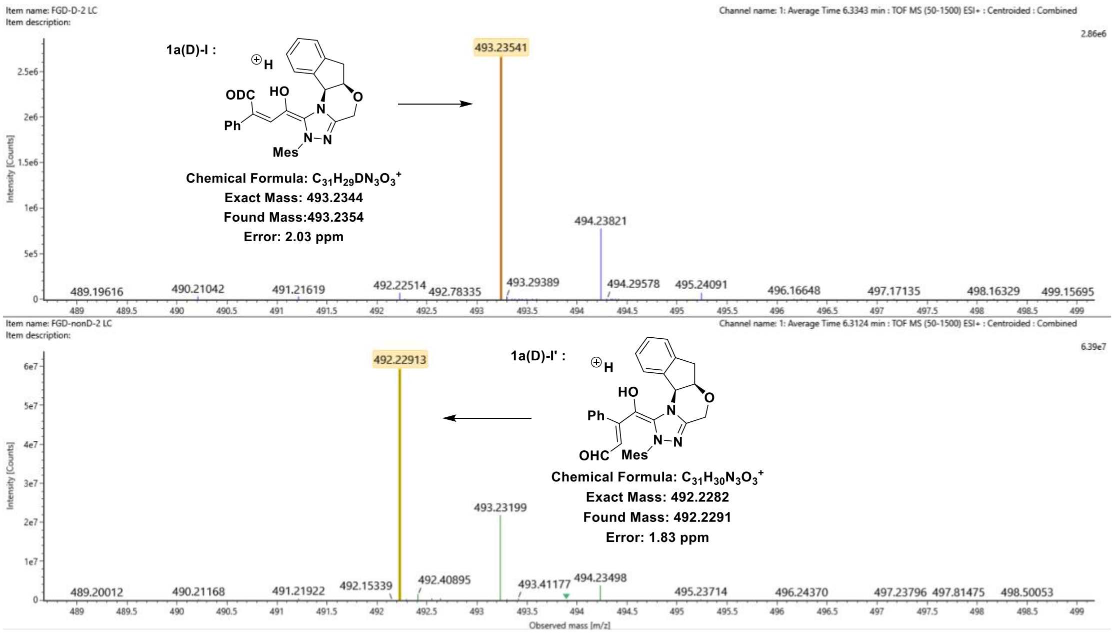

Supplementary Figure 9. LC-HRMS analysis of Breslow intermediate.

001: [1a(D)-I', 68%]: [1a(D)-I, 32%]

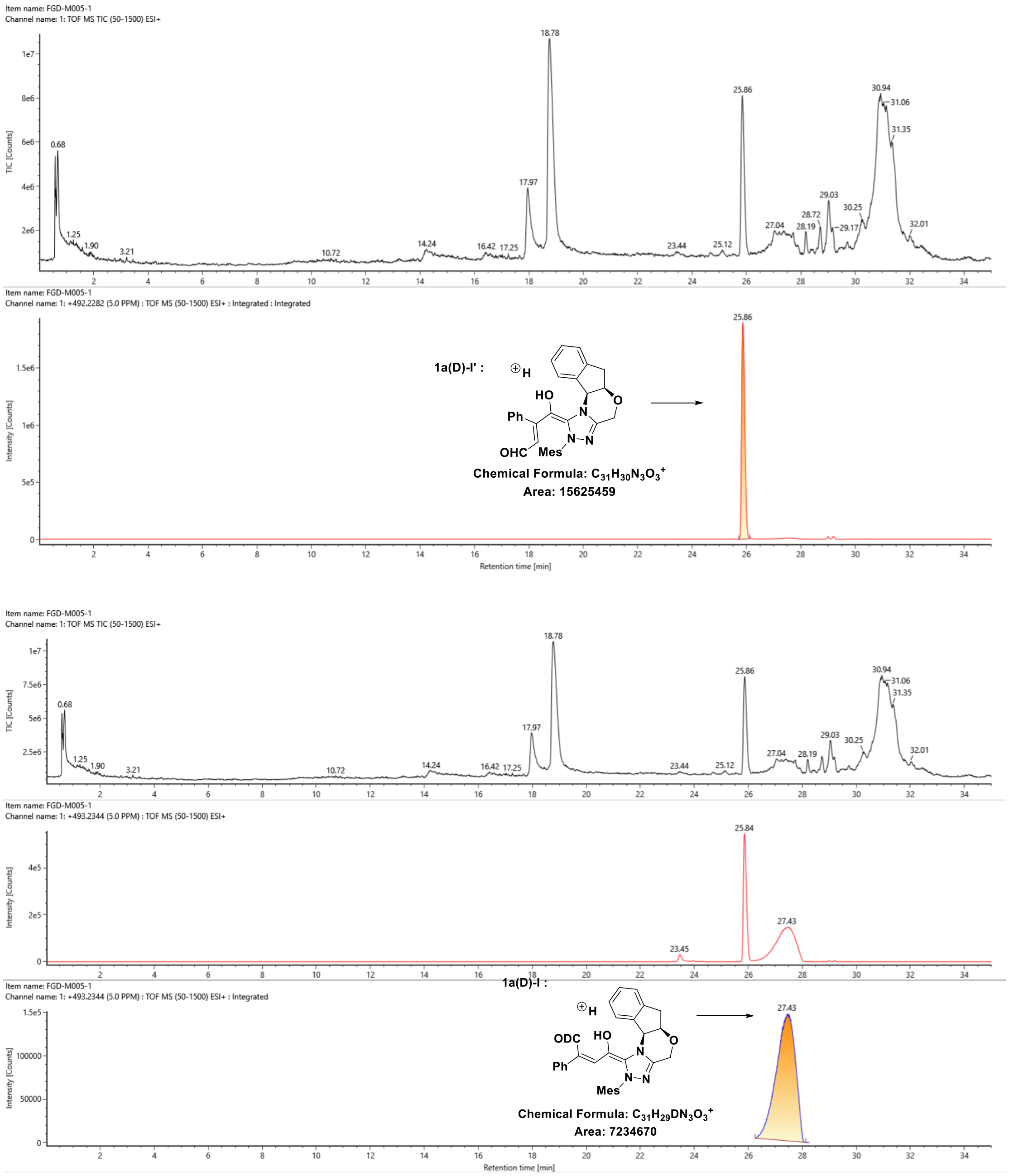

Supplementary Figure 10. LC-HRMS semi-quantitative intensity analysis of Breslow intermediate.

002: [1a(D)-I', 57%]: [1a(D)-I, 43%]

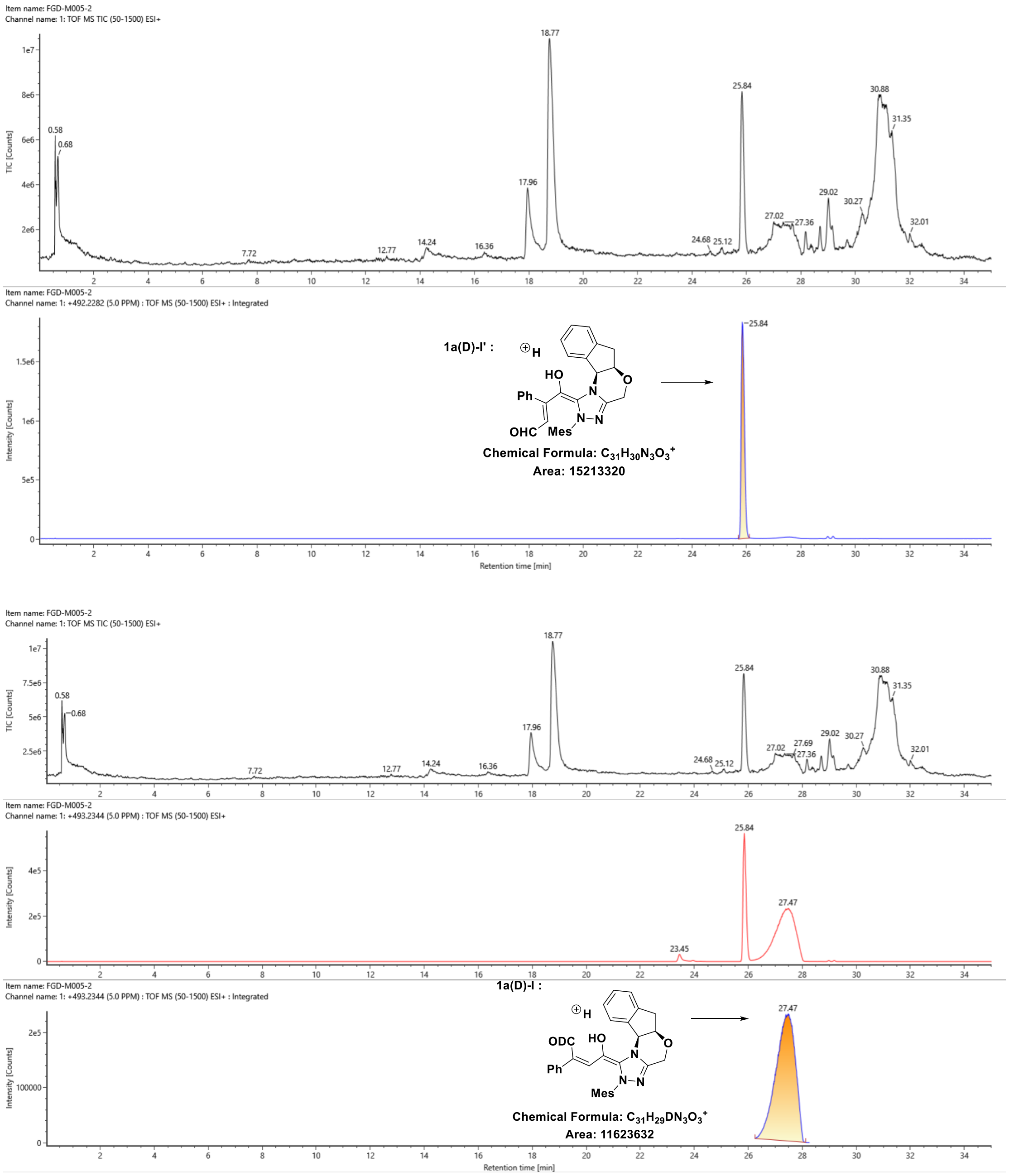

Supplementary Figure 11. LC-HRMS semi-quantitative intensity analysis of Breslow intermediate.

003: [1a(D)-I', 53%]: [1a(D)-I, 47%]

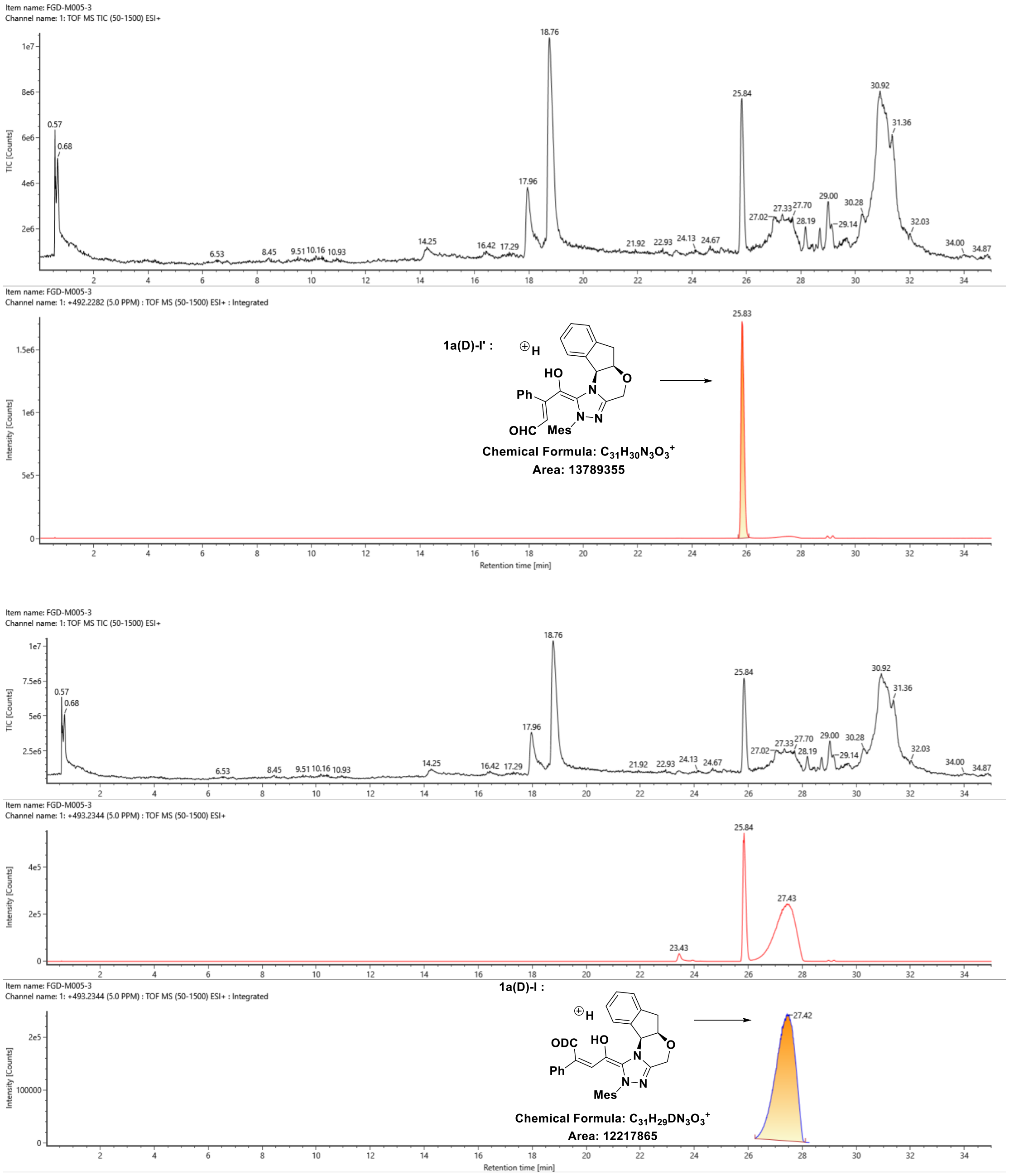

Supplementary Figure 12. LC-HRMS semi-quantitative intensity analysis of Breslow intermediate.

LC-HRMS semi-quantitative intensity analysis of Acylazolium intermediate

To a dry Schlenk tube equipped with a magnetic stir bar, was added **1a(D)** (0.10 mmol), pre-NHC **A** (0.02 mmol), **[O]** (0.10 mmol), MeOH (10.00 mmol) and base (0.02 mmol). After purges with N_2 in glove-box, anhydrous MeCN (2.0 mL), and 4 Å MS (150.0 mg) was added and sealed. The reaction mixture was then stirred at 45 °C in oil bath for 4 hours. It was found that pre-NHC directly reacted with **1a(D)** to form product **10**, and **11** which was determined by liquid chromatography-high resolution mass spectrum (LC-HRMS). These numbers are semi-quantitative, and can provide certain insights on the reaction mechanism.

The intensity of product was detected by the Waters BEH-C_{18} column of length 50.0 mm, 2.1 mm internal diameter and 1.7 μm particle thickness. 10% acetonitrile, 90% water as the initial mobile phase, gradient elution until 100% acetonitrile (from 0.0 min to 15.0 min), and the flow rate of the mobile phase was 300.0 μL/min throughout the gradient. Column temperatures were tested at 40 °C. The samples were taken 5.0 μL from the reaction solution with a micro syringe and diluted to 100.0 μL with pure acetonitrile and analyzed in full scan mode in the mass range of 50 to 1500 m/z. We repeated each experiment three times under the same conditions. We repeated each experiment three times under the same conditions.

Supplementary Table 2. LC-HRMS semi-quantitative intensity analysis of Acylazolium intermediate[a].

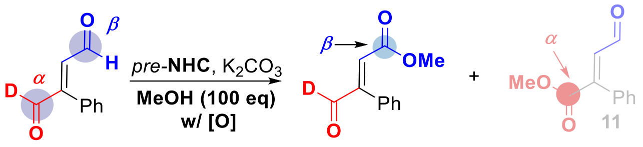

1a(D) **10** 11

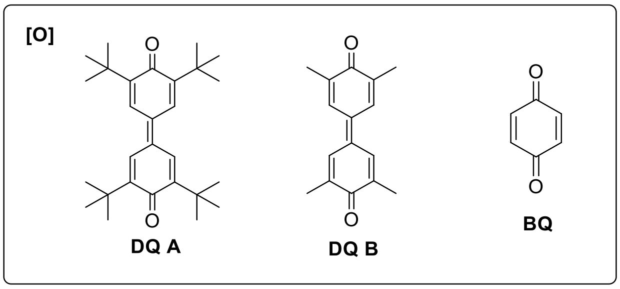

Entry	[O]	10:11[b]
1	**DQ A**	99:1
2	**DQ B**	98:2
3	**BQ**	97:3

[a] Unless otherwise specified, the reactions were conducted with **1a** (0.10 mmol), pre-NHC (0.02 mmol), base (0.02 mmol), [O] (0.10 mmol), MeOH (10.00 mmol) and solvents (2.0 mL) at 45 °C for 4 h. [b] The radio of **10:11** was determined by liquid chromatography-high resolution mass spectrum (LC-HRMS).

[O]: DQ A 10 (99%):11 (1%)

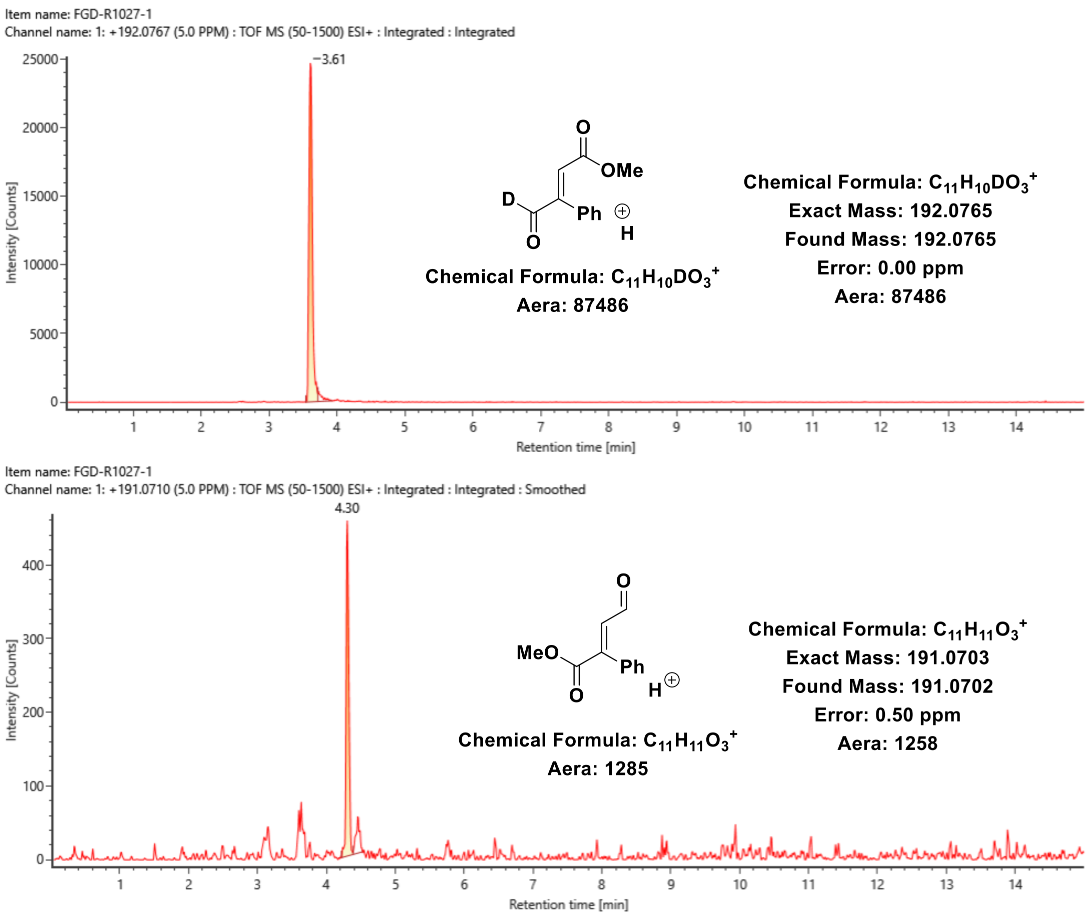

Supplementary Figure 13. LC-HRMS semi-quantitative intensity analysis of Acylazolium intermediate (DQ A).

[O]: DQ B 10 (98%):11 (2%)

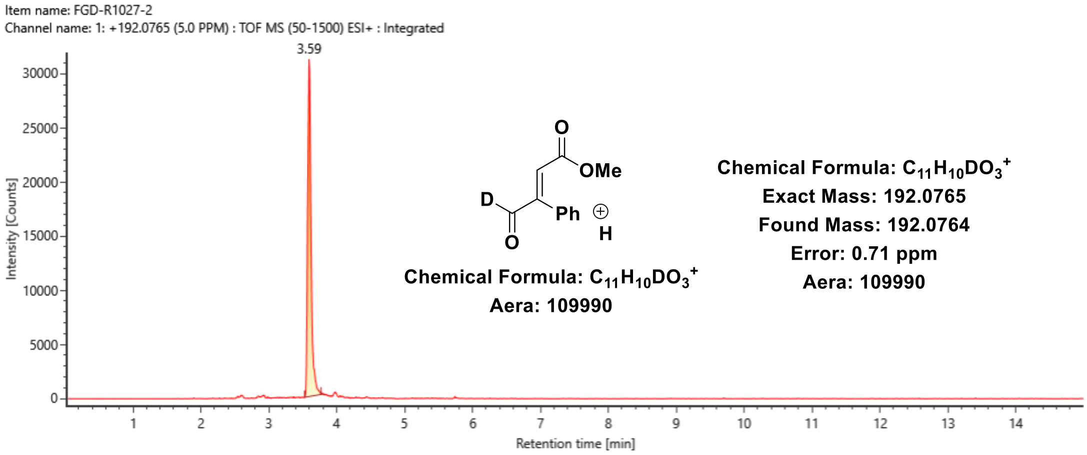

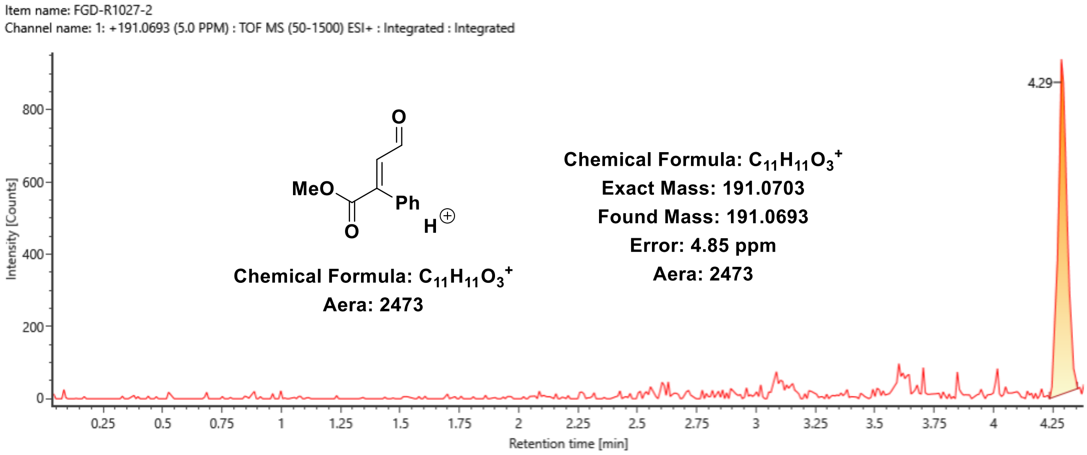

Supplementary Figure 14. LC-HRMS semi-quantitative intensity analysis of Acylazolium intermediate (DQ B).

[O]: BQ 10 (97%):11 (3%)

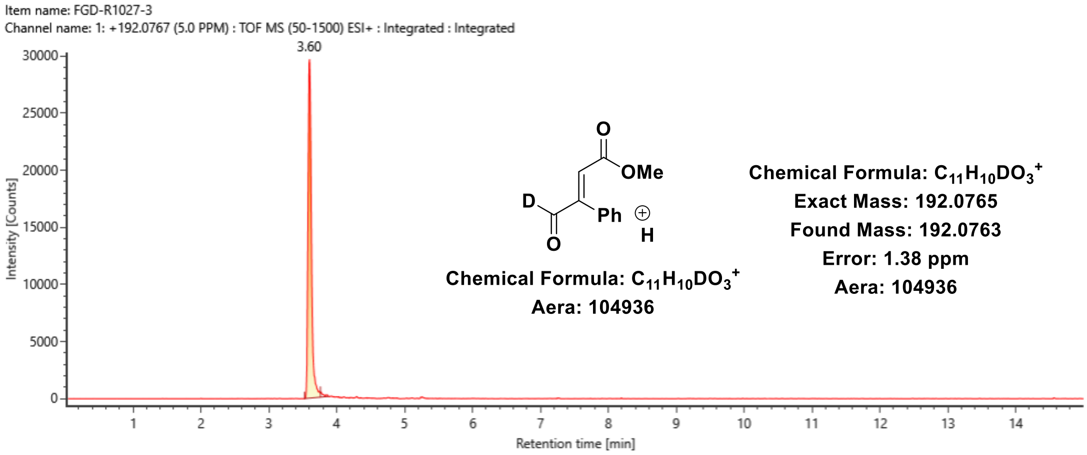

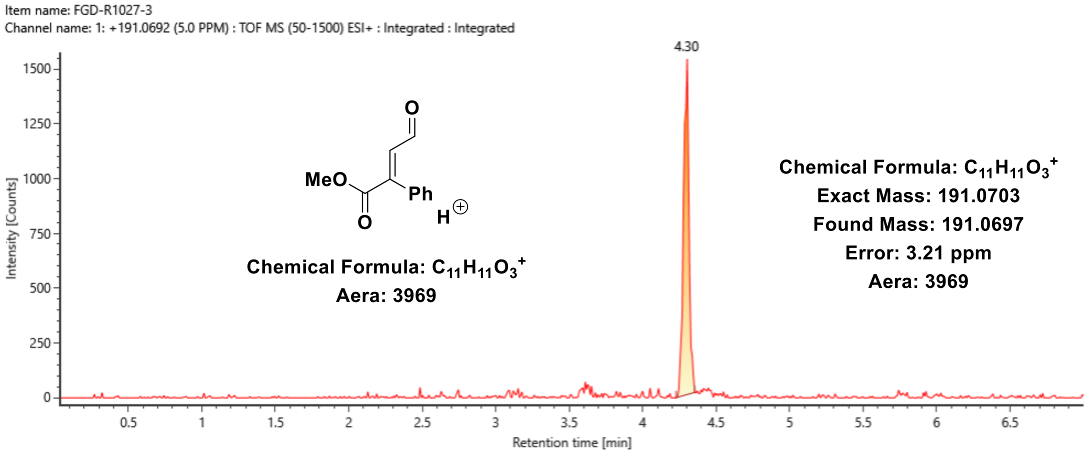

Supplementary Figure 15. LC-HRMS semi-quantitative intensity analysis of Acylazolium intermediate (BQ).

LC-HRMS semi-quantitative intensity analysis of possible polymerized product

To a dry Schlenk tube equipped with a magnetic stir bar, was added **1a** (0.10 mmol), pre-NHC **A** (0.02 mmol), DQ A (0.10 mmol) and base (0.02 mmol). After purges with N_2 in glove-box, anhydrous MeCN (2.0 mL), and 4 Å MS (150.0 mg) was added and sealed. The reaction mixture was then stirred at 45 °C in oil bath for 4 hours. Then to a dry Schlenk tube equipped with a magnetic stir bar, was added **1a(D)** (0.10 mmol), pre-NHC **A** (0.02 mmol), DQ A (0.10 mmol) and base (0.02 mmol). After purges with N_2 in glove-box, anhydrous MeCN (2.0 mL), and 4 Å MS (150.0 mg) was added and sealed. The reaction mixture was then stirred at 45 °C in oil bath for 4 hours. It was found that pre-NHC directly reacted with **1a** and **1a (D)** to form by-products which was determined by liquid chromatography-high resolution mass spectrum (LC-HRMS). These numbers are semi-quantitative, and can provide certain insights on the reaction mechanism.

The intensity of product was detected by the Waters BEH-C_{18} column of length 50.0 mm, 2.1 mm internal diameter and 1.7 μm particle thickness. 10% acetonitrile, 90% water as the initial mobile phase, gradient elution until 100% acetonitrile (from 0.0 min to 40.0 min), and the flow rate of the mobile phase was 300.0 μL/min throughout the gradient. Column temperatures were tested at 40 °C. The samples were taken 10.0 μL from the reaction solution with a micro syringe and diluted to 100.0 μL with pure acetonitrile and analyzed in full scan mode in the mass range of 50 to 1500 m/z.

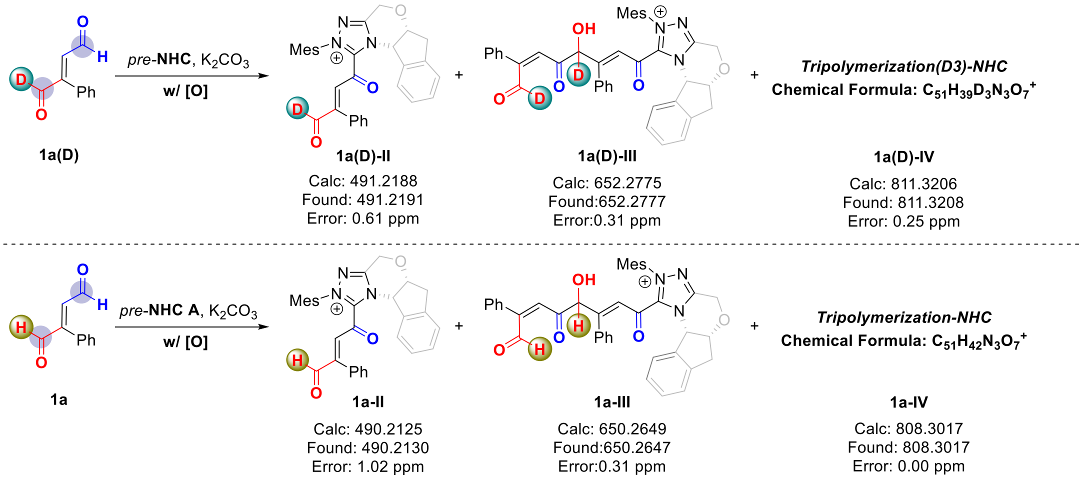

Supplementary Figure 16. LC-HRMS analysis of possible polymerized product.

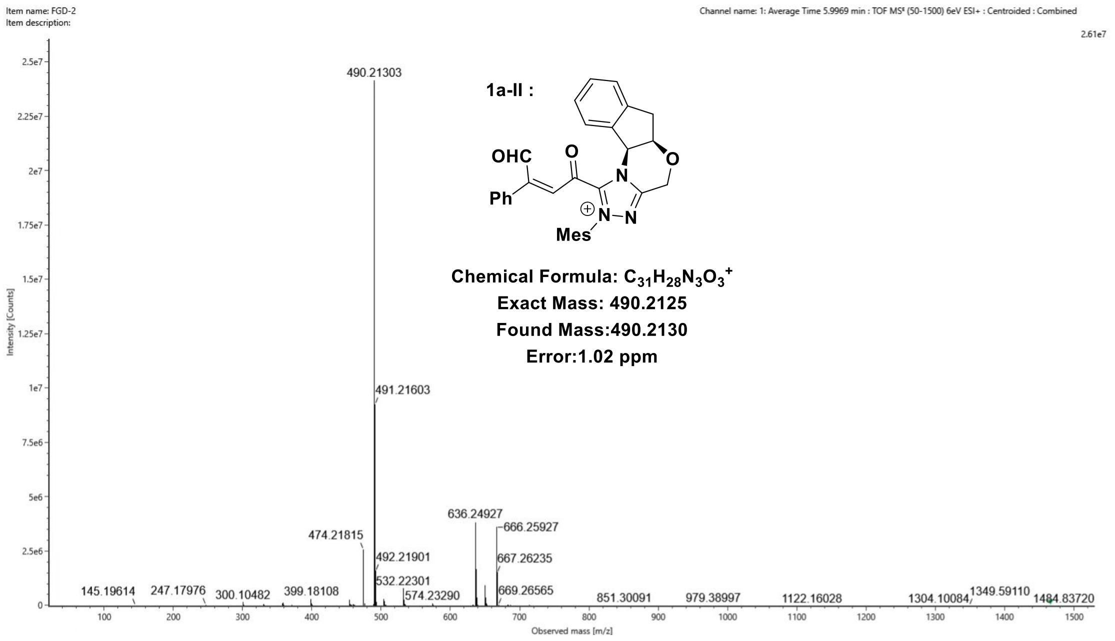

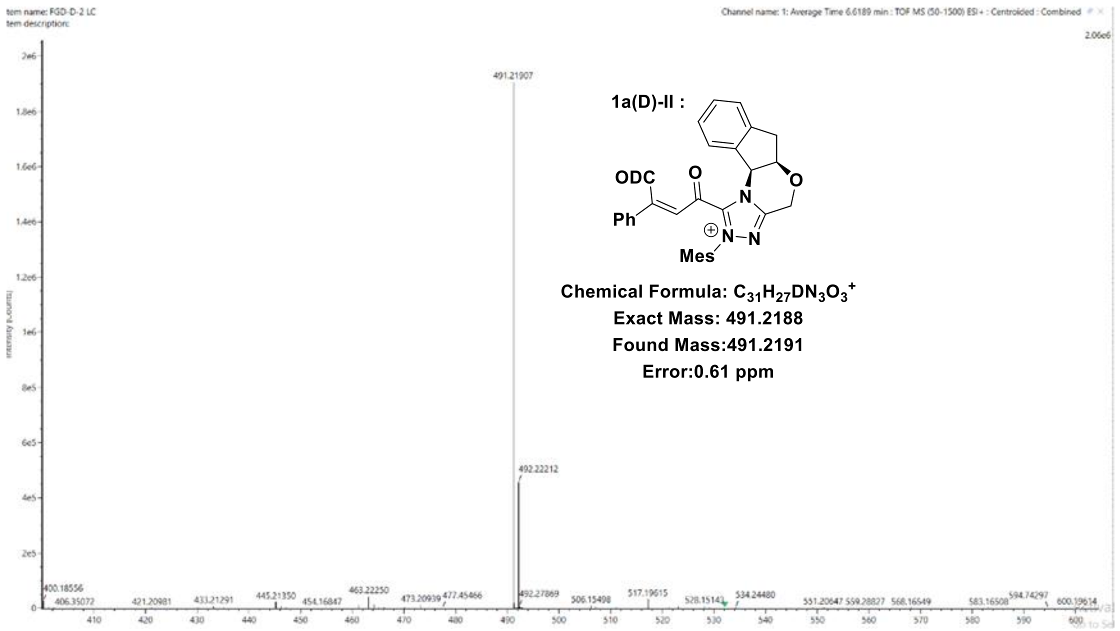

Supplementary Figure 17. HRMS analysis of possible polymerized product.

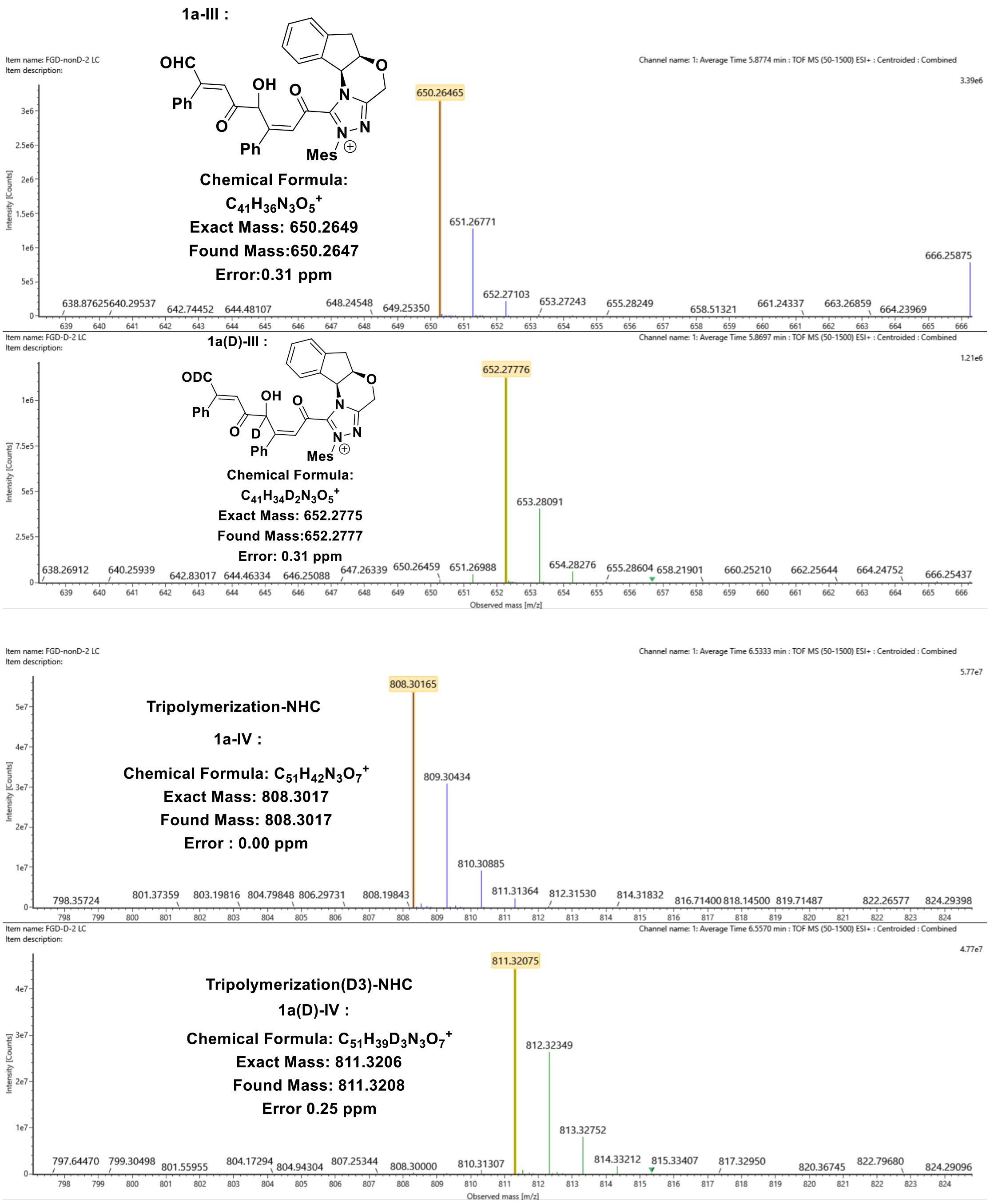

Supplementary Figure 18. HRMS analysis of possible polymerized product.

LC-HRMS-MS semi-quantitative intensity analysis of possible polymerized product

To a dry Schlenk tube equipped with a magnetic stir bar, was added **1a** (0.10 mmol), pre-NHC **A** (0.02 mmol), DQ A (0.10 mmol) and base (0.02 mmol). After purges with N_2 in glove-box, anhydrous MeCN (2.0 mL), and 4 Å MS (150.0 mg) was added and sealed. The reaction mixture was then stirred at 45 °C in oil bath for 4 hours. Then to a dry Schlenk tube equipped with a magnetic stir bar, was added **1a** (0.10 mmol), pre-NHC **A** (0.02 mmol), DQ A (0.10 mmol) and base (0.02 mmol). After purges with N_2 in glove-box, anhydrous MeCN (2.0 mL), and 4 Å MS (150.0 mg) was added and sealed. The reaction mixture was then stirred at 45 °C in oil bath for 4 hours. It was found that pre-NHC directly reacted with **1a** to form by-products which was determined by liquid chromatography-high resolution-accurate mass spectrometer (LC-HRMS-MS). Selected **1a-IV** as the parent ion, retention intensity 30%. These numbers are semi-quantitative, and can provide certain insights on the reaction mechanism.

The intensity of product was detected by the Waters BEH-C_{18} column of length 50.0 mm, 2.1 mm internal diameter and 1.7 μm particle thickness. 10% acetonitrile, 90% water as the initial mobile phase, gradient elution until 100% acetonitrile (from 0.0 min to 15.0 min), and the flow rate of the mobile phase was 300.0 μL/min throughout the gradient. Column temperatures were tested at 40 °C. The samples were taken 5.0 μL from the reaction solution with a micro syringe and diluted to 100.0 μL with pure acetonitrile and analyzed in full scan mode in the mass range of 50 to 1500 m/z. We repeated each experiment three times under the same conditions. We repeated each experiment three times under the same conditions.

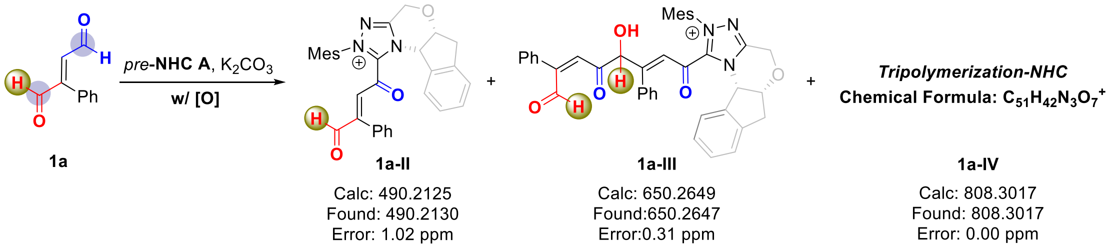

Supplementary Figure 19. LC-HRMS-MS analysis of possible polymerized product.

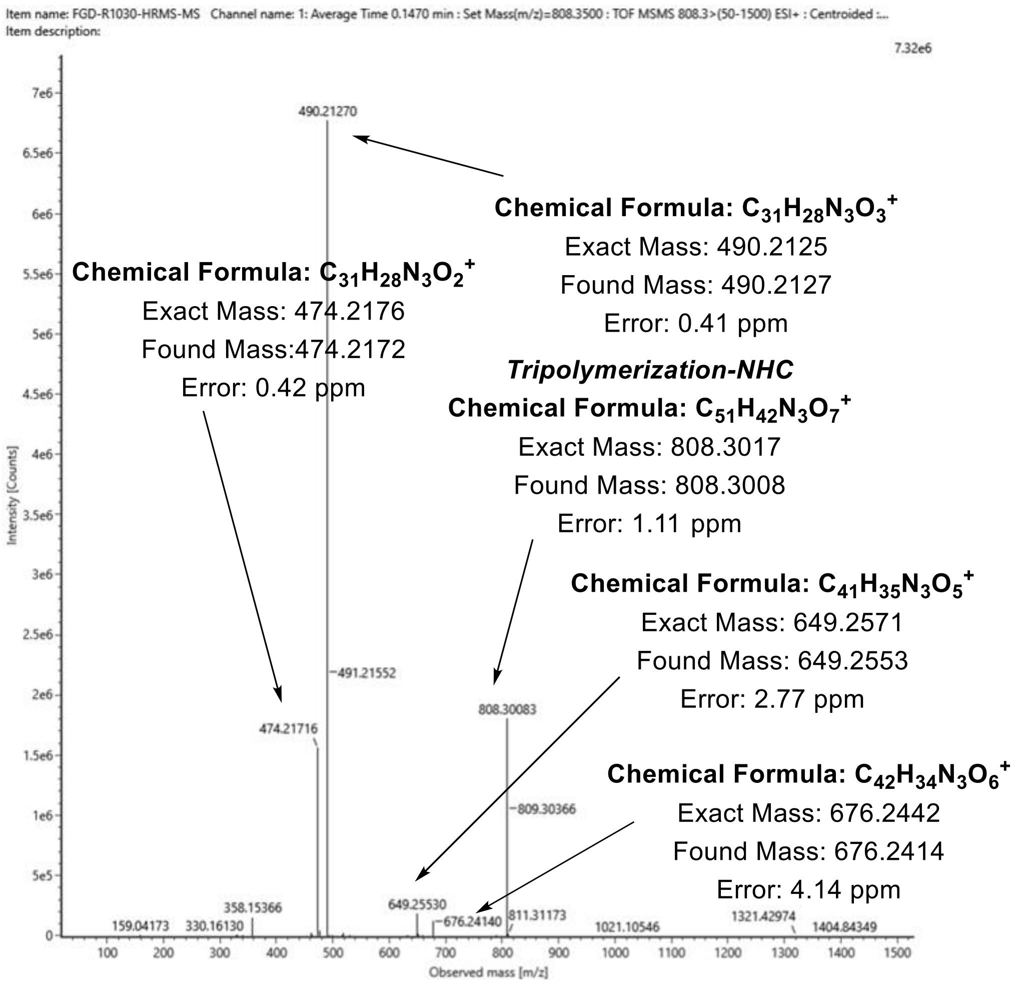

Supplementary Figure 20. LC-HRMS-MS analysis of possible polymerized product.

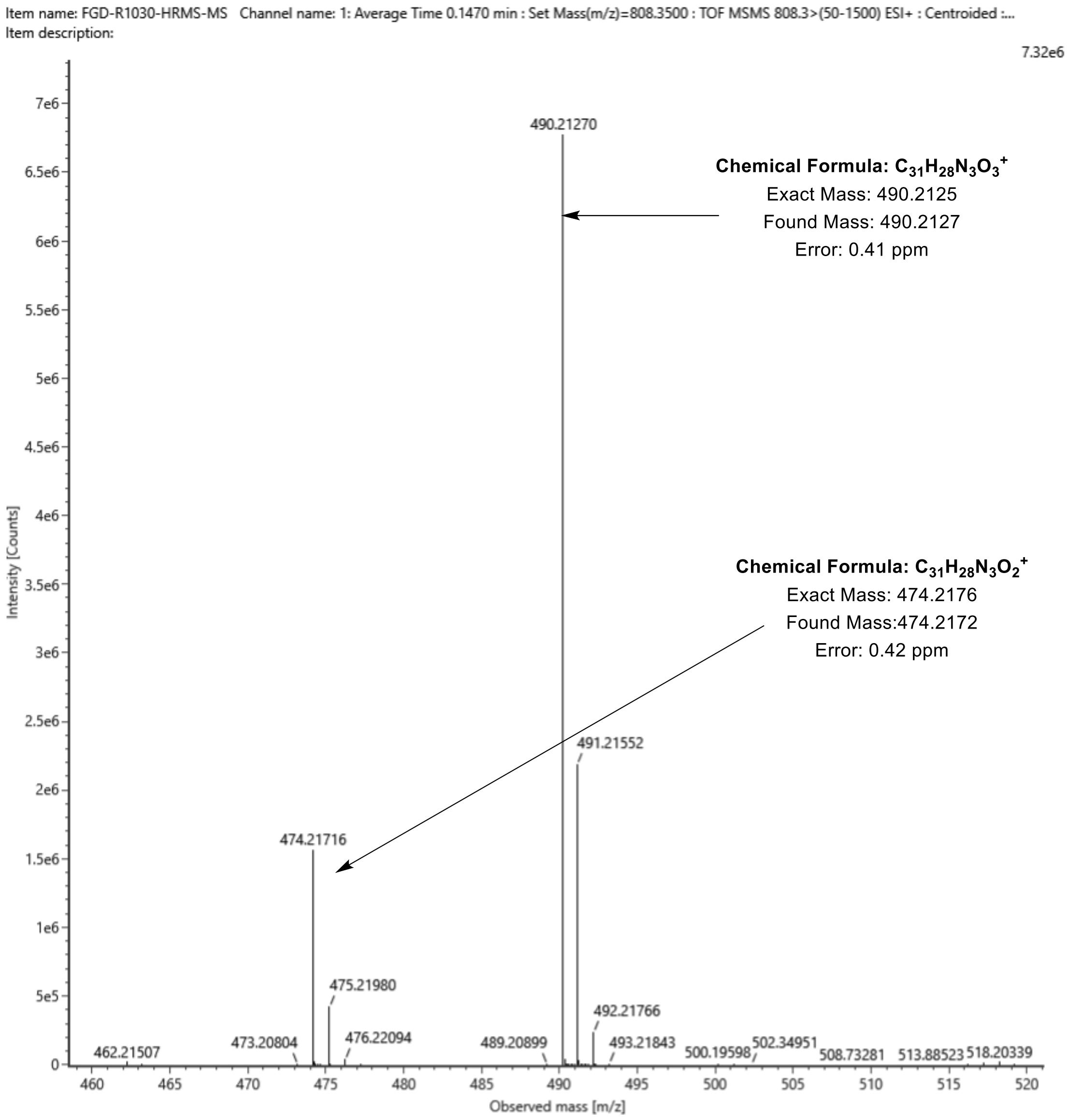

Supplementary Figure 21. LC-HRMS-MS analysis of possible polymerized product.

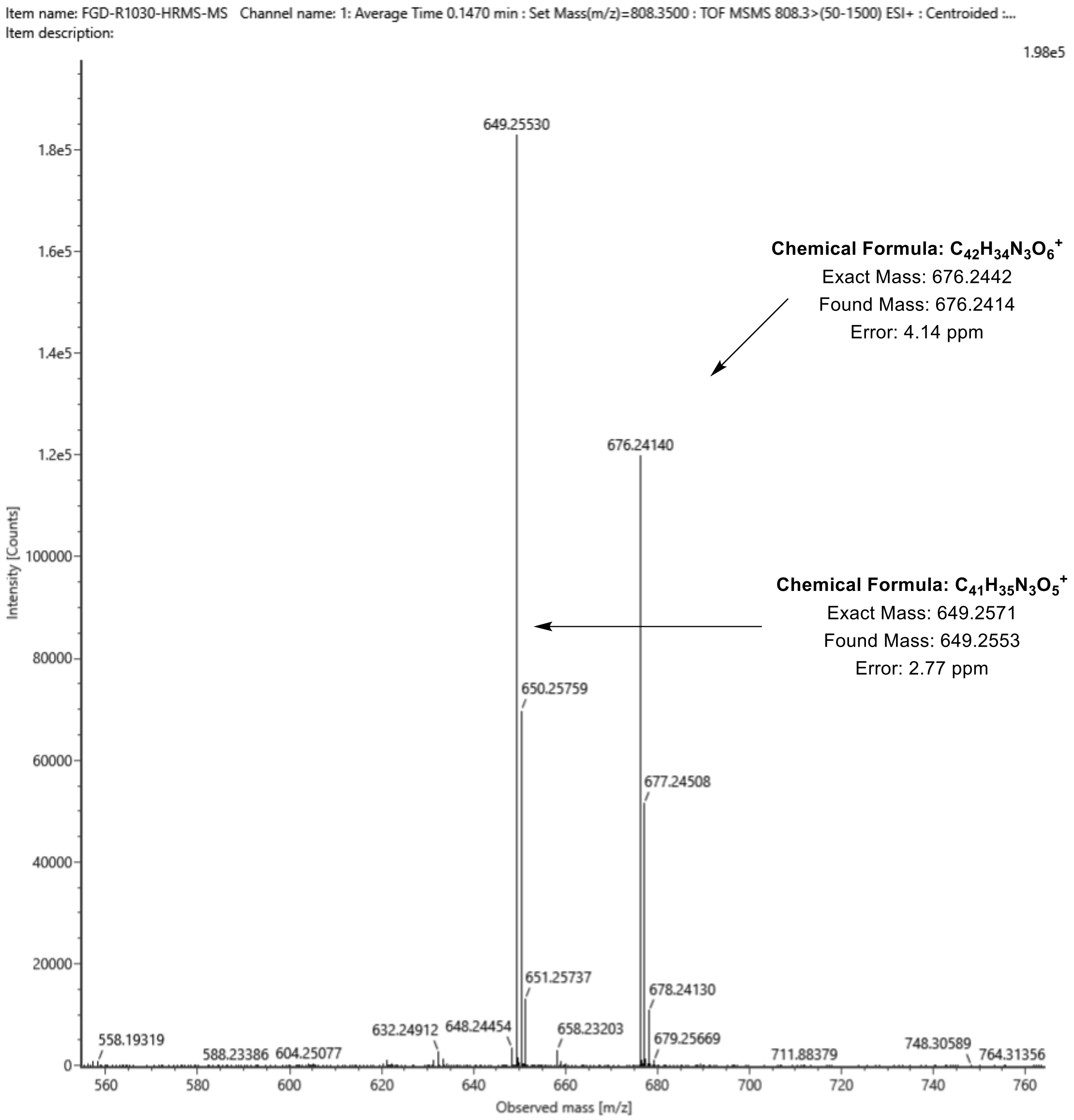

Supplementary Figure 22. LC-HRMS-MS analysis of possible polymerized product.

Antibacterial activity in Vitro *(Xac)*

Antibacterial activities of the title compounds against *Xanthomonas axonopodis* pv. *citri (Xac)* were evaluated by using the turbidimeter test. Thiodiazole-copper and Bismerthiazol were used as the positive controls. The compound was dissolved in 150.0 μL of dimethyl sulfoxide and diluted with 0.1% (V / V) Tween-80 to prepare the solutions of two concentrations (100 and 50 μg/mL). 1.0 mL of the above solution was added to the non-toxic nutrient broth (NB: 1.5 g of beef extract, 2.5 g of peptone, 0.5 g of yeast powder, 5.0 g of glucose and 500 mL of distilled water, pH = 7.0 ~ 7.2) liquid medium in a 4.0 mL tube. Then, 40.0 μL of NB solution containing *Xanthomonas Campestris* pv. *citri (Xac)* was added to 5.0 mL of the NB solution containing the test compound. The inoculated test tube was incubated at (28 ± 1) °C under continuous shaking at 200 rpm for 24 h. Each process is repeated 3 times. The culture growth was monitored by measuring the optical density at 595 nm (OD595) and expressed as corrected turbidity. The relative inhibitory rate was calculated as follows:

I (%) = (C − T) / C × 100%

C: the corrected turbidity value of bacterial growth on untreated NB;

T: the corrected turbidity value of bacterial growth on treated NB;

I: The relative inhibitory rate.

Supplementary Table 3. In vitro antibacterial activity of the target compounds against *Xac* [a].

Compounds	*Xanthomonas axonopodis* pv. *citri (Xac)* Inhibition rate / %	
**3a**	57.15±1.21 (230 μM/L)	26.77±2.01 (120 μM/L)
**3a(D)**	56.94±1.15 (230 μM/L)	30.27±2.45 (120 μM/L)
**3b**	95.81±4.01 (220 μM/L)	37.96±1.17 (110 μM/L)
**3d**	46.24±0.40 (220 μM/L)	33.23±0.17 (110 μM/L)
**3e**	50.97±0.35 (220 μM/L)	33.28±1.82 (110 μM/L)
**3f**	46.40±0.85 (220 μM/L)	30.65±0.66 (110 μM/L)
**3g**	46.67±7.18 (210 μM/L)	35.91±0.21 (110 μM/L)
**3i**	97.42±0.80 (220 μM/L)	35.70±1.50 (110 μM/L)
**4d**	73.06±1.97 (220 μM/L)	28.01±6.41 (110 μM/L)
**4f**	55.86±4.89 (220 μM/L)	36.45±7.58 (110 μM/L)
**4i**	90.48±2.21 (220 μM/L)	53.66±0.87 (110 μM/L)
**4k**	67.31±0.40 (220 μM/L)	50.86±2.48 (110 μM/L)
**4q**	65.48±0.82 (240 μM/L)	40.27±4.29 (120 μM/L)
**4r**	27.80±1.05 (230 μM/L)	23.98±0.84 (110 μM/L)
**5**	68.92±0.68 (200 μM/L)	36.40±5.95 (100 μM/L)
**6**	89.62±1.32 (290 μM/L)	80.43±5.56 (140 μM/L)
**7**	94.78±0.42 (360 μM/L)	80.16±0.40 (180 μM/L)
**8**	92.31±5.16 (230 μM/L)	39.35±2.61 (110 μM/L)
**Thiodiazole copper[b]**	57.11±4.48 (300 μM/L)	27.90±2.14 (150 μM/L)
**Bismerthiazol[b]**	94.52±1.84 (360 μM/L)	55.22±3.44 (180 μM/L)

[a]All data were average data of three replicates. [b]Commercial bactericide, used as the positive control.

II. Supplementary Discussions

Postulated reaction mechanism

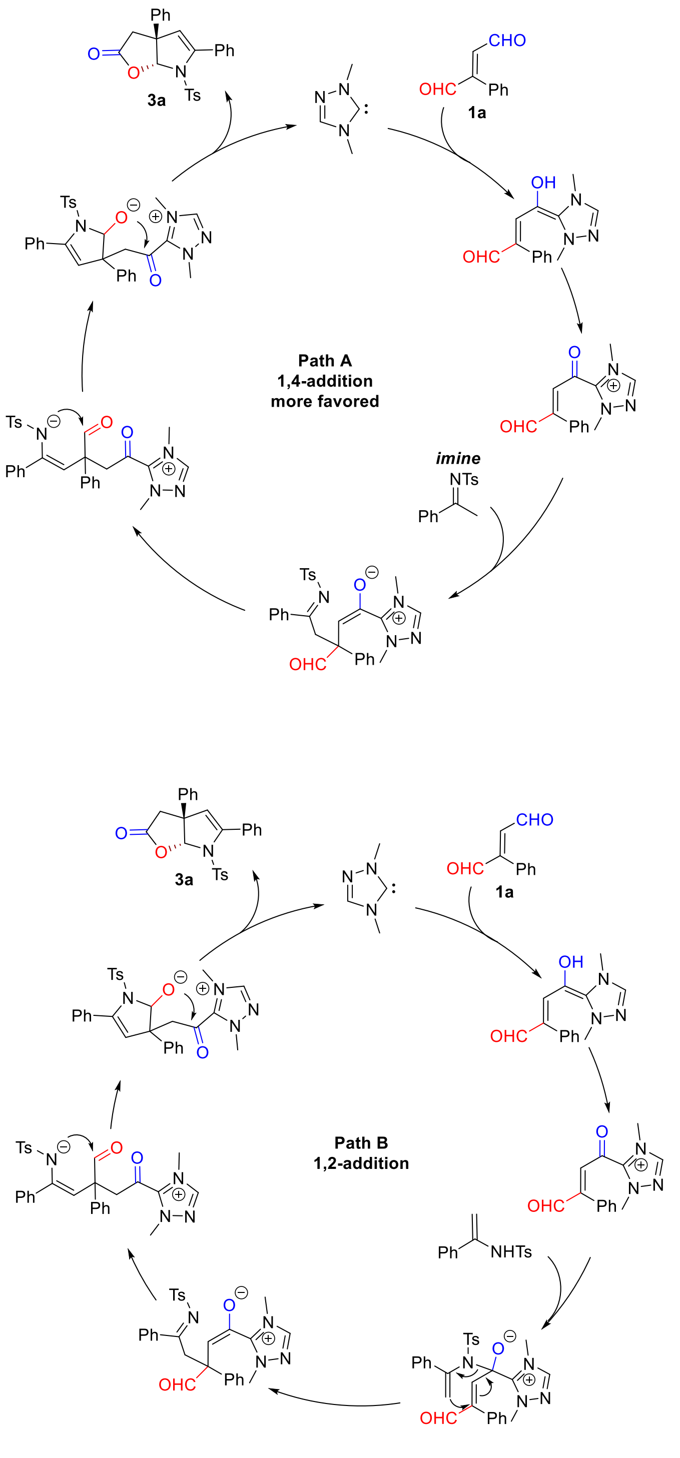

Supplementary Figure 23. Postulated reaction mechanism.

The tautomerism of imines with enamines could be found in DMSO(*d-6*) by ^{1}H NMR. However, switching DMSO(*d-6*) to CD_3CN solvents, the tautomerism of imines with enamines cannot be observed by ^{1}H NMR (0.1 mmol imine and 0.02 mmol additive were dissolved in 1ml CD_3CN, absorbed 0.7 ml the mixture was detected by ^{1}H NMR). Furthermore, multiple metal salts (such as K_2CO_3, $KHCO_3$, and KBF_4) was added to the corresponding solvent, ^{1}H NMR was collected.

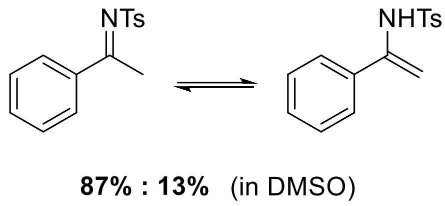

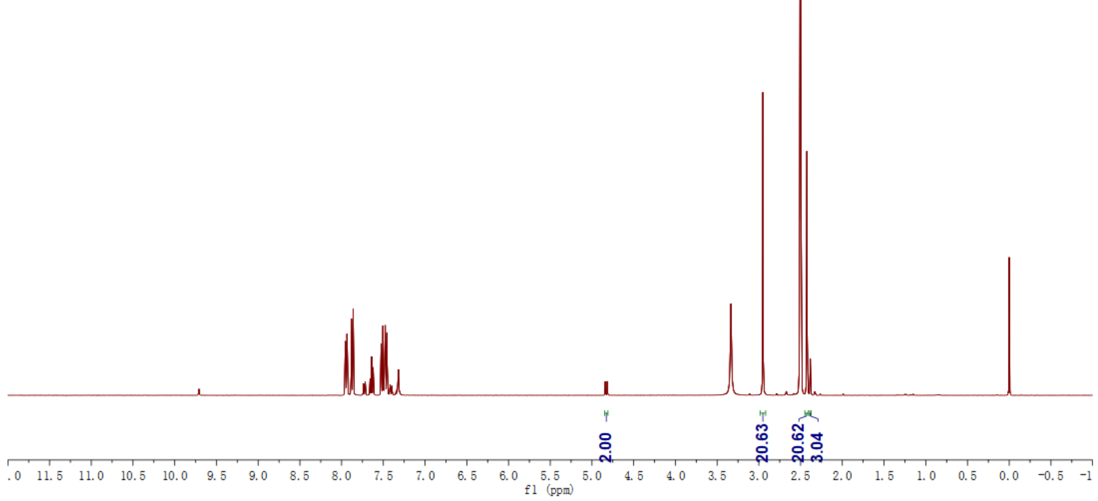

Supplementary Figure 24. 2a in DMSO (d-6) ^{1}H NMR (400 MHz)

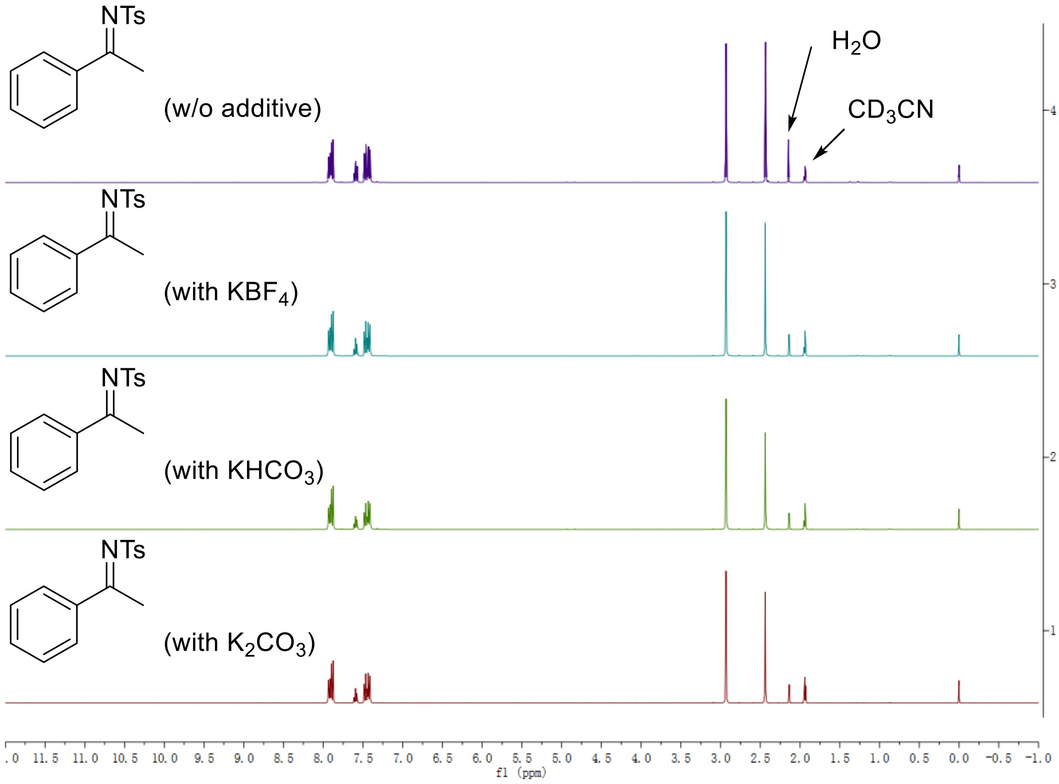

Supplementary Figure 25. 2a in CD_3CN (with additive). ^1H NMR (400 MHz)

III. Supplementary Notes

Characterization of substrates and products

1. Characterization of substrates

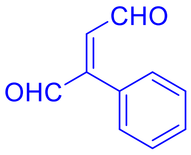

1a

2-phenylfumaraldehyde (1a)

Yellow solid, 50% yield, 0.8 g, m.p. 87-88 °C.

^{1}H NMR (400 MHz, CDCl$_3$) δ 9.96 (s, 1H), 9.91 (d, J = 7.6 Hz, 1H), 7.57 – 7.43 (m, 3H), 7.37 – 7.30 (m, 2H), 6.72 (d, J = 7.6 Hz, 1H).

^{13}C NMR (101 MHz, CDCl$_3$) δ 193.5, 193.1, 153.0, 140.4, 130.3, 130.3, 129.3, 128.6.

GC-HRMS (EI, m/z): Mass calcd. for $C_{10}H_8O_2$; $[M]^+$, 160.0519; found 160.0520.

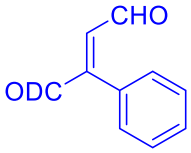

1a(D)

(*E*)-4-*oxo*-3-phenylbut-2-enal-4-*d* [1a(D)]

Yellow solid, 50% yield, 0.8 g, m.p. 87-88 °C.

^{1}H NMR (400 MHz, CDCl$_3$) δ 9.91 (d, J = 7.6 Hz, 1H), 7.57 – 7.43 (m, 3H), 7.37 – 7.30 (m, 2H), 6.72 (d, J = 7.6 Hz, 1H).

^{13}C NMR (101 MHz, CDCl$_3$) δ 193.1, 153.0, 140.4, 130.3, 130.3, 129.3, 128.6.

GC-HRMS (EI, m/z): Mass calcd. for $C_{10}H_7O_2D$; $[M]^+$, 161.0582; found 161.0582.

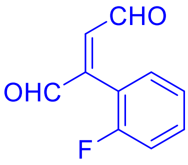

1b

2-(2-fluorophenyl) fumaraldehyde (1b)

Yellow solid, 61% yield, 1.1 g, m.p. 69-72 °C.

^{1}H NMR (400 MHz, CDCl$_3$) δ 9.93 (d, J = 0.9 Hz, 1H), 9.87 (dd, J = 7.6, 2.3 Hz, 1H), 7.57 – 7.44 (m, 1H), 7.36 – 7.15 (m, 3H), 6.83 (d, J = 7.6 Hz, 1H).

^{13}C NMR (101 MHz, CDCl$_3$) δ 192.2 (d, J = 1.9 Hz), 192.1, 159.8 (d, J = 249.3 Hz), 147.9, 141.1, 132.2 (d, J = 8.2 Hz), 132.0 (d, J = 2.7 Hz), 124.3 (d, J = 3.7 Hz), 117.5 (d, J = 15.8 Hz), 116.2 (d, J = 21.5 Hz).

^{19}F NMR (376 MHz, CDCl$_3$) δ -112.6.

GC-HRMS (EI, m/z): Mass calcd. for $C_{10}H_7O_2F$; $[M]^+$, 178.0425; found 178.0426.

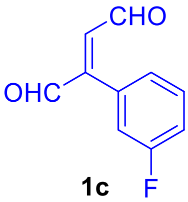

1c

2-(3-fluorophenyl) fumaraldehyde (1c)

Yellow solid, 56% yield, 1.0 g, m.p. 64-67 °C.

^{1}H NMR (400 MHz, CDCl$_3$) δ 9.94 (s, 1H), 9.92 (d, J = 7.6 Hz, 1H), 7.47 (td, J = 8.2, 6.1 Hz, 1H), 7.22 (m, 1H), 7.12 – 7.04 (m, 2H), 6.75 (d, J = 7.6 Hz, 1H).

^{13}C NMR (101 MHz, CDCl$_3$) δ 192.8, 192.5, 162.5 (d, J = 248.5 Hz), 151.6 (d, J = 2.1 Hz), 141.0, 131.2 (d, J = 8.0 Hz), 130.3 (d, J = 8.1 Hz), 126.2 (d, J = 3.0 Hz), 117.4 (d, J = 21.2 Hz), 117.3 (d, J = 23.2 Hz).

^{19}F NMR (376 MHz, CDCl$_3$) δ -111.6.

GC-HRMS (EI, m/z): Mass calcd. for $C_{10}H_7O_2F$; $[M]^+$, 178.0425; found 178.0428.

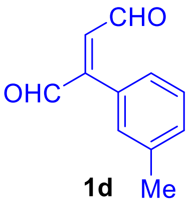

1d

2-(*m*-tolyl) fumaraldehyde (1d)

Yellow solid, 63% yield, 1.1 g, m.p. 59-62 °C.

^{1}H NMR (400 MHz, CDCl$_3$) δ 9.95 (s, 1H), 9.90 (d, J = 7.6 Hz, 1H), 7.43 – 7.29 (m, 2H), 7.12 (d, J = 7.5 Hz, 2H), 6.70 (d, J = 7.6 Hz, 1H), 2.41 (s, 3H).

^{13}C NMR (101 MHz, CDCl$_3$) δ 193.6, 193.2, 153.2, 140.1, 138.5, 131.0, 130.8, 129.3, 128.5, 127.5, 21.3.

GC-HRMS (EI, m/z): Mass calcd. for $C_{11}H_{10}O_2$; $[M]^+$, 174.0675; found 174.0675.

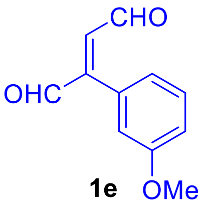

1e

2-(3-methoxyphenyl) fumaraldehyde (1e)

Yellow solid, 63% yield, 1.2 g, m.p. 50-52 °C.

^{1}H NMR (400 MHz, CDCl$_3$) δ 9.95 (s, 1H), 9.91 (d, J = 7.6 Hz, 1H), 7.39 (t, J = 7.9 Hz, 1H), 7.11 – 7.00 (m, 1H), 6.94 – 6.83 (m, 2H), 6.71 (d, J = 7.6 Hz, 1H), 3.84 (s, 3H).

^{13}C NMR (101 MHz, CDCl$_3$) δ 191.9, 191.6, 158.1, 151.4, 138.8, 129.2, 128.2, 121.3, 114.3, 114.3, 53.9.

GC-HRMS (EI, m/z): Mass calcd. for $C_{11}H_{10}O_3$; $[M]^+$, 190.0624; found 190.0624.

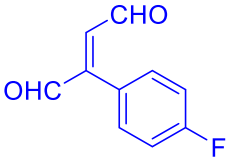

1f

2-(4-fluorophenyl) fumaraldehyde (1f)

Yellow solid, 35% yield, 0.4 g, m.p. 104-107 °C.

^{1}H NMR (400 MHz, CDCl$_3$) δ 9.95 (s, 1H), 9.92 (d, J = 7.6 Hz, 1H), 7.33 (dd, J = 8.6, 5.3 Hz, 2H), 7.19 (t, J = 8.6 Hz, 2H), 6.73 (d, J = 7.6 Hz, 1H).

^{13}C NMR (101 MHz, CDCl$_3$) δ 193.4, 192.6, 163.9 (d, J = 251.6 Hz), 151.8, 140.8, 132.4 (d, J = 8.6 Hz), 125.2 (d, J = 3.5 Hz), 115.9 (d, J = 22.1 Hz).

^{19}F NMR (376 MHz, CDCl$_3$) δ -109.4.

GC-HRMS (EI, m/z): Mass calcd. for $C_{10}H_7O_2F$; $[M]^+$, 178.0425; found 178.0426.

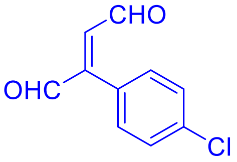

1g

2-(4-chlorophenyl) fumaraldehyde (1g)

Yellow solid, 47% yield, 0.9 g, m.p. 85-86 °C.

^{1}H NMR (400 MHz, CDCl$_3$) δ 9.94 (s, 1H), 9.91 (d, J = 7.6 Hz, 1H), 7.48 (d, J = 8.5 Hz, 2H), 7.28 (d, J = 8.3 Hz, 2H), 6.73 (d, J = 7.6 Hz, 1H).

^{13}C NMR (101 MHz, CDCl$_3$) δ 193.1, 192.5, 151.7, 140.9, 136.8, 131.6, 128.9, 127.6.

GC-HRMS (EI, m/z): Mass calcd. for $C_{10}H_7O_2Cl$; $[M]^+$, 194.0129; found 194.0131.

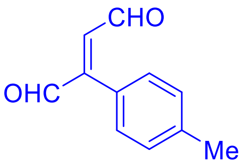

1h

2-(*p*-tolyl) fumaraldehyde (1h)

Yellow oil, 43% yield, 0.7 g,

^{1}H NMR (400 MHz, $CDCl_3$) δ 9.93 (s, 1H), 9.90 (d, J = 7.6 Hz, 1H), 7.31 – 7.26 (m, 2H), 7.25 – 7.21 (m, 2H), 6.68 (d, J = 7.6 Hz, 1H), 2.41 (s, 3H).

^{13}C NMR (101 MHz, $CDCl_3$) δ 193.8, 193.3, 152.9, 140.7, 140.0, 130.4, 129.3, 126.4, 21.4.

GC-HRMS (EI, m/z): Mass calcd. for $C_{11}H_{10}O_2$; $[M]^+$, 174.0675; found 174.0678.

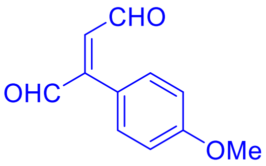

1i

2-(4-methoxyphenyl) fumaraldehyde (1i)

Yellow solid, 45% yield, 0.9 g, m.p. 81-82 °C.

^{1}H NMR (400 MHz, $CDCl_3$) δ 9.94 (s, 1H), 9.92 (d, J = 7.6 Hz, 1H), 7.30 (d, J = 8.8 Hz, 2H), 7.00 (d, J = 8.8 Hz, 2H), 6.65 (d, J = 7.6 Hz, 1H), 3.86 (s, 3H).

^{13}C NMR (101 MHz, $CDCl_3$) δ 194.1, 193.3, 161.4, 152.3, 139.71, 132.1, 121.5, 114.1, 55.4.

GC-HRMS (EI, m/z): Mass calcd. for $C_{11}H_{10}O_3$; $[M]^+$, 190.0624; found 190.0625.

2. Characterization of products

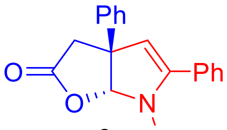

3a

(3a*R*,6a*R*)-3a,5-diphenyl-6-tosyl-3,3a,6,6a-tetrahydro-2*H*-furo[2,3-b]pyrrol-2-one (3a)

White solid, 66% yield, 28.5 mg, m.p. 175-176 °C.

$[\alpha]^{25}_D$ = -72.5 (c = 0.5 in $CHCl_3$).

^{1}H NMR (400 MHz, $CDCl_3$) δ 7.52 – 7.45 (m, 2H), 7.44 – 7.32 (m, 3H), 7.25 (d, J = 6.1 Hz, 3H), 7.15 (d, J = 8.3 Hz, 2H), 7.06 (dd, J = 7.5, 1.9 Hz, 2H), 6.91 (d, J = 8.2 Hz, 2H), 6.51 (s, 1H), 5.43 (s, 1H), 3.05 (m, 2H), 2.32 (s, 3H).

^{13}C NMR (101 MHz, $CDCl_3$) δ 173.2, 144.5, 144.1, 140.5, 134.5, 130.7, 129.8, 129.3, 129.1, 128.6, 128.0, 127.4, 127.2, 125.7, 118.2, 101.0, 56.2, 41.9, 21.5.

HRMS (ESI, m/z): Mass calcd. for $C_{25}H_{21}NO_4SNa^+$ $[M+Na]^+$, 454.1083; found 454.1091.

HPLC analysis (Chiralcel IB; 25 °C, IPA/Hexane = 40/60, 0.8 mL/min, 254 nm), Rt_1 (major) = 24.6 min, Rt_2 (minor) = 15.8 min; 98:2 er.

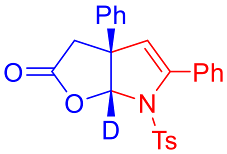

3a(D)

(3a*R*,6a*R*)-3a,5-diphenyl-6-tosyl-3,3a,6,6a-tetrahydro-2*H*-furo[2,3-b]pyrrol-2-one [3a(D)]

White solid, 66% yield, 28.5 mg, m.p. 175-176 °C.

$[\alpha]^{25}_D$ = -72.5 (c = 0.5 in $CHCl_3$).

^{1}H NMR (400 MHz, $CDCl_3$) δ 7.52 – 7.45 (m, 2H), 7.44 – 7.32 (m, 3H), 7.25 (d, J = 6.1 Hz, 3H), 7.15 (d, J = 8.3 Hz, 2H), 7.06 (dd, J = 7.5, 1.9 Hz, 2H), 6.91 (d, J = 8.2 Hz, 2H), 5.43 (s, 1H), 3.05 (m, 2H), 2.32 (s, 3H).

^{13}C NMR (101 MHz, CDCl$_3$) δ 173.2, 144.5, 144.1, 140.5, 134.5, 130.7, 129.8, 129.3, 129.1, 128.6, 128.0, 127.4, 127.2, 125.7, 118.2, 56.2, 41.9, 21.5.

HRMS (ESI, m/z): Mass calcd. for $C_{25}H_{20}DNO_4SNa^+$ [M+Na]$^+$, 455.1146 found 455.1149.

HPLC analysis (Chiralcel IB; 25 °C, IPA/Hexane = 40/60, 0.8 mL/min, 254 nm), Rt_1 (major) = 24.8 min, Rt_2 (minor) = 15.1 min; 98:2 er.

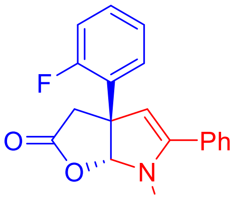

3b

(3a*R*,6a*R*)-3a-(2-fluorophenyl)-5-phenyl-6-tosyl-3,3a,6,6a-tetrahydro-2*H*-furo[2,3-b]pyrrol-2-one (3b)

White solid, 31% yield, 13.8 mg, m.p. 221-222 °C.

[α]$^{25}_{D}$ = -92.6(c = 0.5 in $CHCl_3$).

^{1}H NMR (400 MHz, CDCl$_3$) δ 7.53 (dd, J = 4.3, 3.8 Hz, 2H), 7.40 (dt, J = 7.1, 6.6 Hz, 3H), 7.30 – 7.23 (m, 2H), 7.17 (d, J = 8.1 Hz, 2H), 7.09 (dd, J = 11.4, 8.3 Hz, 1H), 6.87 (dd, J = 11.1, 3.5 Hz, 2H), 6.76 (t, J = 7.7 Hz, 1H), 6.57 (d, J = 0.6 Hz, 1H), 5.47 (s, 1H), 3.11 (m, 2H), 2.28 (s, 3H).

^{13}C NMR (101 MHz, CDCl$_3$) δ 173.0, 159.8 (d, J = 246.3 Hz), 145.2, 144.2, 134.1, 130.8, 130.0, 129.5 (d, J = 1.2 Hz), 129.3, 128.4, 128.0, 127.8 (d, J = 3.7 Hz), 127.0, 126.4, 124.4, 117.1, 116.4 (d, J = 21.6 Hz), 99.0 (d, J = 5.0 Hz), 53.3, 40.3, 21.5.

^{19}F NMR (376 MHz, CDCl$_3$) δ -113.0.

HRMS (ESI, m/z): Mass calcd. for $C_{25}H_{20}FNO_4SNa^+$ [M+Na]$^+$, 472.0989; found 472.0993.

HPLC analysis (Chiralcel IB; 25 °C, IPA/Hexane = 10/90, 1.0 mL/min, 254 nm), Rt_1 (major) = 40.2 min, Rt_2 (minor) = 43.7 min; 98:2 er.

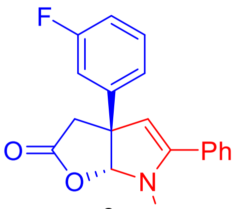

3c

(3a*R*,6a*R*)-3a-(3-fluorophenyl)-5-phenyl-6-tosyl-3,3a,6,6a-tetrahydro-2*H*-furo[2,3-b]pyrrol-2-one (3c)

White solid, 40% yield, 17.8 mg, m.p. 214-215°C.

[α]$^{25}_{D}$ = -38.6 (c = 0.3 in $CHCl_3$).

^{1}H NMR (400 MHz, CDCl$_3$) δ 7.56 – 7.50 (m, 2H), 7.44 – 7.36 (m, 3H), 7.31 – 7.24 (m, 1H), 7.16 (d, J = 8.2 Hz, 2H), 6.94 (d, J = 7.9 Hz, 4H), 6.56 (dd, J = 9.9, 1.9 Hz, 1H), 6.42 (s, 1H), 5.40 (s, 1H), 3.03 (m, 2H), 2.33 (s, 3H).

^{13}C NMR (101 MHz, CDCl$_3$) δ 172.8, 162.7 (d, J = 248.1 Hz), 145.3, 144.5, 143.2 (d, J = 6.6 Hz), 134.2, 130.9 (d, J = 8.4 Hz), 130.5, 130.0, 129.4, 128.5, 128.0, 127.2, 121.3 (d, J = 2.9 Hz), 117.4, 114.4 (d, J = 21.1 Hz), 113.4 (d, J = 23.1 Hz), 100.9, 55.9, 41.8, 21.4.

^{19}F NMR (376 MHz, CDCl$_3$) δ -110.7.

HRMS (ESI, m/z): Mass calcd. for $C_{25}H_{20}FNO_4SNa^+$ [M+Na]$^+$, 472.0989; found 472.0996.

HPLC analysis (Chiralcel IB; 25 °C, IPA/Hexane = 40/60, 0.8 mL/min, 254 nm), Rt_1 (major) = 17.3 min, Rt_2 (minor) = 14.5 min; 99:1 er.

(3a*R*,6a*R*)-5-phenyl-3a-(*m*-tolyl)-6-tosyl-3,3a,6,6a-tetrahydro-2*H*-furo[2,3-b]pyrrol-2-one (3d)

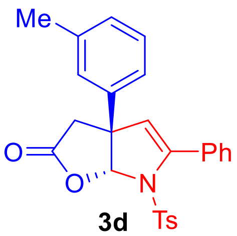

3d

White solid, 58% yield, 25.8 mg, m.p. 199-200°C.

[α]$^{25}_{D}$ = -60.1 (*c* = 1.0 in $CHCl_3$).

^{1}H NMR (400 MHz, $CDCl_3$) *δ* 7.53 – 7.47 (m, 2H), 7.46 – 7.33 (m, 3H), 7.12 (t, *J* = 7.8 Hz, 3H), 7.06 (d, *J* = 7.4 Hz, 1H), 6.89 (d, *J* = 8.0 Hz, 2H), 6.84 (d, *J* = 7.9 Hz, 2H), 6.50 (s, 1H), 5.45 (s, 1H), 3.02 (m, 2H), 2.31 (s,6H).

^{13}C NMR (101 MHz, $CDCl_3$) *δ* 173.3, 144.3, 144.0, 140.6, 138.7, 134.4, 130.8, 129.8, 129.2, 129.0, 128.6, 128.2, 128.0, 127.3, 126.4, 122.8, 118.4, 101.1, 56.1, 42.1, 21.5, 21.5.

HRMS (ESI, m/z): Mass calcd. for $C_{26}H_{23}NO_4SNa^+$ [M+Na]$^+$, 468.1240; found 468.1245.

HPLC analysis (Chiralcel IB; 25 °C, IPA/Hexane = 40/60, 0.8 mL/min, 254 nm), Rt$_1$ (major) = 24.0 min, Rt$_2$ (minor) = 13.4 min; 98:2 er.

(3a*R*,6a*R*)-3a-(3-methoxyphenyl)-5-phenyl-6-tosyl-3,3a,6,6a-tetrahydro-2*H*-furo[2,3-b]pyrrol-2-one (3e)

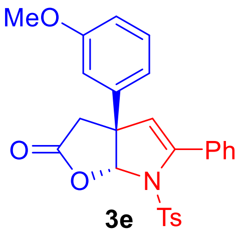

3e

White solid, 41% yield, 18.3 mg, m.p. 201-202°C.

[α]$^{25}_{D}$ = -14.0 (*c* = 0.4 in $CHCl_3$).

^{1}H NMR (400 MHz, $CDCl_3$) *δ* 7.50 – 7.32 (m, 5H), 7.23 – 7.13 (m, 3H), 6.93 (d, *J* = 8.0 Hz, 2H), 6.79 (dd, *J* = 8.2, 1.9 Hz, 1H), 6.69 (dd, *J* = 7.7, 1.0 Hz, 1H), 6.51 (t, *J* = 1.9 Hz, 2H), 5.42 (s, 1H), 3.80 (s, 3H), 3.04 (m, 2H), 2.32 (s, 3H).

^{13}C NMR (101 MHz, $CDCl_3$) *δ* 173.1, 159.9, 144.6, 144.2, 142.2, 134.6, 130.7, 130.2, 129.8, 129.2, 128.5, 128.0, 127.3, 118.1, 117.8, 112.6, 111.9, 100.9, 56.2, 55.2, 41.8, 21.5.

HRMS (ESI, m/z): Mass calcd. for $C_{26}H_{23}NO_5SNa^+$ [M+Na]$^+$, 484.1189; found 484.1195.

HPLC analysis (Chiralcel IB; 25 °C, IPA/Hexane = 40/60, 0.8 mL/min, 254 nm), Rt$_1$ (major) = 24.5 min, Rt$_2$ (minor) = 16.4 min; 99:1 er.

(3a*R*,6a*R*)-3a-(4-fluorophenyl)-5-phenyl-6-tosyl-3,3a,6,6a-tetrahydro-2*H*-furo[2,3-b]pyrrol-2-one (3f)

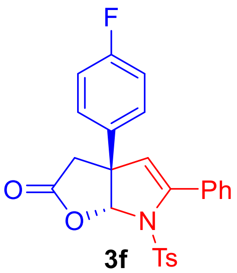

3f

White solid, 54% yield, 24.1 mg, m.p. 217-218°C.

[α]$^{25}_{D}$ = -71.1 (*c* = 1.0 in $CHCl_3$).

^{1}H NMR (400 MHz, $CDCl_3$) *δ* 7.54 – 7.48 (m, 2H), 7.46 – 7.33 (m, 3H), 7.20 – 7.10 (m, 2H), 7.07 – 7.00 (m, 2H), 6.96 – 6.85 (m, 4H), 6.43 (d, *J* = 0.7 Hz, 1H), 5.43 (d, *J* = 0.5 Hz, 1H), 3.56 (m, 2H), 2.34 (s, 3H).

^{13}C NMR (101 MHz, $CDCl_3$) *δ* 173.0, 161.9 (d, *J* = 247.9 Hz), 144.8, 144.5, 136.3 (d, *J* = 3.4 Hz), 134.4, 130.6, 130.0, 129.4, 128.5, 128.0, 127.6 (d, *J* = 8.4 Hz), 127.2, 118.0, 115.9 (d, *J* = 21.7 Hz), 100.9, 55.7, 41.8, 21.5.

^{19}F NMR (376 MHz, $CDCl_3$) *δ* -114.0.

HRMS (ESI, m/z): Mass calcd. for $C_{25}H_{20}FNO_4SNa^+$ [M+Na]$^+$, 472.0989; found 472.0989.

HPLC analysis (Chiralcel IB; 25 °C, IPA/Hexane = 40/60, 0.8 mL/min, 254 nm), Rt$_1$ (major) = 19.0

min, Rt_2 (minor) = 14.3 min; 96:4 er.

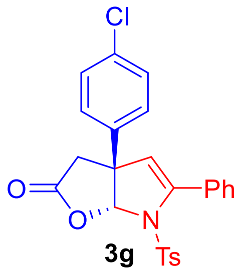

3g

(3a*R*,6a*R*)-3a-(4-chlorophenyl)-5-phenyl-6-tosyl-3,3a,6,6a-tetrahydro-2*H*-furo[2,3-b]pyrrol-2-one (3g)

White solid, 61% yield, 27.2mg, m.p. 175-176°C.

$[\alpha]^{25}_{D}$ = -67.4 (c = 1.0 in $CHCl_3$).

^{1}H NMR (400 MHz, $CDCl_3$) δ 7.61 – 7.50 (m, 2H), 7.41 (ddd, J = 19.0, 7.7, 6.2 Hz, 3H), 7.17 (dd, J = 14.3, 8.5 Hz, 4H), 6.95 (dd, J = 11.9, 5.2 Hz, 4H), 6.41 (d, J = 0.6 Hz, 1H), 5.44 (d, J = 0.4 Hz, 1H), 3.01 (m, 2H), 2.37 (s, 3H).

^{13}C NMR (101 MHz, $CDCl_3$) δ 172.8, 145.1, 144.7, 139.1, 134.2, 133.5, 130.6, 130.0, 129.3, 129.1, 128.5, 128.1, 127.2, 127.1, 117.7, 100.9, 55.8, 41.8, 21.5.

HRMS (ESI, m/z): Mass calcd. for $C_{25}H_{20}ClNO_4SNa^+$ $[M+Na]^+$, 488.0694; found 488.0693.

HPLC analysis (Chiralcel IB; 25 °C, IPA/Hexane = 40/60, 0.8 mL/min, 254 nm), Rt_1 (major) = 31.5 min, Rt_2 (minor) = 16.8 min; 99:1 er.

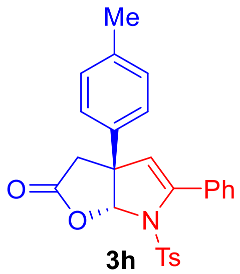

3h

(3a*R*,6a*R*)-5-phenyl-3a-(*p*-tolyl)-6-tosyl-3,3a,6,6a-tetrahydro-2*H*-furo[2,3-b]pyrrol-2-one (3h)

White solid, 30% yield, 13.4 mg, m.p. 195-196°C.

$[\alpha]^{25}_{D}$ = -31.2 (c = 0.4 in $CHCl_3$).

^{1}H NMR (400 MHz, $CDCl_3$) δ 7.41 (ddd, J = 23.7, 11.4, 6.8 Hz, 5H), 7.16 (d, J = 8.3 Hz, 2H), 7.05 (d, J = 8.1 Hz, 2H), 6.92 (dd, J = 8.0, 5.4 Hz, 4H), 6.48 (s, 1H), 5.42 (s, 1H), 3.03 (m, 2H), 2.35 (d, J = 13.3 Hz, 6H).

^{13}C NMR (101 MHz, $CDCl_3$) δ 173.3, 144.3, 144.1, 137.5, 137.3, 134.7, 130.8, 129.7, 129.7, 129.2, 128.6, 127.9, 127.2, 125.6, 118.4, 101.0, 55.9, 41.8, 21.5, 21.0.

HRMS (ESI, m/z): Mass calcd. for $C_{26}H_{23}NO_4SNa^+$ $[M+Na]^+$, 468.1240; found 468.1247.

HPLC analysis (Chiralcel IB; 25 °C, IPA/Hexane = 40/60, 0.8 mL/min, 254 nm), Rt_1 (major) = 36.5 min, Rt_2 (minor) = 16.1 min; 98:2 er.

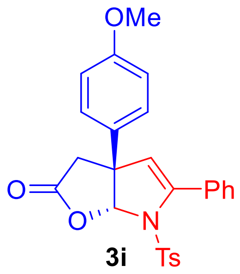

3i

(3a*R*,6a*R*)-3a-(4-methoxyphenyl)-5-phenyl-6-tosyl-3,3a,6,6a-tetrahydro-2*H*-furo[2,3-b]pyrrol-2-one (3i)

White solid, 56% yield, 25.0 mg, m.p. 202-203°C.

$[\alpha]^{25}_{D}$ = -60.7 (c = 0.5 in $CHCl_3$).

^{1}H NMR (400 MHz, $CDCl_3$) δ 7.45 (d, J = 7.0 Hz, 2H), 7.42 – 7.38 (m, 1H), 7.34 (d, J = 7.0 Hz, 2H), 7.18 (d, J = 8.2 Hz, 2H), 6.96 (dd, J = 10.5, 8.6 Hz, 4H), 6.77 (d, J = 8.7 Hz, 2H), 6.46 (s, 1H), 5.41 (s, 1H), 3.83 (s, 3H), 3.03 (m, 2H), 2.34 (s, 3H).

^{13}C NMR (101 MHz, $CDCl_3$) δ 173.3, 159.0, 144.2, 144.1, 134.9, 132.3, 130.7, 129.7, 129.3, 128.6, 127.9, 127.3, 126.9, 118.4, 114.4, 101.0, 55.6, 55.3, 41.6, 21.5.

HRMS (ESI, m/z): Mass calcd. for $C_{26}H_{23}NO_5SNa^+$ $[M+Na]^+$, 484.1189; found 484.1199.

HPLC analysis (Chiralcel IB; 25 °C, IPA/Hexane = 40/60, 0.8 mL/min, 254 nm), Rt_1 (major) = 47.4 min, Rt_2 (minor) = 21.1 min; 97:3 er.

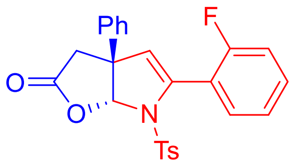

4a

(3a*R*,6a*R*)-5-(2-fluorophenyl)-3a-phenyl-6-tosyl-3,3a,6,6a-tetrahydro-2*H*-furo[2,3-b]pyrrol-2-one (4a)

White solid, 68% yield, 30.3 mg, m.p. 164-165 °C.

[α]$^{25}_{D}$ = -46.4 (c = 0.5 in $CHCl_3$).

^{1}H NMR (400 MHz, $CDCl_3$) δ 7.52 (td, J = 7.5, 1.7 Hz, 1H), 7.39 (tdd, J = 7.2, 5.2, 1.7 Hz, 1H), 7.29 – 7.21 (m, 3H), 7.16 (ddd, J = 7.4, 5.7, 1.5 Hz, 3H), 7.13 – 7.04 (m, 3H), 6.91 (d, J = 8.0 Hz, 2H), 6.43 (d, J = 0.5 Hz, 1H), 5.60 (s, 1H), 3.06 (m, 2H), 2.32 (s, 3H).

^{13}C NMR (101 MHz, $CDCl_3$) δ 173.1, 160.1 (d, J = 250.6 Hz), 144.2, 140.2, 137.0, 134.3, 131.8 (d, J = 1.8 Hz), 131.3 (d, J = 8.4 Hz), 129.4, 129.1, 127.4, 127.2, 125.8, 123.6 (d, J = 3.6 Hz), 120.7 (d, J = 3.8 Hz), 118.5 (d, J = 12.9 Hz), 115.6 (d, J = 21.7 Hz), 100.2, 56.7, 41.5, 21.5.

^{19}F NMR (376 MHz, $CDCl_3$) δ -113.8.

HRMS (ESI, m/z): Mass calcd. for $C_{25}H_{20}FNO_4SNa^+$ $[M+Na]^+$, 472.0989; found 472.0998.

HPLC analysis (Chiralcel IB; 25 °C, IPA/Hexane = 40/60, 0.8 mL/min, 254 nm), Rt_1 (major) = 20.2 min, Rt_2 (minor) = 14.7 min; 96:4 er.

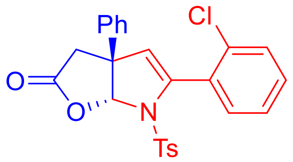

4b

(3a*R*,6a*R*)-5-(2-chlorophenyl)-3a-phenyl-6-tosyl-3,3a,6,6a-tetrahydro-2*H*-furo[2,3-b]pyrrol-2-one (4b)

White solid, 71% yield, 31.6 mg, m.p. 185-187 °C.

[α]$^{25}_{D}$ = -34.2 (c = 0.2 in $CHCl_3$).

^{1}H NMR (400 MHz, $CDCl_3$) δ 7.48 (d, J = 7.6 Hz, 1H), 7.30 (ddd, J = 11.6, 7.8, 3.2 Hz, 6H), 7.21 (dd, J = 10.6, 6.0 Hz, 4H), 6.96 (d, J = 8.1 Hz, 2H), 6.51 (s, 1H), 5.56 (s, 1H), 3.10 (m, 2H), 2.33 (s, 3H).

^{13}C NMR (101 MHz, $CDCl_3$) δ 173.2, 144.2, 140.1, 139.4, 134.9, 133.6, 132.4, 130.6, 129.7, 129.3, 129.2, 128.9, 127.7, 127.5, 126.1, 125.9, 120.5, 99.7, 56.8, 40.9, 21.5.

HRMS (ESI, m/z): Mass calcd. for $C_{25}H_{20}ClNO_4SNa^+$ $[M+Na]^+$, 488.0694; found 488.0701.

HPLC analysis (Chiralcel IB; 25 °C, IPA/Hexane = 40/60, 0.8 mL/min, 254 nm), Rt_1 (major) = 17.1 min, Rt_2 (minor) = 14.3 min; 95:5 er.

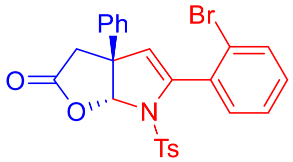

4c

(3a*R*,6a*R*)-5-(2-bromophenyl)-3a-phenyl-6-tosyl-3,3a,6,6a-tetrahydro-2*H*-furo[2,3-b]pyrrol-2-one (4c)

White solid, 62% yield, 27.6 mg, m.p. 187-189 °C.

[α]$^{25}_{D}$ = -26.3 (c = 1.0 in $CHCl_3$).

^{1}H NMR (400 MHz, $CDCl_3$) δ 7.47 (dd, J = 7.7, 1.7 Hz, 2H), 7.32 (t, J = 6.7 Hz, 5H), 7.23 (dd, J = 12.0, 5.7 Hz, 4H), 6.98 (d, J = 8.1 Hz, 2H), 6.55 (s, 1H), 5.53 (s, 1H), 3.12 (m, 2H), 2.34 (s, 3H).

^{13}C NMR (101 MHz, CDCl$_3$) δ 173.3, 144.2, 140.9, 140.1, 135.0, 132.9, 132.6, 130.8, 129.4, 129.2, 127.7, 127.6, 126.7, 125.9, 120.3, 99.7, 56.7, 40.8, 21.5.

HRMS (ESI, m/z): Mass calcd. for $C_{25}H_{20}BrNO_4SNa^+$ [M+Na]$^+$, 532.0189; found 532.0190.

HPLC analysis (Chiralcel IB; 25 °C, IPA/Hexane = 40/60, 0.8 mL/min, 254 nm), Rt$_1$ (major) = 17.2 min, Rt$_2$ (minor) = 14.8 min; 95:5 er.

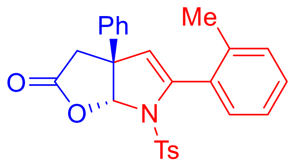

4d

(3a*R*,6a*R*)-3a-phenyl-5-(*o*-tolyl)-6-tosyl-3,3a,6,6a-tetrahydro-2*H*-furo[2,3-b]pyrrol-2-one (4d)

White solid, 67% yield, 29.9 mg, m.p. 160-162 °C.

[α]$^{25}_{D}$ = -59.4 (c = 0.5 in CHCl$_3$).

^{1}H NMR (400 MHz, CDCl$_3$) δ 7.36 – 7.22 (m, 7H), 7.13 (dd, J = 23.6, 7.9 Hz, 4H), 6.97 (d, J = 8.1 Hz, 2H), 6.60 (s, 1H), 5.29 (s, 1H), 3.13 (m, 2H), 2.34 (s, 3H), 2.15 (s, 3H).

^{13}C NMR (101 MHz, CDCl$_3$) δ 173.3, 144.0, 140.1, 138.2, 135.5, 130.4, 130.0, 129.7, 129.6, 129.3, 129.2, 129.2, 127.8, 127.6, 125.8, 125.0, 118.1, 99.7, 56.1, 40.8, 21.5, 19.7.

HRMS (ESI, m/z): Mass calcd. for $C_{26}H_{23}NO_4SNa^+$ [M+Na]$^+$, 468.1240; found 468.1237.

HPLC analysis (Chiralcel IB; 25 °C, IPA/Hexane = 40/60, 0.8 mL/min, 254 nm), Rt$_1$ (major) = 17.4 min, Rt$_2$ (minor) = 13.5 min; 96:4 er.

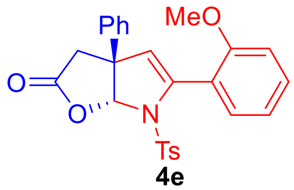

4e

(3a*R*,6a*R*)-5-(2-methoxyphenyl)-3a-phenyl-6-tosyl-3,3a,6,6a-tetrahydro-2*H*-furo[2,3-b]pyrrol-2-one (4e)

White solid, 61% yield, 27.2 mg, m.p. 182-184 °C.

[α]$^{25}_{D}$ = -31.6 (c = 0.5 in CHCl$_3$).

^{1}H NMR (400 MHz, CDCl$_3$) δ 7.39 – 7.28 (m, 5H), 7.22 (dd, J = 13.5, 5.3 Hz, 4H), 7.00 – 6.89 (m, 3H), 6.77 (d, J = 8.3 Hz, 1H), 6.51 (s, 1H), 5.40 (s, 1H), 3.62 (s, 3H), 3.10 (m, 2H), 2.34 (s, 3H).

^{13}C NMR (101 MHz, CDCl$_3$) δ 173.5, 157.5, 143.6, 140.4, 139.4, 135.6, 131.7, 130.9, 129.1, 129.0, 127.6, 127.6, 125.9, 119.7, 119.0, 118.6, 110.4, 99.7, 56.2, 55.2, 40.8, 21.5.

HRMS (ESI, m/z): Mass calcd. for $C_{26}H_{23}NO_5SNa^+$ [M+Na]$^+$, 484.1189; found 484.1194.

HPLC analysis (Chiralcel IB; 25 °C, IPA/Hexane = 40/60, 0.8 mL/min, 254 nm), Rt$_1$ (major) = 54.9 min, Rt$_2$ (minor) = 51.4 min; 98:2 er.

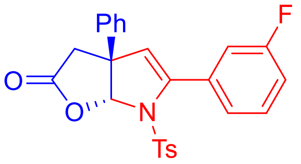

4f

(3a*R*,6a*R*)-5-(3-fluorophenyl)-3a-phenyl-6-tosyl-3,3a,6,6a-tetrahydro-2*H*-furo[2,3-b]pyrrol-2-one (4f)

White solid, 58% yield, 25.8 mg, m.p. 222-223 °C.

[α]$^{25}_{D}$ = -50.9 (*c* = 0.3 in $CHCl_3$).

^{1}H NMR (400 MHz, $CDCl_3$) δ 7.36 – 7.23 (m, 5H), 7.19 (d, *J* = 8.3 Hz, 2H), 7.15 – 7.08 (m, 2H), 7.07 – 7.02 (m, 2H), 6.94 (d, *J* = 8.0 Hz, 2H), 6.51 (d, *J* = 0.5 Hz, 1H), 5.49 (s, 1H), 3.06 (m, 2H), 2.33 (s, 3H).

^{13}C NMR (101 MHz, $CDCl_3$) δ 173.0, 162.2 (d, *J* = 246.7 Hz), 144.4, 143.3 (d, *J* = 2.5 Hz), 140.2, 134.5, 132.7 (d, *J* = 8.4 Hz), 129.6 (d, *J* = 8.4 Hz), 129.4, 129.2, 127.5, 127.2, 125.6, 124.3 (d, *J* = 2.9 Hz), 119.1, 116.7 (d, *J* = 21.0 Hz), 115.5 (d, *J* = 23.2 Hz), 100.8, 56.3, 41.6, 21.5.

^{19}F NMR (376 MHz, $CDCl_3$) δ -112.8.

HRMS (ESI, m/z): Mass calcd. for $C_{25}H_{20}FNO_4SNa^+$ $[M+Na]^+$, 472.0989; found 472.0991.

HPLC analysis (Chiralcel IB; 25 °C, IPA/Hexane = 40/60, 0.8 mL/min, 254 nm), Rt_1 (major) = 25.2min, Rt_2 (minor) = 15.1 min; 95:5 er.

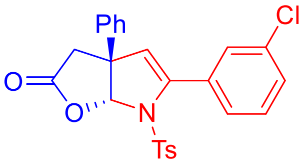

4g

(3a*R*,6a*R*)-5-(3-chlorophenyl)-3a-phenyl-6-tosyl-3,3a,6,6a-tetrahydro-2*H*-furo[2,3-b]pyrrol-2-one (4g)

White solid, 52% yield, 24.3 mg, m.p. 198-199 °C.;

[α]$^{25}_{D}$ = -42.9 (*c* = 1 in $CHCl_3$).

^{1}H NMR (400 MHz, $CDCl_3$) δ 7.43 – 7.35 (m, 2H), 7.29 (ddd, *J* = 7.1, 4.0, 1.8 Hz, 5H), 7.19 (d, *J* = 8.3 Hz, 2H), 7.12 – 7.02 (m, 2H), 6.95 (d, *J* = 8.1 Hz, 2H), 6.53 (s, 1H), 5.49 (s, 1H), 3.06 (m, 2H), 2.33 (s, 3H).

^{13}C NMR (101 MHz, $CDCl_3$) δ 173.0, 144.5, 143.0, 140.2, 134.5, 133.9, 132.4, 129.8, 129.5, 129.3, 129.2, 128.3, 127.6, 127.2, 126.9, 125.7, 119.2, 100.6, 56.4, 41.5, 21.5.

HRMS (ESI, m/z): Mass calcd. for $C_{26}H_{20}ClNO_4SNa^+$ $[M+Na]^+$, 488.0694; found 488.0690.

HPLC analysis (Chiralcel IB; 25 °C, IPA/Hexane = 40/60, 0.8 mL/min, 254 nm), Rt_1 (major) = 25.6 min, Rt_2 (minor) = 15.5 min; 97:3 er.

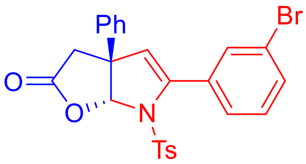

4h

(3a*R*,6a*R*)-5-(3-bromophenyl)-3a-phenyl-6-tosyl-3,3a,6,6a-tetrahydro-2*H*-furo[2,3-b]pyrrol-2-one (4h)

White solid, 45% yield, 23.2 mg, m.p. 182-183 °C.

[α]$^{25}_{D}$ = -25.5 (*c* = 0.2 in $CHCl_3$).

^{1}H NMR (400 MHz, $CDCl_3$) δ 7.52 (d, *J* = 8.1 Hz, 1H), 7.44 (d, *J* = 7.4 Hz, 2H), 7.31 – 7.18 (m, 6H), 7.09 (dd, *J* = 6.6, 2.8 Hz, 2H), 6.98 (d, *J* = 8.1 Hz, 2H), 6.55 (s, 1H), 5.47 (s, 1H), 3.07 (m, 2H), 2.34 (s, 3H).

^{13}C NMR (101 MHz, $CDCl_3$) δ 173.0, 144.5, 142.8, 140.1, 134.7, 132.7, 132.6, 131.1, 129.6, 129.5, 129.2, 127.6, 127.4, 127.3, 125.7, 121.9, 119.2, 100.5, 56.4, 41.4, 21.6.

HRMS (ESI, m/z): Mass calcd. for $C_{25}H_{20}BrNO_4SNa^+$ $[M+H]^+$, 532.0189; found 532.0186.

HPLC analysis (Chiralcel IB; 25 °C, IPA/Hexane = 40/60, 0.8 mL/min, 254 nm), Rt_1 (major) = 24.0 min, Rt_2 (minor) = 15.4 min; 97:3 er.

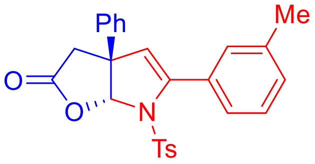

4i

(3a*R*,6a*R*)-3a-phenyl-5-(*m*-tolyl)-6-tosyl-3,3a,6,6a-tetrahydro-2*H*-furo[2,3-b]pyrrol-2-one (4i)

White solid, 64% yield, 28.5 mg, m.p. 199-200 °C.;

$[\alpha]^{25}_{D}$ = -40.9 (*c* = 1 in $CHCl_3$).

^{1}H NMR (400 MHz, $CDCl_3$) δ 7.29 – 7.23 (m, 5H), 7.23 – 7.17 (m, 4H), 7.13 – 7.07 (m, 2H), 6.94 (d, J = 8.4 Hz, 2H), 6.54 (s, 1H), 5.41 (s, 1H), 3.06 (m, 2H), 2.33 (d, J = 3.6 Hz, 6H).

^{13}C NMR (101 MHz, $CDCl_3$) δ 172.3, 143.6, 143.1, 139.5, 136.6, 134.0, 129.6, 128.4, 128.2, 128.2, 127.0, 126.5, 126.4, 124.8, 124.7, 117.0, 99.8, 55.3, 40.7, 20.6, 20.3.

HRMS (ESI, m/z): Mass calcd. for $C_{26}H_{23}NO_4SNa^+$ $[M+Na]^+$, 468.1240; found 468.1229.

HPLC analysis (Chiralcel IB; 25 °C, IPA/Hexane = 40/60, 0.8 mL/min, 254 nm), Rt_1 (major) = 19.7 min, Rt_2 (minor) = 13.3 min; 99:1 er.

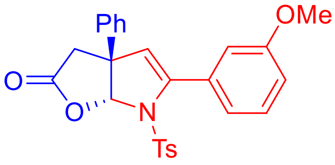

4j

(3a*R*,6a*R*)-5-(3-methoxyphenyl)-3a-phenyl-6-tosyl-3,3a,6,6a-tetrahydro-2*H*-furo[2,3-b]pyrrol-2-one (4j)

White solid, 69% yield, 30.7 mg, m.p. 238-240 °C.

$[\alpha]^{25}_{D}$ = -24.3 (*c* = 0.1 in $CHCl_3$).

^{1}H NMR (400 MHz, $CDCl_3$) δ 7.30 – 7.22 (m, 4H), 7.19 (d, J = 8.3 Hz, 2H), 7.09 – 7.02 (m, 3H), 6.94 (dt, J = 10.9, 5.7 Hz, 4H), 6.52 (d, J = 0.6 Hz, 1H), 5.45 (d, J = 0.5 Hz, 1H), 3.79 (s, 3H), 3.06 (m, 2H), 2.32 (s, 3H).

^{13}C NMR (101 MHz, $CDCl_3$) δ 173.2, 159.0, 144.3, 144.1, 140.4, 134.7, 131.9, 129.3, 129.1, 129.0, 127.5, 127.3, 125.7, 120.9, 118.3, 115.9, 113.8, 100.9, 56.2, 55.3, 41.7, 21.5.

HRMS (ESI, m/z): Mass calcd. for $C_{26}H_{23}NO_5SNa^+$ $[M+Na]^+$, 484.1189; found 484.1189.

UPLC analysis (Chiralcel IB; 25 °C, IPA/Hexane = 40/60, 0.8 mL/min, 254 nm), Rt_1 (major) = 24.7 min, Rt_2 (minor) =15.5 min; 97:3 er.

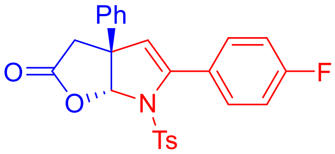

4k

(3a*R*,6a*R*)-5-(4-fluorophenyl)-3a-phenyl-6-tosyl-3,3a,6,6a-tetrahydro-2*H*-furo[2,3-b]pyrrol-2-one (4k)

White solid, 62% yield, 27.6 mg, m.p. 207-208 °C.

$[\alpha]^{25}_{D}$ = -49.4 (*c* = 0.3 in $CHCl_3$).

^{1}H NMR (400 MHz, $CDCl_3$) δ 7.53 – 7.39 (m, 2H), 7.30 – 7.22 (m, 3H), 7.14 (d, J = 8.3 Hz, 2H), 7.05 (t, J = 8.7 Hz, 4H), 6.92 (d, J = 8.2 Hz, 2H), 6.50 (s, 1H), 5.41 (s, 1H), 3.05 (m, 2H), 2.32 (s, 3H).

^{13}C NMR (101 MHz, CDCl$_3$) δ 173.1, 163.5 (d, J = 250.1 Hz), 144.2, 143.4, 140.4, 134.5, 130.5 (d, J = 8.3 Hz), 129.4, 129.1, 127.5, 127.2, 126.7 (d, J = 3.7 Hz), 125.7, 118.0, 115.1 (d, J = 22.0 Hz), 100.8, 56.2, 41.8, 21.5.

^{19}F NMR (376 MHz, CDCl$_3$) δ -110.3.

HRMS (ESI, m/z): Mass calcd. for $C_{25}H_{20}FNO_4SNa^+$ [M+Na]$^+$, 472.0989; found 472.0991.

HPLC analysis (Chiralcel IB; 25 °C, IPA/Hexane = 40/60, 0.8 mL/min, 254 nm), Rt$_1$ (major) = 22.2min, Rt$_2$ (minor) = 14.3 min; 99:1 er.

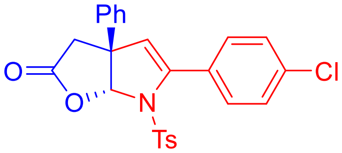

4l

(3a*R*,6a*R*)-5-(4-chlorophenyl)-3a-phenyl-6-tosyl-3,3a,6,6a-tetra hydro-2*H*-furo[2,3-b]pyrrol-2-one (4l)

White solid, 60% yield, 26.7 mg, m.p. 194-196 °C.

[α]$^{25}_{D}$ = -118.3 (c = 0.1 in CHCl$_3$).

^{1}H NMR (400 MHz, CDCl$_3$) δ 7.42 (d, J = 8.5 Hz, 2H), 7.34 (d, J = 8.5 Hz, 2H), 7.25 (dd, J = 5.3, 3.3 Hz, 3H), 7.15 (d, J = 8.3 Hz, 2H), 7.03 (dd, J = 7.9, 1.6 Hz, 2H), 6.92 (d, J = 8.1 Hz, 2H), 6.49 (s, 1H), 5.45 (s, 1H), 3.05 (m, 2H), 2.32 (s, 3H).

^{13}C NMR (101 MHz, CDCl$_3$) δ 173.0, 144.3, 143.4, 140.3, 135.8, 134.4, 129.8, 129.4, 129.1, 128.3, 127.5, 127.2, 125.7, 118.6, 100.8, 56.3, 41.7, 21.5.

HRMS (ESI, m/z): Mass calcd. for $C_{25}H_{20}ClNO_4SNa^+$ [M+Na]$^+$, 488.0694; found 488.0693.

HPLC analysis (Chiralcel IB; 25 °C, IPA/Hexane = 40/60, 0.8 mL/min, 254 nm), Rt$_1$ (major) = 22.9min, Rt$_2$ (minor) = 14.5 min; 97:3 er.

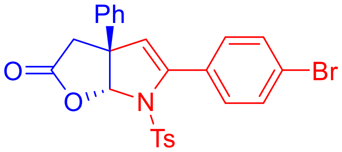

4m

(3a*R*,6a*R*)-5-(4-bromophenyl)-3a-phenyl-6-tosyl-3,3a,6,6a-tetra hydro-2*H*-furo[2,3-b]pyrrol-2-one (4m)

White solid, 67% yield, 29.9 mg, m.p. 236-237 °C.

[α]$^{25}_{D}$ = -76.8 (c = 0.1 in CHCl$_3$).

^{1}H NMR (400 MHz, CDCl$_3$) δ 7.50 (d, J = 8.5 Hz, 2H), 7.35 (d, J = 8.5 Hz, 2H), 7.25 (dd, J = 5.8, 4.0 Hz, 3H), 7.15 (d, J = 8.3 Hz, 2H), 7.02 (dd, J = 7.9, 1.6 Hz, 2H), 6.92 (d, J = 8.0 Hz, 2H), 6.49 (d, J = 0.6 Hz, 1H), 5.46 (s, 1H), 3.05 (m, 2H), 2.32 (s, 3H).

^{13}C NMR (101 MHz, CDCl$_3$) δ 173.0, 144.3, 143.5, 140.3, 134.4, 131.2, 130.0, 129.6, 129.4, 129.1, 127.5, 127.2, 125.7, 124.1, 118.7, 100.8, 56.3, 41.7, 21.5.

HRMS (ESI, m/z): Mass calcd. for $C_{25}H_{20}BrNO_4SNa^+$ [M+Na]$^+$, 532.0189; found 532.0182.

HPLC analysis (Chiralcel IB; 25 °C, IPA/Hexane = 40/60, 0.8 mL/min, 254 nm), Rt$_1$ (major) = 23.7 min, Rt$_2$ (minor) = 18.9 min; 97:3 er.

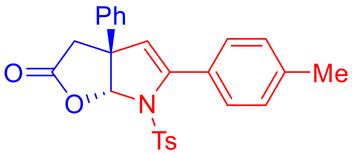

4n

(3a*R*,6a*R*)-3a-phenyl-5-(*p*-tolyl)-6-tosyl-3,3a,6,6a-tetrahydro-2*H*-furo[2,3-b]pyrrol-2-one (4n)

White solid, 73% yield, 32.5 mg, m.p. 216-217 °C.

[α]$^{25}_{D}$ = -27.6 (*c* = 0.1 in $CHCl_3$).

^{1}H NMR (400 MHz, $CDCl_3$) δ 7.39 (d, *J* = 8.1 Hz, 2H), 7.20 (ddd, *J* = 21.4, 11.4, 7.9 Hz, 7H), 7.03 (dd, *J* = 7.8, 1.6 Hz, 2H), 6.89 (d, *J* = 8.1 Hz, 2H), 6.48 (s, 1H), 5.39 (s, 1H), 3.03 (m, 2H), 2.40 (s, 3H), 2.31 (s, 3H).

^{13}C NMR (101 MHz, $CDCl_3$) δ 173.2, 144.6, 144.0, 140.7, 140.0, 134.5, 129.3, 129.0, 128.7, 128.5, 127.8, 127.3, 127.2, 125.7, 117.4, 101.1, 56.1, 42.0, 21.5, 21.4.

HRMS (ESI, m/z): Mass calcd. for $C_{26}H_{23}NO_4SNa^+$ [M+Na]$^+$, 468.1240; found 468.1242.

HPLC analysis (Chiralcel IB; 25 °C, IPA/Hexane = 40/60, 0.8 mL/min, 254 nm), Rt_1 (major) = 20.3 min, Rt_2 (minor) = 13.3 min; 99:1 er.

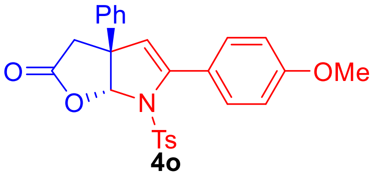

4o

(3a*R*,6a*R*)-5-(4-methoxyphenyl)-3a-phenyl-6-tosyl-3,3a,6,6a-tetrahydro-2*H*-furo[2,3-b]pyrrol-2-one (4o)

White solid, 70% yield, 31.2 mg, m.p. 202-203 °C.

[α]$^{25}_{D}$ = -25.0 (*c* = 0.1 in $CHCl_3$).

^{1}H NMR (400 MHz, $CDCl_3$) δ 7.47 – 7.38 (m, 2H), 7.29 – 7.21 (m, 3H), 7.12 (d, *J* = 8.3 Hz, 2H), 7.03 (dd, *J* = 7.9, 1.6 Hz, 2H), 6.89 (d, *J* = 8.7 Hz, 4H), 6.48 (d, *J* = 0.6 Hz, 1H), 5.34 (s, 1H), 3.86 (s, 3H), 3.13 (m, 2H), 2.31 (s, 3H).

^{13}C NMR (101 MHz, $CDCl_3$) δ 173.3, 160.8, 144.2, 144.0, 140.7, 134.5, 130.1, 129.3, 129.0, 127.3, 127.2, 125.7, 122.9, 116.6, 113.4, 101.1, 56.0, 55.3, 42.1, 21.5.

HRMS (ESI, m/z): Mass calcd. for $C_{26}H_{23}NO_5SNa^+$ [M+Na]$^+$,484.1189; found 484.1191.

HPLC analysis (Chiralcel IB; 25 °C, IPA/Hexane = 40/60, 0.8 mL/min, 254 nm), Rt_1 (major) = 24.1 min, Rt_2 (minor) = 15.9min; 98:2 er.

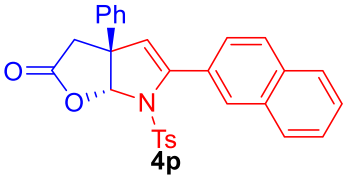

4p

(3a*R*,6a*R*)-5-(naphthalen-2-yl)-3a-phenyl-6-tosyl-3,3a,6,6a-tetrahydro-2*H*-furo[2,3-b]pyrrol-2-one (4p)

White solid, 62% yield, 29.9 mg, m.p. 210-212 °C.

[α]$^{25}_{D}$ = -17.2 (*c* = 0.5 in $CHCl_3$).

^{1}H NMR (400 MHz, $CDCl_3$) δ 7.94 – 7.73 (m, 4H), 7.61 – 7.48 (m, 3H), 7.30 – 7.22 (m, 3H), 7.16 (d, *J* = 8.3 Hz, 2H), 7.11 (dd, *J* = 6.4, 3.2 Hz, 2H), 6.88 (d, *J* = 8.0 Hz, 2H), 6.57 (s, 1H), 5.55 (s, 1H), 3.08 (m, 2H), 2.31 (s, 3H).

^{13}C NMR (101 MHz, $CDCl_3$) δ 173.2, 144.5, 144.1, 140.5, 134.7, 133.8, 132.5, 129.3, 129.2, 128.3, 128.2, 128.0, 127.8, 127.6, 127.5, 127.3, 127.0, 126.5, 125.9, 125.8, 118.7, 100.9, 56.3, 41.8, 21.5.

HRMS (ESI, m/z): Mass calcd. for $C_{29}H_{23}NO_4SNa^+$ [M+Na]$^+$, 504.1240; found 504.1238.

HPLC analysis (Chiralcel IB; 25 °C, IPA/Hexane = 40/60, 0.8 mL/min, 254 nm), Rt_1 (major) = 25.2 min, Rt_2 (minor) = 15.9 min; 98:2 er.

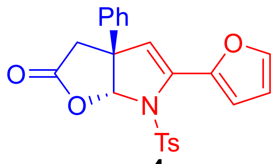

4q

(3a*R*,6a*R*)-5-(furan-2-yl)-3a-phenyl-6-tosyl-3,3a,6,6a-tetrahydro-2*H*-furo[2,3-b]pyrrol-2-one (4q)

White solid, 88% yield, 37.1 mg, m.p. 173-174 °C.

[α]$^{25}_{D}$ = +15.4 (*c* = 0.4 in $CHCl_3$).

^{1}H NMR (500 MHz, $CDCl_3$) δ 7.44 (d, *J* = 1.6 Hz, 1H), 7.24 (t, *J* = 7.6 Hz, 5H), 7.02 (dd, *J* = 7.8, 1.5 Hz, 2H), 6.93 (d, *J* = 8.3 Hz, 2H), 6.88 (d, *J* = 3.5 Hz, 1H), 6.51 (s, 1H), 6.49 (dd, *J* = 3.5, 1.8 Hz, 1H), 5.72 (s, 1H), 3.18 (m, 2H), 2.32 (s, 3H).

^{13}C NMR (101 MHz, $CDCl_3$) δ 172.9, 144.4, 144.2, 143.5, 140.2, 134.3, 134.2, 129.4, 129.1, 127.5, 127.1, 125.8, 116.6, 113.3, 111.7, 101.1, 56.1, 42.0, 21.5.

HRMS (ESI, m/z): Mass calcd. for $C_{23}H_{19}NO_5SNa^+$ [M+Na]$^+$, 444.0876; found 444.0894.

HPLC analysis (Chiralcel IB; 25 °C, IPA/Hexane = 40/60, 0.5 mL/min, 254 nm), Rt$_1$ (major) = 31.4 min, Rt$_2$ (minor) = 16.1 min; 96:4 er.

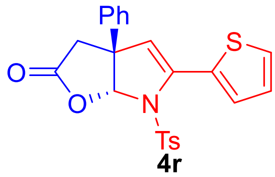

4r

(3a*R*,6a*R*)-3a-phenyl-5-(thiophen-2-yl)-6-tosyl-3,3a,6,6a-tetrahydro-2*H*-furo[2,3-b]pyrrol-2-one (4r)

White solid, 75% yield, 31.6 mg, m.p. 188-189 °C.

[α]$^{25}_{D}$ = -90.6 (*c* = 0.5 in $CHCl_3$).

^{1}H NMR (400 MHz, $CDCl_3$) δ 7.38 – 7.32 (m, 2H), 7.27 – 7.22 (m, 3H), 7.18 (d, *J* = 8.3 Hz, 2H), 7.08 – 7.00 (m, 3H), 6.91 (d, *J* = 8.1 Hz, 2H), 6.52 (s, 1H), 5.49 (s, 1H), 3.12 (m, 2H), 2.32 (s, 3H).

^{13}C NMR (101 MHz, $CDCl_3$) δ 173.0, 144.2, 140.3, 137.6, 134.4, 131.2, 130.1, 129.4, 129.1, 127.5, 127.4, 127.3, 127.0, 125.8, 117.9, 100.9, 56.1, 41.9, 21.5.

HRMS (ESI, m/z): Mass calcd. for $C_{23}H_{19}NO_4S_2Na^+$ [M+Na]$^+$, 460.0648; found 460.0650.

HPLC analysis (Chiralcel IB; 25 °C, IPA/Hexane = 40/65, 0.8 mL/min, 254 nm), Rt$_1$ (major) = 23.3 min, Rt$_2$ (minor) =16.6 min; 97:3 er.

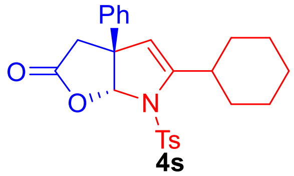

4s

(3a*R*,6a*R*)-5-cyclohexyl-3a-phenyl-6-tosyl-3,3a,6,6a-tetrahydro-2*H*-furo[2,3-b]pyrrol-2-one (4s)

White solid, 55% yield, 24.1 mg, m.p. 205-206 °C.

[α]$^{25}_{D}$ = +48.9 (*c* = 0.2 in $CHCl_3$).

^{1}H NMR (400 MHz, $CDCl_3$) δ 7.50 (d, *J* = 8.3 Hz, 2H), 7.25 – 7.21 (m, 3H), 7.09 – 7.00 (m, 4H), 6.36 (s, 1H), 5.08 (s, 1H), 3.00 (m, 2H), 2.64 (t, *J* = 11.0 Hz, 1H), 2.35 (s, 3H), 2.05 – 1.78 (m, 3H), 1.68 (dd, *J* = 21.8, 8.0 Hz, 2H), 1.31 (ddd, *J* = 14.8, 12.2, 6.3 Hz, 2H), 1.18 (dt, *J* = 12.3, 5.2 Hz, 2H), 0.90 (qd, *J* = 12.3, 3.4 Hz, 1H).

^{13}C NMR (101 MHz, $CDCl_3$) δ 173.3, 150.2, 144.0, 140.4, 135.9, 129.6, 129.0, 127.4, 127.0, 125.7, 112.8, 100.1, 55.3, 41.3, 36.7, 33.7, 31.1, 26.2, 26.1, 26.0, 21.5.

HRMS (ESI, m/z): Mass calcd. for $C_{25}H_{27}NO_4SNa^+$ [M+Na]$^+$, 460.1553; found 460.1551.

HPLC analysis (Chiralcel IB; 25 °C, IPA/Hexane = 40/60, 0.8 mL/min, 254 nm), Rt$_1$ (major) = 14.5 min, Rt$_2$ (minor) = 10.9 min; 99:1 er.

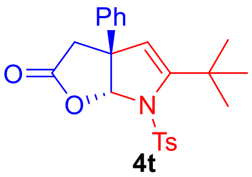

4t

(3a*R*,6a*R*)-5-(*tert*-butyl)-3a-phenyl-6-tosyl-3,3a,6,6a-tetrahydro-2*H*-furo[2,3-b]pyrrol-2-one (4t)

White solid, 47% yield, 19.3 mg, m.p. 249-250 °C.

[α]$^{25}_{D}$ = -105.8 (c = 0.5 in $CHCl_3$).

^{1}H NMR (400 MHz, $CDCl_3$) δ 7.36 (d, J = 8.4 Hz, 2H), 7.23 – 7.15 (m, 3H), 7.04 – 6.97 (m, 2H), 6.88 (d, J = 8.0 Hz, 2H), 6.36 (d, J = 0.8 Hz, 1H), 5.35 (d, J = 0.8 Hz, 1H), 2.98 (m, 2H), 2.27 (s, 3H), 1.38 (s, 9H).

^{13}C NMR (101 MHz, $CDCl_3$) δ 173.0, 154.7, 143.8, 140.1, 135.7, 129.4, 129.0, 127.2, 126.9, 126.0, 117.6, 101.9, 54.9, 42.4, 34.3, 30.2, 21.4.

HRMS (ESI, m/z): Mass calcd. for $C_{23}H_{25}NO_4SNa^+$ $[M+Na]^+$, 434.1397; found 434.1399.

HPLC analysis (Chiralcel IB; 25 °C, IPA/Hexane = 40/60, 0.8 mL/min, 254 nm), Rt_1 (major) = 15.1 min, Rt_2 (minor) = 12.5 min; 94:6 er.

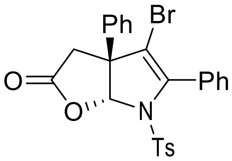

5

(3a*R*,6a*R*)-4-bromo-3a,5-diphenyl-6-tosyl-3,3a,6,6a-tetrahydro-2*H*-furo [2,3-b]pyrrol-2-one (5)

White solid, 87% yield, 31.3 mg, m.p. 177-178°C.

[α]$^{25}_{D}$ = +196.2 (c = 1.0 in $CHCl_3$).

^{1}H NMR (400 MHz, $CDCl_3$) δ 7.44 – 7.38 (m, 1H), 7.35 – 7.29 (m, 7H), 7.25 – 7.16 (m, 4H), 7.02 (d, J = 8.2 Hz, 2H), 6.58 (s, 1H), 3.26 (m, 2H), 2.37 (s, 3H).

^{13}C NMR (101 MHz, $CDCl_3$) δ 172.1, 144.5, 140.2, 137.9, 135.2, 130.2, 129.9, 129.5, 129.3, 128.8, 128.1, 127.8, 127.5, 126.0, 111.2, 97.9, 59.9, 38.6, 21.6.

HRMS (ESI, m/z): Mass calcd. for $C_{25}H_{20}NO_4NaBr^+$ $[M+Na]^+$, 532.0189; found 532.0188.

HPLC analysis (Chiralcel IB; 25 °C, IPA/Hexane = 40/60, 0.8 mL/min, 254 nm), Rt_1 (major) = 9.8 min, Rt_2 (minor) = 14.7 min; 99:1 er.

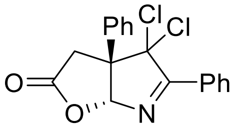

6

(3a*R*,6a*R*)-4,4-dichloro-3a,5-diphenyl-3,3a,4,6a-tetrahydro-2*H*-furo[2,3-b] pyrrol-2-one (6)

White solid, 73% yield, 27.1 mg, m.p. 118-119 °C.

[α]$^{25}_{D}$ = +192.9 (c = 1.0 in $CHCl_3$).

^{1}H NMR (400 MHz, $CDCl_3$) δ 8.31 – 8.11 (m, 2H), 7.67 – 7.56 (m, 3H), 7.48 (ddd, J = 11.1, 8.3, 5.3 Hz, 5H), 6.66 (s, 1H), 3.06 (m, 2H).

^{13}C NMR (101 MHz, $CDCl_3$) δ 171.8, 171.0, 136.0, 132.6, 129.6, 129.3, 129.1, 128.6, 128.3, 126.7, 100.0, 90.8, 66.6, 42.9.

HRMS (ESI, m/z): Mass calcd. for $C_{18}H_{13}NO_2Cl_2Na^+$ $[M+Na]^+$, 368.0216; found 368.0215.

HPLC analysis (Chiralcel IB; 25 °C, IPA/Hexane = 40/60, 0.8 mL/min, 254 nm), Rt_1 (major) = 8.7 min, Rt_2 (minor) = 10.6 min; 99:1 er.

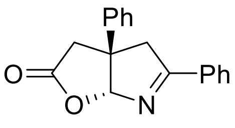

7

(3a*S*,6a*R*)-3a,5-diphenyl-3,3a,4,6a-tetrahydro-2*H*-furo[2,3-b]pyrrol-2- one (7)

White solid, 60% yield, 26.8 mg, m.p. 93-95°C.

[α]$^{25}_{D}$ = +55.8 (*c* = 1 in $CHCl_3$).

^{1}H NMR (400 MHz, $CDCl_3$) δ 8.00 – 7.90 (m, 2H), 7.59 – 7.52 (m, 1H), 7.48 (t, *J* = 7.4 Hz, 2H), 7.44 – 7.39 (m, 2H), 7.36 – 7.30 (m, 3H), 6.57 (s, 1H), 3.59 (ddd, *J* = 51.3, 17.3, 1.5 Hz, 2H), 2.90 (dd, *J* = 57.2, 18.0 Hz, 2H).

^{13}C NMR (101 MHz, $CDCl_3$) δ 175.7, 174.5, 142.7, 132.6, 132.3, 129.5, 128.8, 128.4, 127.7, 125.4, 110.1, 52.8, 48.5, 43.1.

HRMS (ESI, m/z): Mass calcd. for $C_{18}H_{16}NO_2^+$ $[M+H]^+$, 278.1176; found 278.1176.

HPLC analysis (Chiralcel IB; 25 °C, IPA/Hexane = 40/60, 0.5 mL/min, 254 nm), Rt_1 (major) = 13.7 min, Rt_2 (minor) = 15.1 min; 99:1 er.

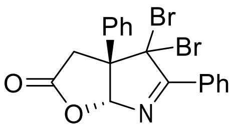

8

(3a*R*,6a*R*)-4,4-dibromo-3a,5-diphenyl-3,3a,4,6a-tetrahydro-2*H*-furo[2,3-b]pyrrol-2-one (8)

White solid, 88% yield, 38.3 mg, m.p. 125-126°C.

[α]$^{25}_{D}$ = +198.7 (*c* = 1.0 in $CHCl_3$).

^{1}H NMR (400 MHz, $CDCl_3$) δ 8.35 – 8.17 (m, 2H), 7.77 – 7.66 (m, 2H), 7.63 – 7.55 (m, 1H), 7.54 – 7.41 (m, 5H), 6.63 (s, 1H), 3.07 (m, 2H).

^{13}C NMR (101 MHz, $CDCl_3$) δ 171.8, 171.4, 137.5, 132.4, 129.8, 129.2, 129.1, 128.4, 126.7, 99.5, 66.8, 65.4, 44.3.

HRMS (ESI, m/z): Mass calcd. for $C_{18}H_{13}NO_2NaBr_2^+$ $[M+Na]^+$, 455.9205; found 455.9204.

HPLC analysis (Chiralcel IB; 25 °C, IPA/Hexane = 40/60, 0.8 mL/min, 254 nm), Rt_1 (major) = 9.4 min, Rt_2 (minor) = 14.2 min; 99:1 er.

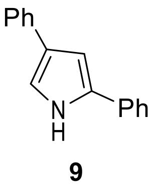

9

2,4-diphenyl-1*H*-pyrrole (9)

White solid, 76% yield, 16.1 mg, m.p. 179-180°C.

^{1}H NMR (400 MHz, $CDCl_3$) δ 8.45 (s, 1H), 7.54 (ddd, *J* = 22.3, 8.2, 1.0 Hz, 4H), 7.38 (ddd, *J* = 13.4, 9.6, 4.9 Hz, 4H), 7.21 (dd, *J* = 15.0, 7.5 Hz, 2H), 7.14 (dd, *J* = 2.6, 1.8 Hz, 1H), 6.83 (dd, *J* = 2.7, 1.7 Hz, 1H).

^{13}C NMR (101 MHz, $CDCl_3$) δ 135.5, 133.1, 132.5, 128.9, 128.6, 126.6, 126.5, 125.7, 125.2, 123.8, 115.5, 104.1.

IV. Supplementary Figures

Stereochemistry determination via X-ray crystallographic analysis

The absolute stereochemistry was determined by the X-ray diffraction. These crystals were deposited in the Cambridge Crystallographic Data Centre and assigned.

www.ccdc.cam.ac.uk/data request/cif.

Supplementary Table 4. X-ray crystallographic analysis.

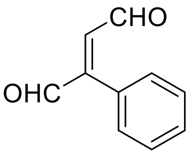 **1a**	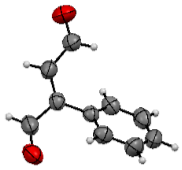 CCDC: 2156768
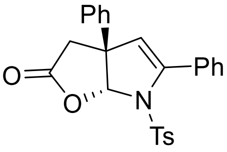 **3a**	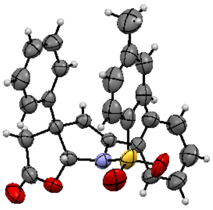 CCDC:2076667
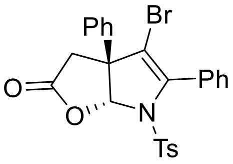 **5**	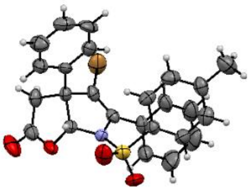 CCDC: 2156751
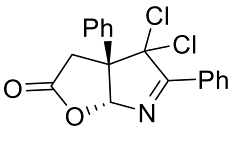 **6**	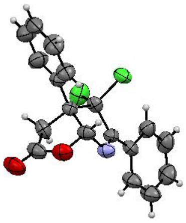 CCDC:2156752

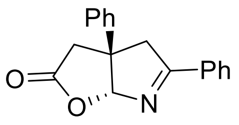 **7**	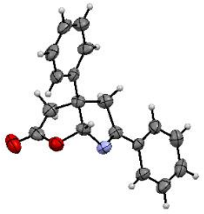<br>CCDC:2156755

^{1}H NMR, ^{13}C NMR, ^{19}F NMR and HPLC spectra

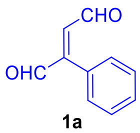

1a

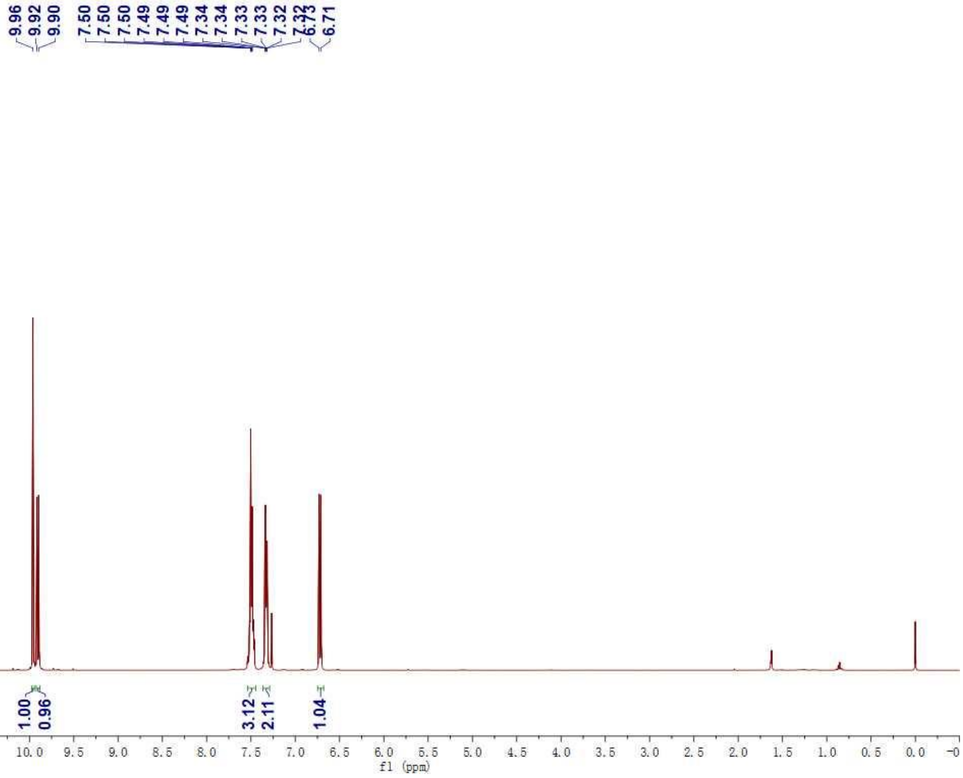

Supplementary Figure 26. ^{1}H NMR (400 MHz, $CDCl_3$) of **1a**

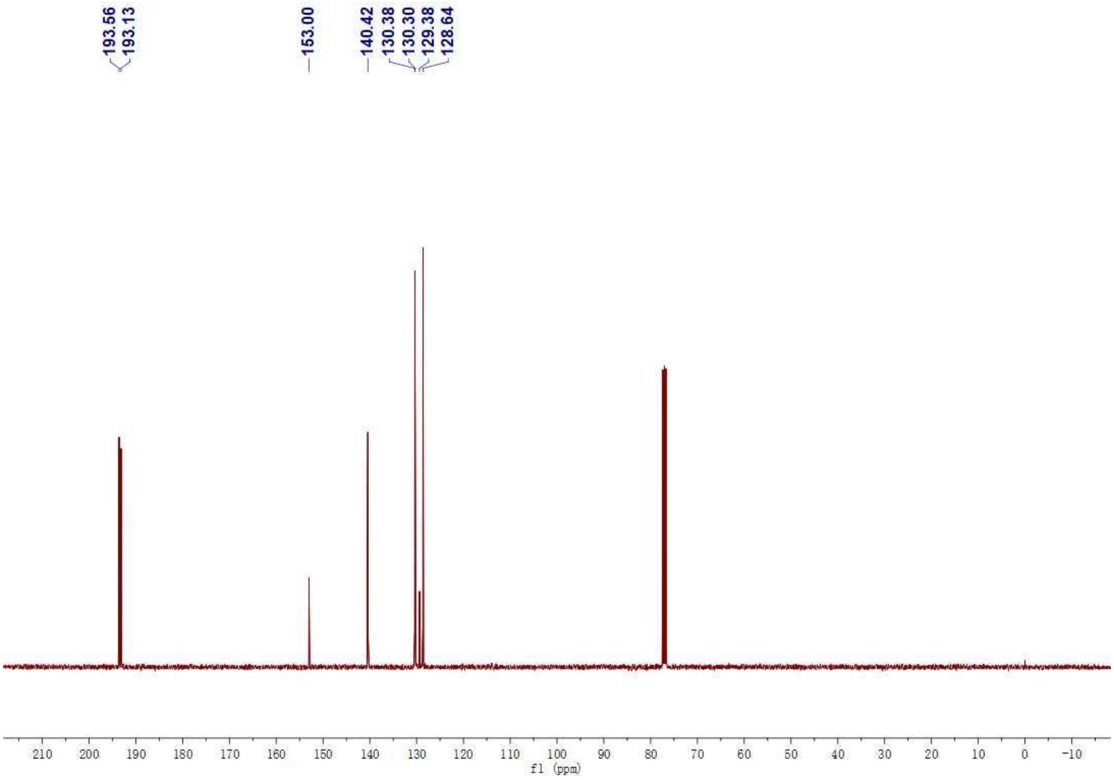

Supplementary Figure 27. ^{13}C NMR (101 MHz, $CDCl_3$) of **1a**

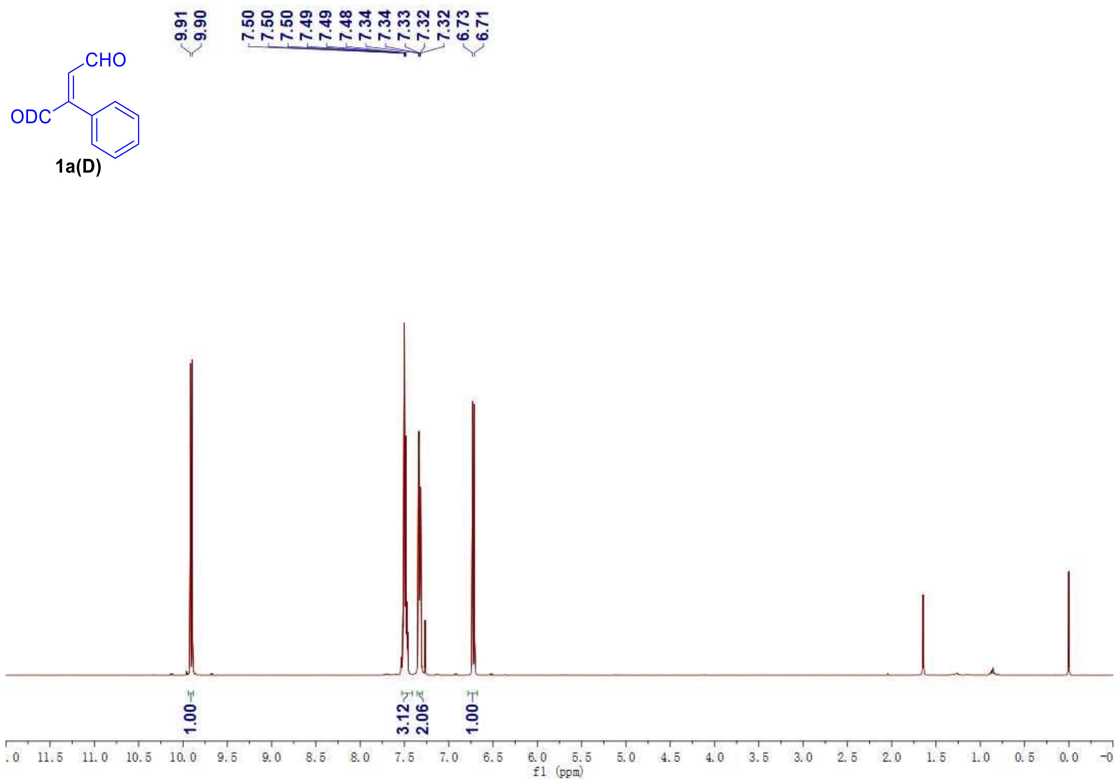

Supplementary Figure 28. ^{1}H NMR (400 MHz, $CDCl_3$) of **1a(D)**

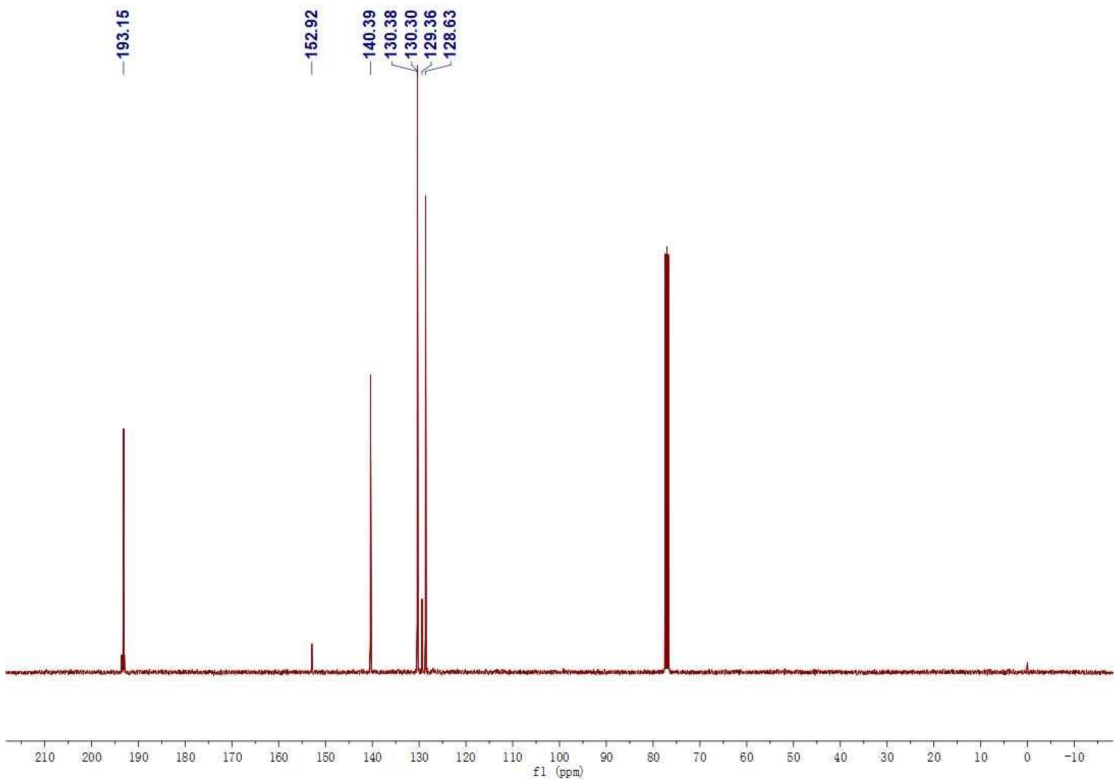

Supplementary Figure 29. ^{13}C NMR (101 MHz, $CDCl_3$) of **1a(D)**

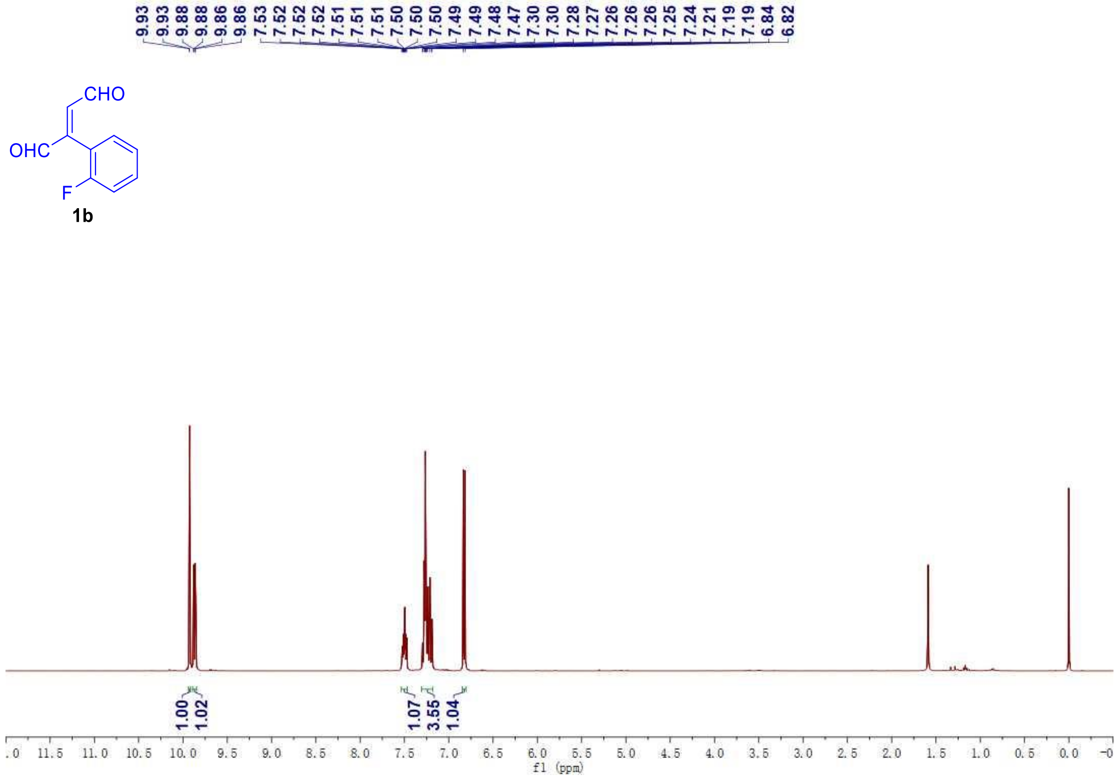

Supplementary Figure 30. ^{1}H NMR (400 MHz, $CDCl_3$) of **1b**

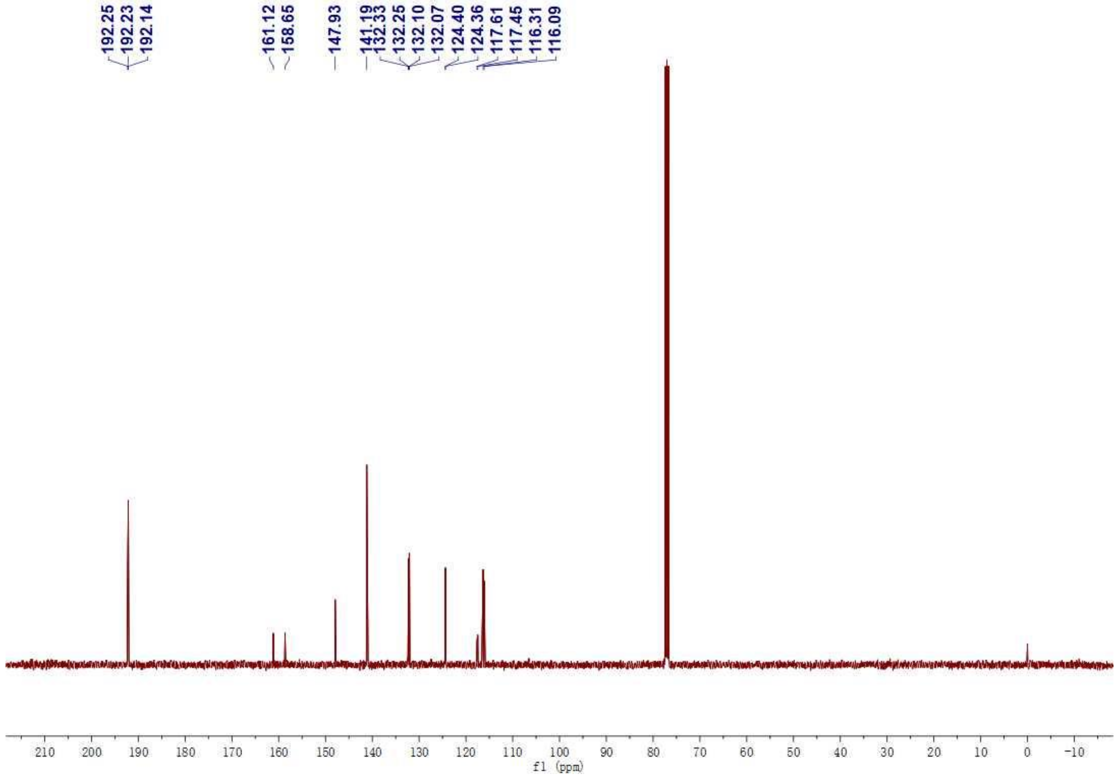

Supplementary Figure 31. ^{13}C NMR (101 MHz, $CDCl_3$) of **1b**

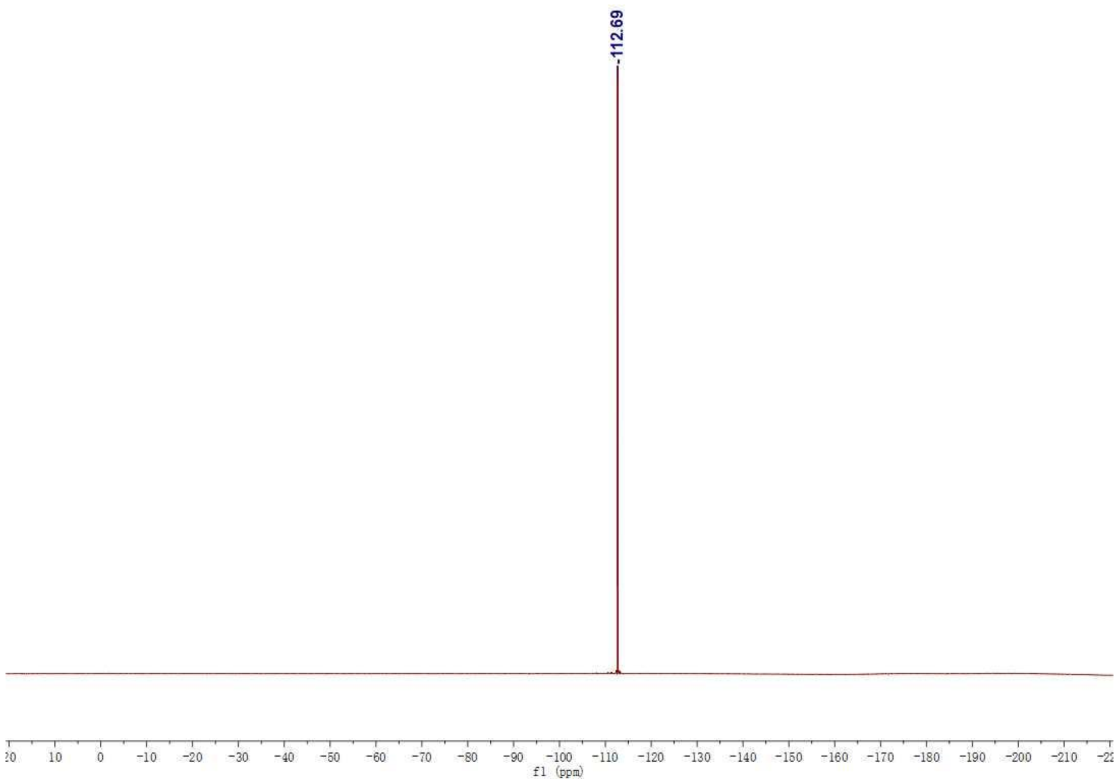

Supplementary Figure 32. ^{19}F NMR (376 MHz, $CDCl_3$) of **1b**

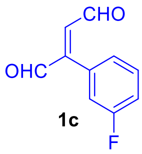

1c

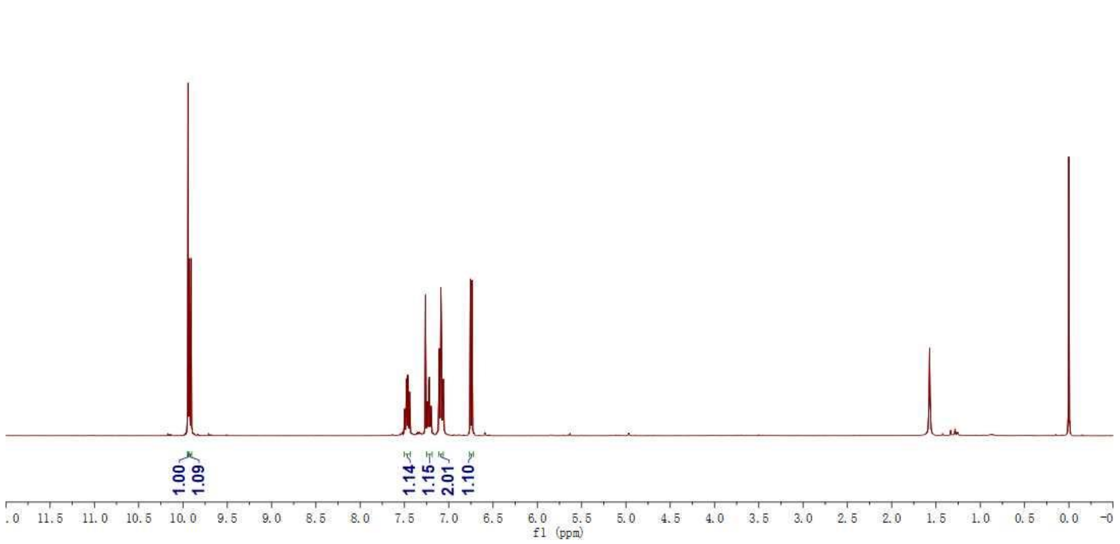

Supplementary Figure 33. ^{1}H NMR (400 MHz, $CDCl_3$) of **1c**

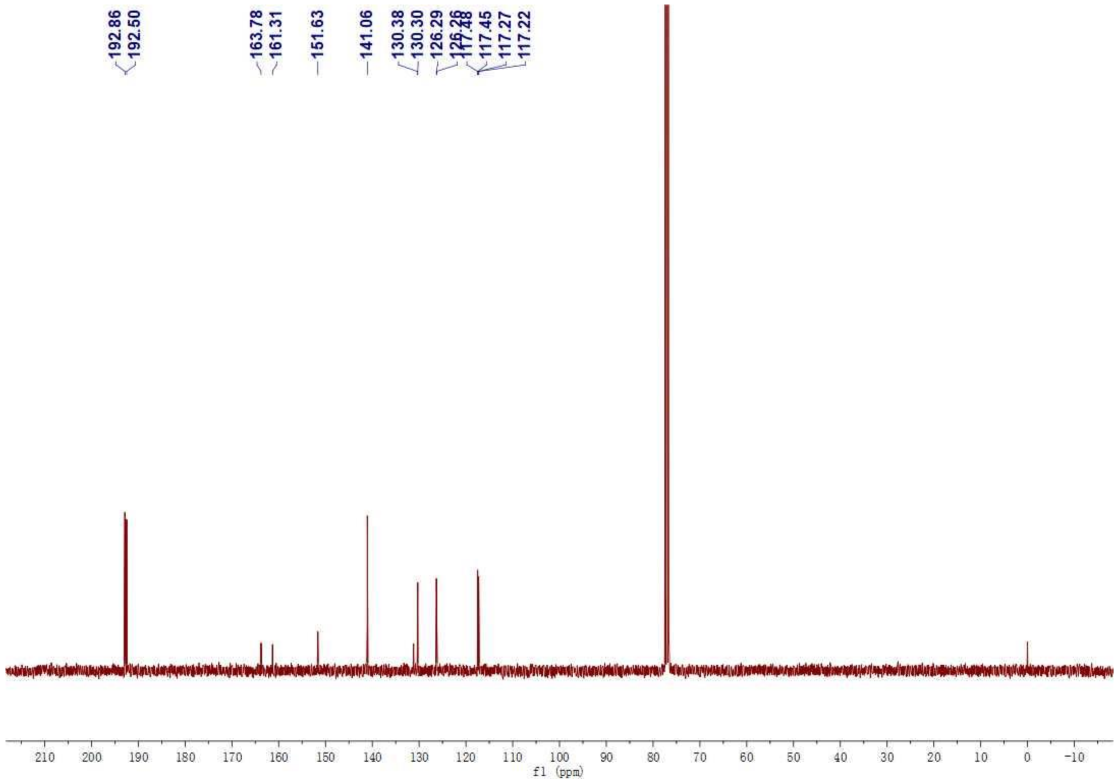

Supplementary Figure 34. ^{13}C NMR (101 MHz, $CDCl_3$) of **1c**

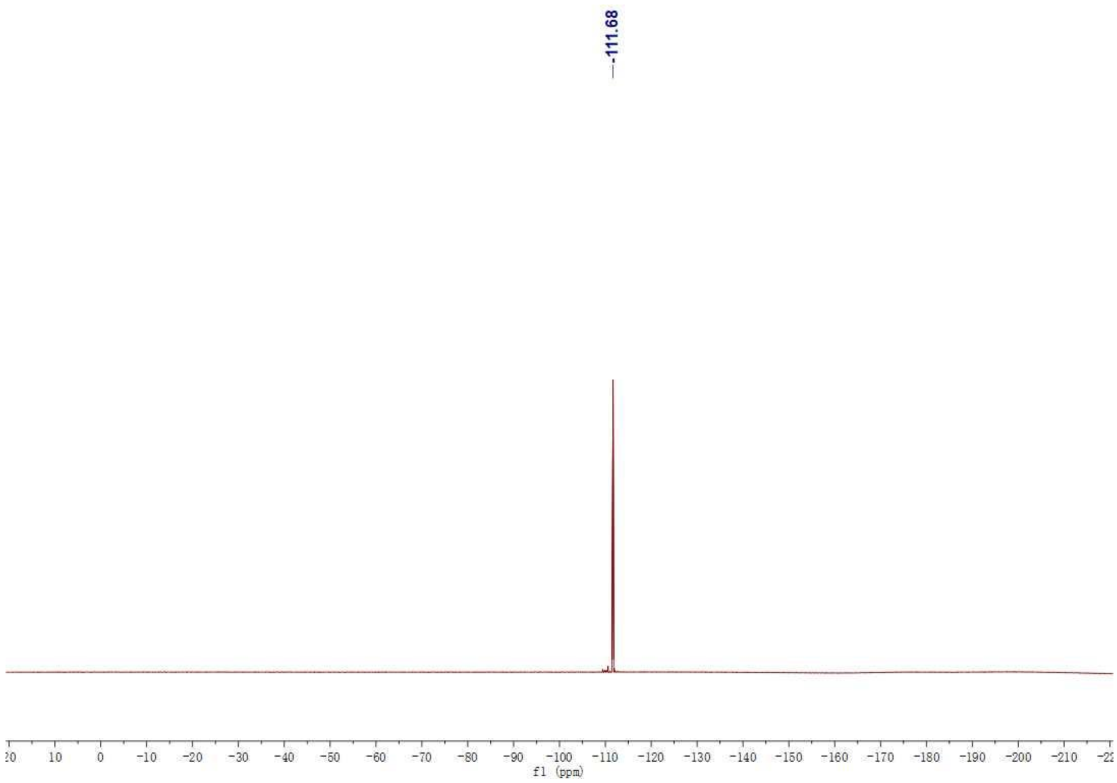

Supplementary Figure 35. ^{19}F NMR (376 MHz, $CDCl_3$) of **1c**

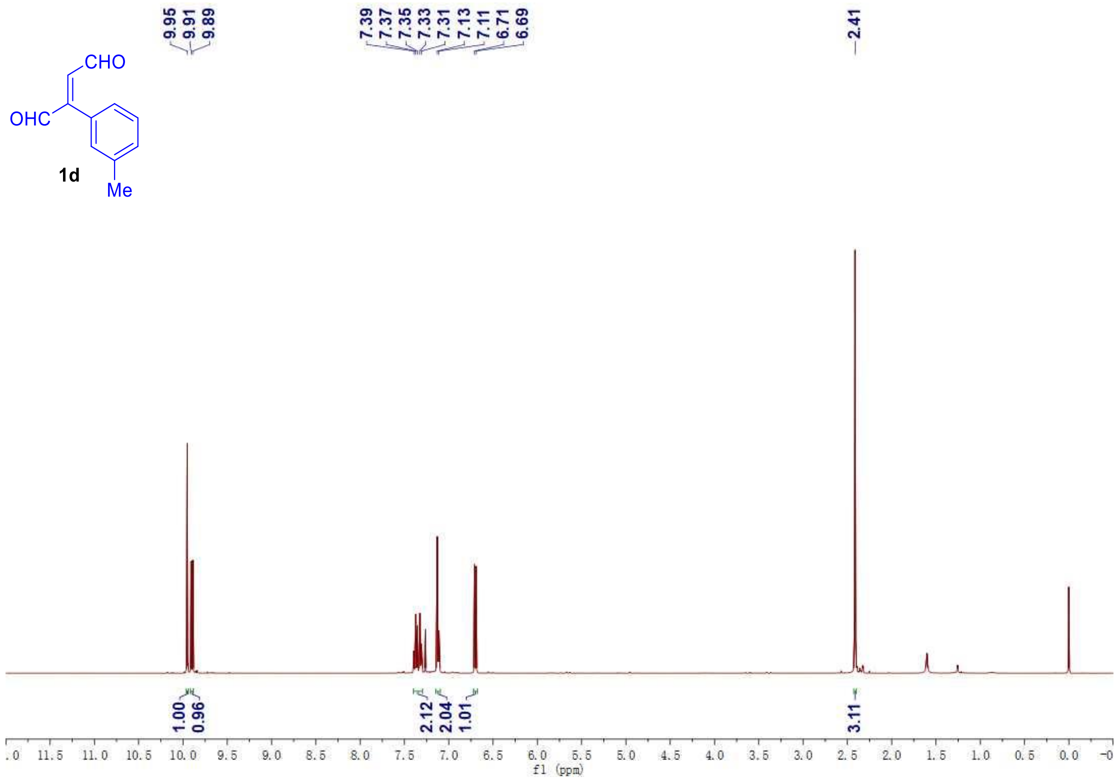

Supplementary Figure 36. ^{1}H NMR (400 MHz, $CDCl_3$) of **1d**

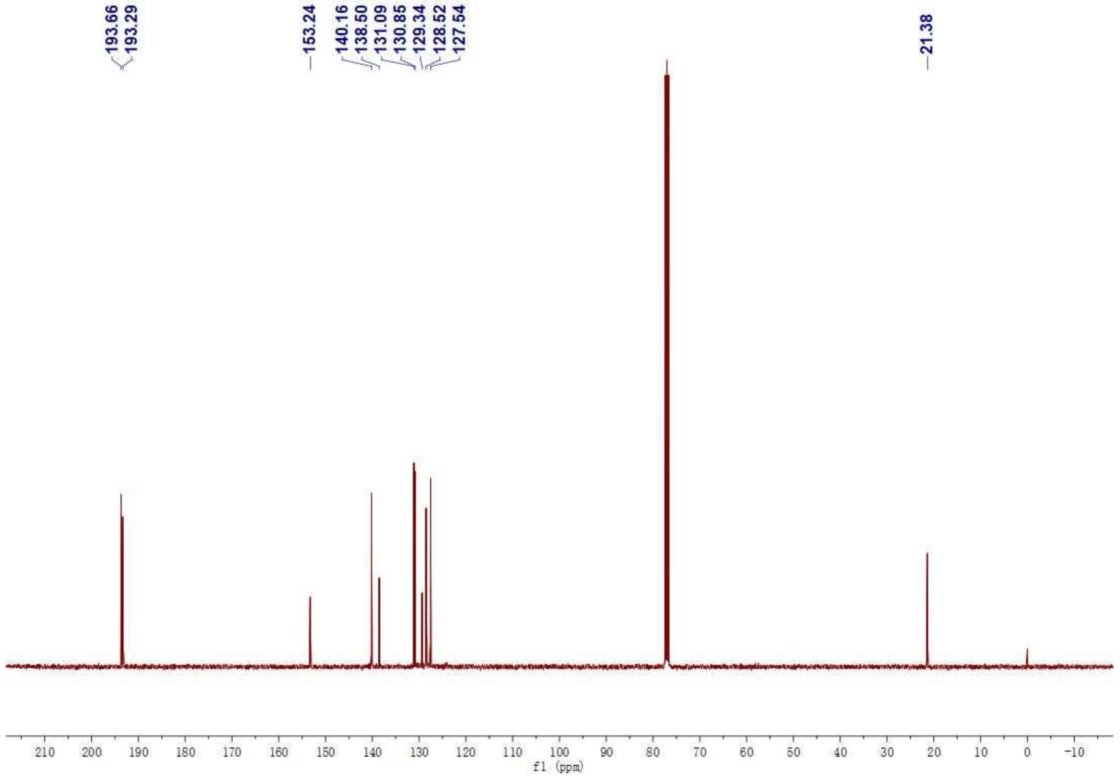

Supplementary Figure 37. ^{13}C NMR (101 MHz, $CDCl_3$) of **1d**

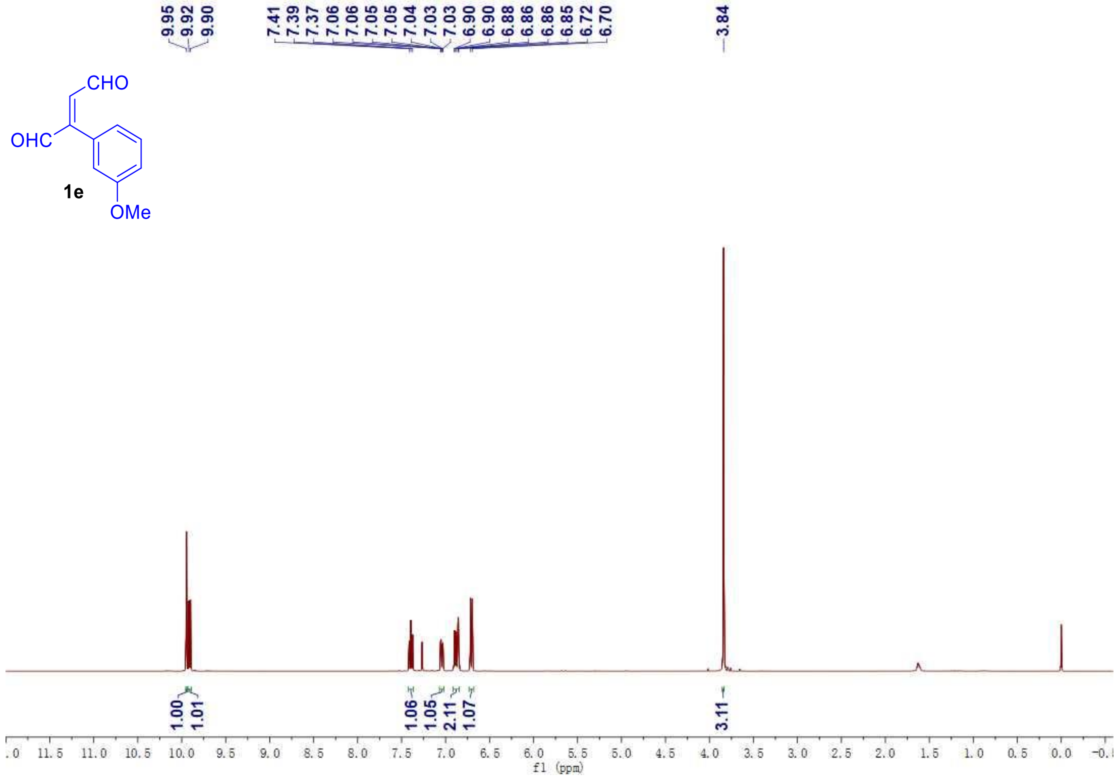

Supplementary Figure 38. ^{1}H NMR (400 MHz, $CDCl_3$) of **1e**

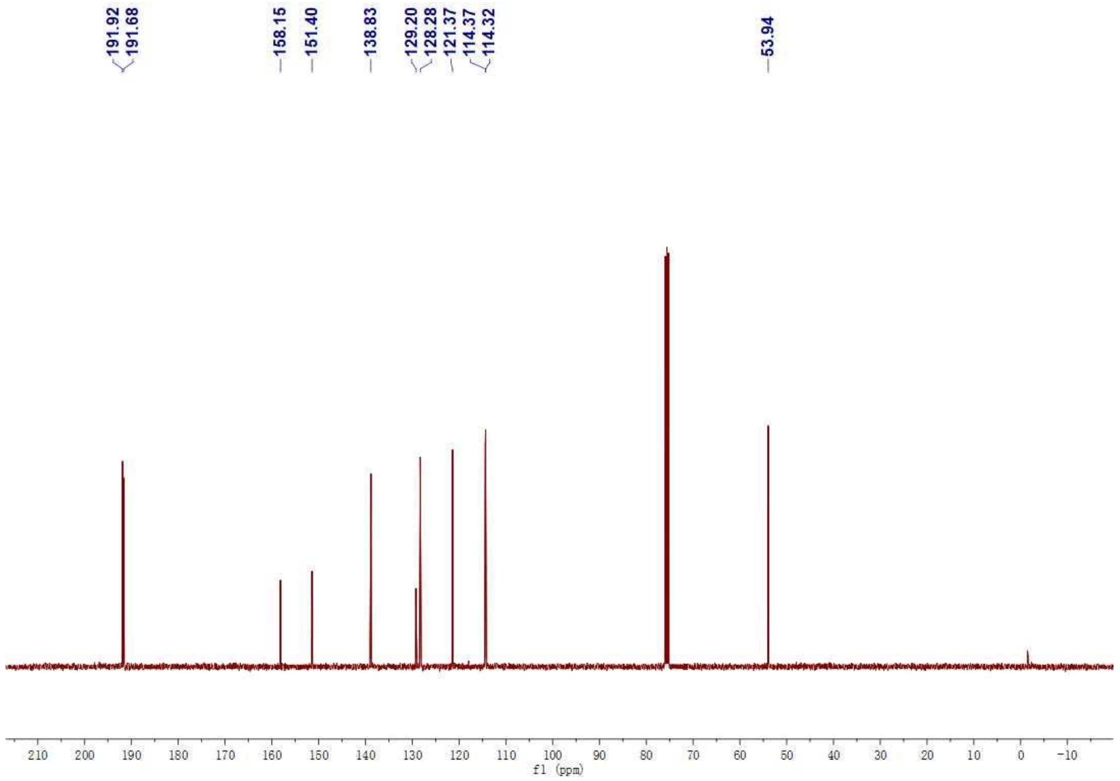

Supplementary Figure 39. ^{13}C NMR (101 MHz, $CDCl_3$) of **1e**

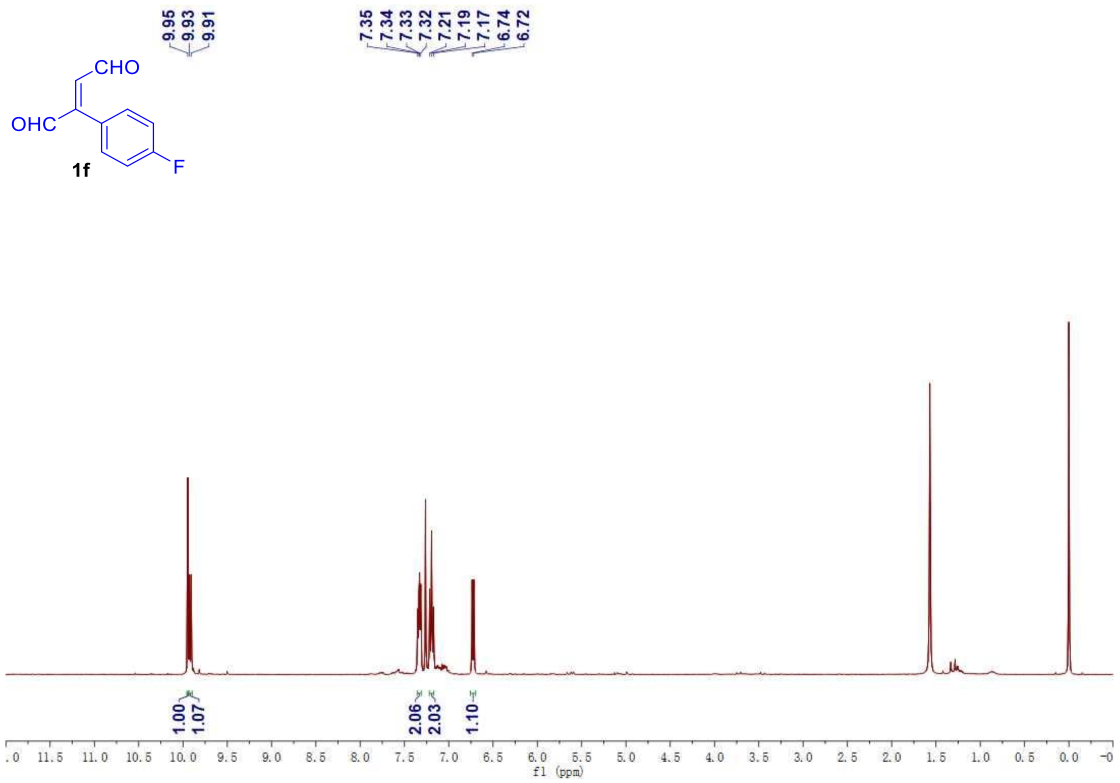

Supplementary Figure 40. ^{1}H NMR (400 MHz, $CDCl_3$) of **1f**

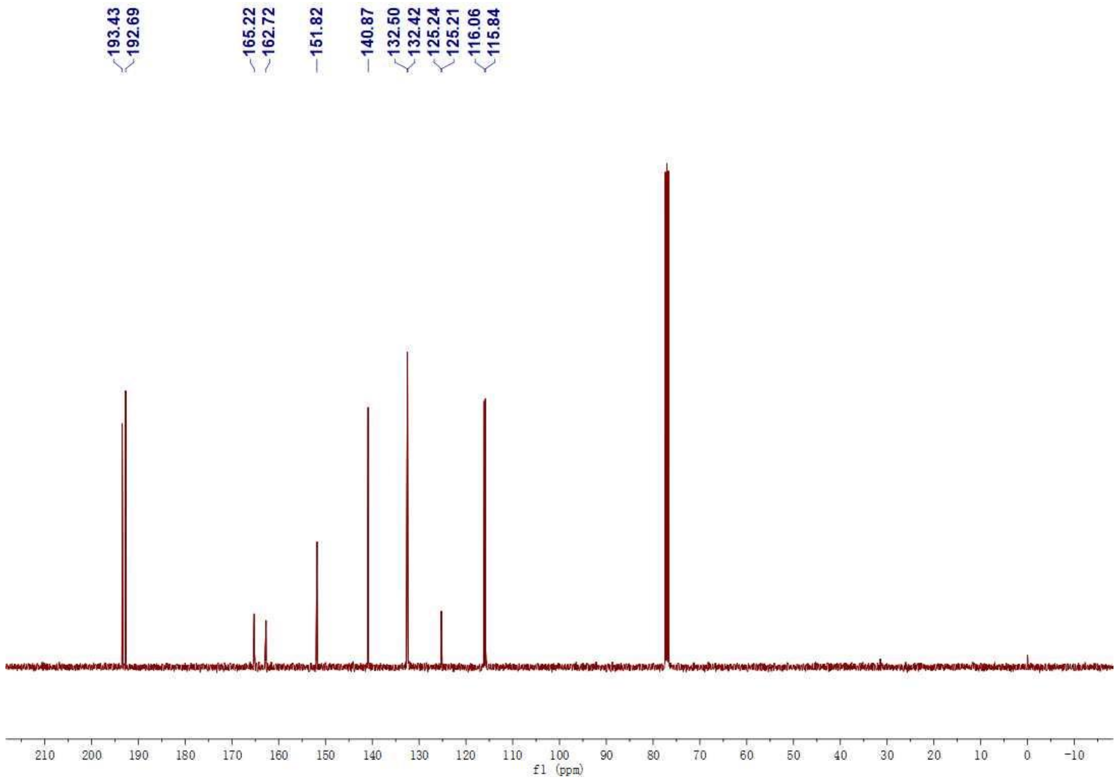

Supplementary Figure 41. ^{13}C NMR (101 MHz, $CDCl_3$) of **1f**

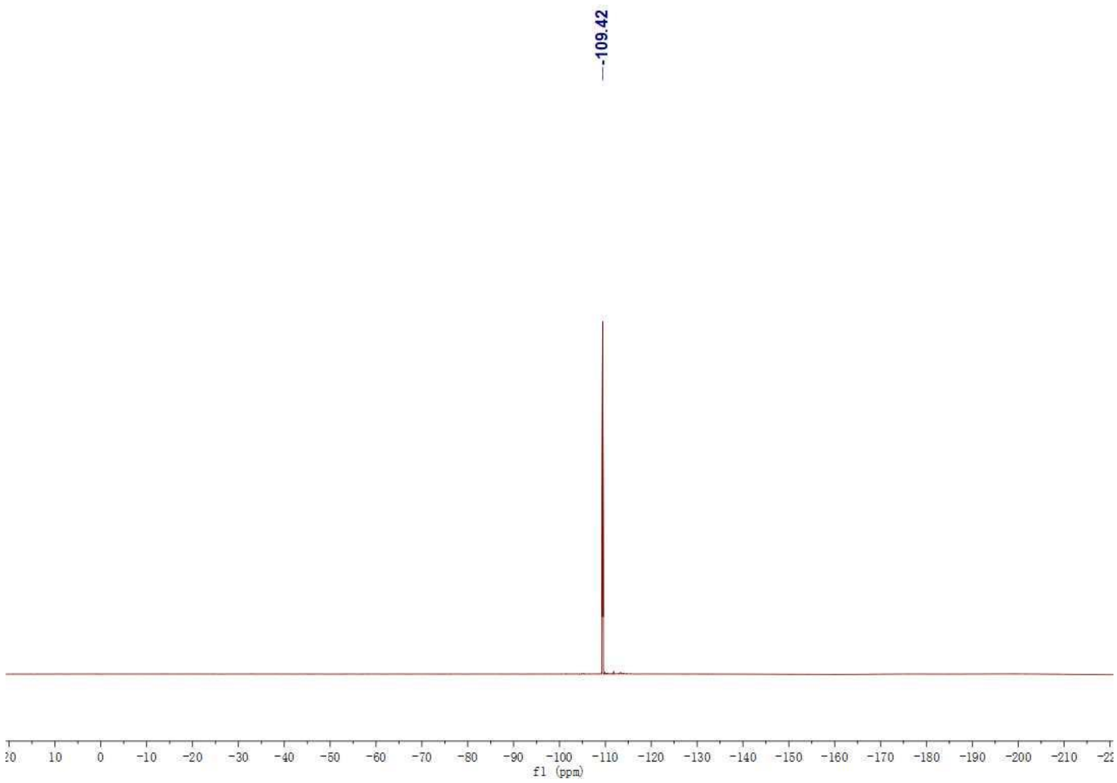

Supplementary Figure 42. ^{19}F NMR (376 MHz, $CDCl_3$) of **1f**

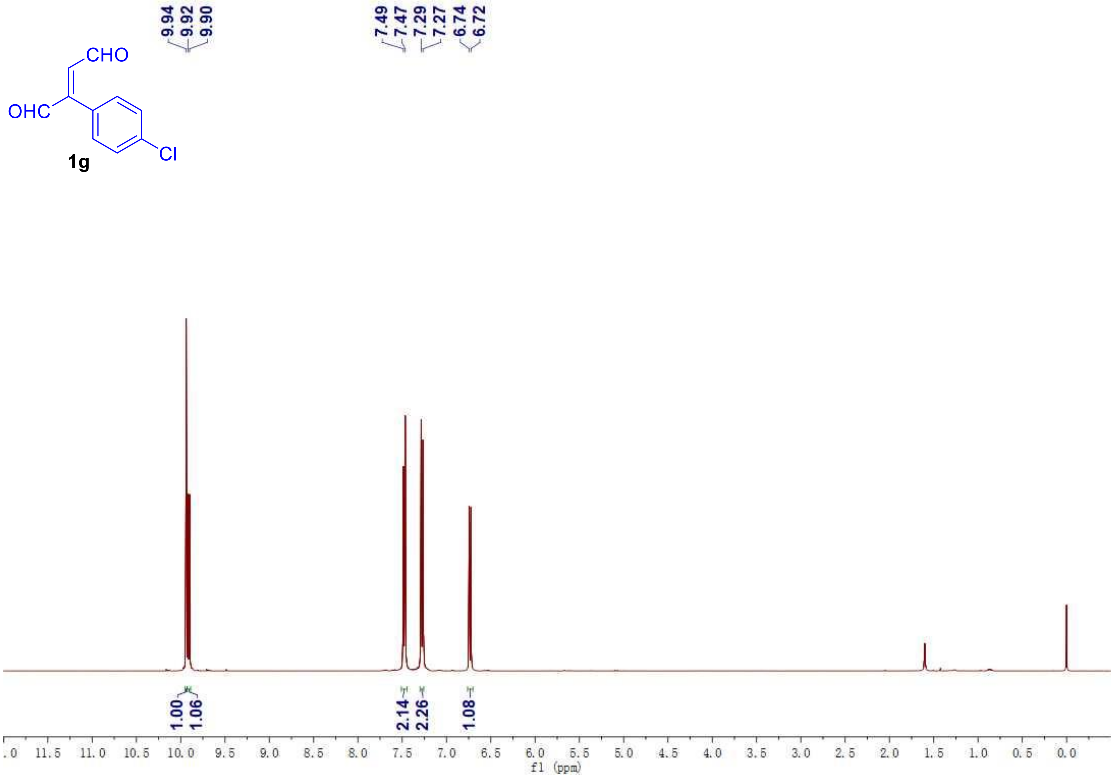

Supplementary Figure 43. ^{1}H NMR (400 MHz, $CDCl_3$) of **1g**

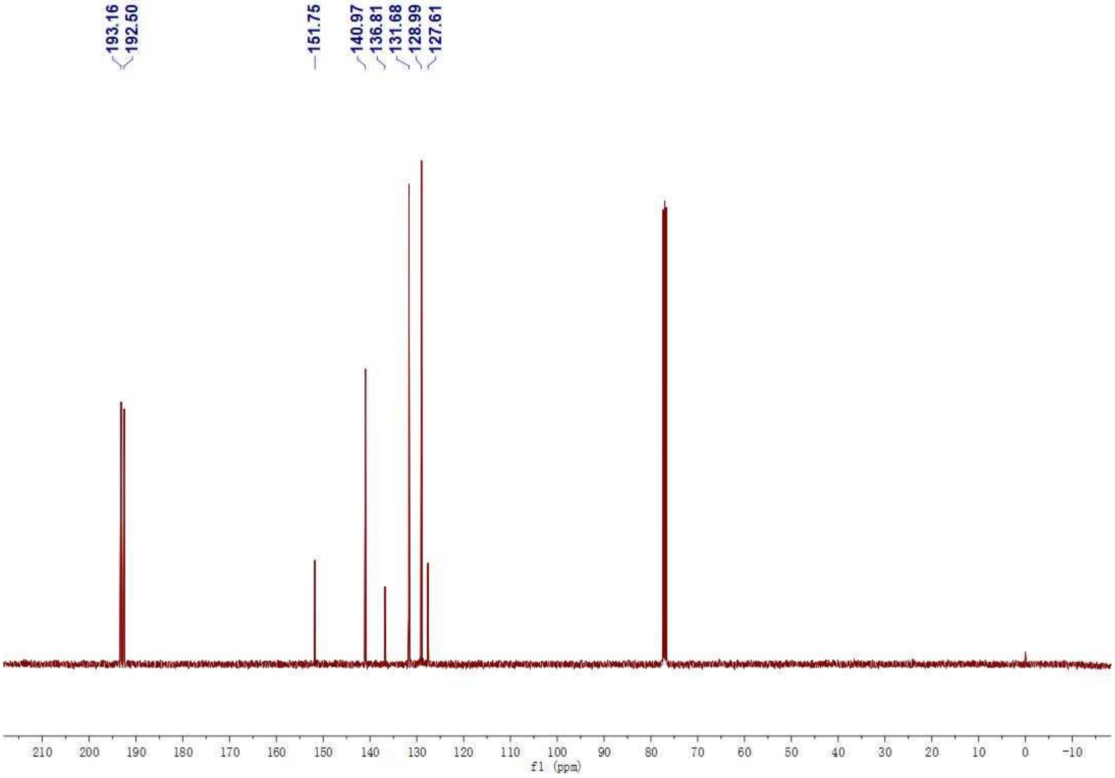

Supplementary Figure 44. ^{13}C NMR (101 MHz, $CDCl_3$) of **1g**

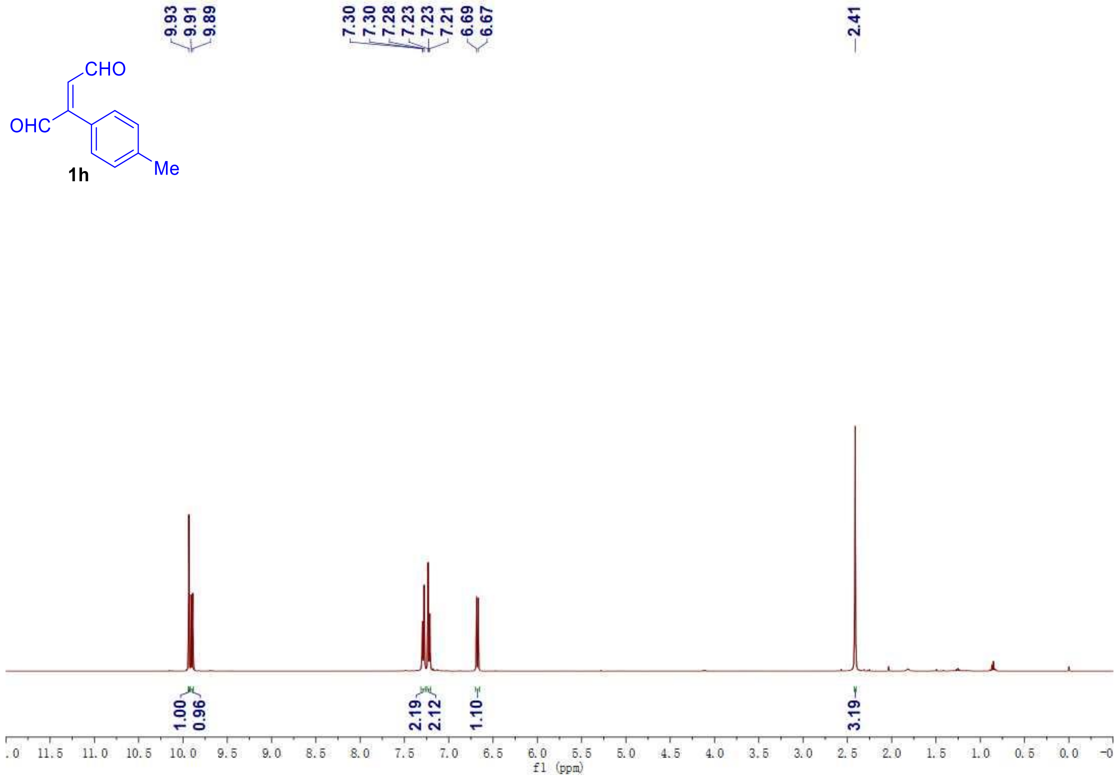

Supplementary Figure 45. ^{1}H NMR (400 MHz, $CDCl_3$) of **1h**

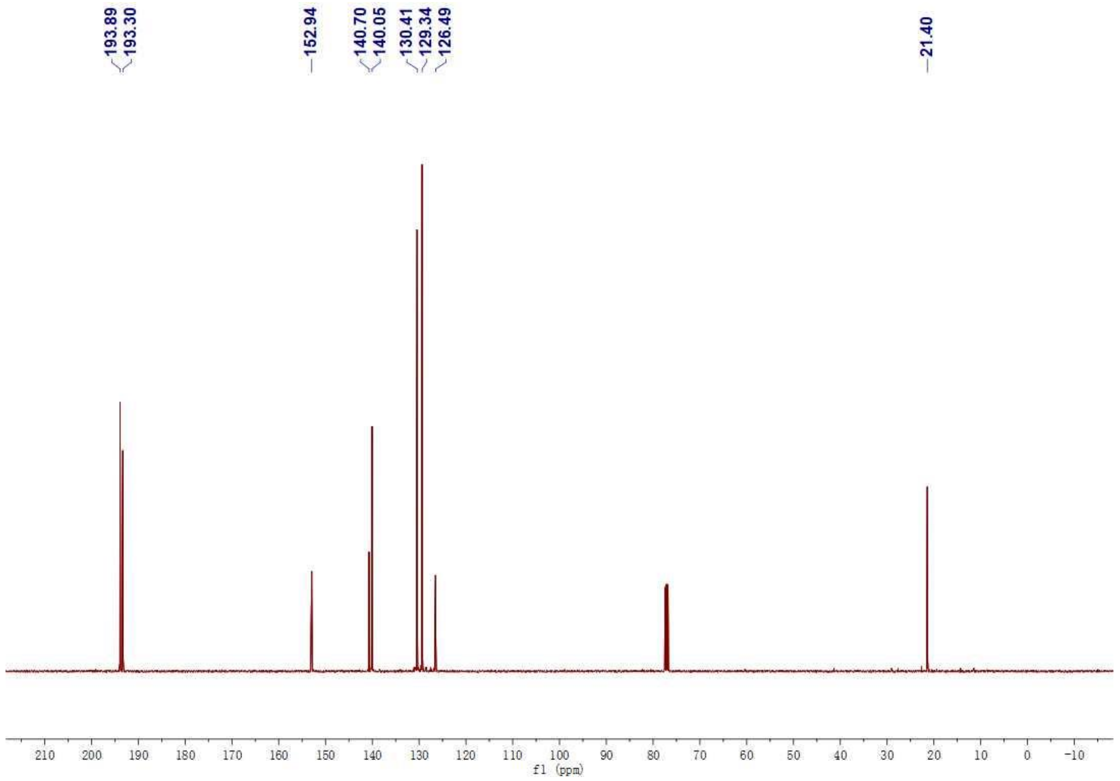

Supplementary Figure 46. ^{13}C NMR (101 MHz, $CDCl_3$) of **1h**

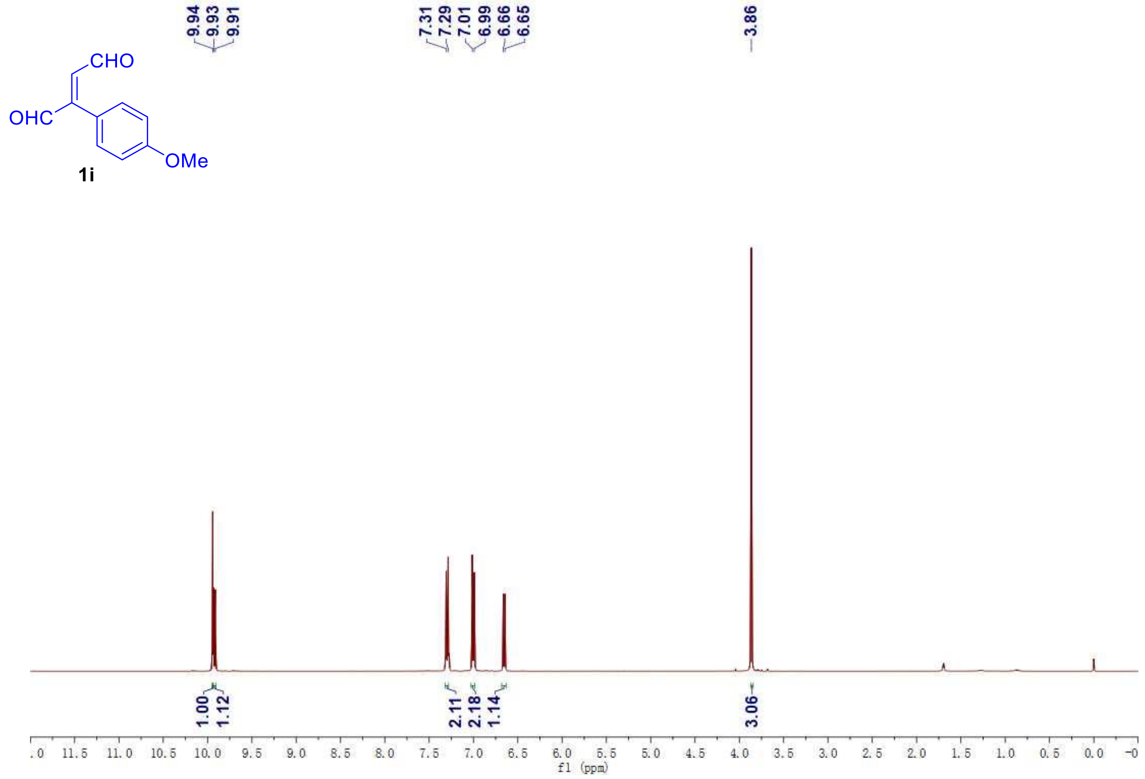

Supplementary Figure 47. ^{1}H NMR (400 MHz, $CDCl_3$) of **1i**

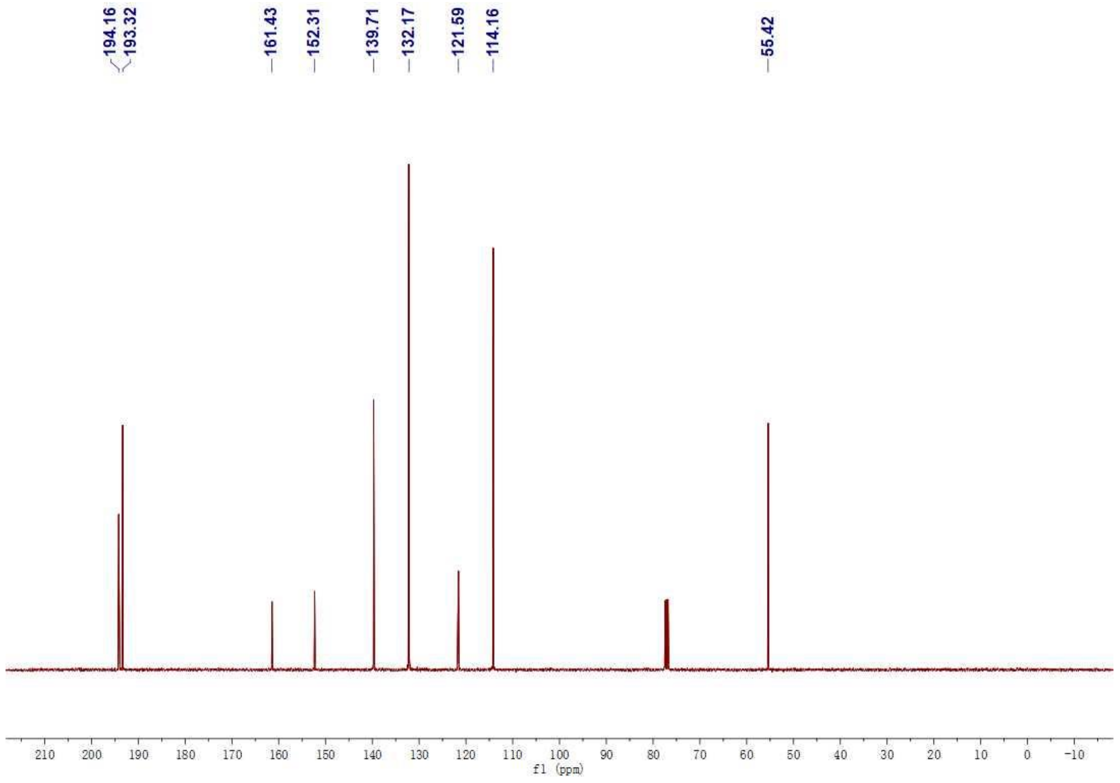

Supplementary Figure 48. ^{13}C NMR (101 MHz, $CDCl_3$) of **1i**

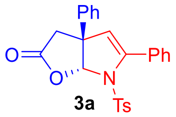

3a

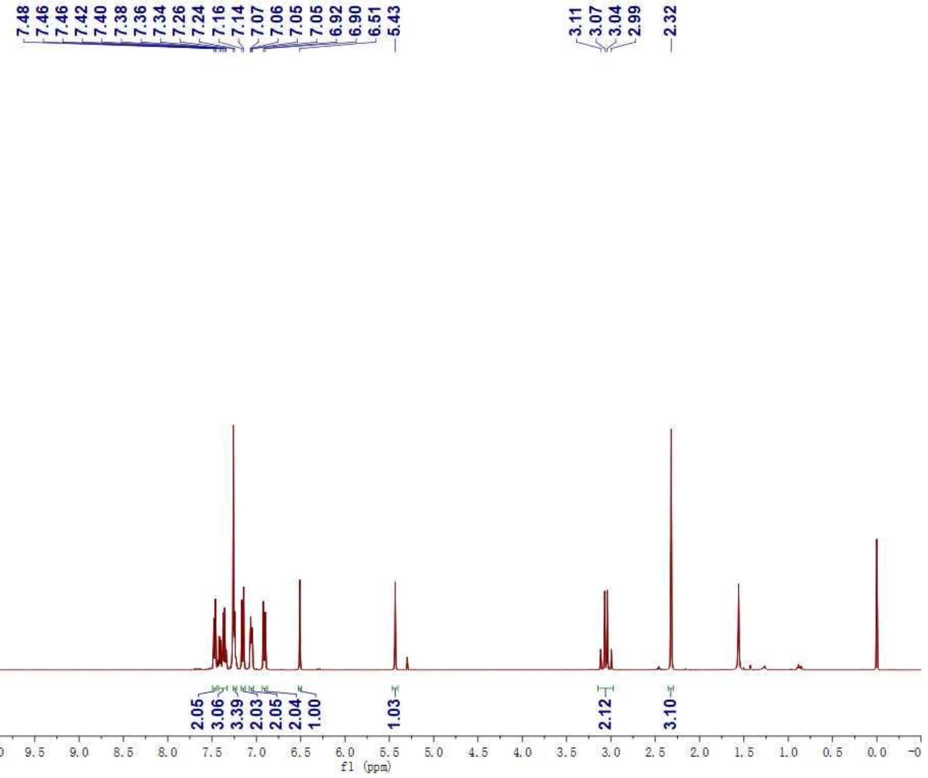

Supplementary Figure 49. ^{1}H NMR (400 MHz, $CDCl_3$) of **3a**

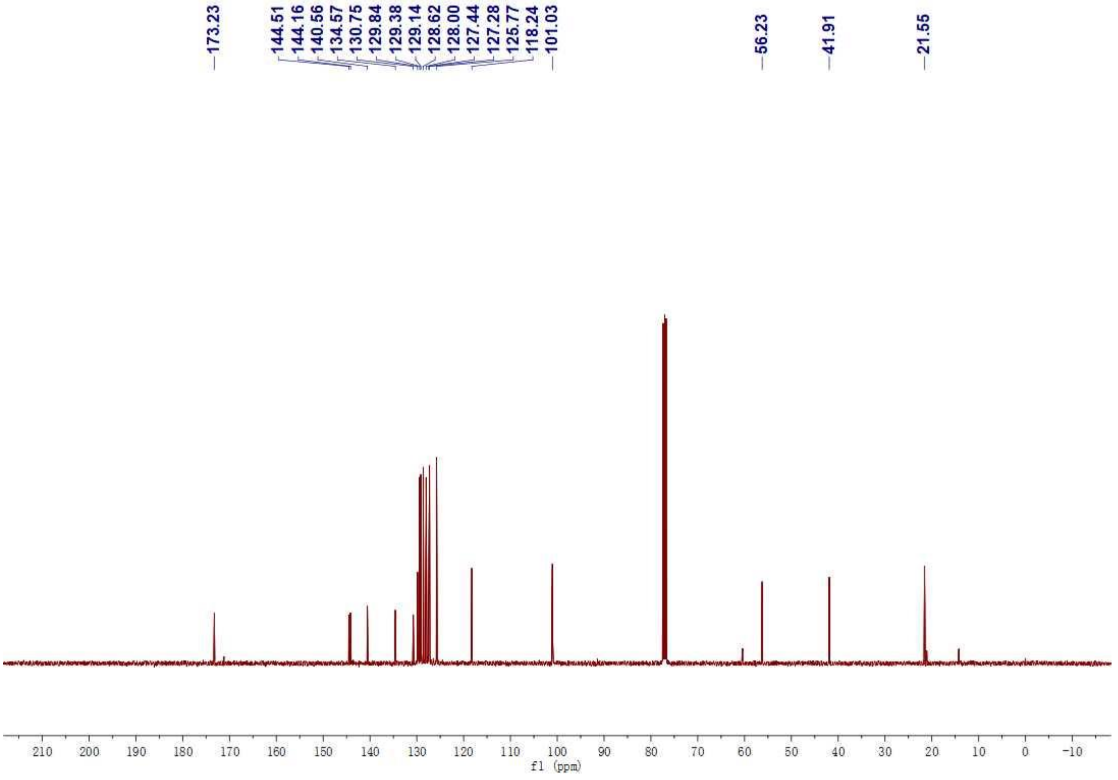

Supplementary Figure 50. ^{13}C NMR (101 MHz, $CDCl_3$) of **3a**

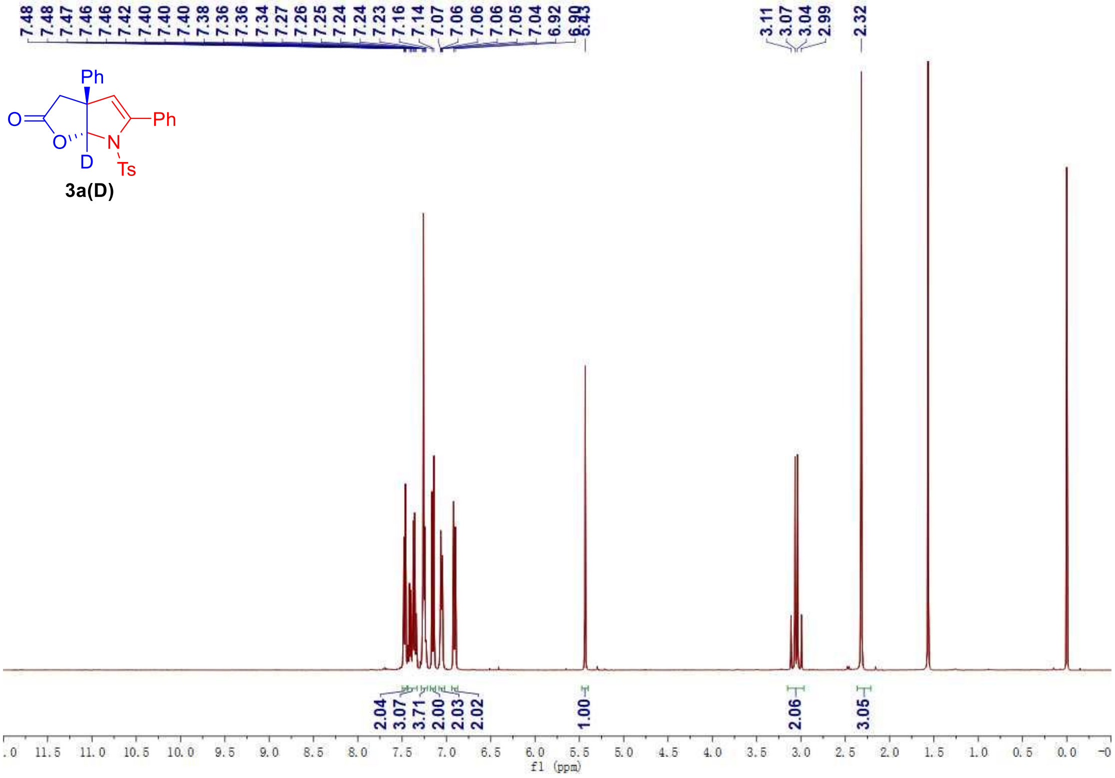

Supplementary Figure 51. ^{1}H NMR (400 MHz, $CDCl_3$) of **3a(D)**

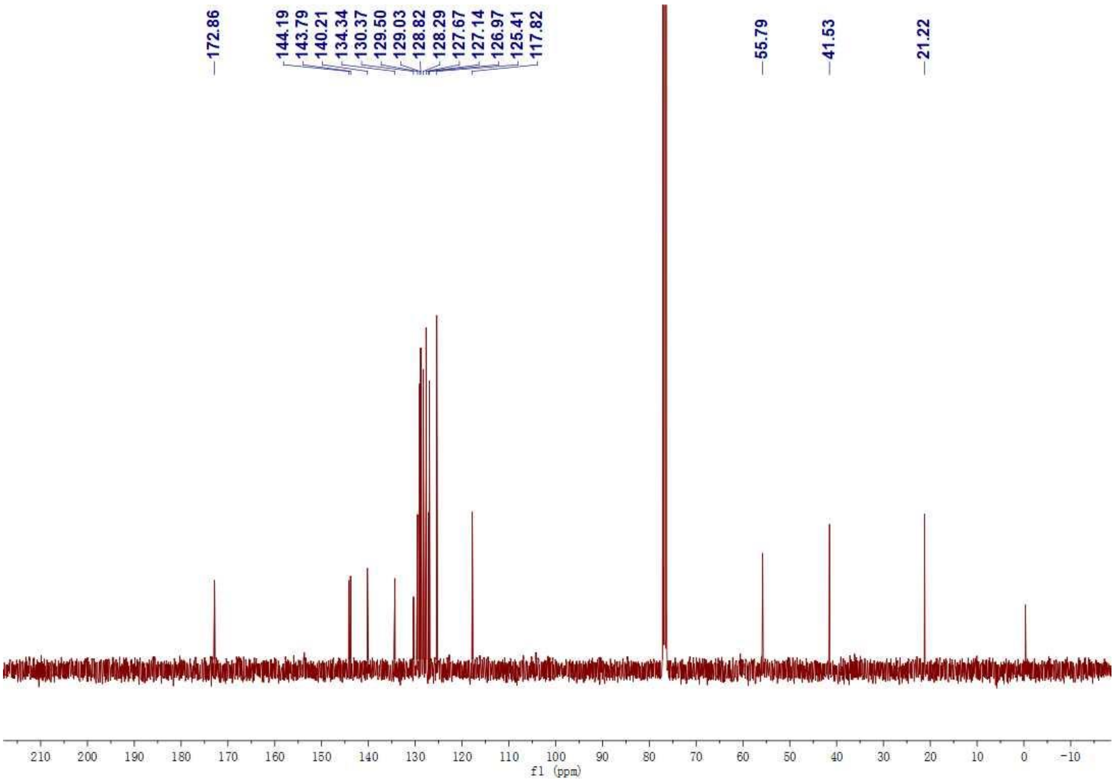

Supplementary Figure 52. ^{13}C NMR (101 MHz, $CDCl_3$) of **3a(D)**

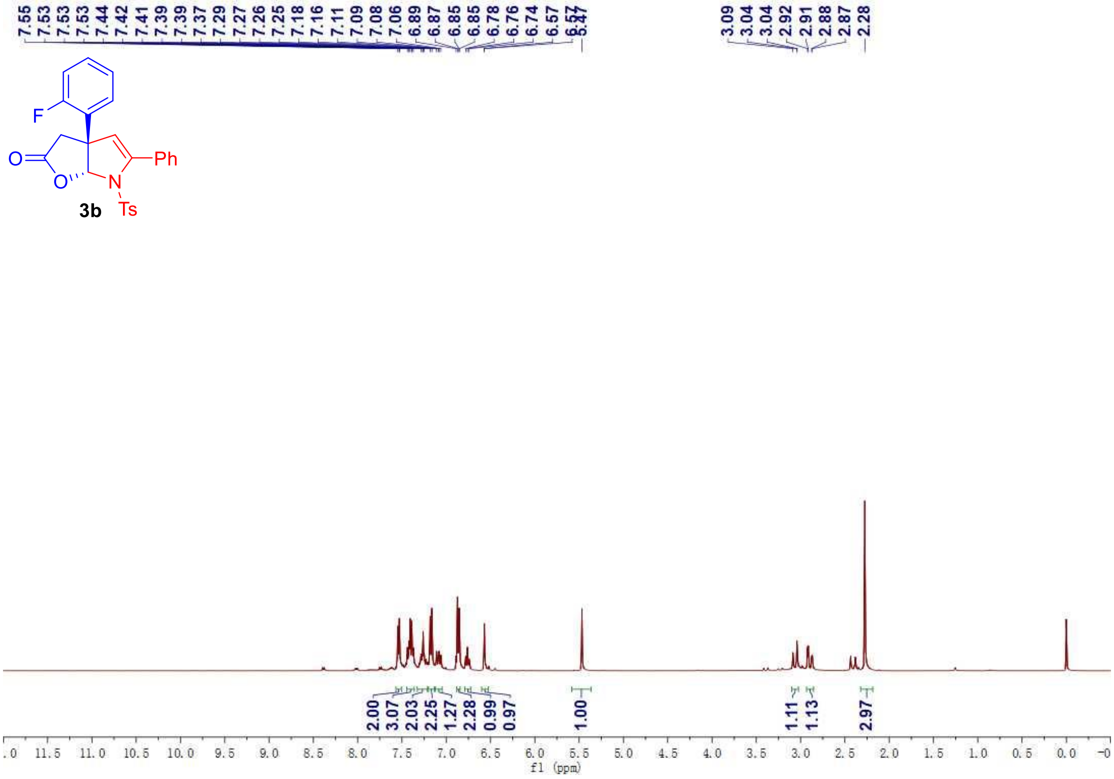

Supplementary Figure 53. ^{1}H NMR (400 MHz, $CDCl_3$) of **3b**

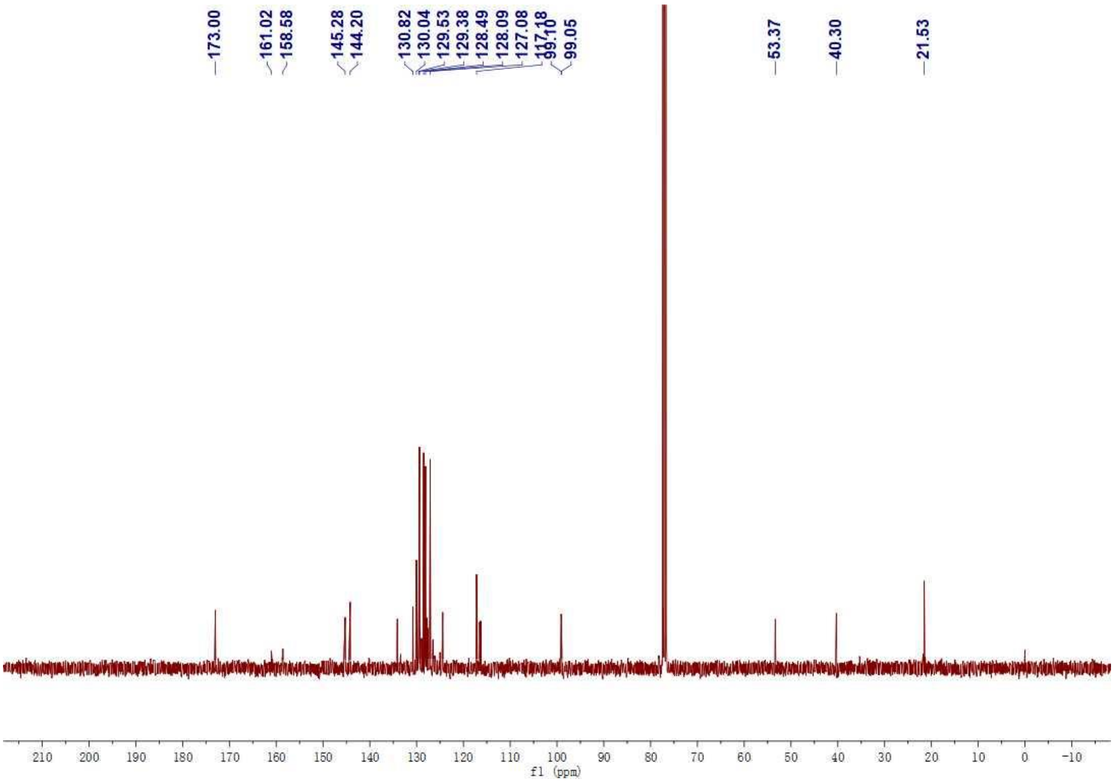

Supplementary Figure 54. ^{13}C NMR (101 MHz, $CDCl_3$) of **3b**

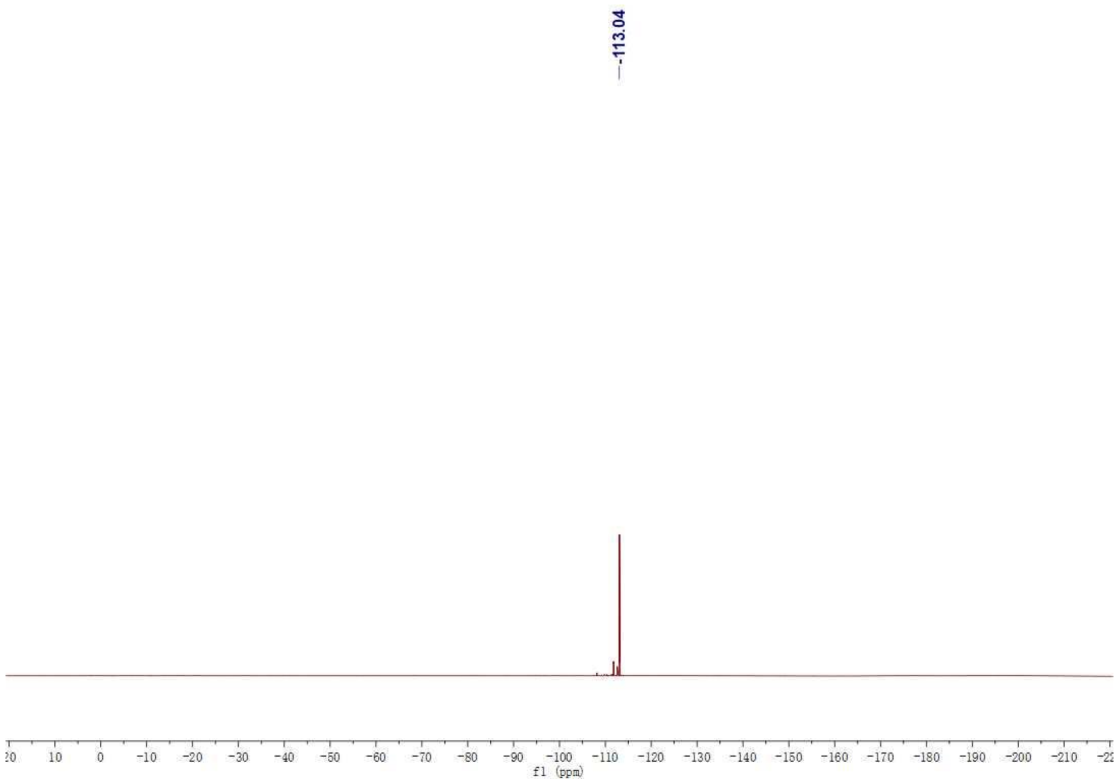

Supplementary Figure 55. ^{19}F NMR (376 MHz, $CDCl_3$) of **3b**

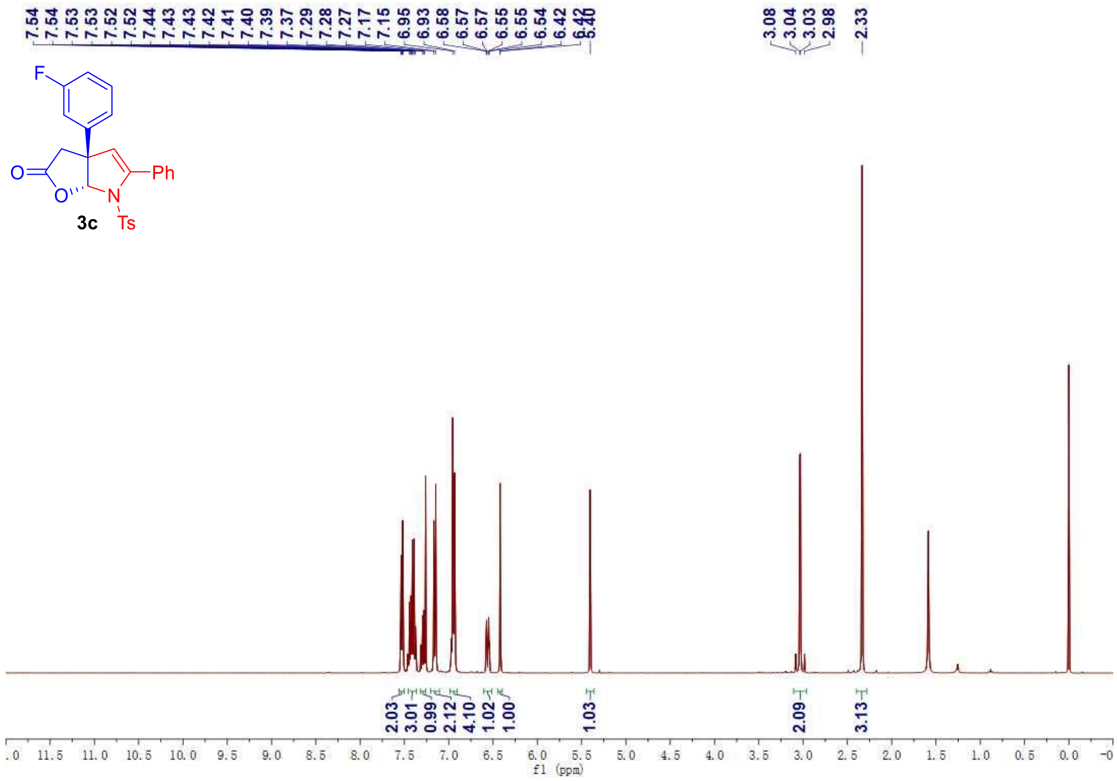

Supplementary Figure 56. ^{1}H NMR (400 MHz, $CDCl_3$) of **3c**

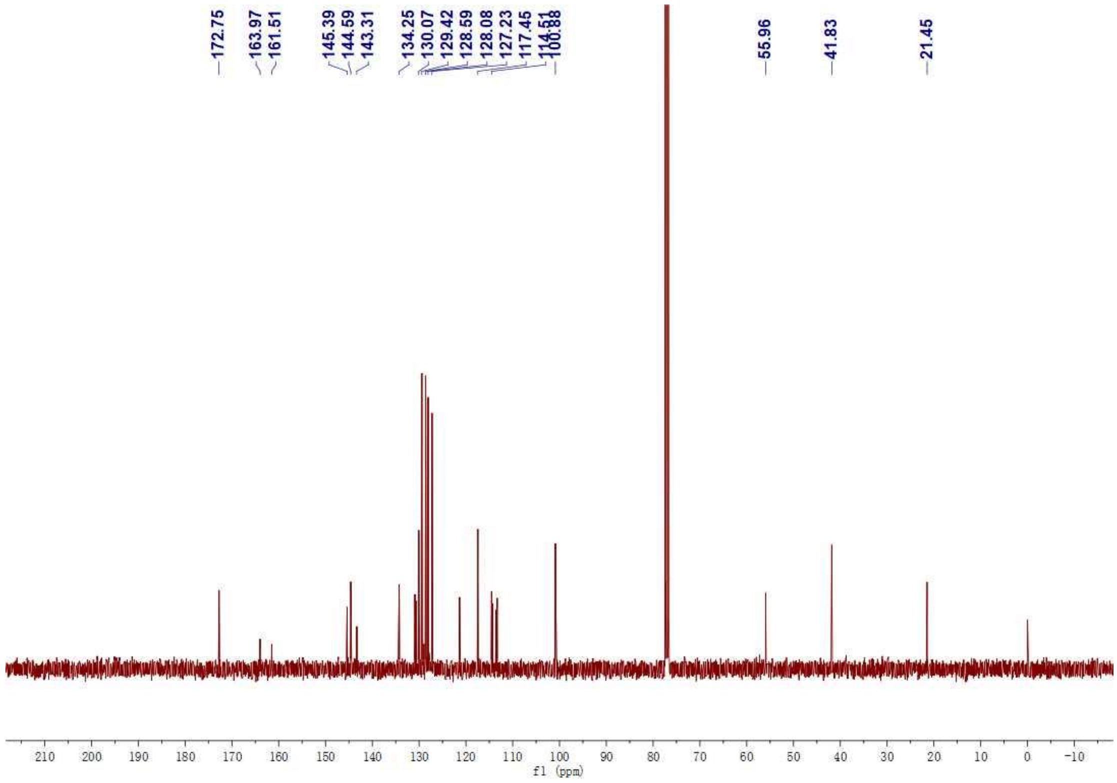

Supplementary Figure 57. ^{13}C NMR (101 MHz, $CDCl_3$) of **3c**

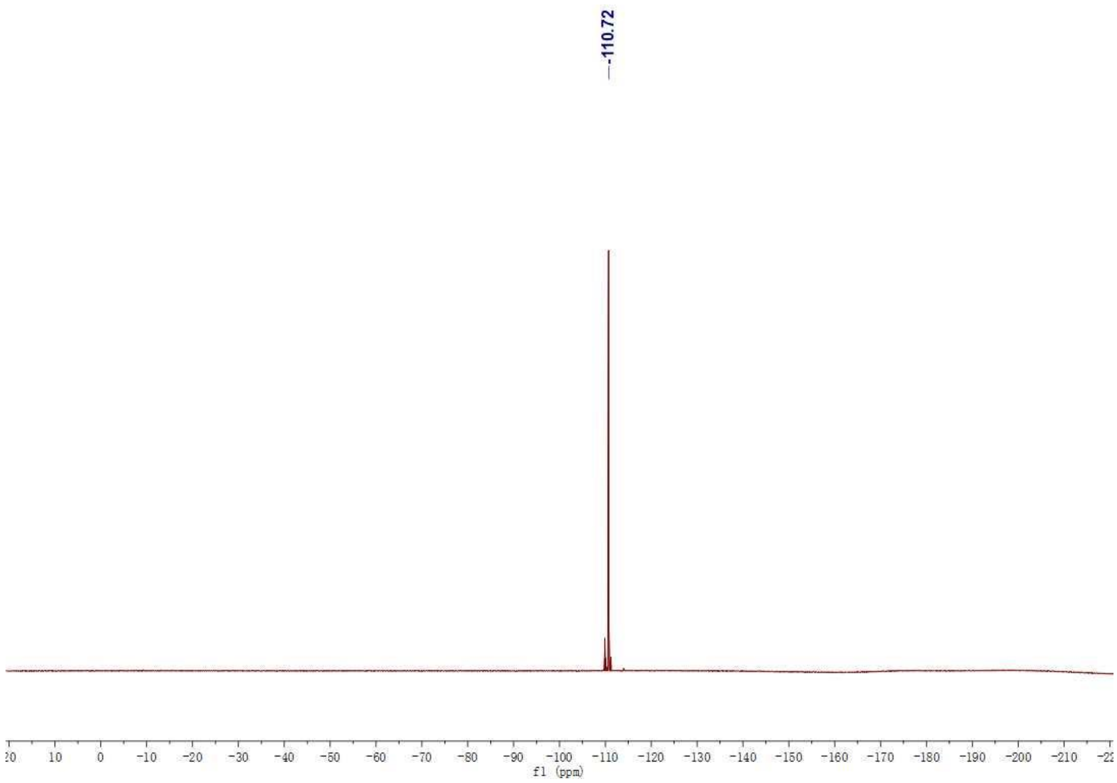

Supplementary Figure 58. ^{19}F NMR (376 MHz, $CDCl_3$) of **3c**

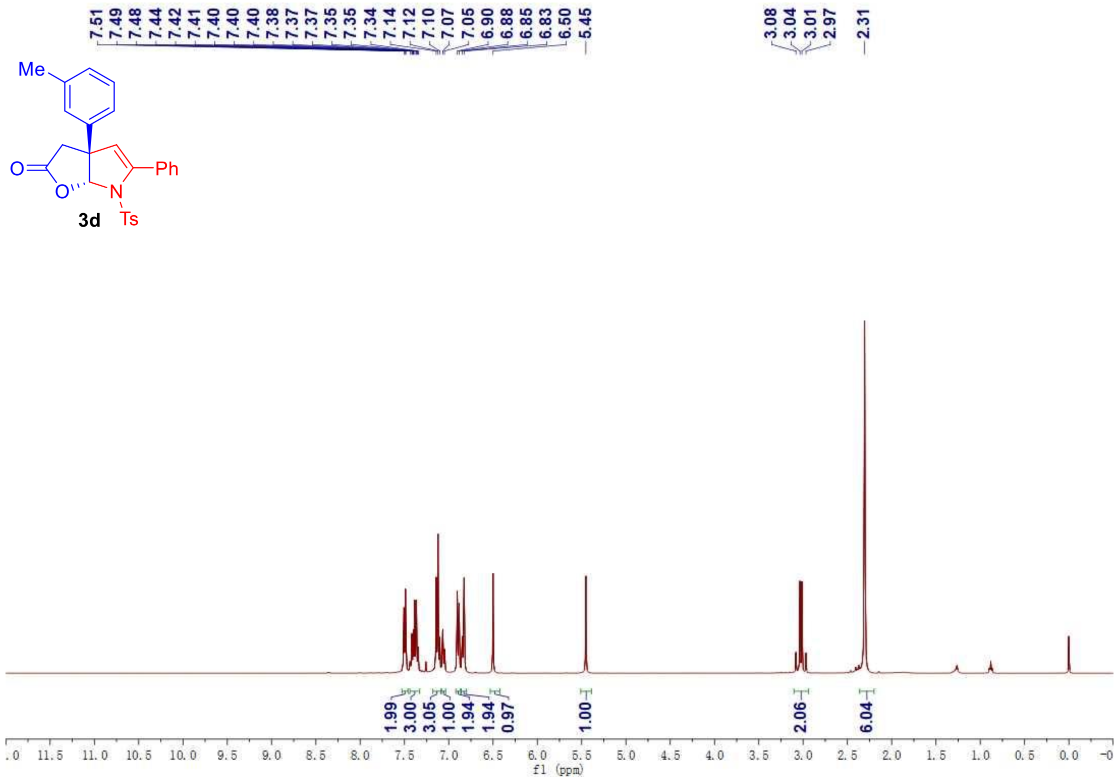

Supplementary Figure 59. ^{1}H NMR (400 MHz, $CDCl_3$) of **3d**

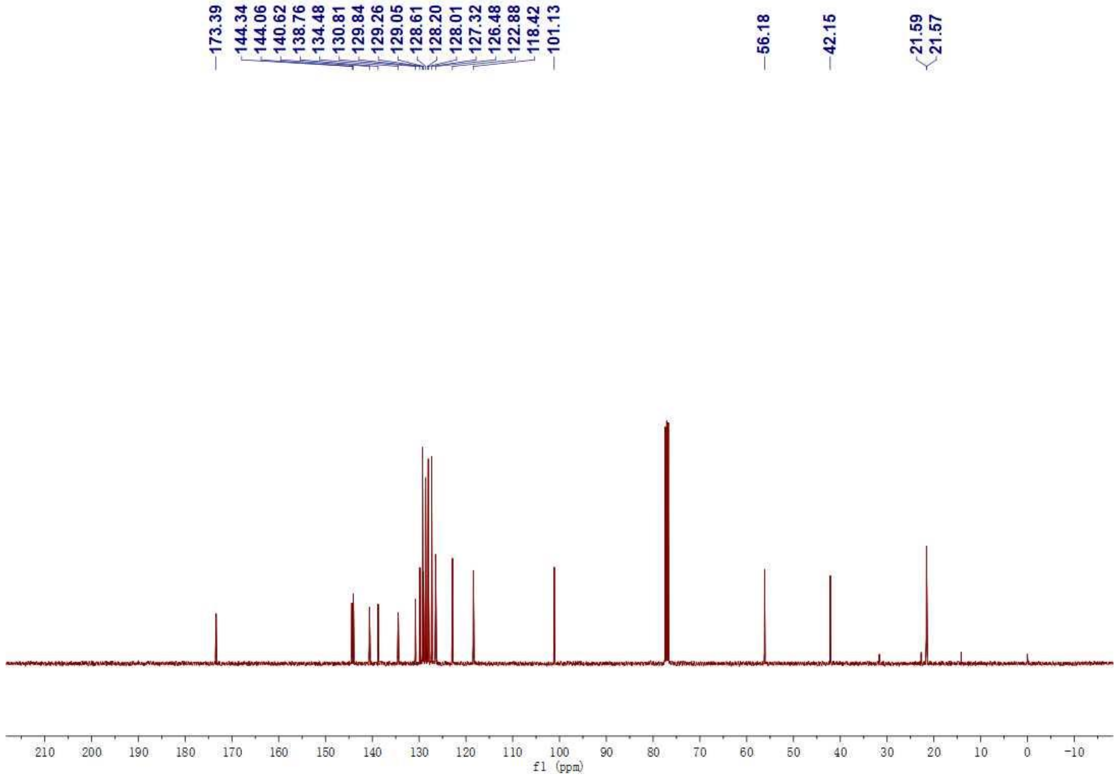

Supplementary Figure 60. ^{13}C NMR (101 MHz, $CDCl_3$) of **3d**

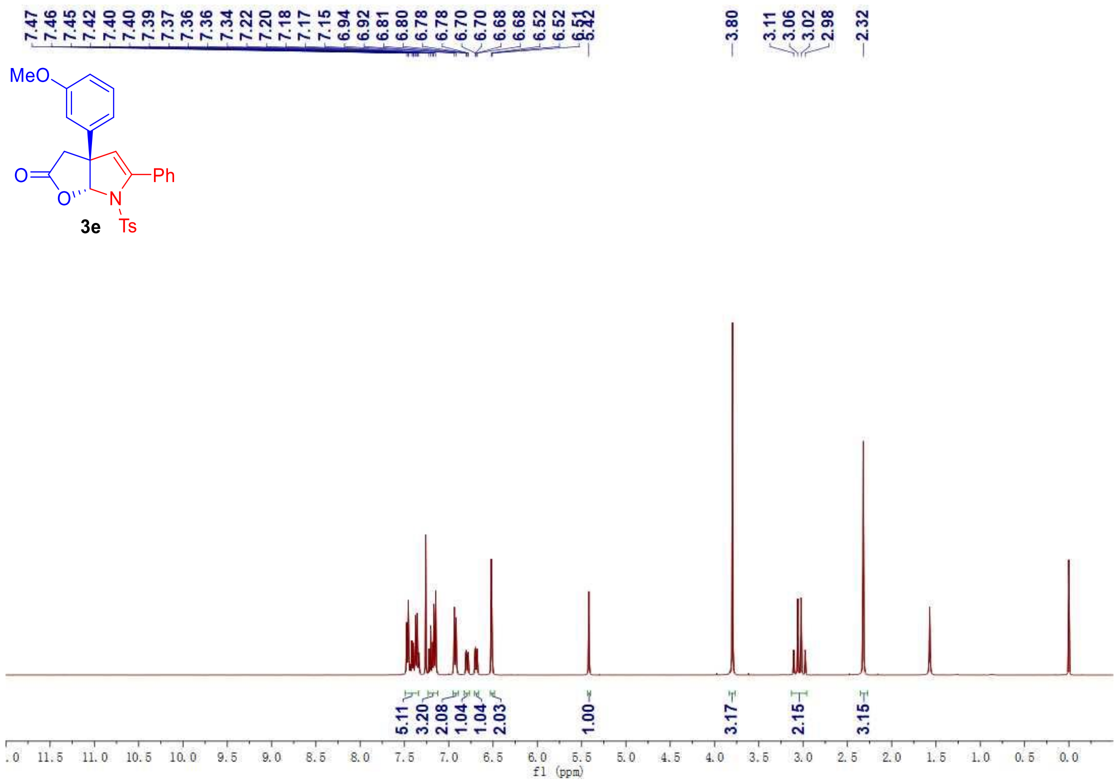

Supplementary Figure 61. ^{1}H NMR (400 MHz, $CDCl_3$) of **3e**

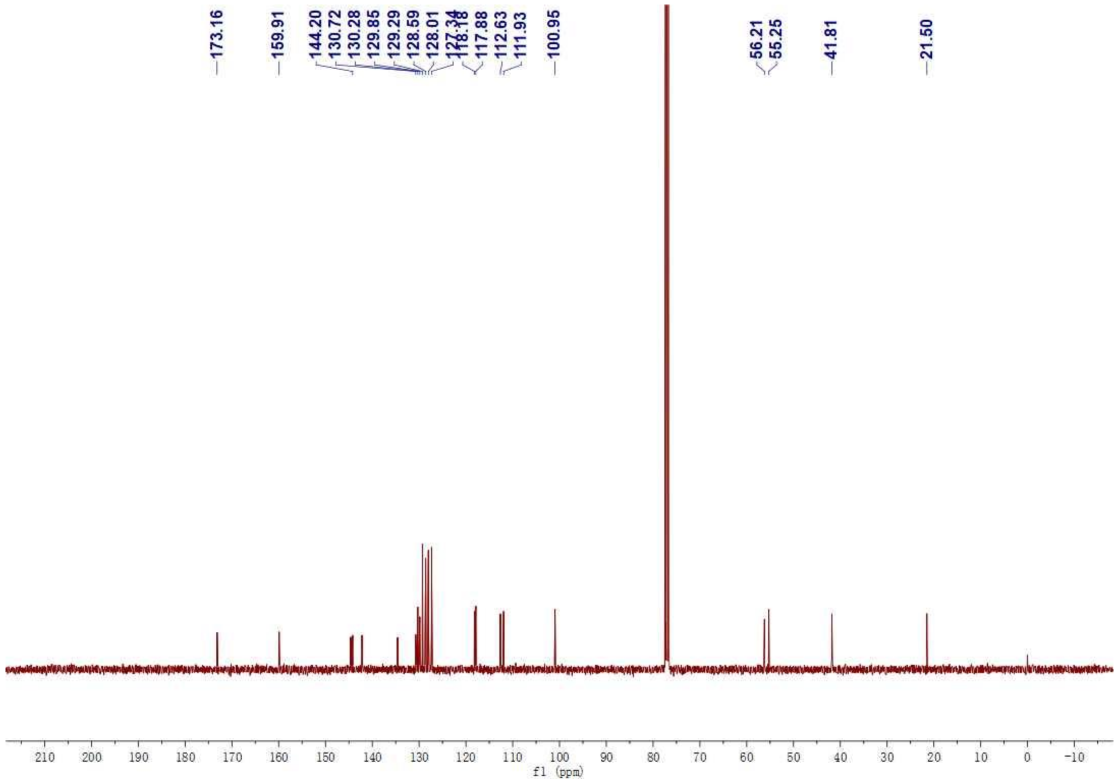

Supplementary Figure 62. ^{13}C NMR (101 MHz, $CDCl_3$) of **3e**

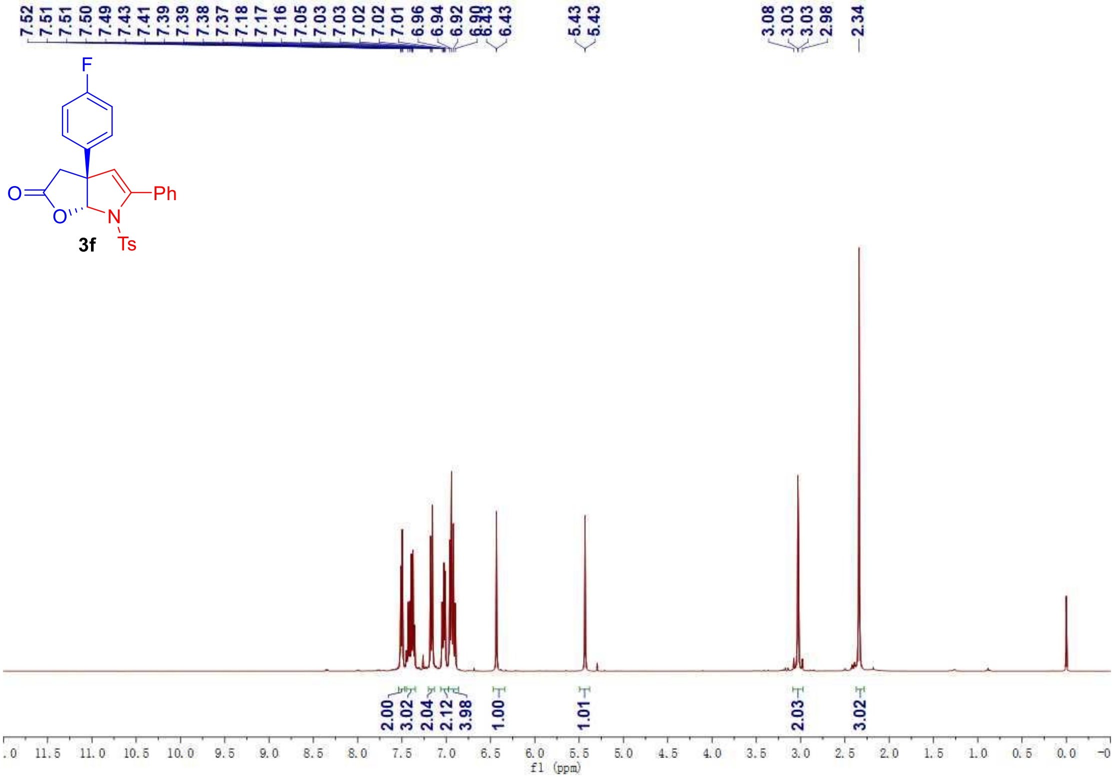

Supplementary Figure 63. ^{1}H NMR (400 MHz, $CDCl_3$) of **3f**

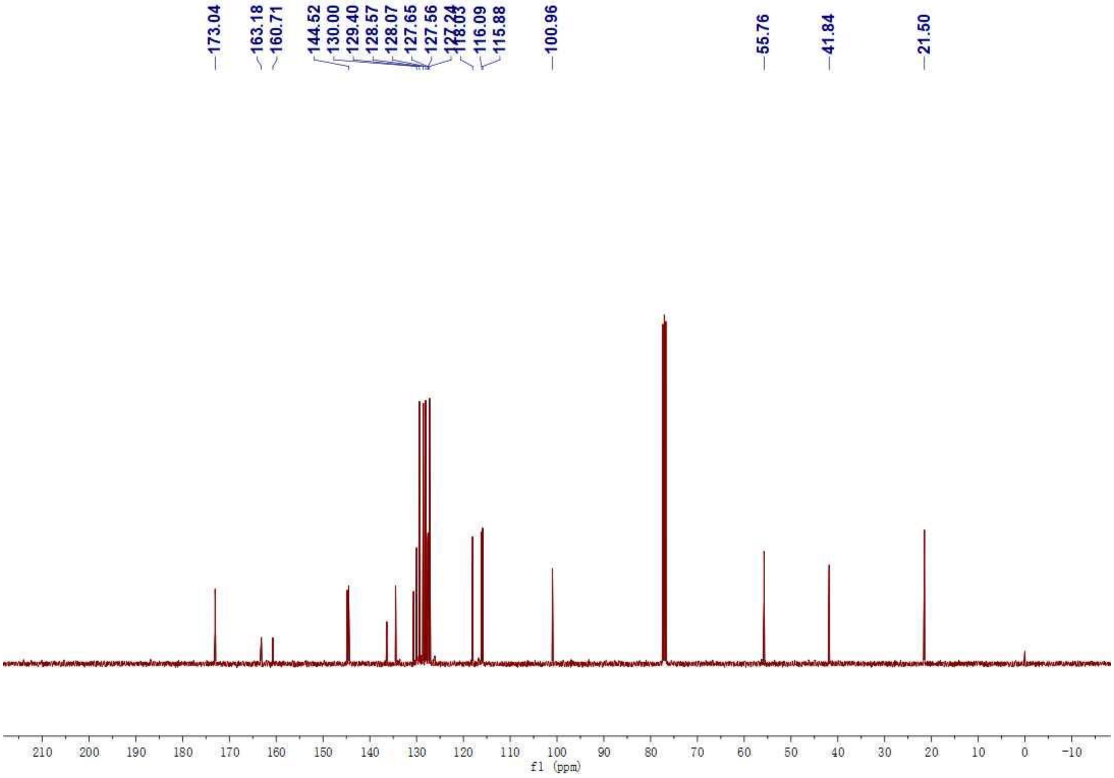

Supplementary Figure 64. ^{13}C NMR (101 MHz, $CDCl_3$) of **3f**

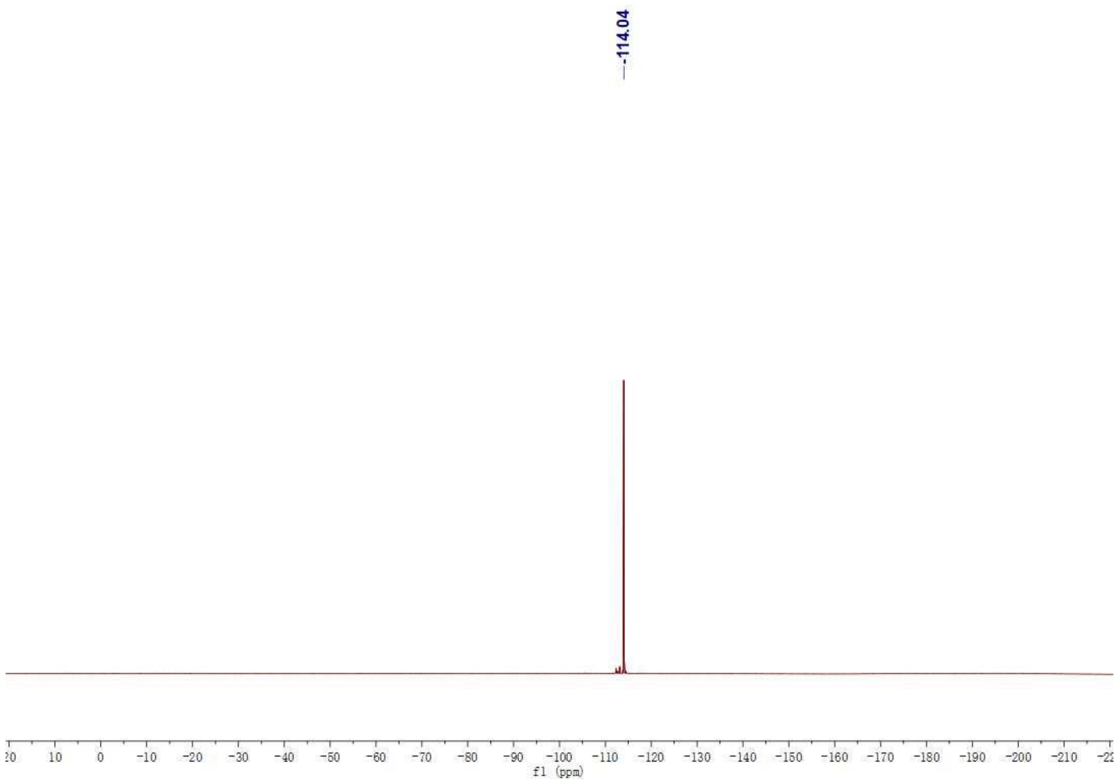

Supplementary Figure 65. ^{19}F NMR (376 MHz, $CDCl_3$) of **3f**

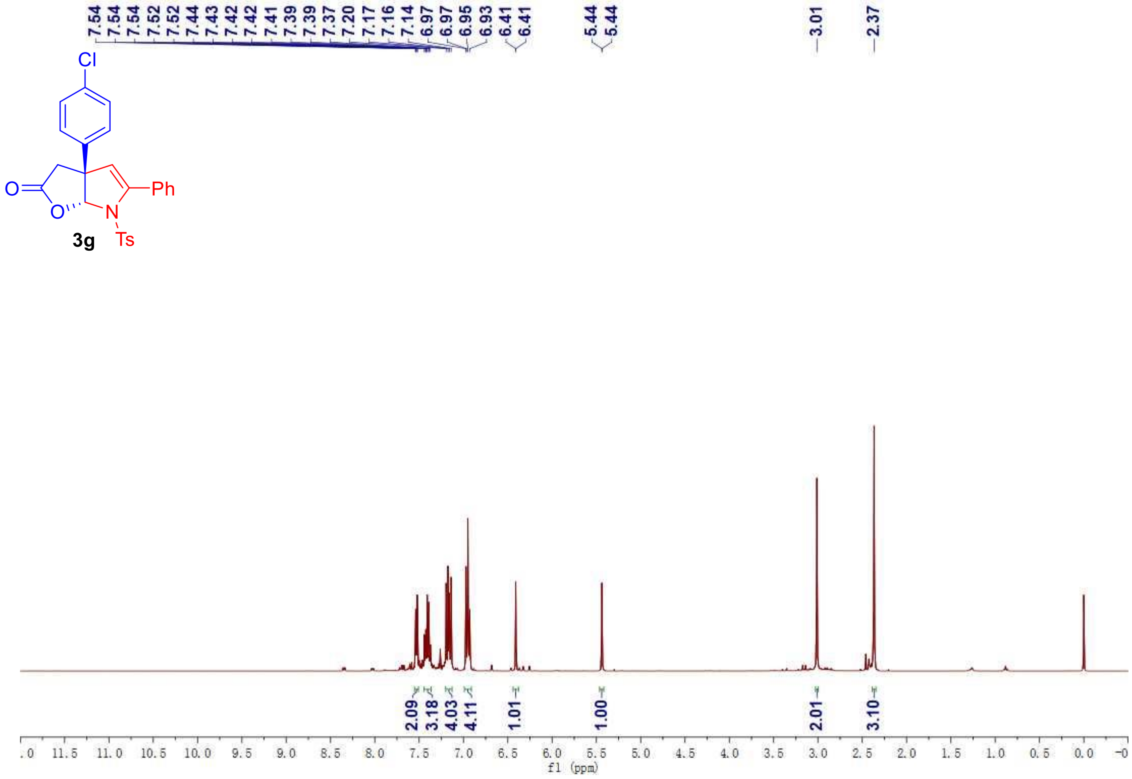

Supplementary Figure 66. ^{1}H NMR (400 MHz, $CDCl_3$) of **3g**

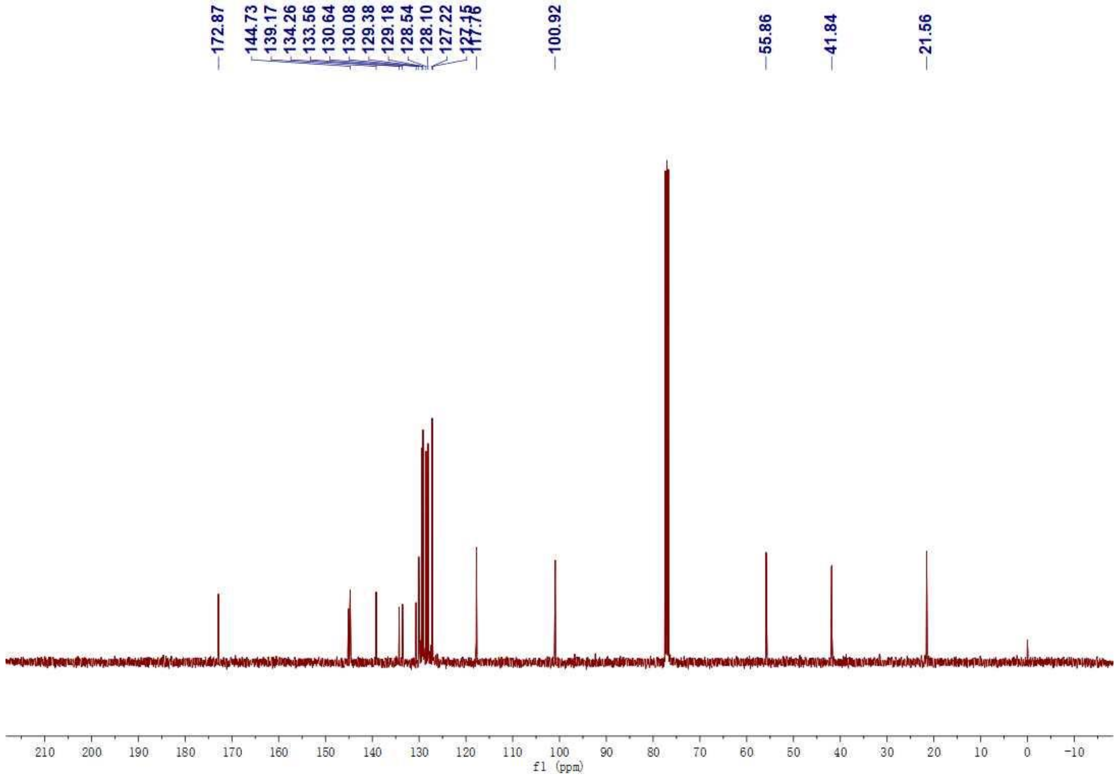

Supplementary Figure 67. ^{13}C NMR (101 MHz, $CDCl_3$) of **3g**

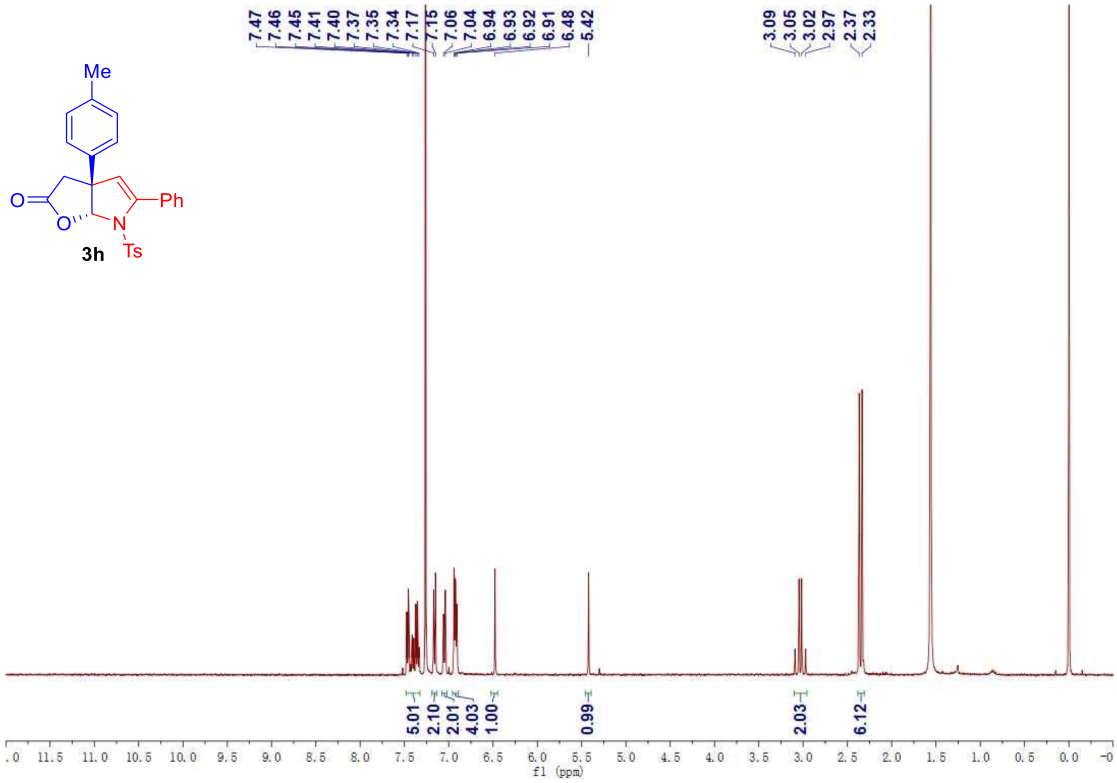

Supplementary Figure 68. [13]H NMR (400 MHz, $CDCl_3$) of **3h**

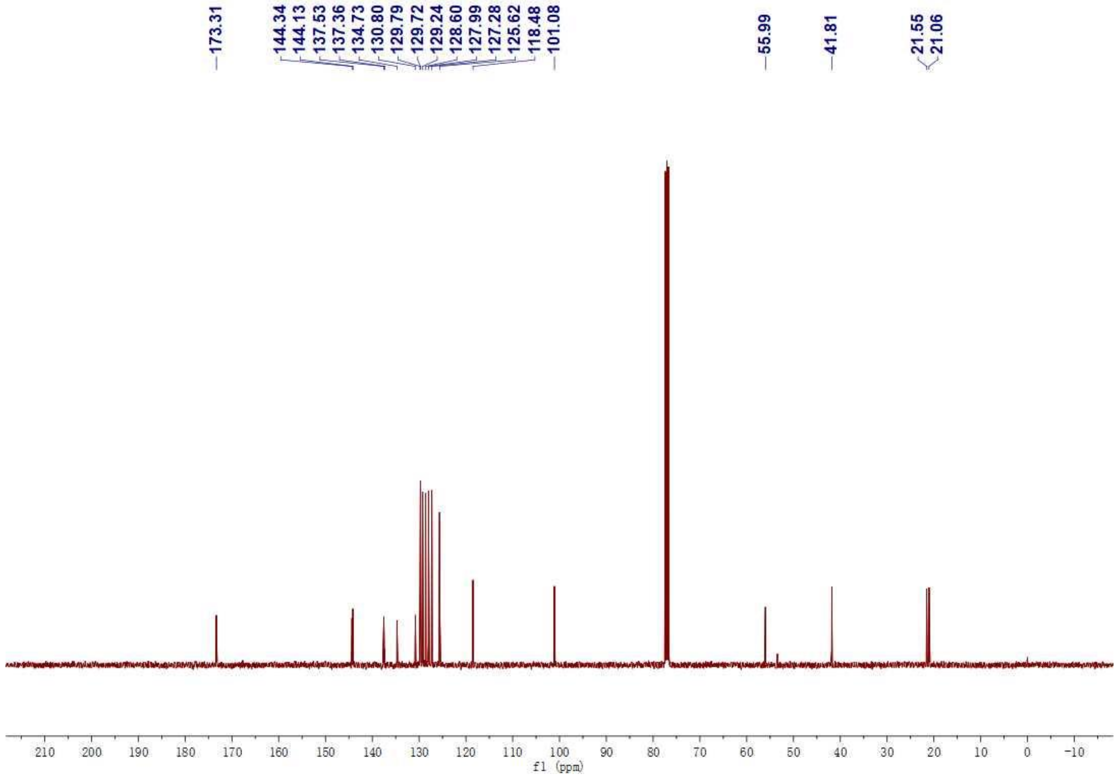

Supplementary Figure 69. ^{13}C NMR (101 MHz, $CDCl_3$) of **3h**

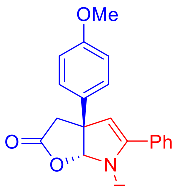

3i

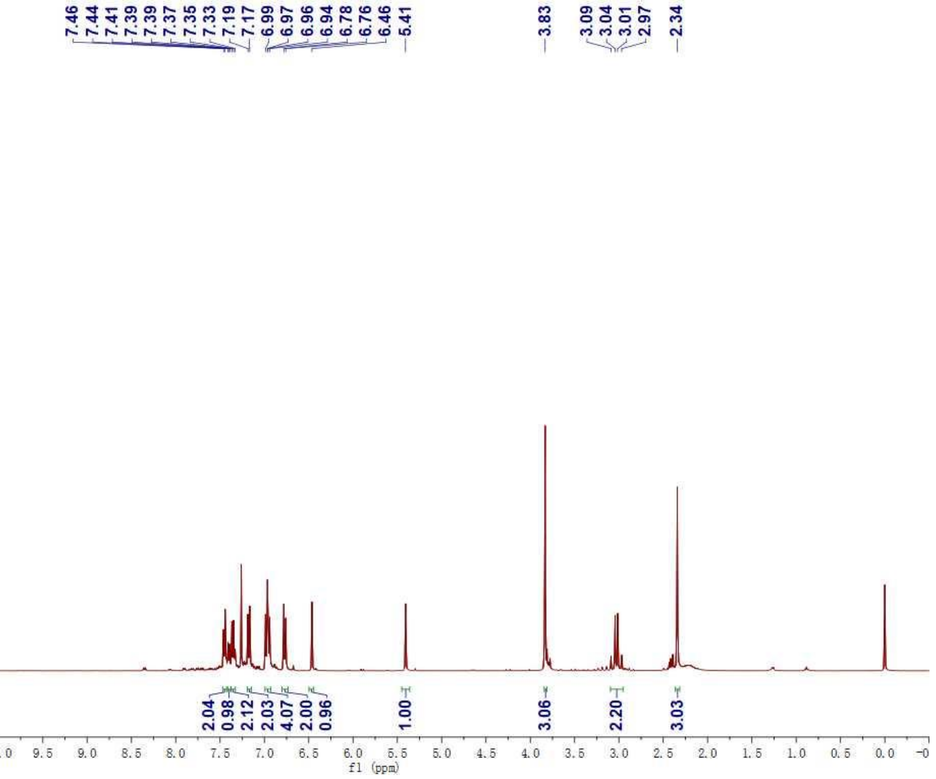

Supplementary Figure 70. ^{1}H NMR (400 MHz, $CDCl_3$) of **3i**

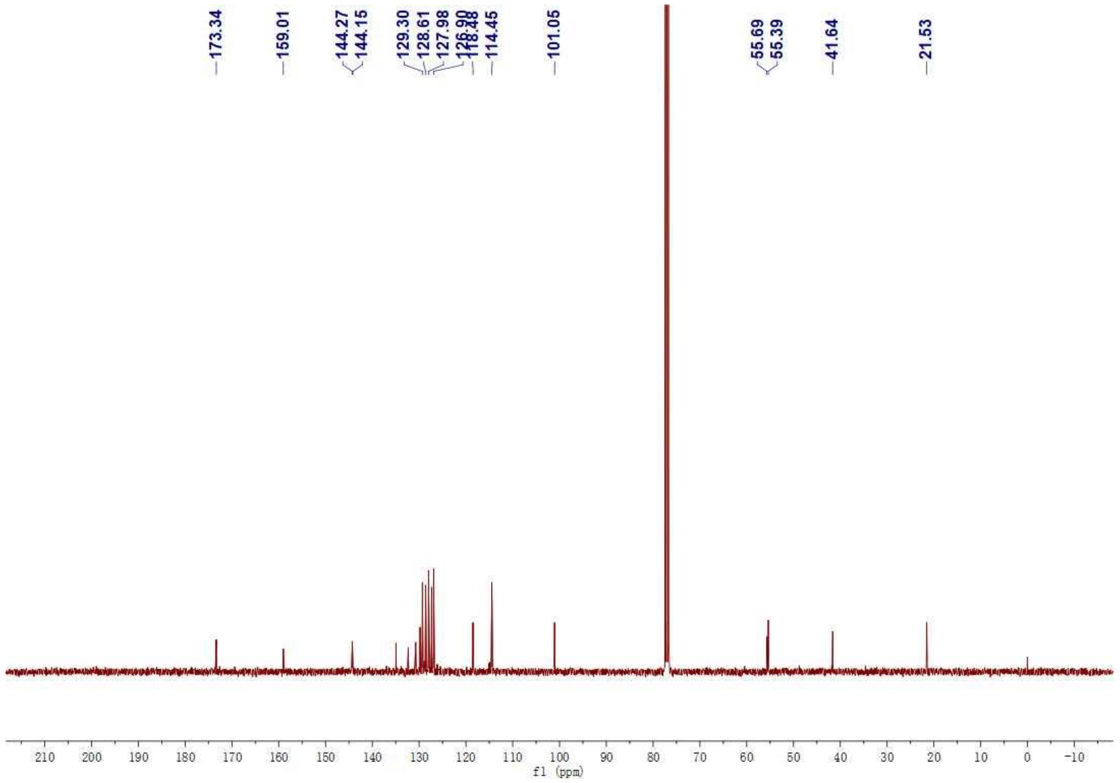

Supplementary Figure 71. ^{13}C NMR (101 MHz, $CDCl_3$) of **3i**

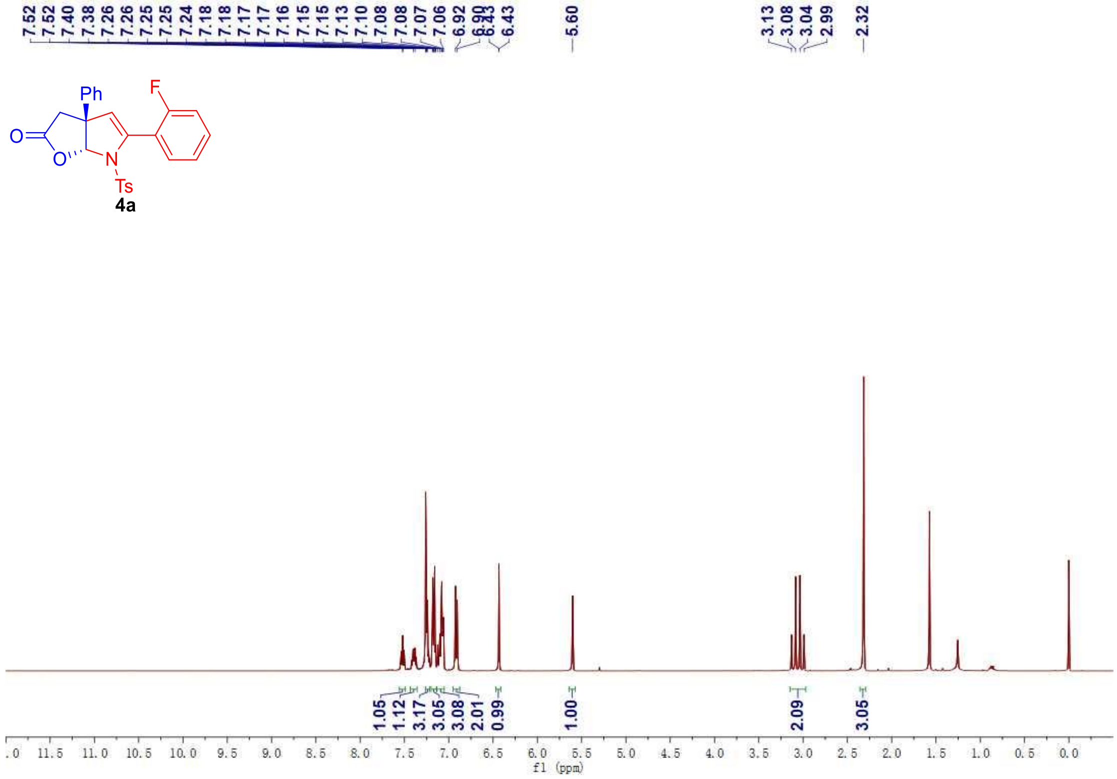

Supplementary Figure 72. ^{1}H NMR (400 MHz, $CDCl_3$) of **4a**

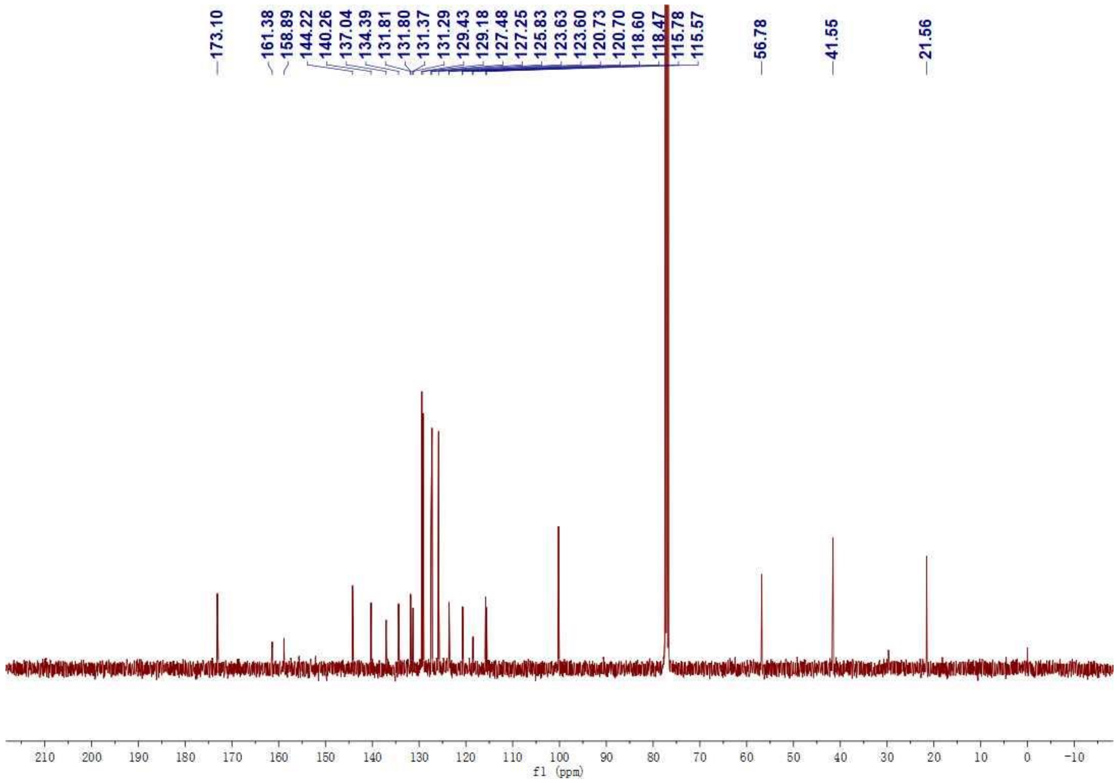

Supplementary Figure 73. ^{13}C NMR (101 MHz, $CDCl_3$) of **4a**

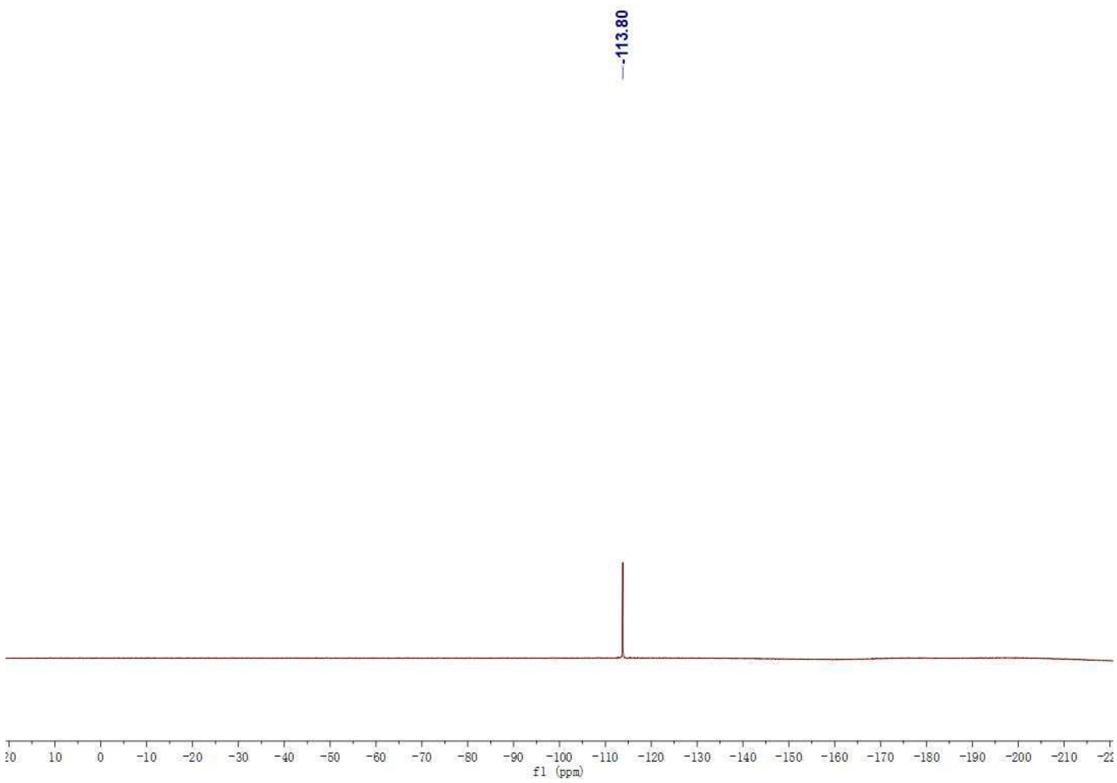

Supplementary Figure 74. ^{19}F NMR (376 MHz, $CDCl_3$) of **4a**

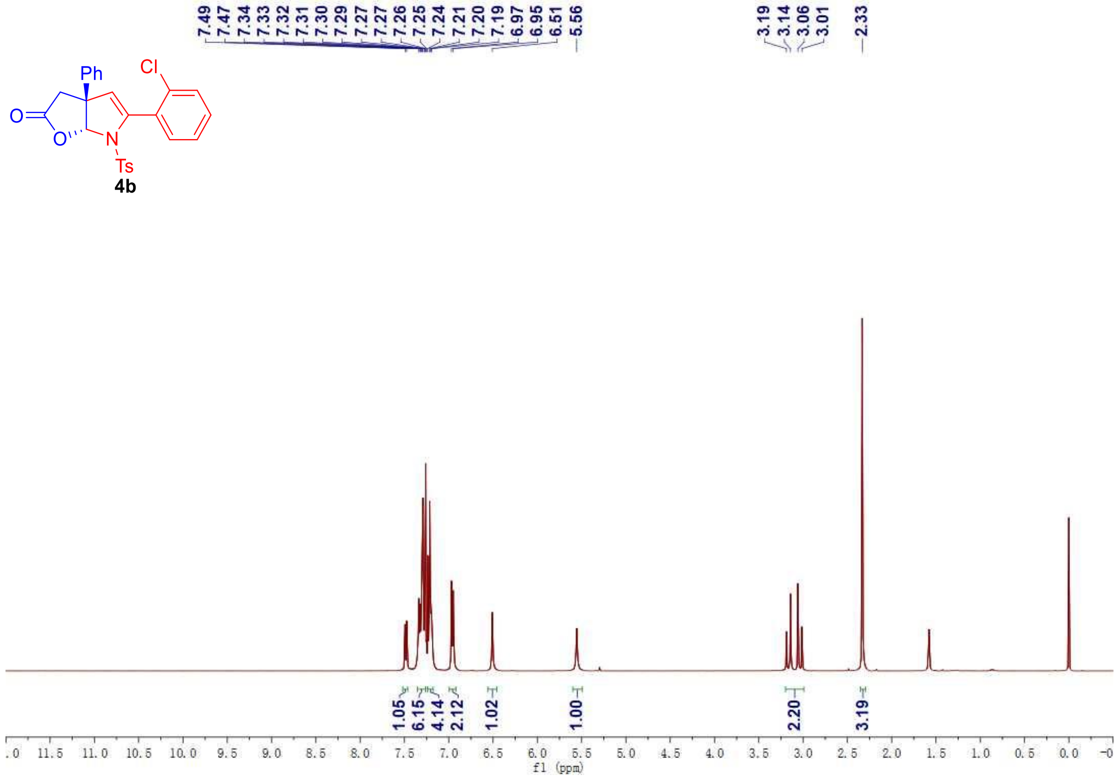

Supplementary Figure 75. ^{1}H NMR (400 MHz, $CDCl_3$) of **4b**

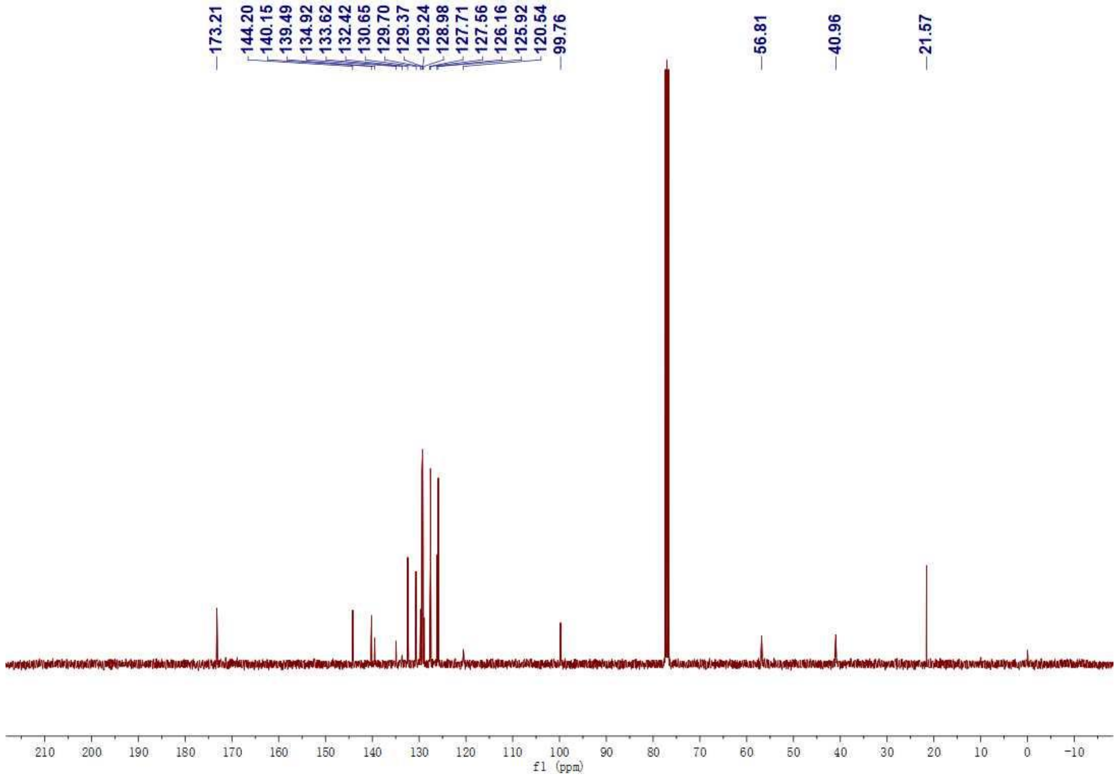

Supplementary Figure 76. ^{13}C NMR (101 MHz, $CDCl_3$) of **4b**

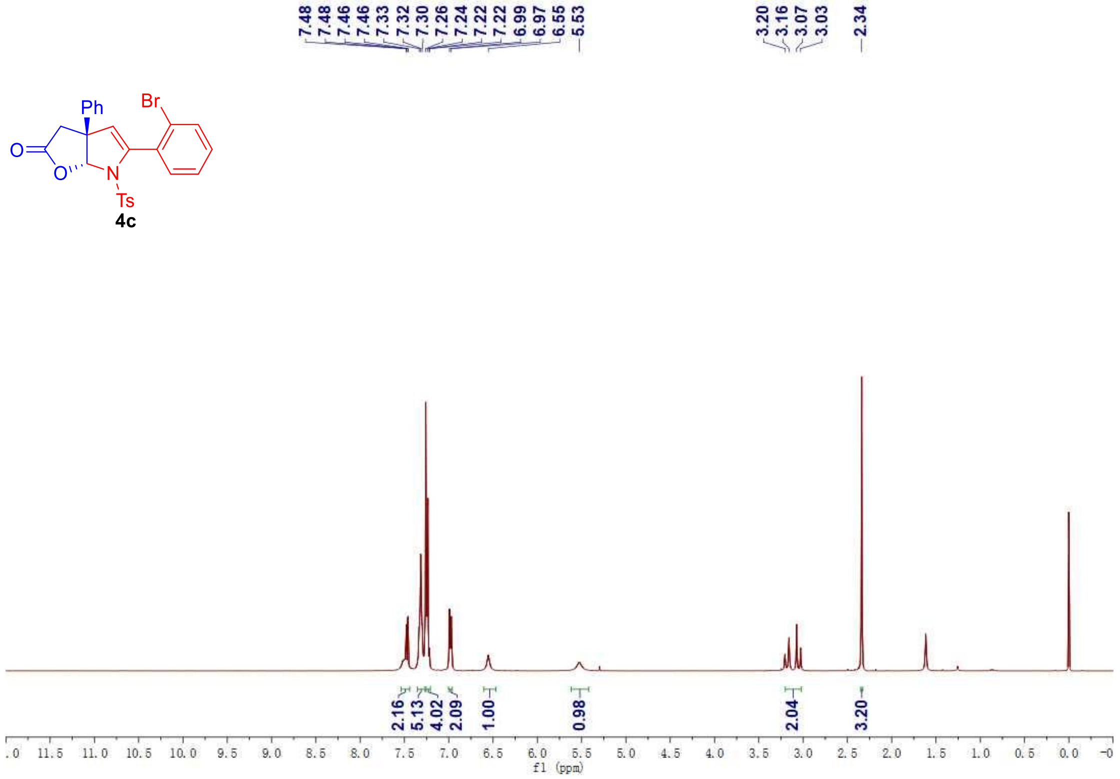

Supplementary Figure 77. ^{1}H NMR (400 MHz, $CDCl_3$) of **4c**

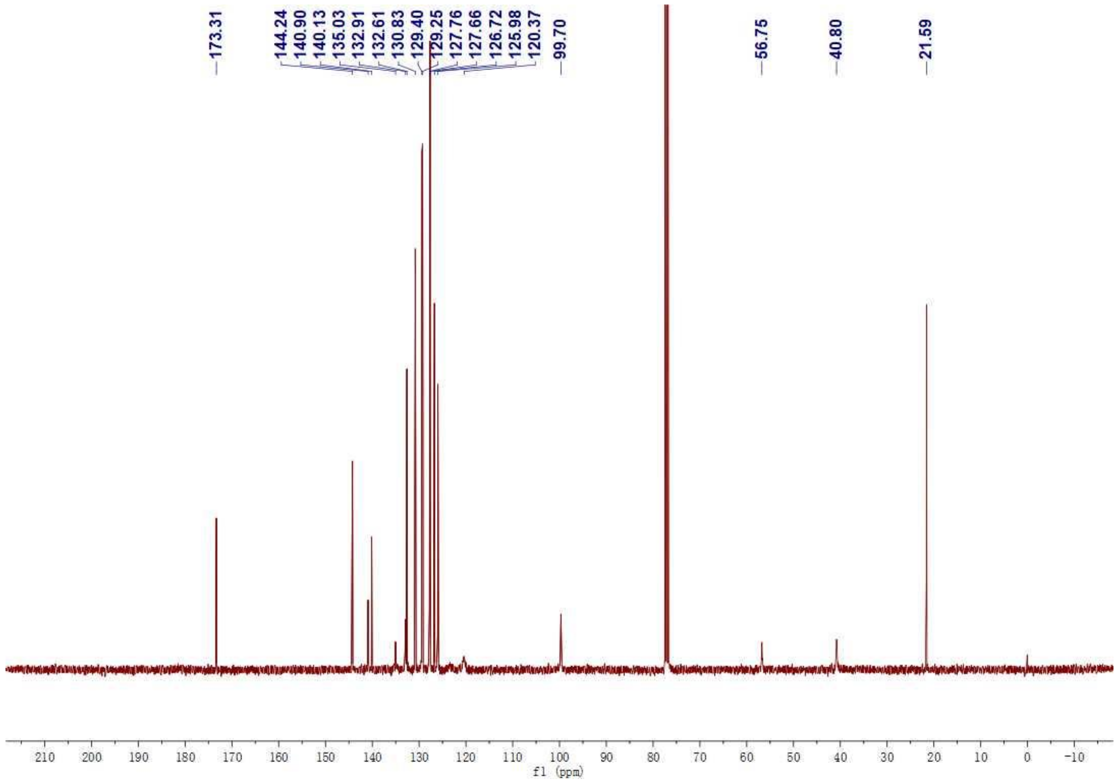

Supplementary Figure 78. ^{13}C NMR (101 MHz, $CDCl_3$) of **4c**

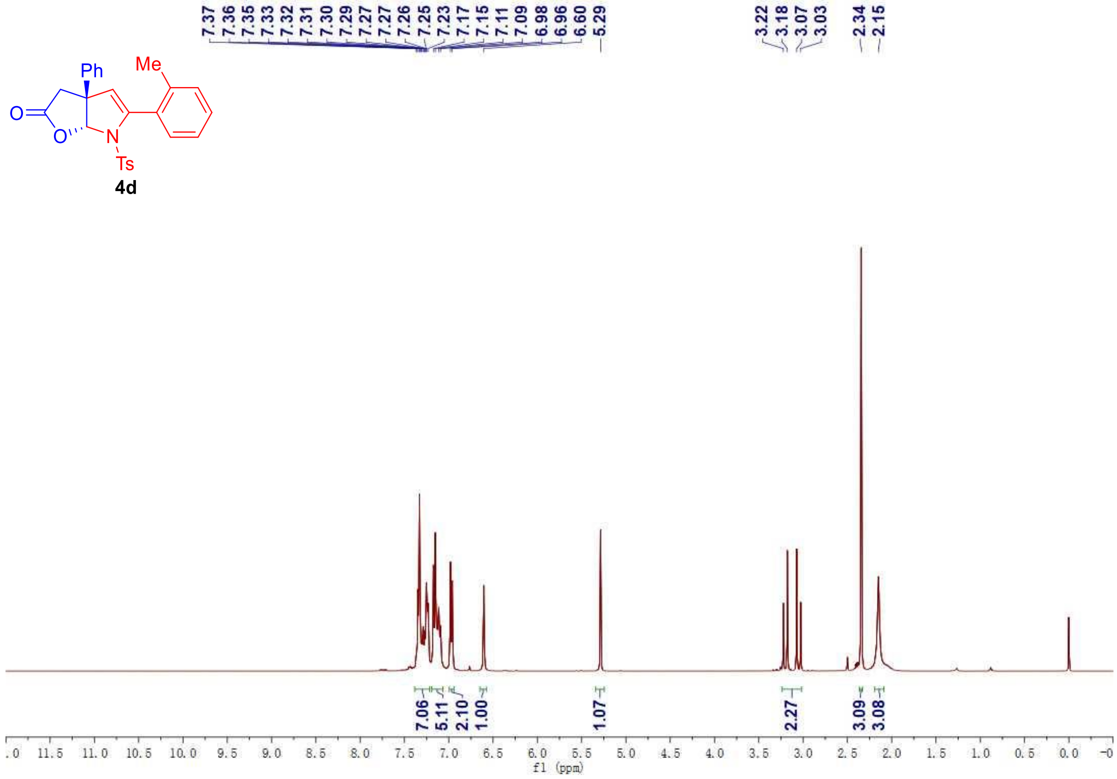

Supplementary Figure 79. ^{1}H NMR (400 MHz, $CDCl_3$) of **4d**

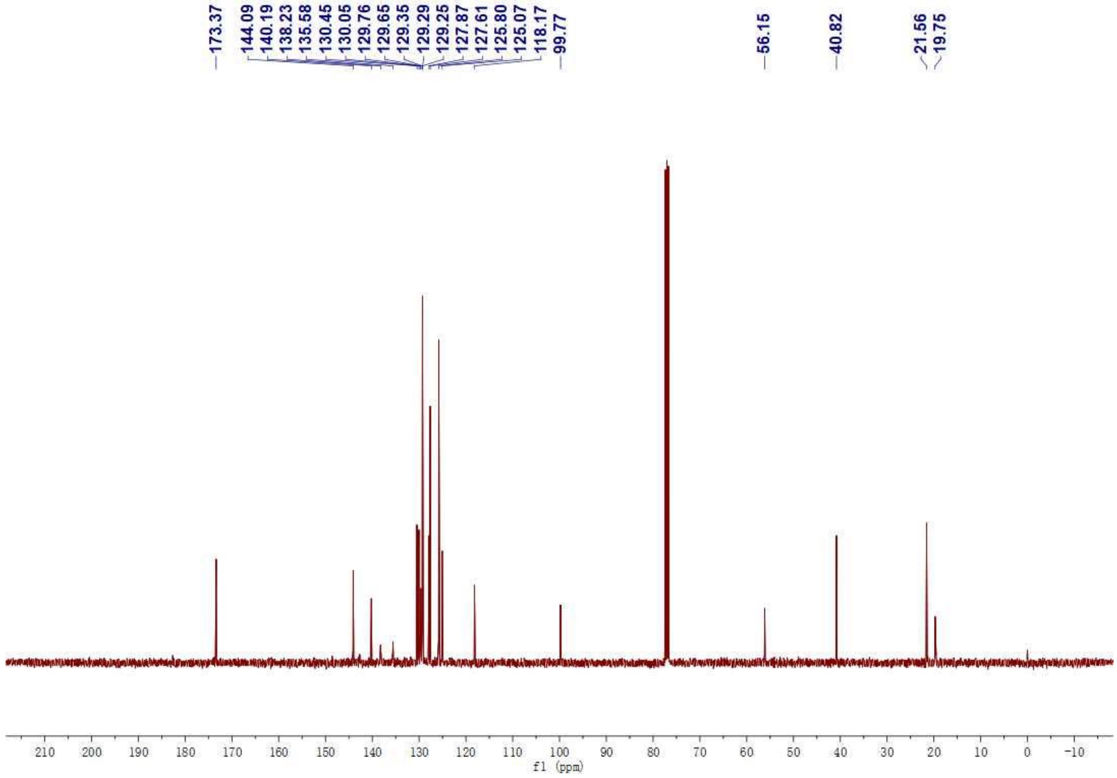

Supplementary Figure 80. ^{13}C NMR (101 MHz, $CDCl_3$) of **4d**

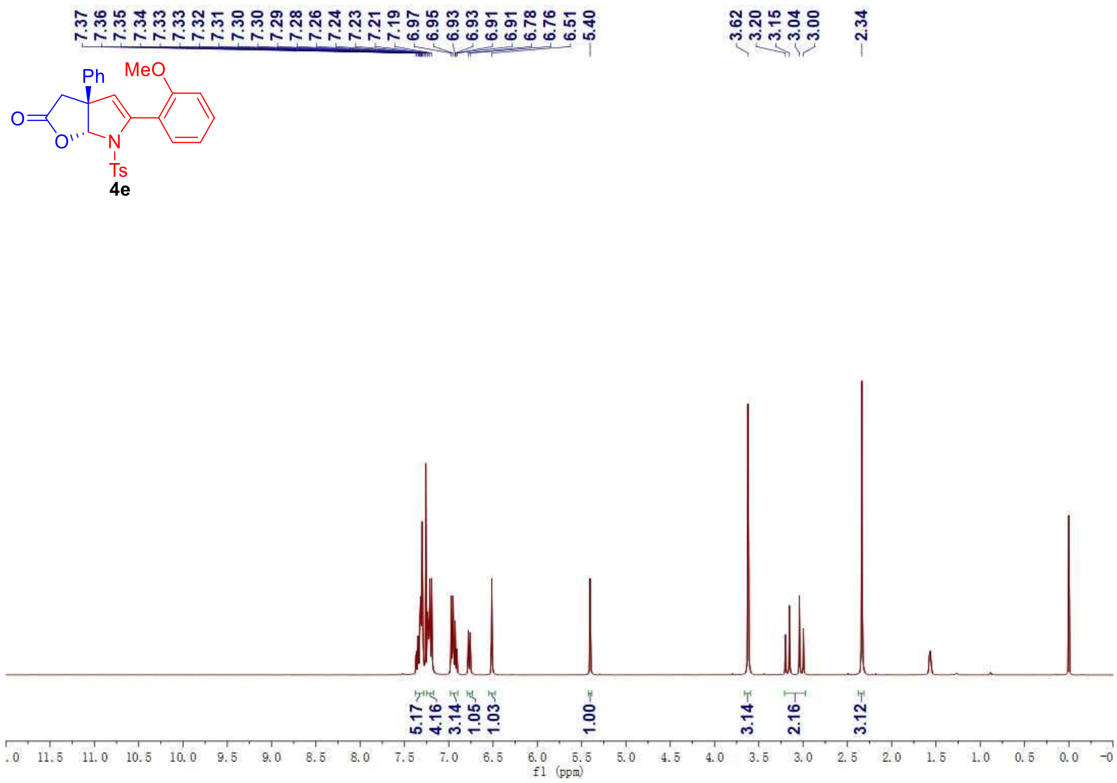

Supplementary Figure 81. ^{1}H NMR (400 MHz, $CDCl_3$) of **4e**

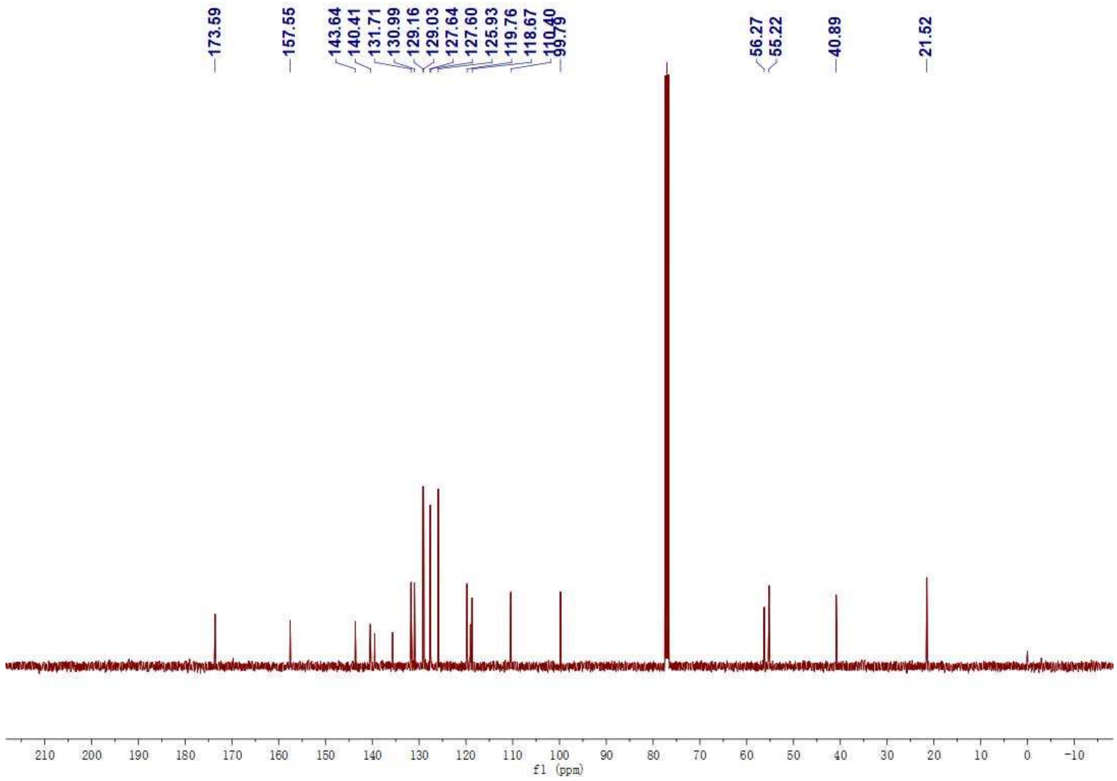

Supplementary Figure 82. ^{13}C NMR (101 MHz, $CDCl_3$) of **4e**

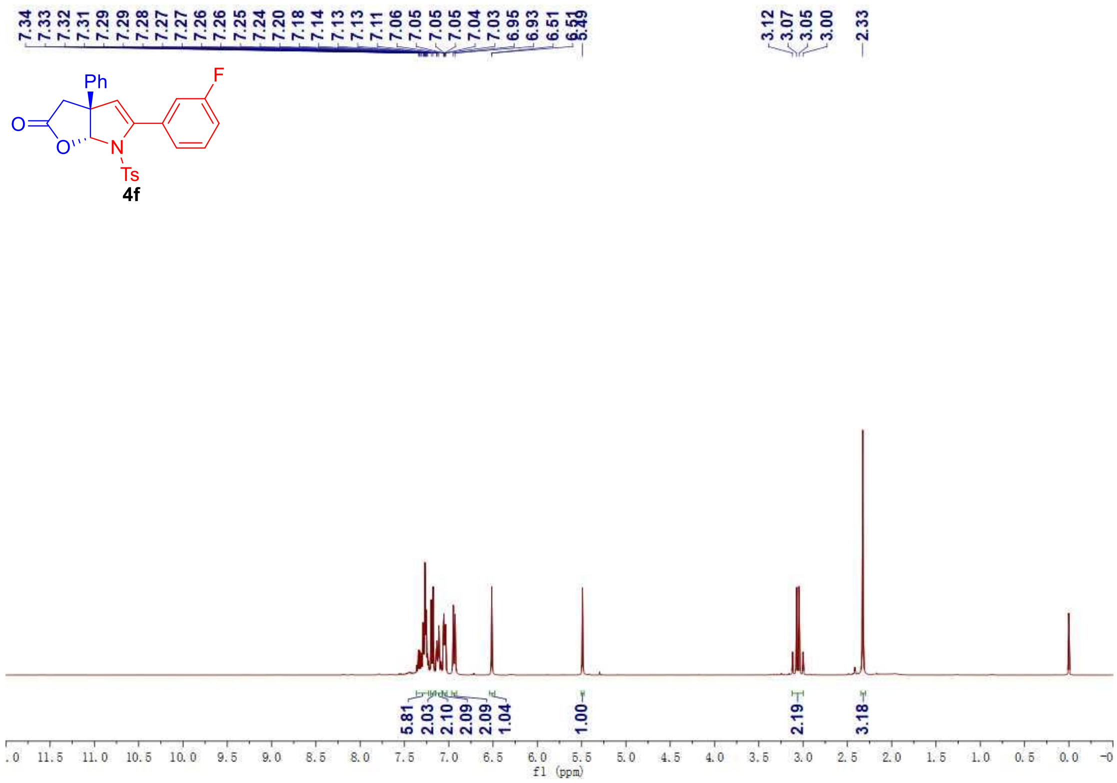

Supplementary Figure 83. ^{1}H NMR (400 MHz, $CDCl_3$) of **4f**

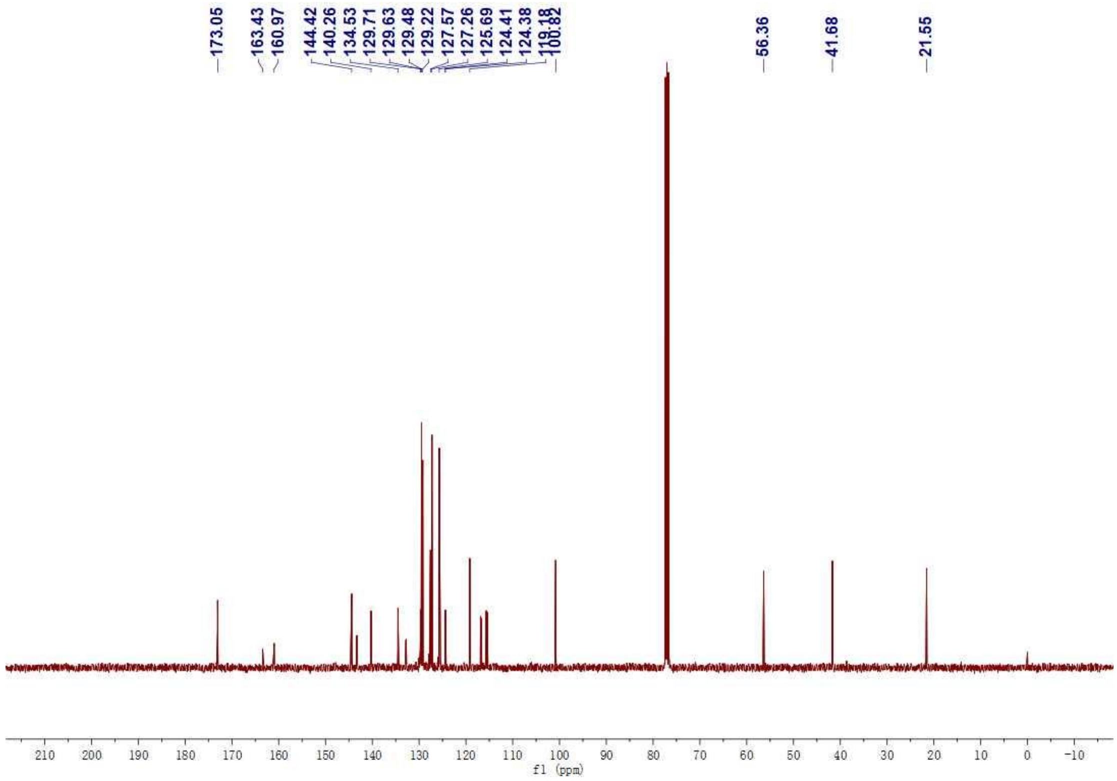

Supplementary Figure 84. ^{13}C NMR (101 MHz, $CDCl_3$) of **4f**

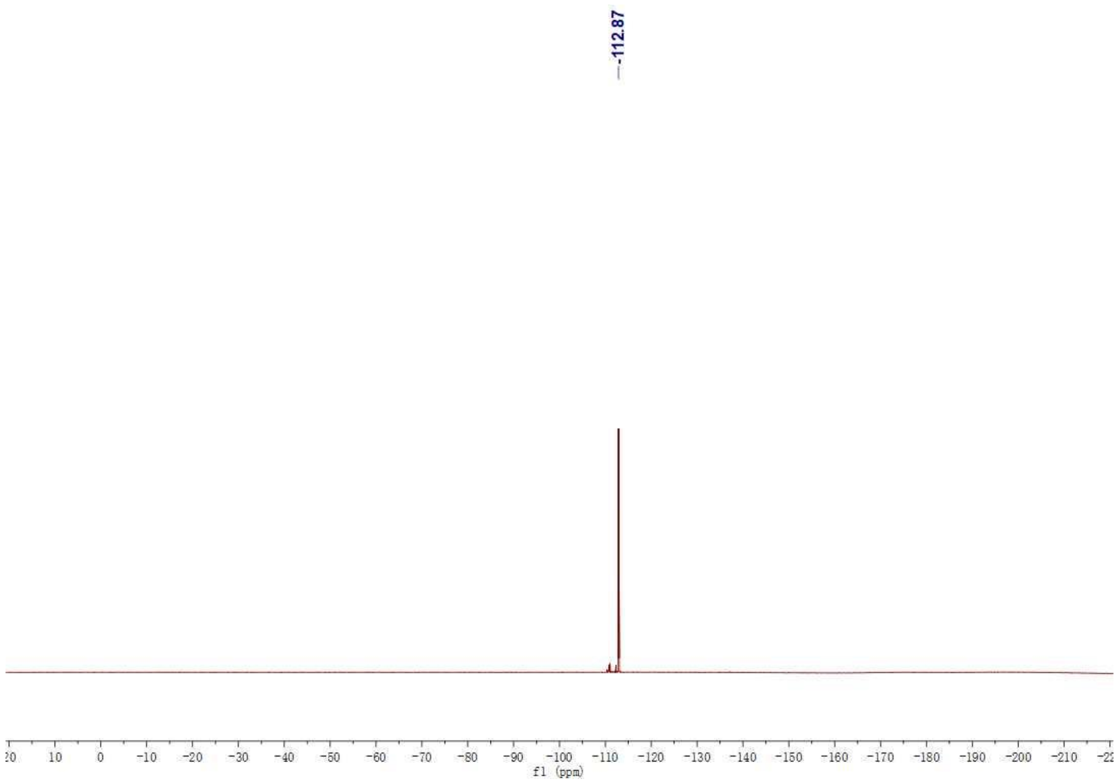

Supplementary Figure 85. ^{19}F NMR (376 MHz, $CDCl_3$) of **4f**

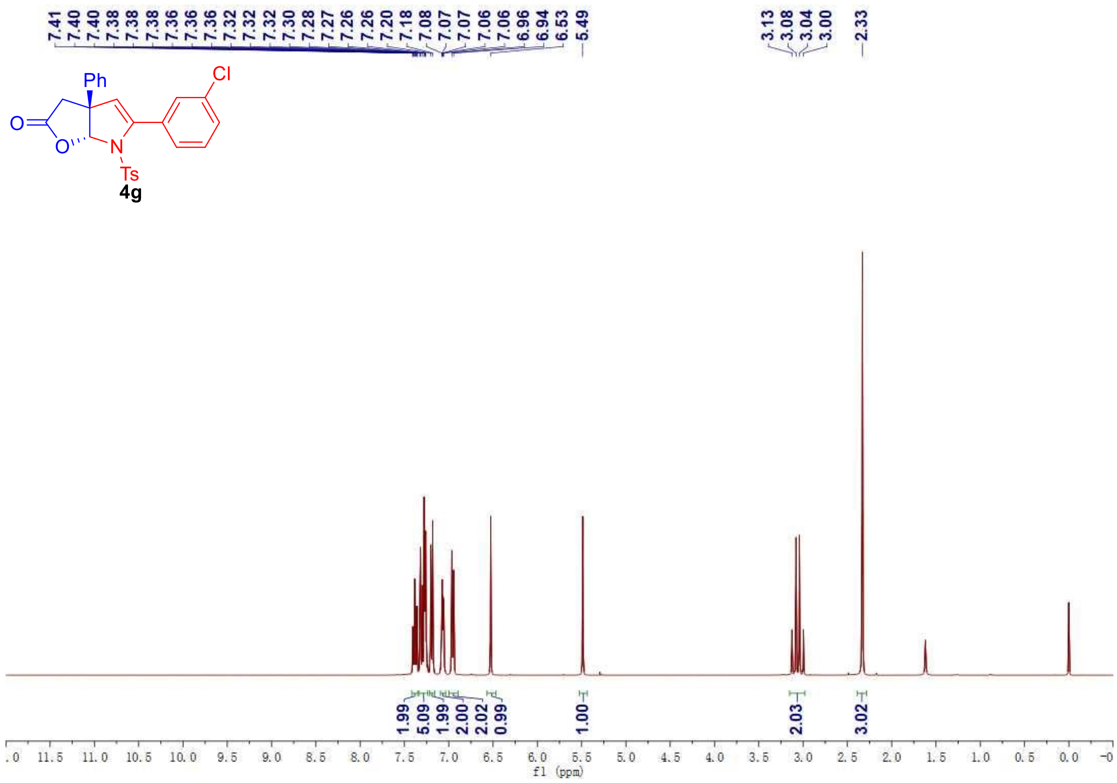

Supplementary Figure 86. ^{1}H NMR (400 MHz, $CDCl_3$) of **4g**

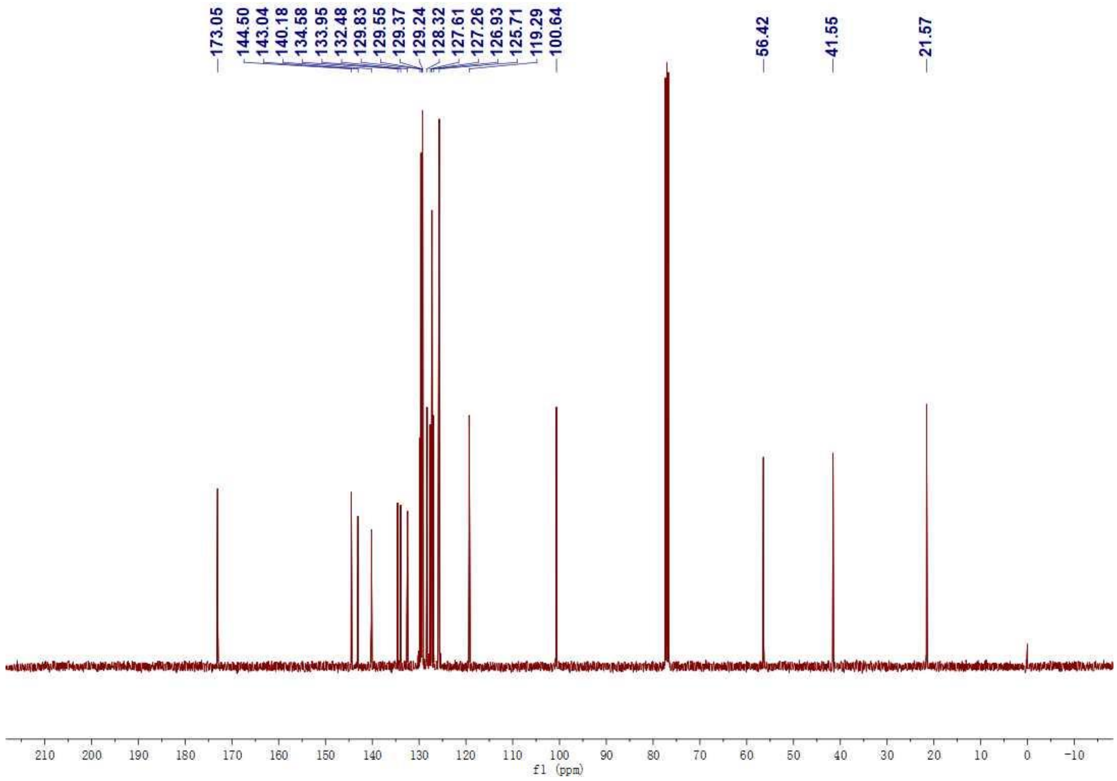

Supplementary Figure 87. ^{13}C NMR (101 MHz, $CDCl_3$) of **4g**

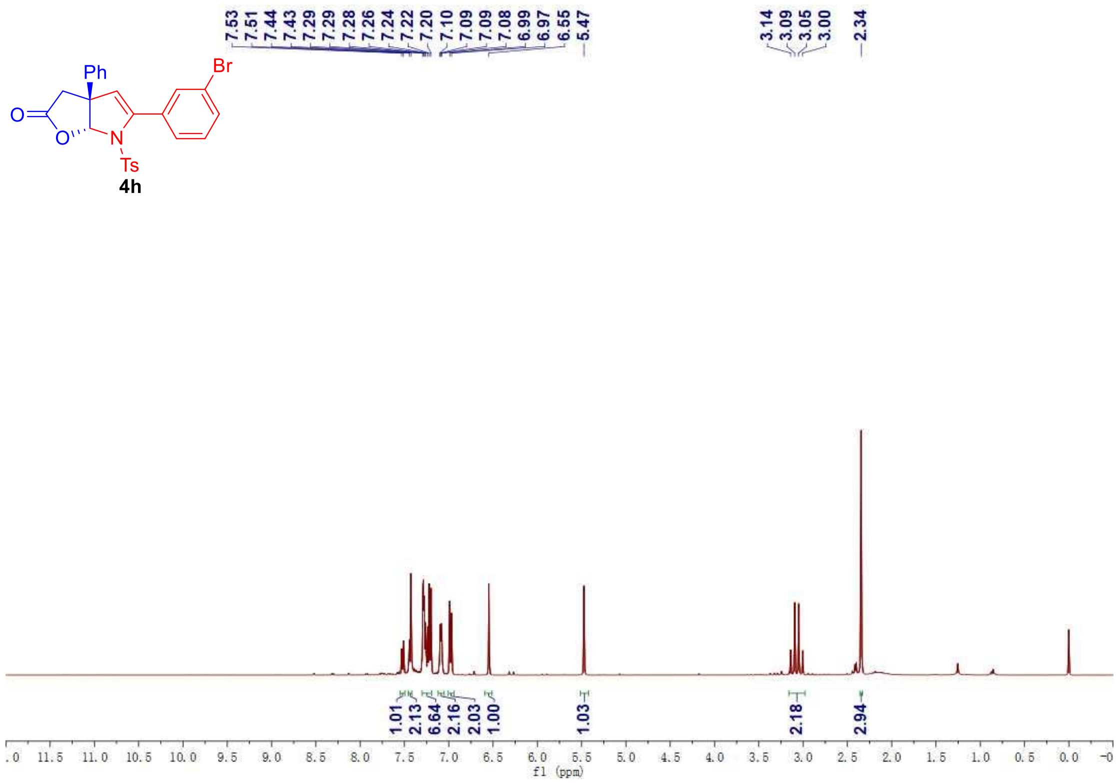

Supplementary Figure 88. ^{1}H NMR (400 MHz, $CDCl_3$) of **4h**

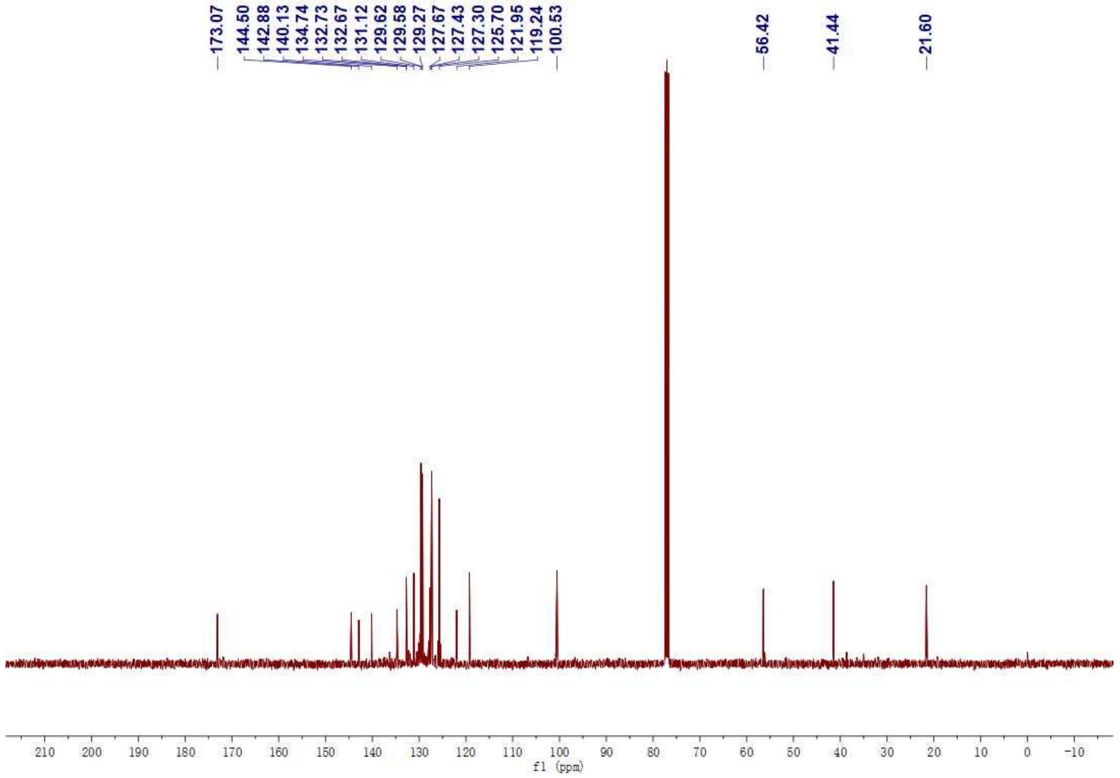

Supplementary Figure 89. ^{13}C NMR (101 MHz, $CDCl_3$) of **4h**

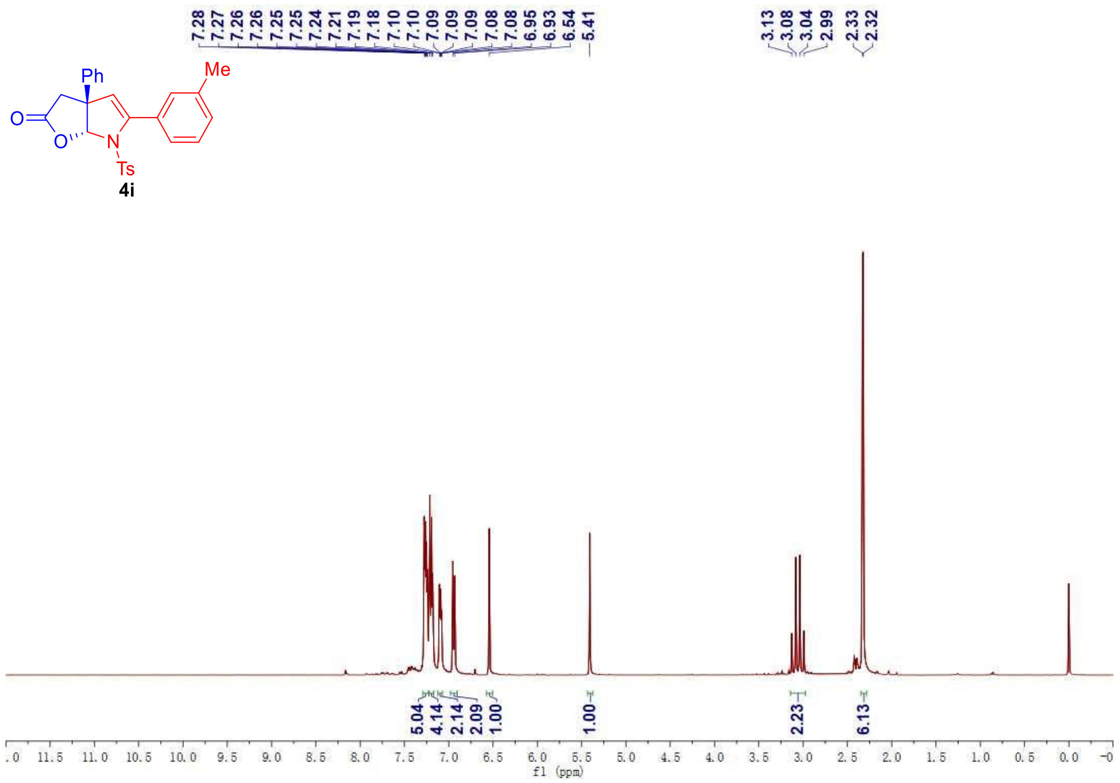

Supplementary Figure 90. ^{1}H NMR (400 MHz, $CDCl_3$) of **4i**

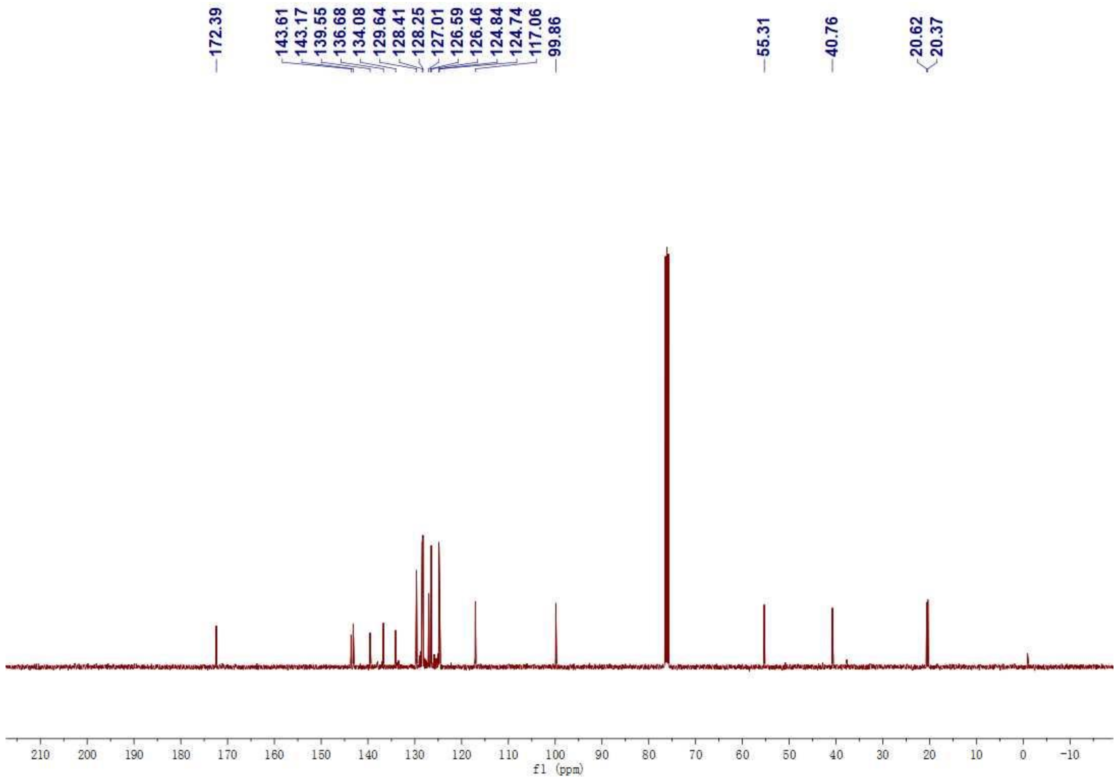

Supplementary Figure 91. ^{13}C NMR (101 MHz, $CDCl_3$) of **4i**

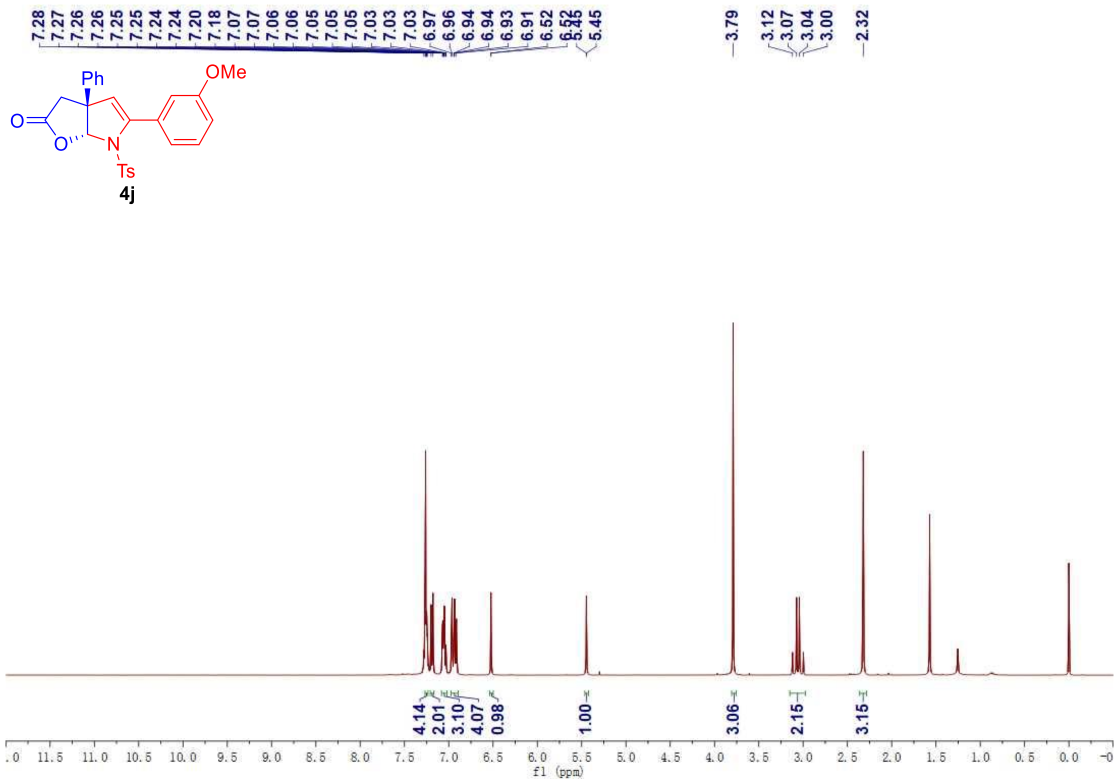

Supplementary Figure 92. ^{1}H NMR (400 MHz, $CDCl_3$) of **4j**

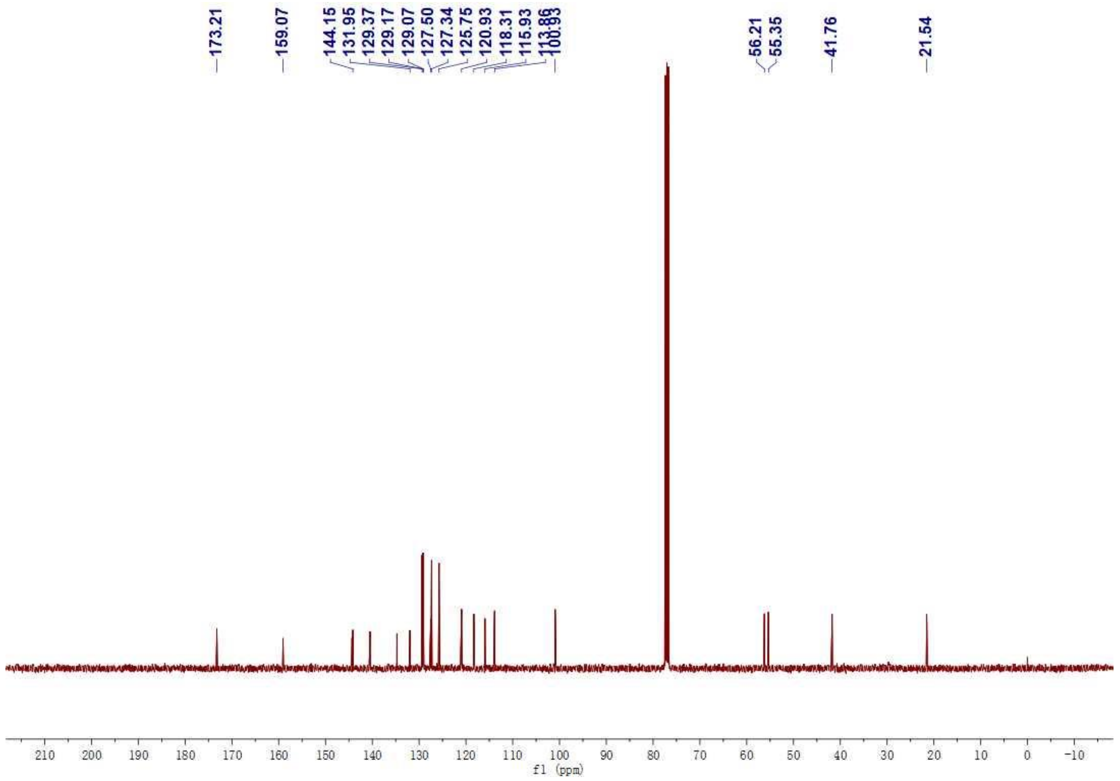

Supplementary Figure 93. ^{13}C NMR (101 MHz, $CDCl_3$) of **4j**

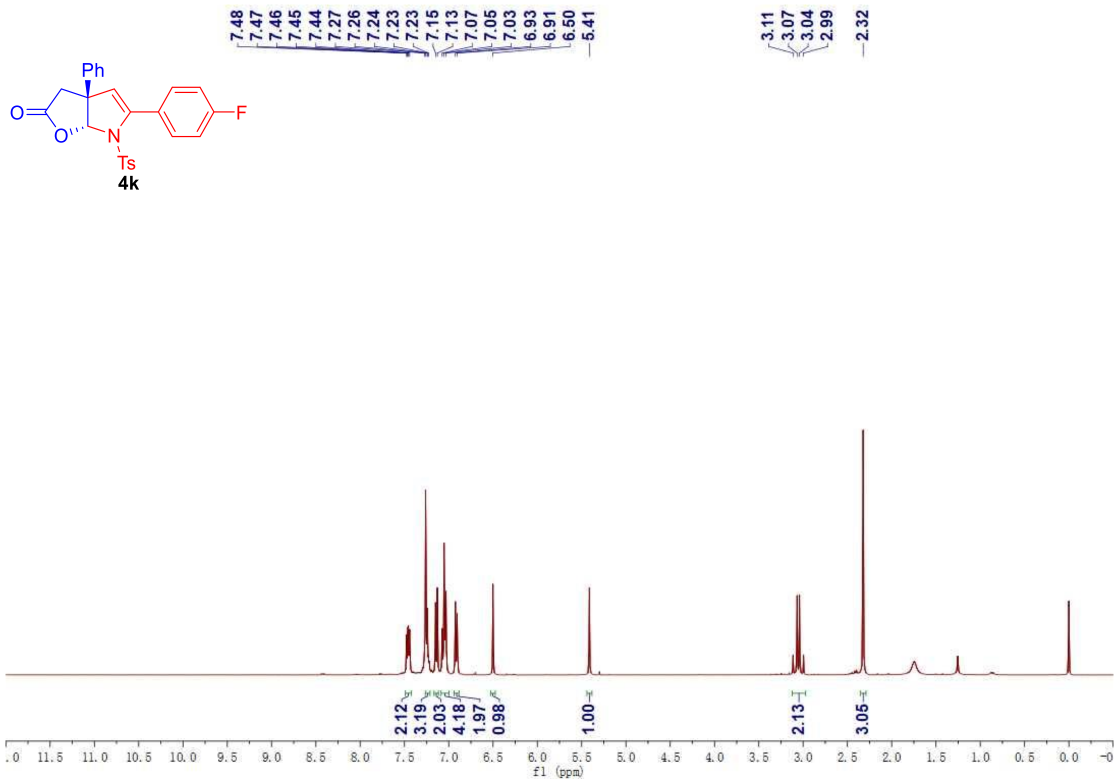

Supplementary Figure 94. ^{1}H NMR (400 MHz, $CDCl_3$) of **4k**

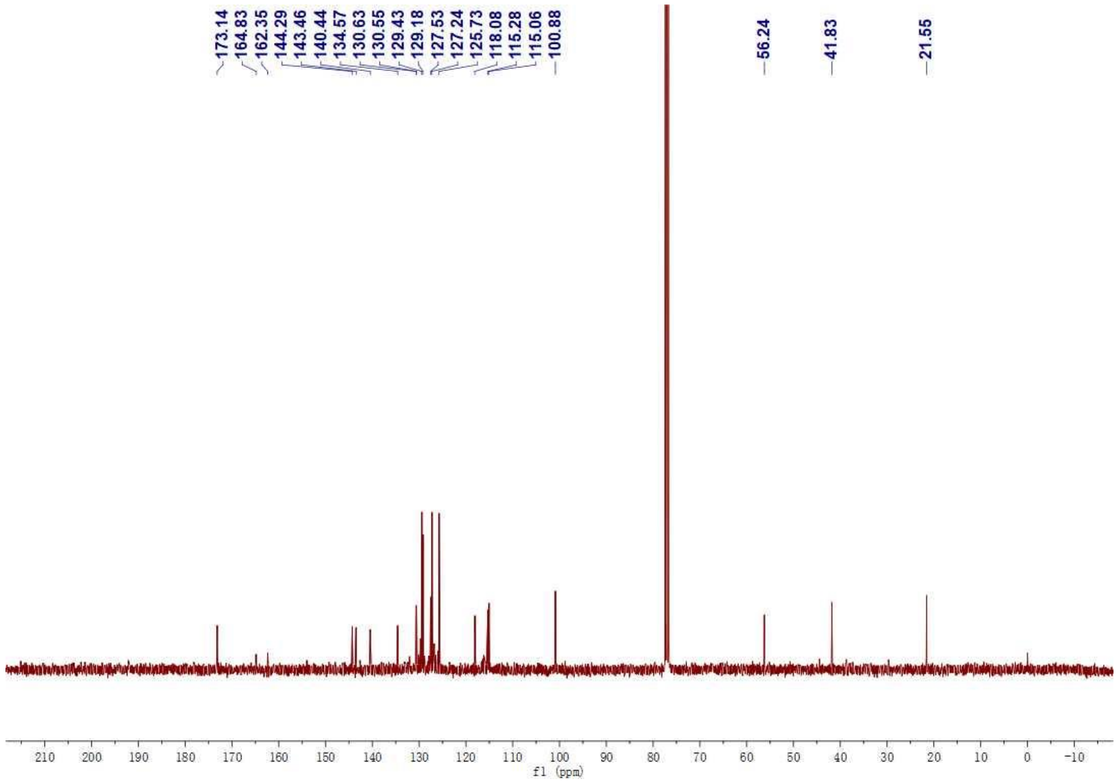

Supplementary Figure 95. ^{13}C NMR (101 MHz, $CDCl_3$) of **4k**

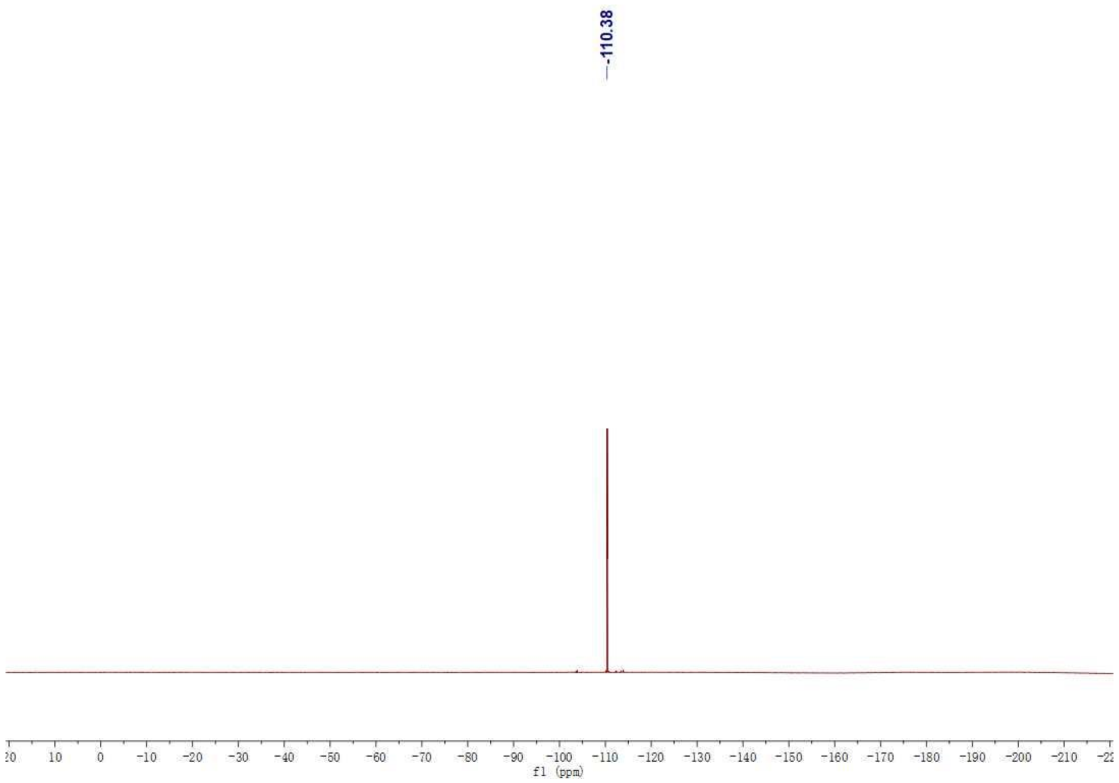

Supplementary Figure 96. ^{19}F NMR (101 MHz, $CDCl_3$) of **4k**

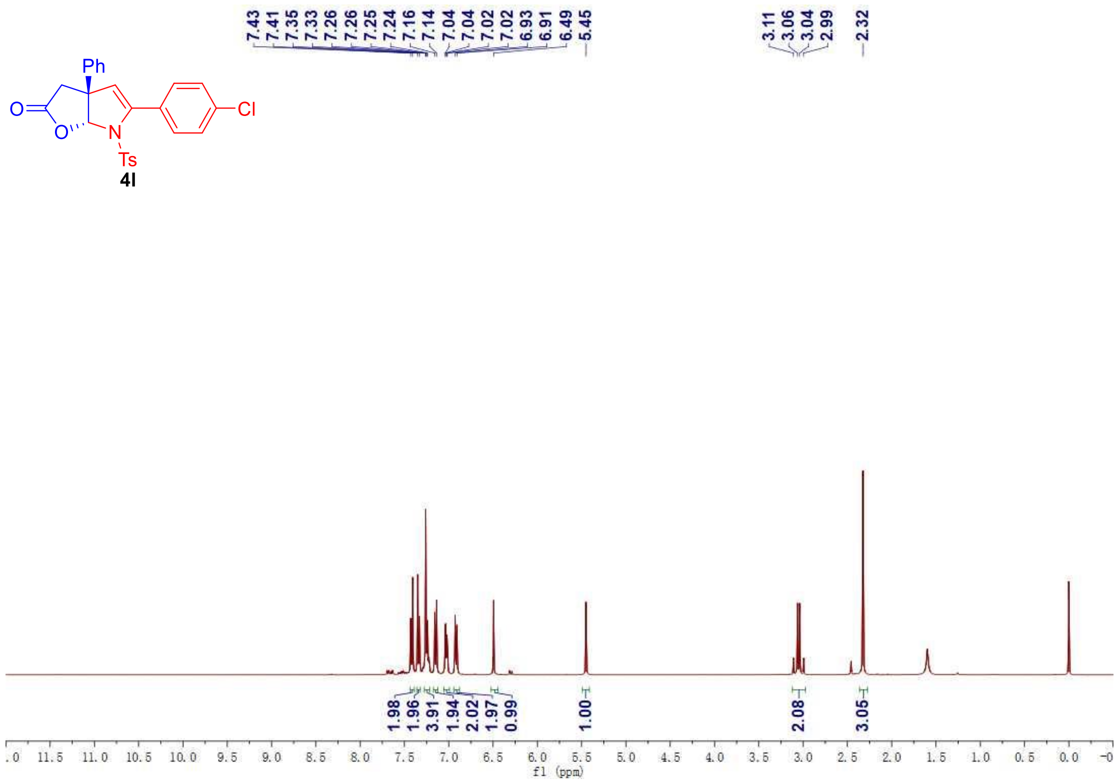

Supplementary Figure 97. ^{1}H NMR (400 MHz, $CDCl_3$) of **4l**

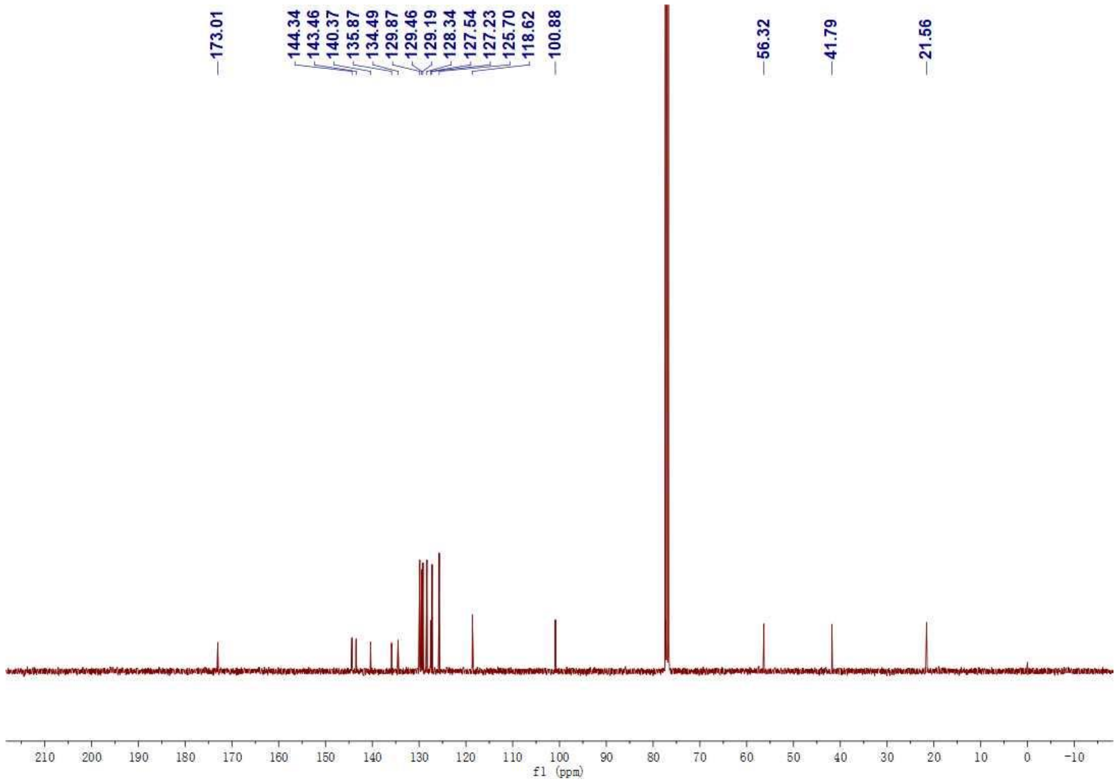

Supplementary Figure 98. ^{13}C NMR (101 MHz, $CDCl_3$) of **4l**

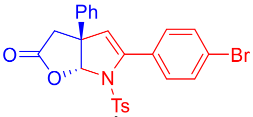

4m

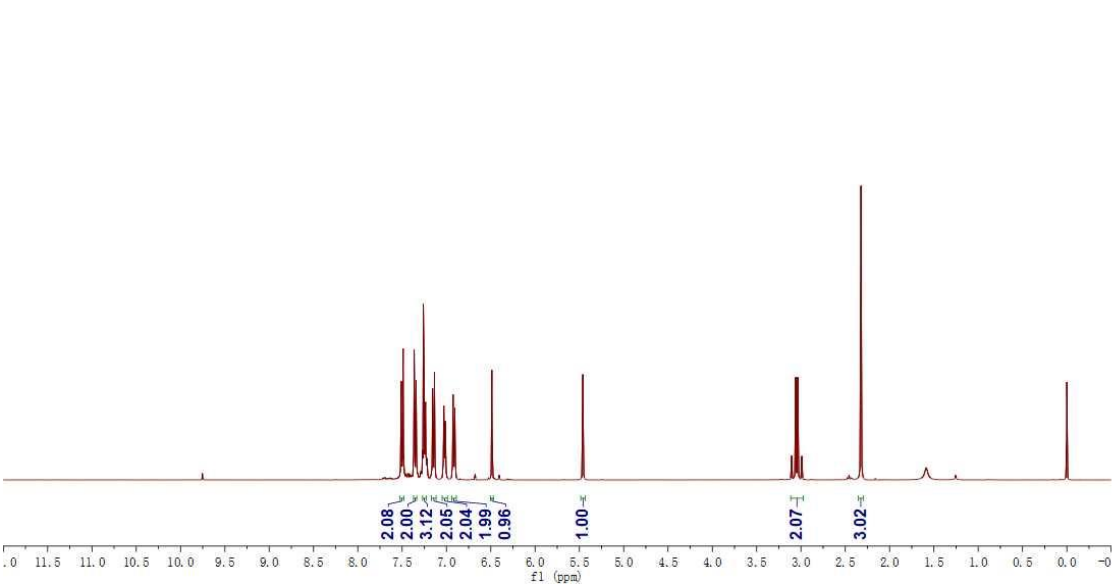

Supplementary Figure 99. ^{1}H NMR (400 MHz, $CDCl_3$) of **4m**

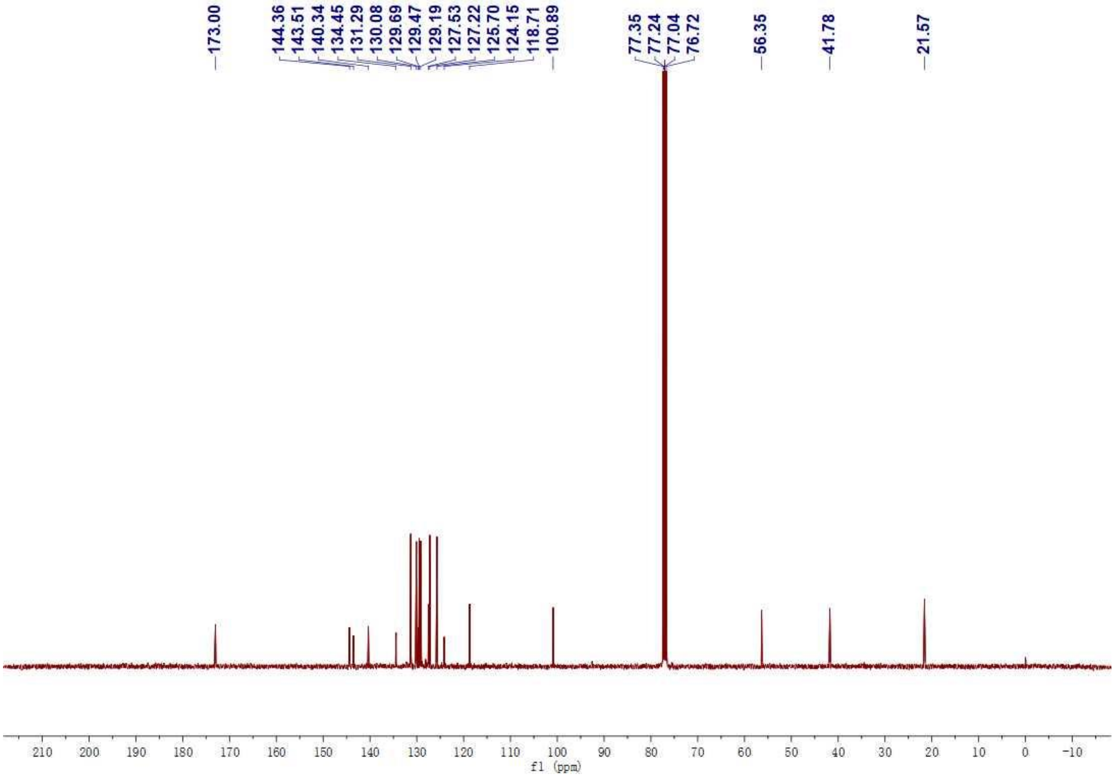

Supplementary Figure 100. ^{13}C NMR (101 MHz, $CDCl_3$) of **4m**

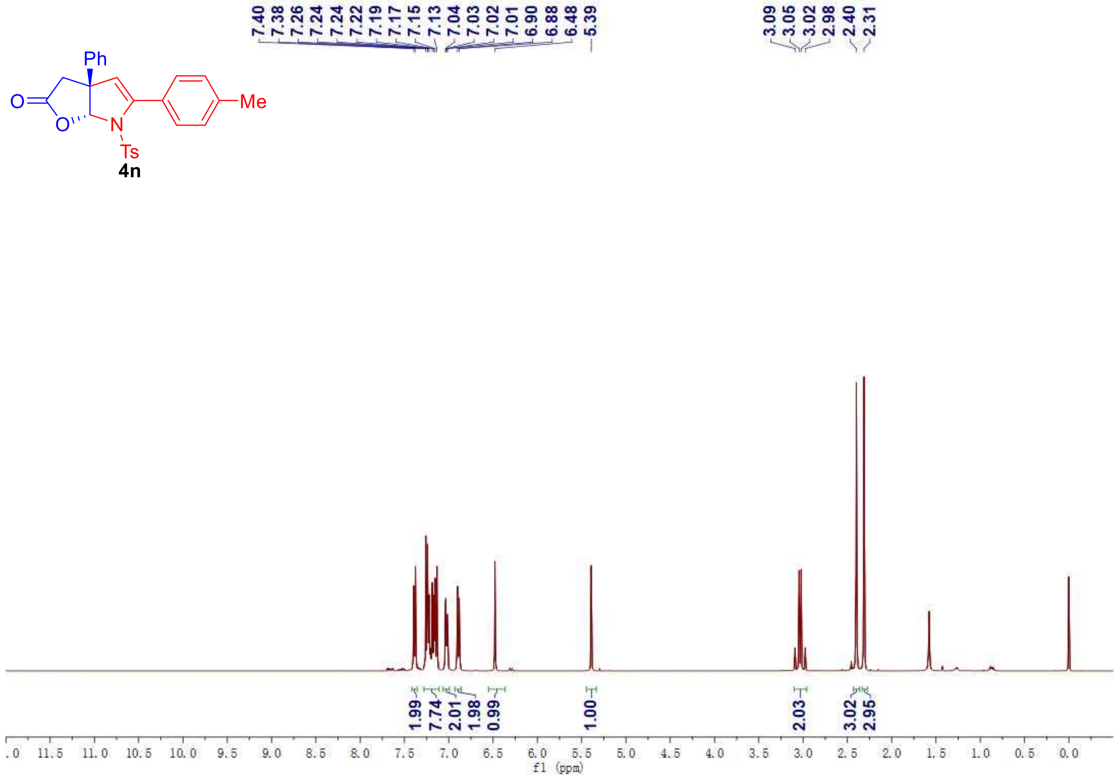

Supplementary Figure 101. ^{1}H NMR (400 MHz, $CDCl_3$) of **4n**

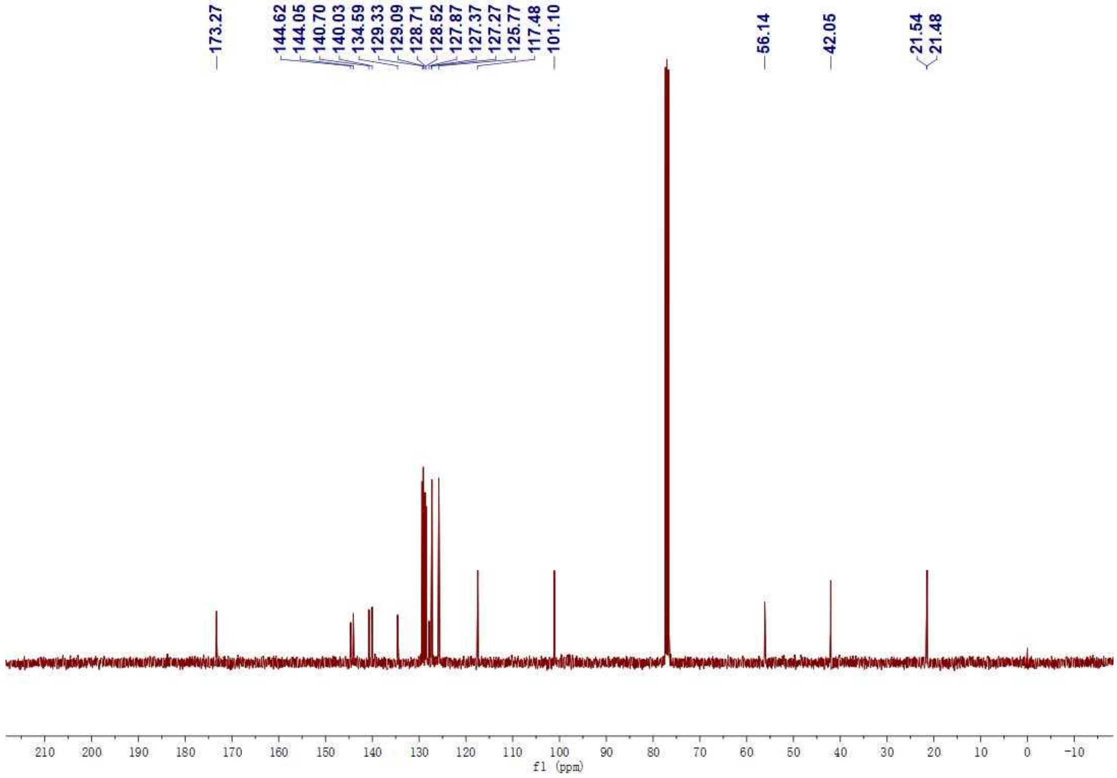

Supplementary Figure 102. ^{13}C NMR (101 MHz, $CDCl_3$) of **4n**

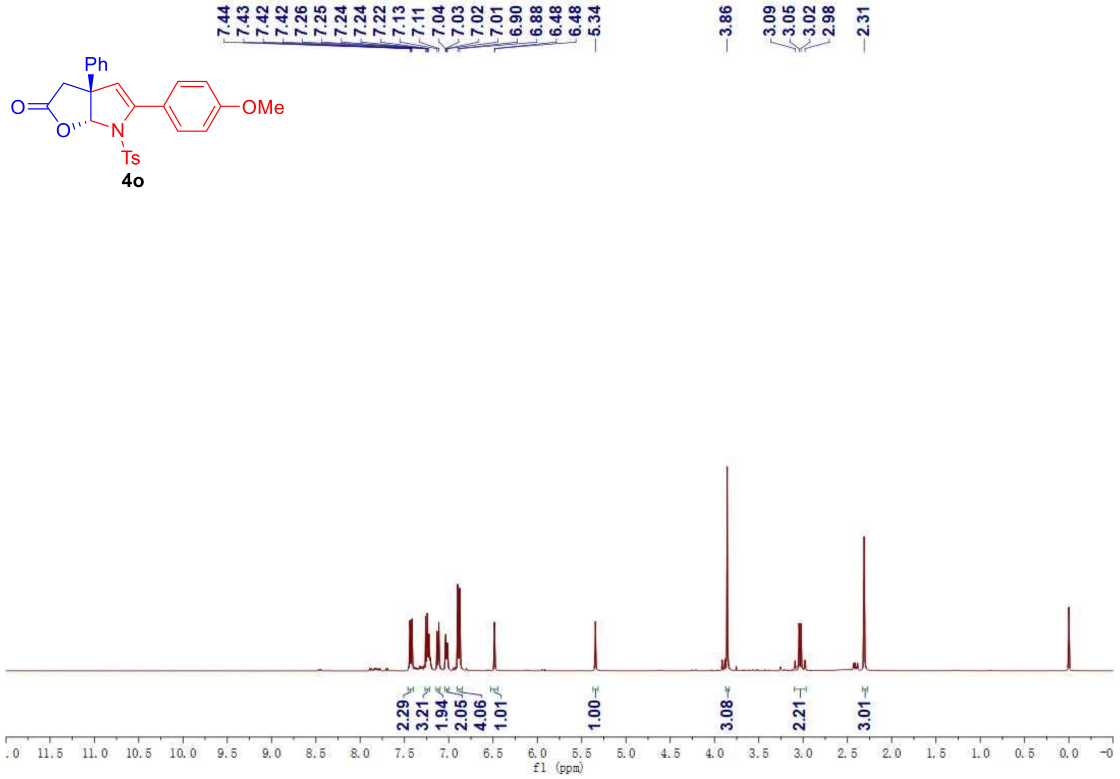

Supplementary Figure 103. ^{1}H NMR (400 MHz, $CDCl_3$) of **4o**

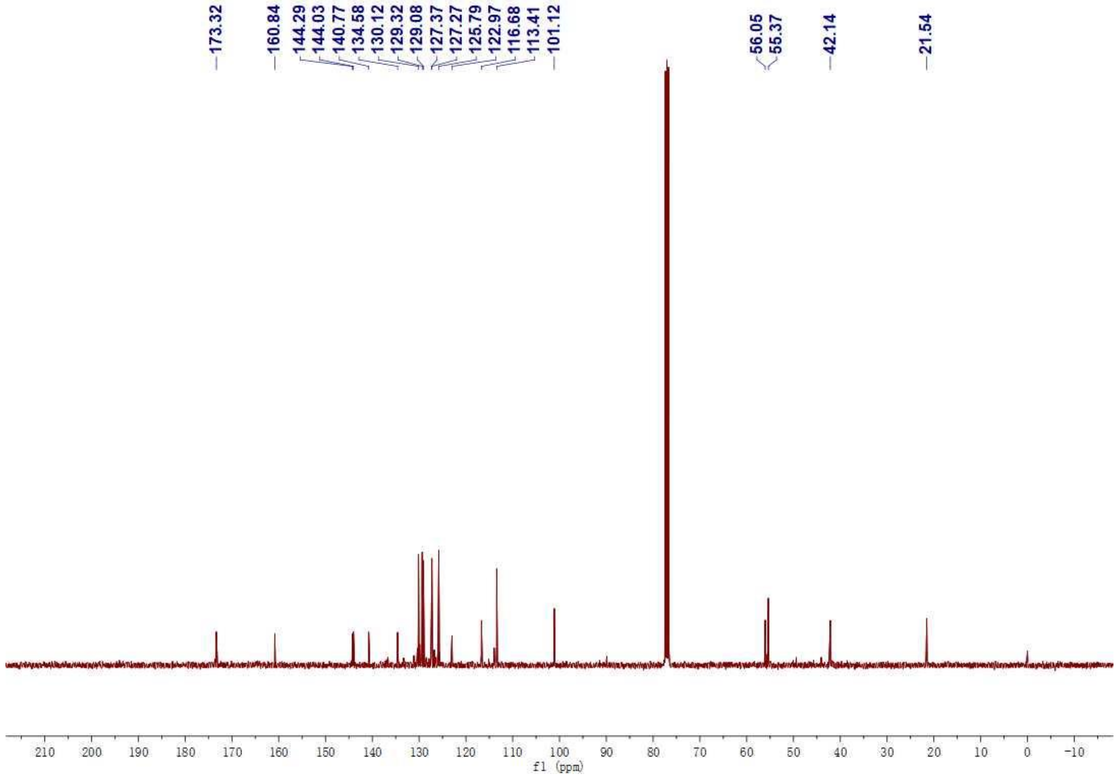

Supplementary Figure 104. ^{13}C NMR (101 MHz, $CDCl_3$) of **4o**

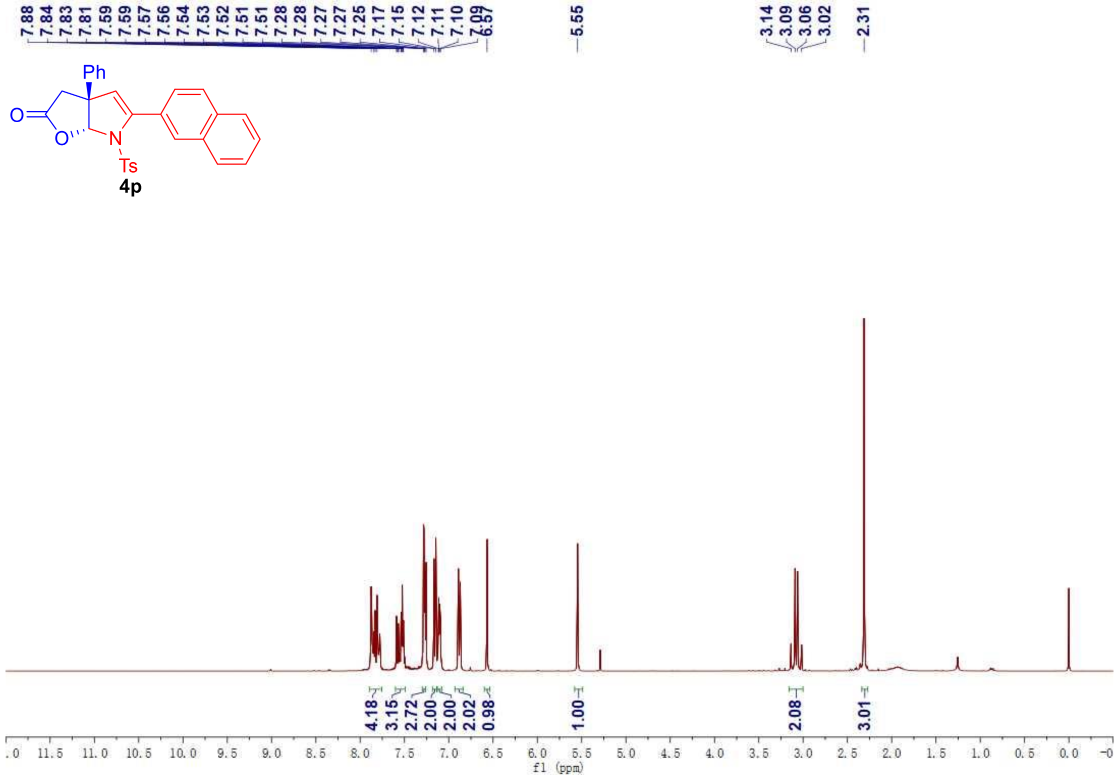

Supplementary Figure 105. ^{1}H NMR (400 MHz, $CDCl_3$) of **4p**

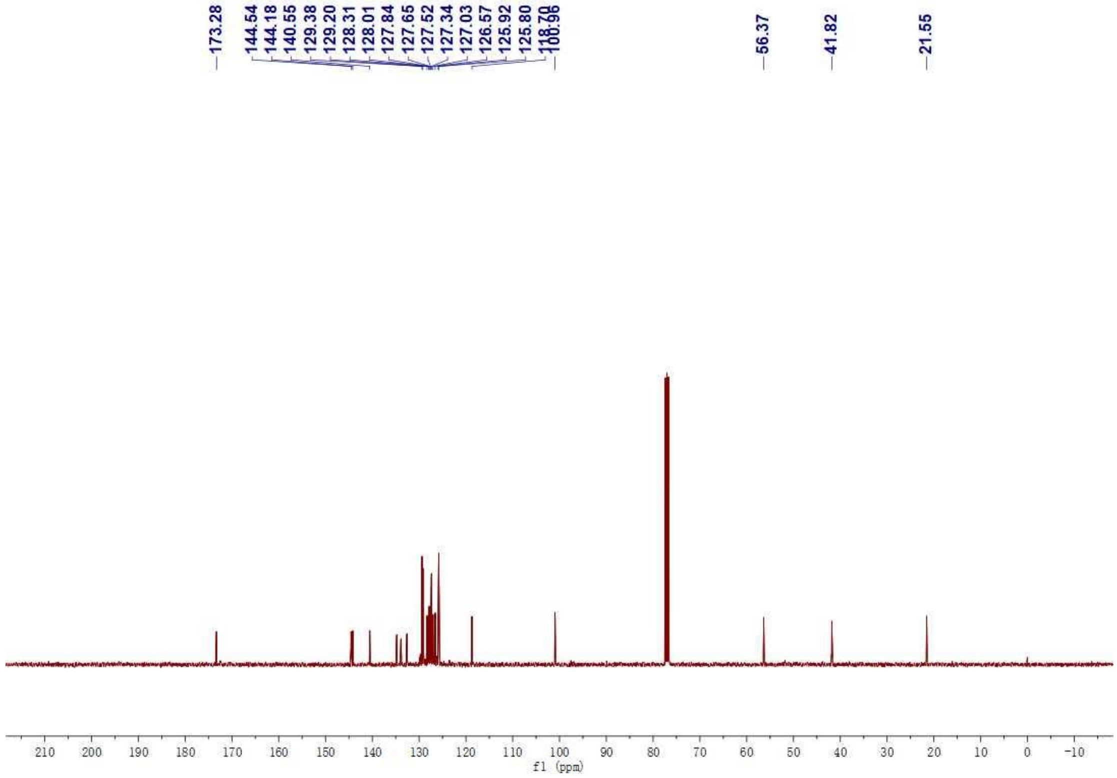

Supplementary Figure 106. ^{13}C NMR (101 MHz, $CDCl_3$) of **4p**

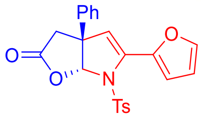

4q

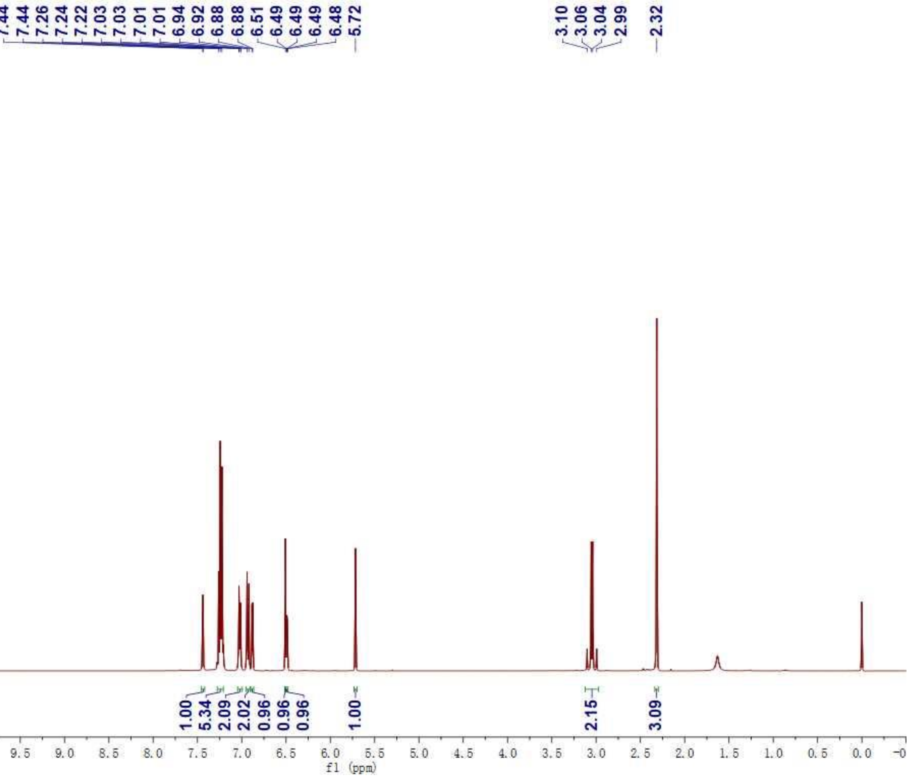

Supplementary Figure 107. ^{1}H NMR (400 MHz, $CDCl_3$) of **4q**

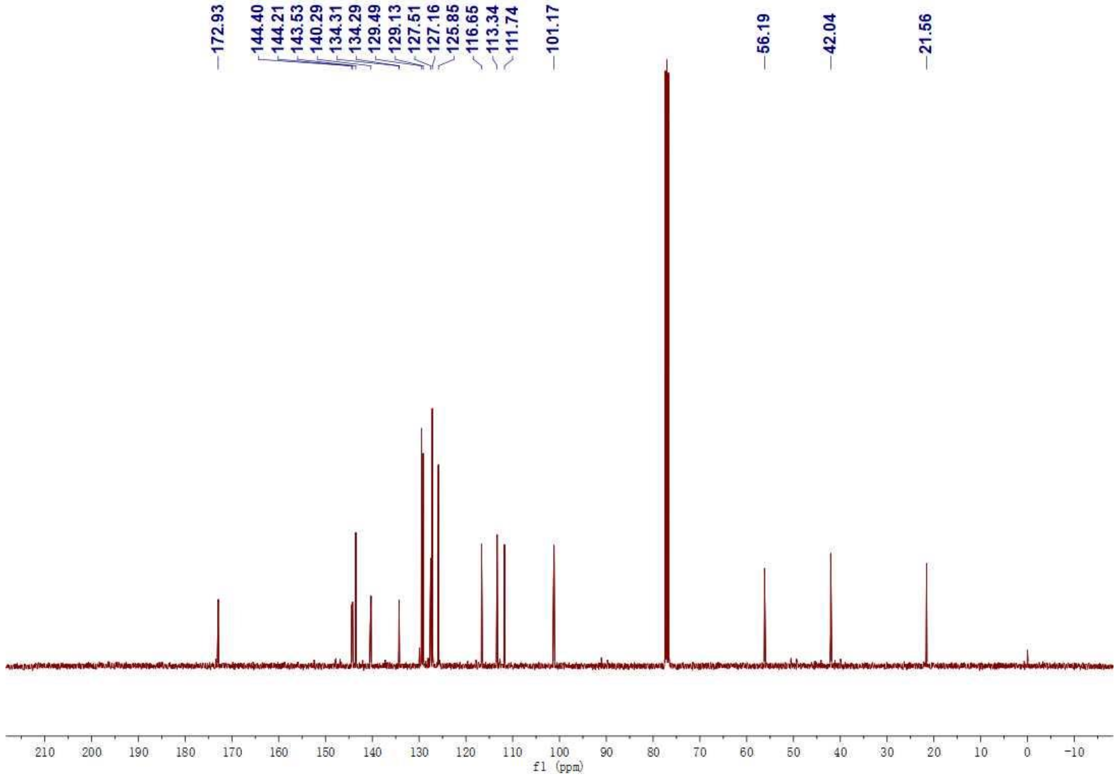

Supplementary Figure 108. ^{13}C NMR (101 MHz, $CDCl_3$) of **4q**

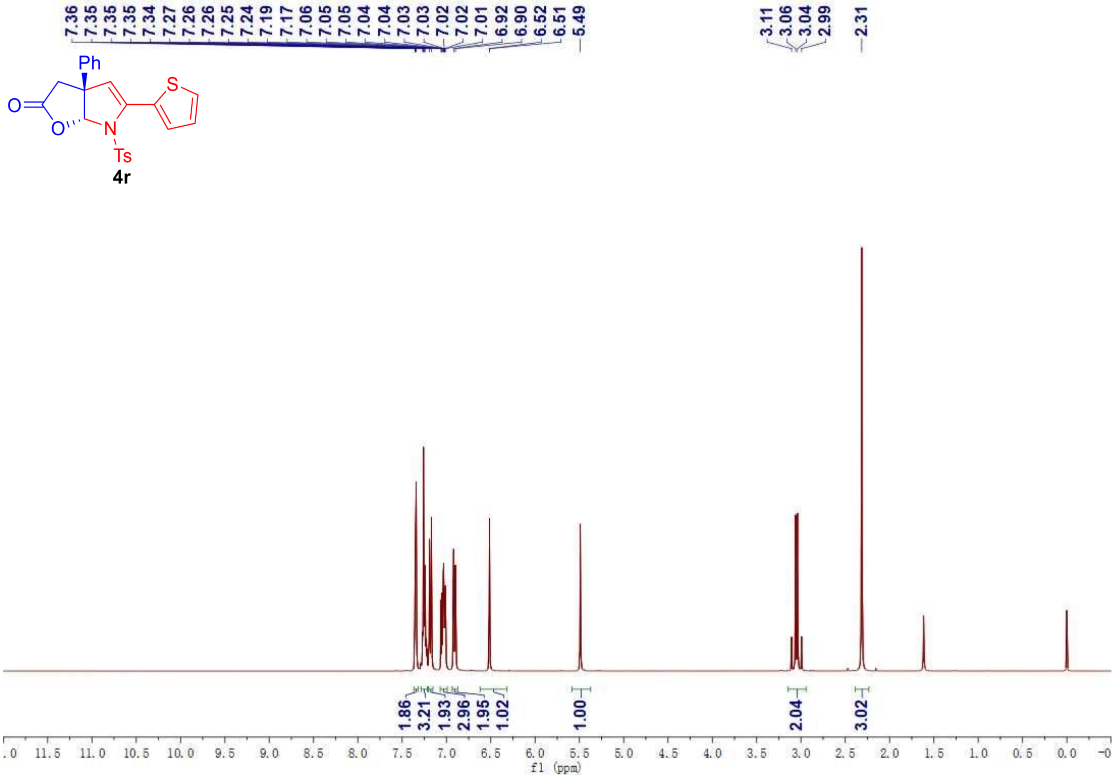

Supplementary Figure 109. ^{1}H NMR (400 MHz, $CDCl_3$) of **4r**

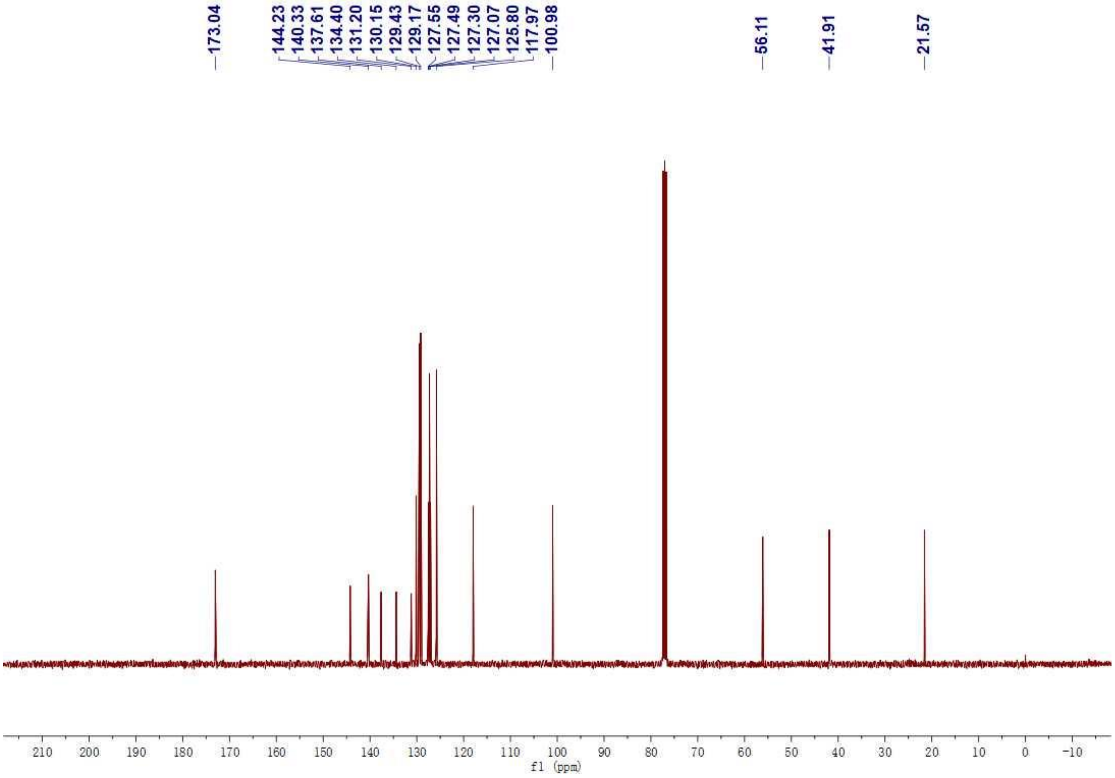

Supplementary Figure 110. ^{13}C NMR (101 MHz, $CDCl_3$) of **4r**

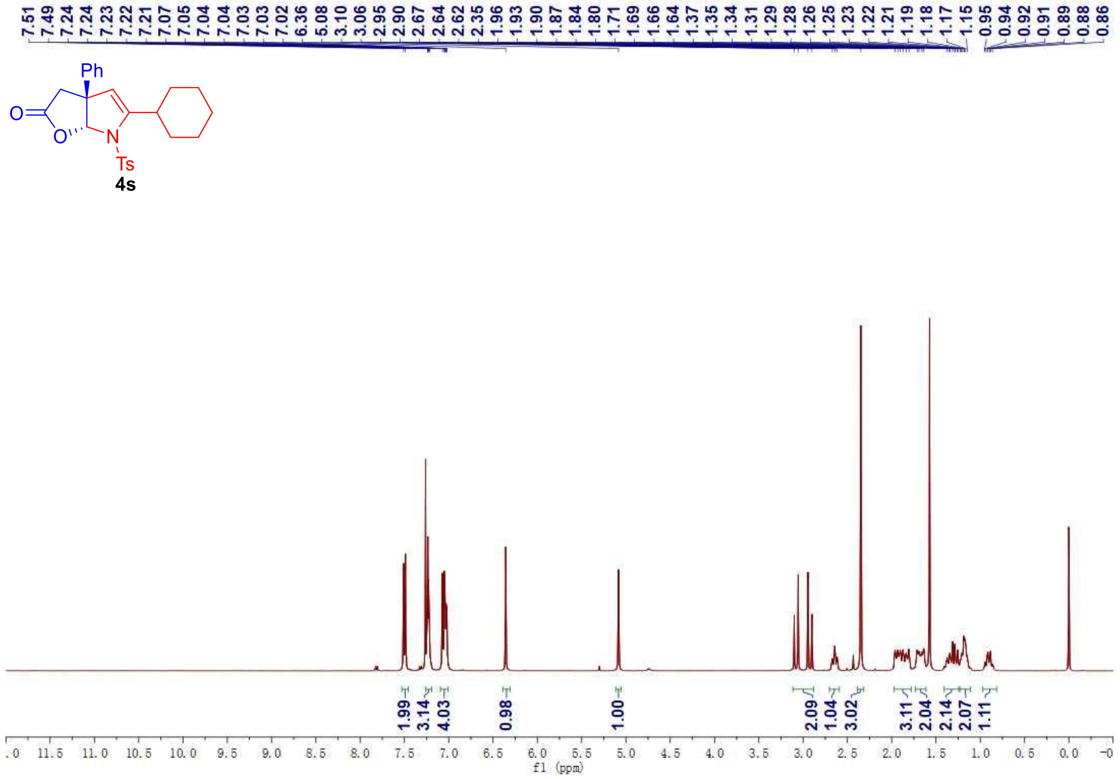

Supplementary Figure 111. ^{1}H NMR (400 MHz, $CDCl_3$) of **4s**

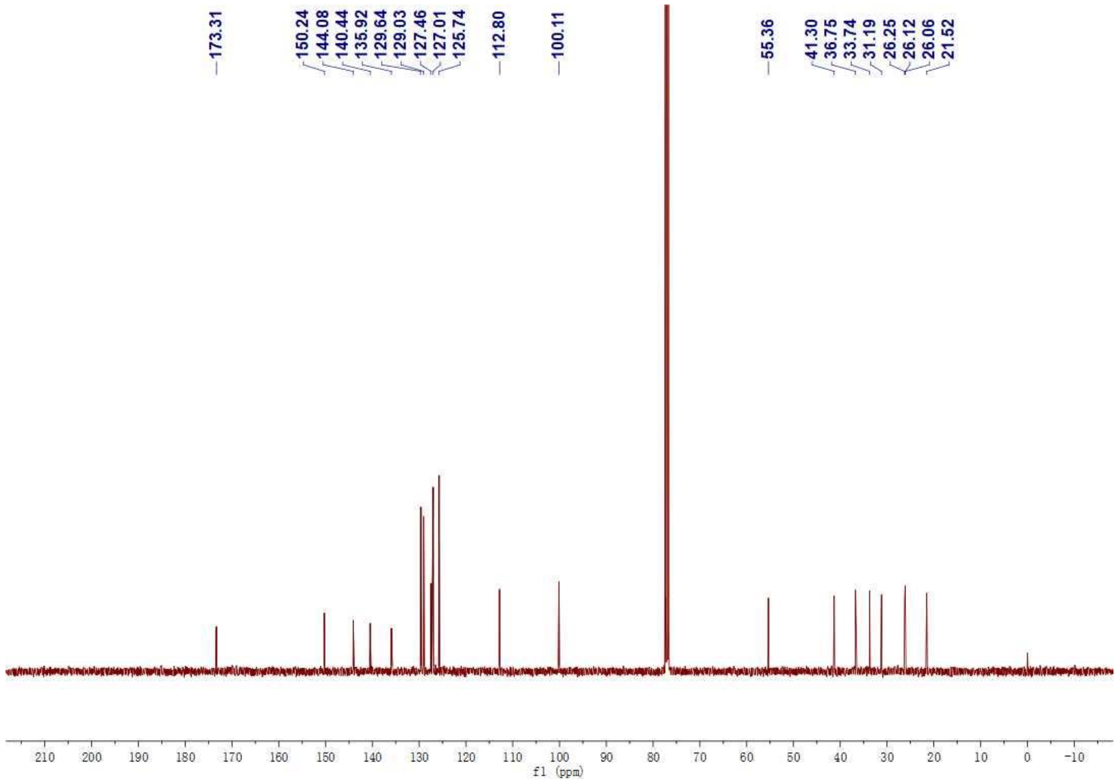

Supplementary Figure 112. ^{13}C NMR (101 MHz, $CDCl_3$) of **4s**

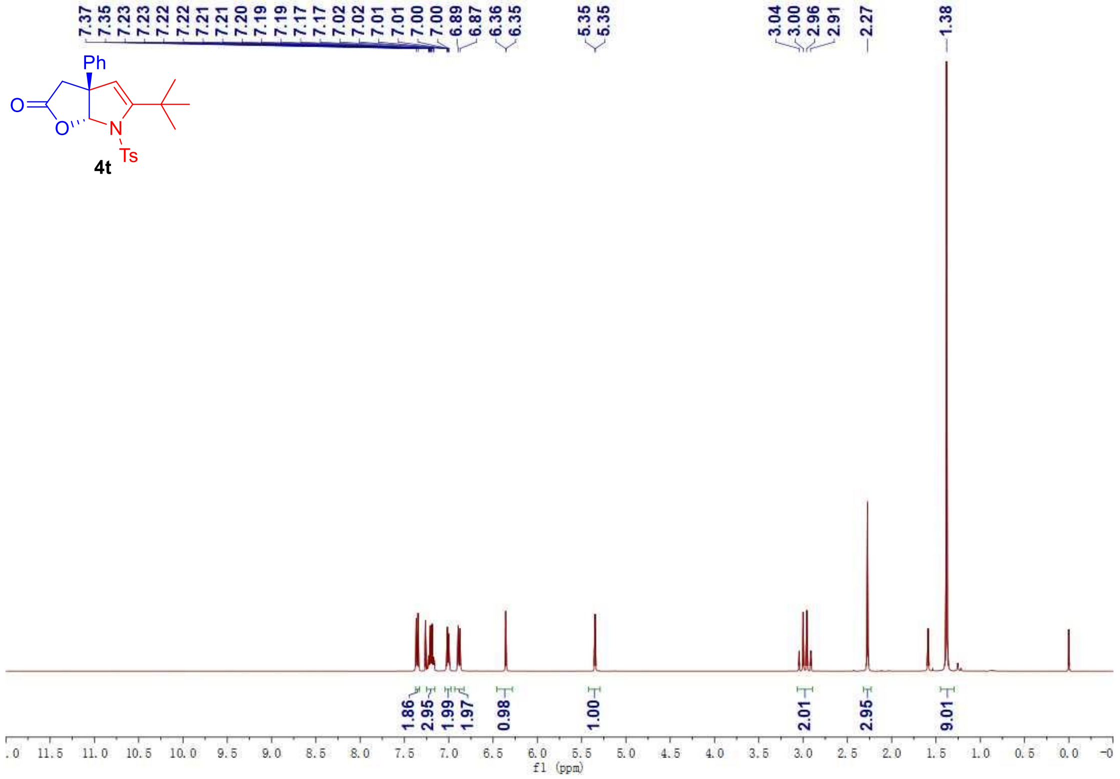

Supplementary Figure 113. ^{1}H NMR (400 MHz, $CDCl_3$) of **4t**

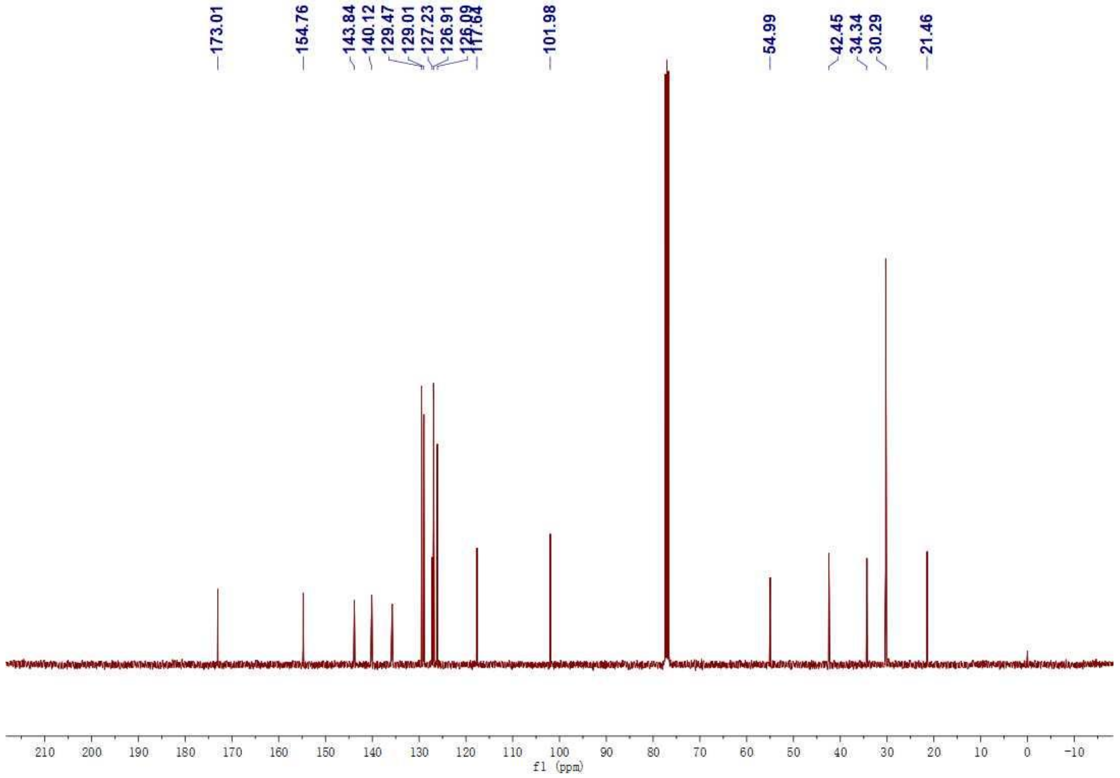

Supplementary Figure 114. ^{13}C NMR (101 MHz, $CDCl_3$) of **4t**

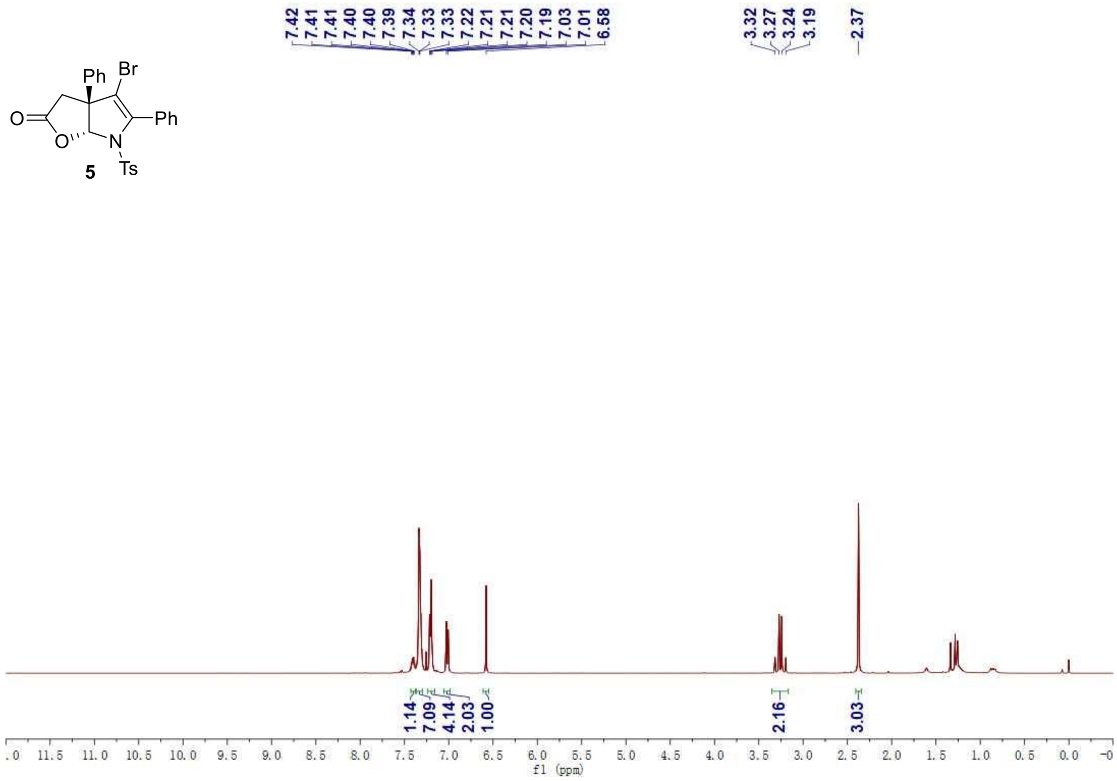

Supplementary Figure 115. ^{1}H NMR (400 MHz, $CDCl_3$) of **5**

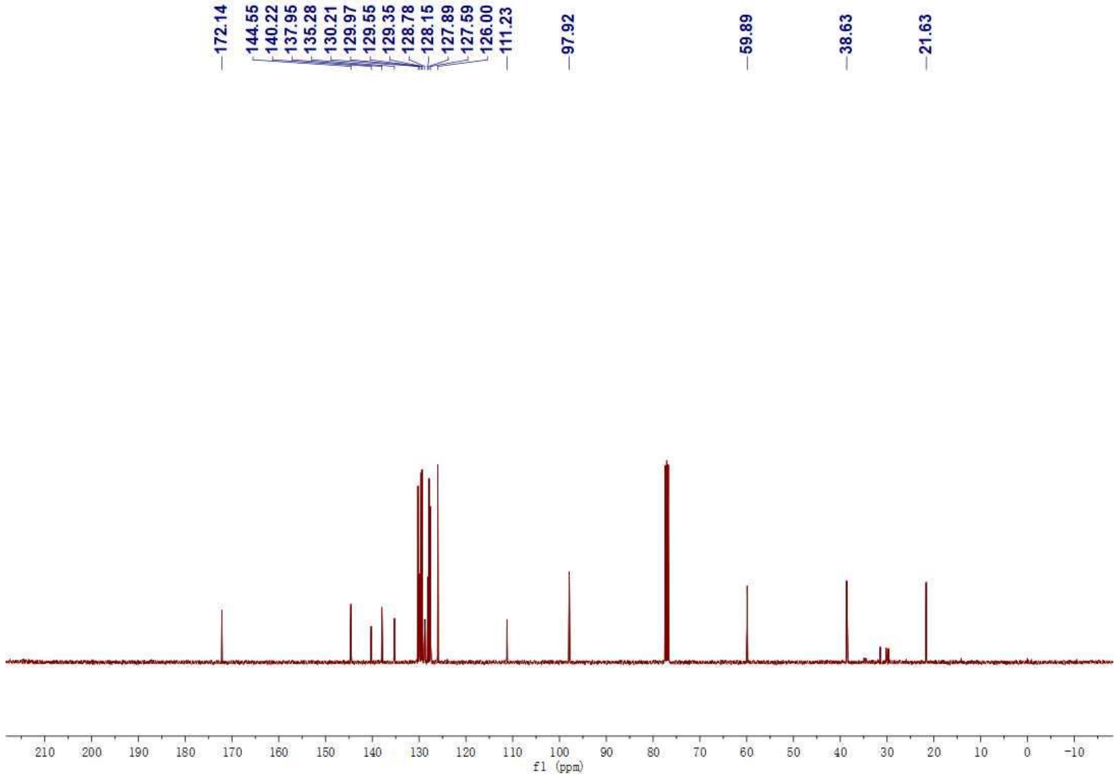

Supplementary Figure 116. ^{13}C NMR (101 MHz, $CDCl_3$) of **5**

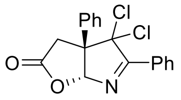

6

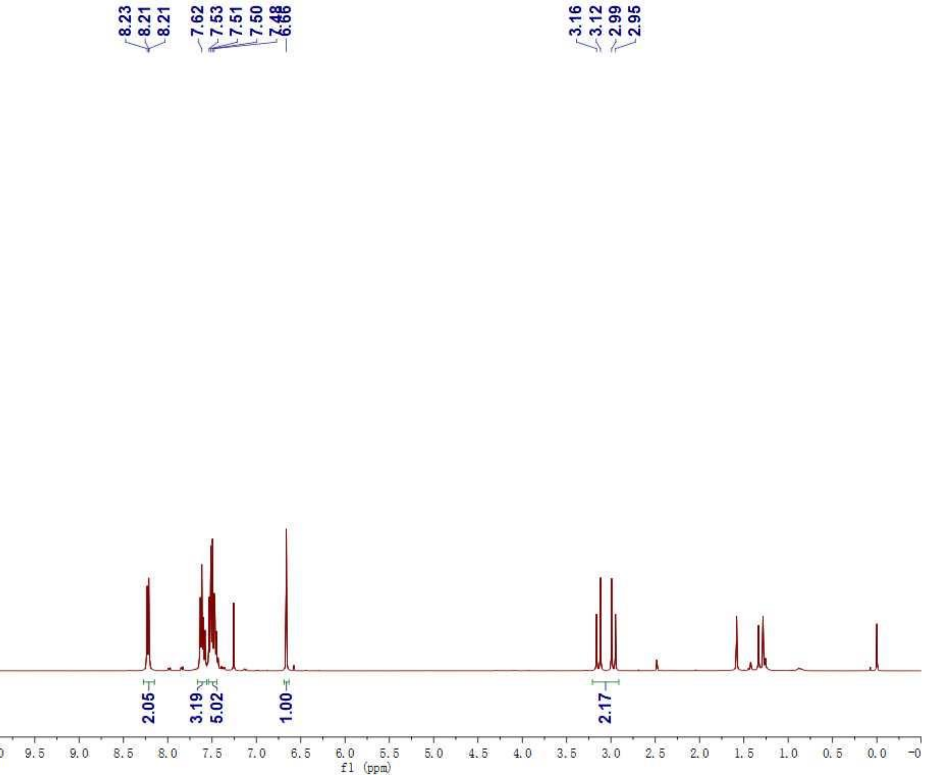

Supplementary Figure 117. ^{1}H NMR (400 MHz, $CDCl_3$) of **6**

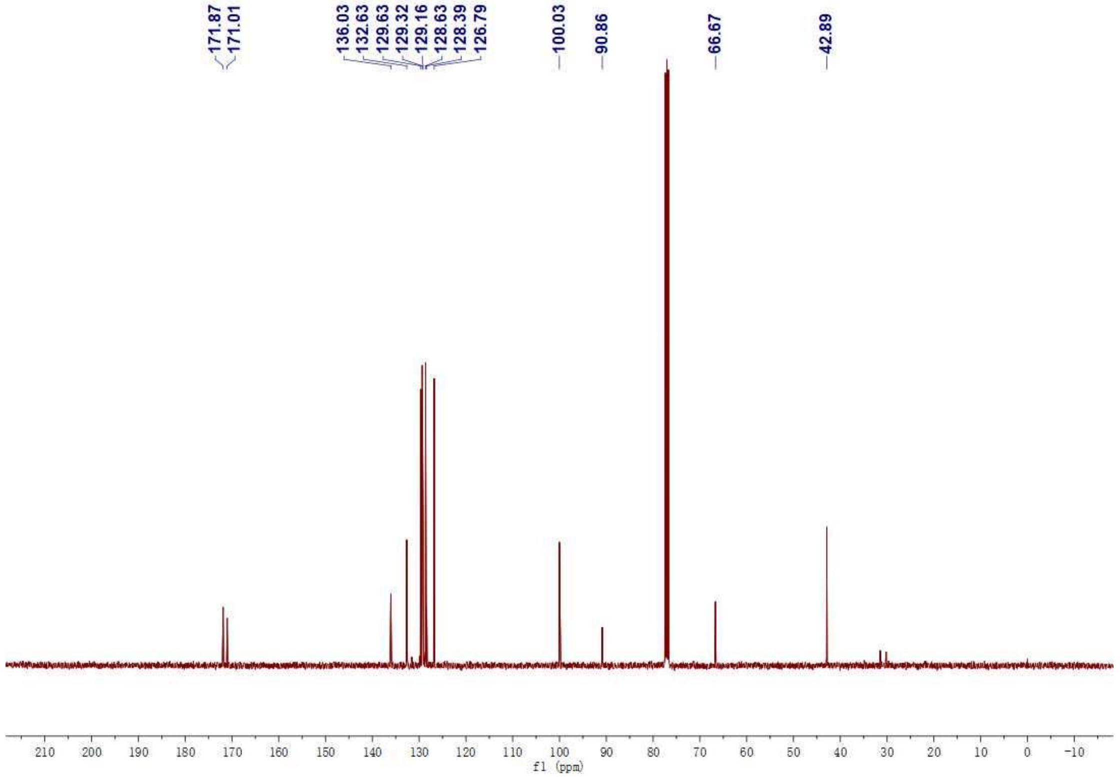

Supplementary Figure 118. ^{13}C NMR (101 MHz, $CDCl_3$) of **6**

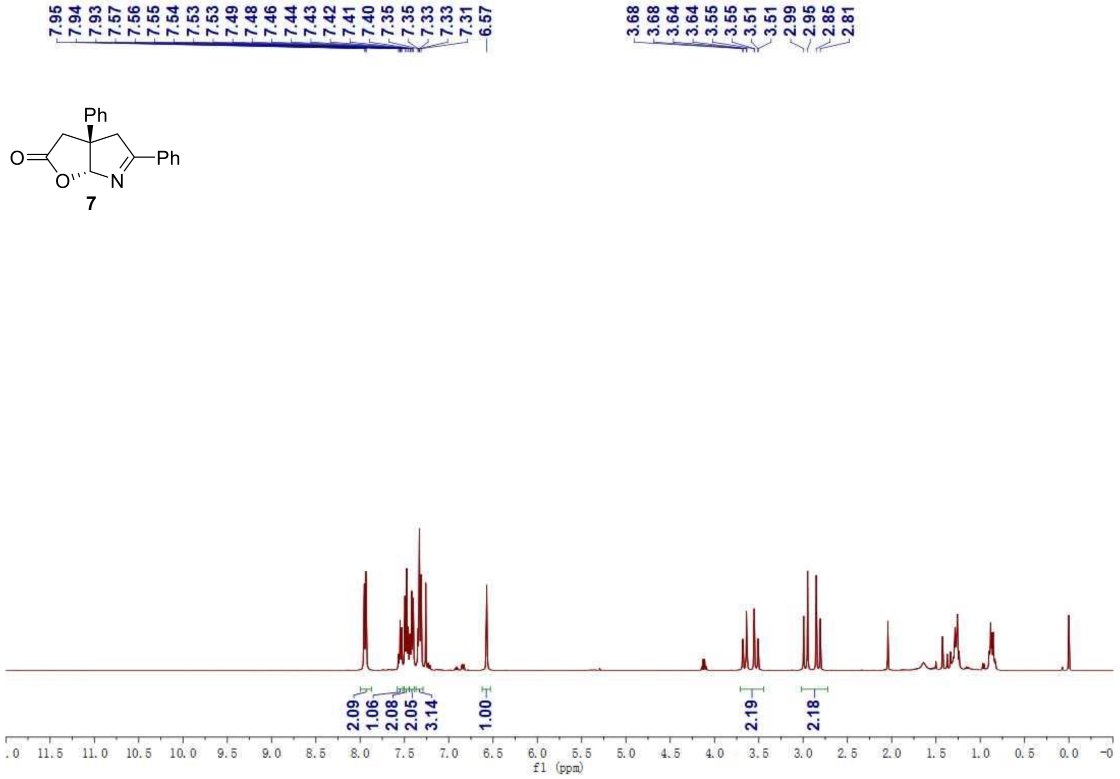

Supplementary Figure 119. ^{1}H NMR (400 MHz, $CDCl_3$) of **7**

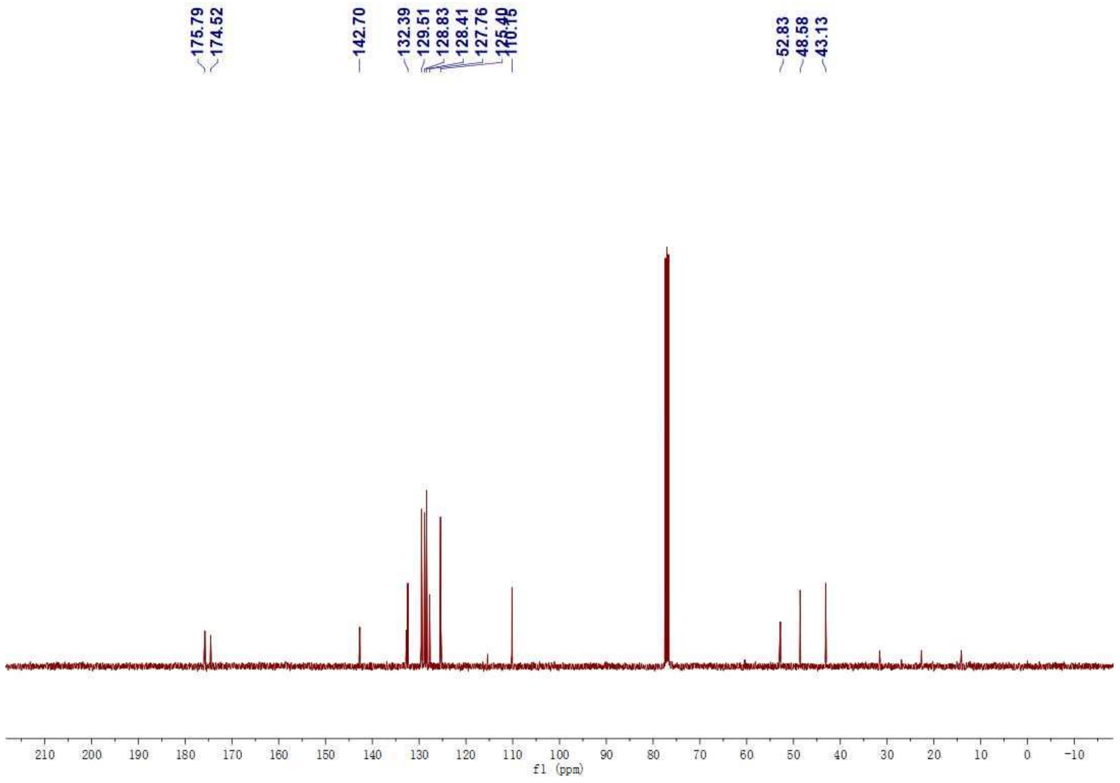

Supplementary Figure 120. ^{13}C NMR (101 MHz, $CDCl_3$) of **7**

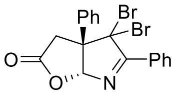

8

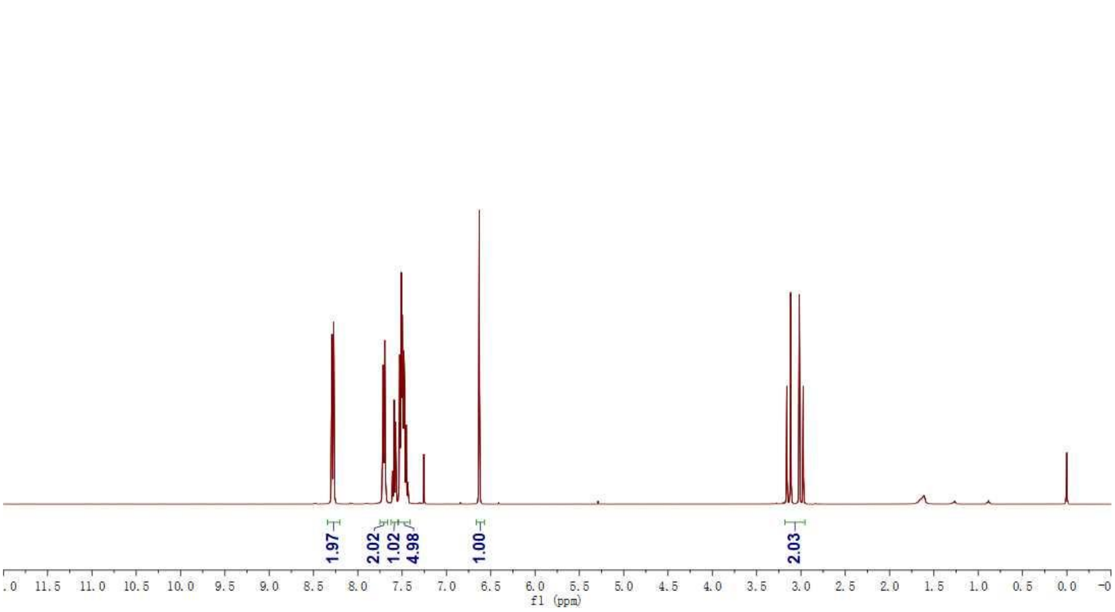

Supplementary Figure 121. ^{1}H NMR (400 MHz, $CDCl_3$) of **8**

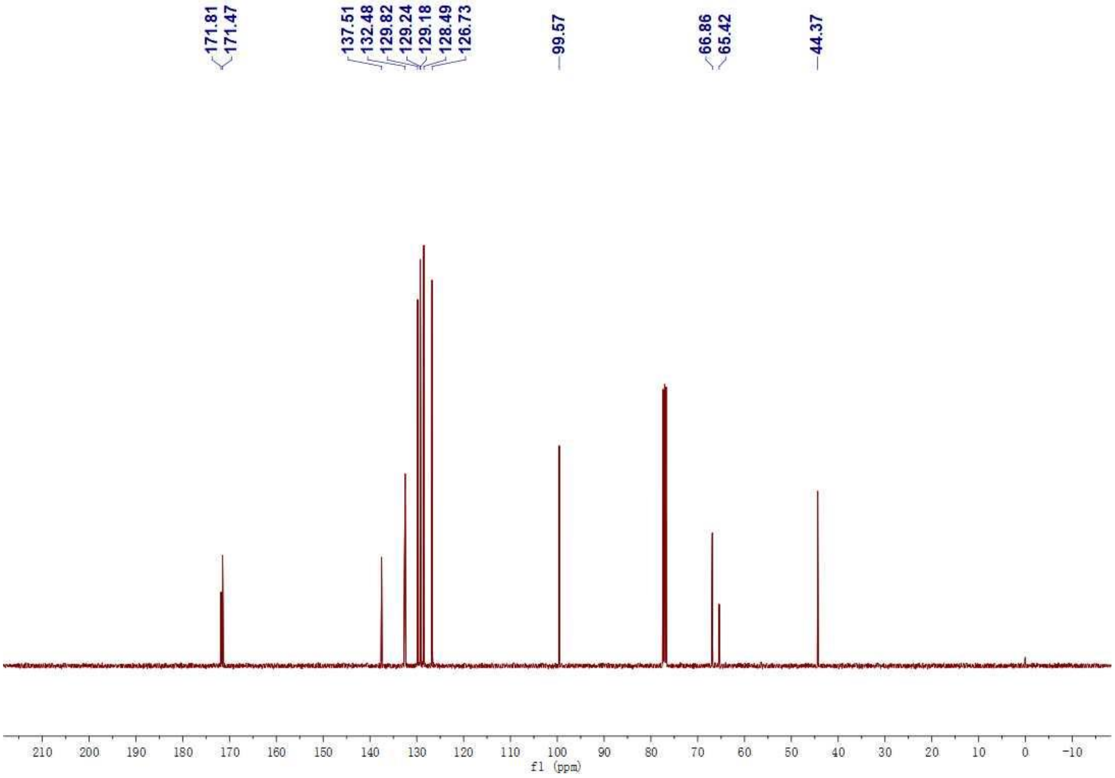

Supplementary Figure 122. ^{13}C NMR (101 MHz, $CDCl_3$) of **8**

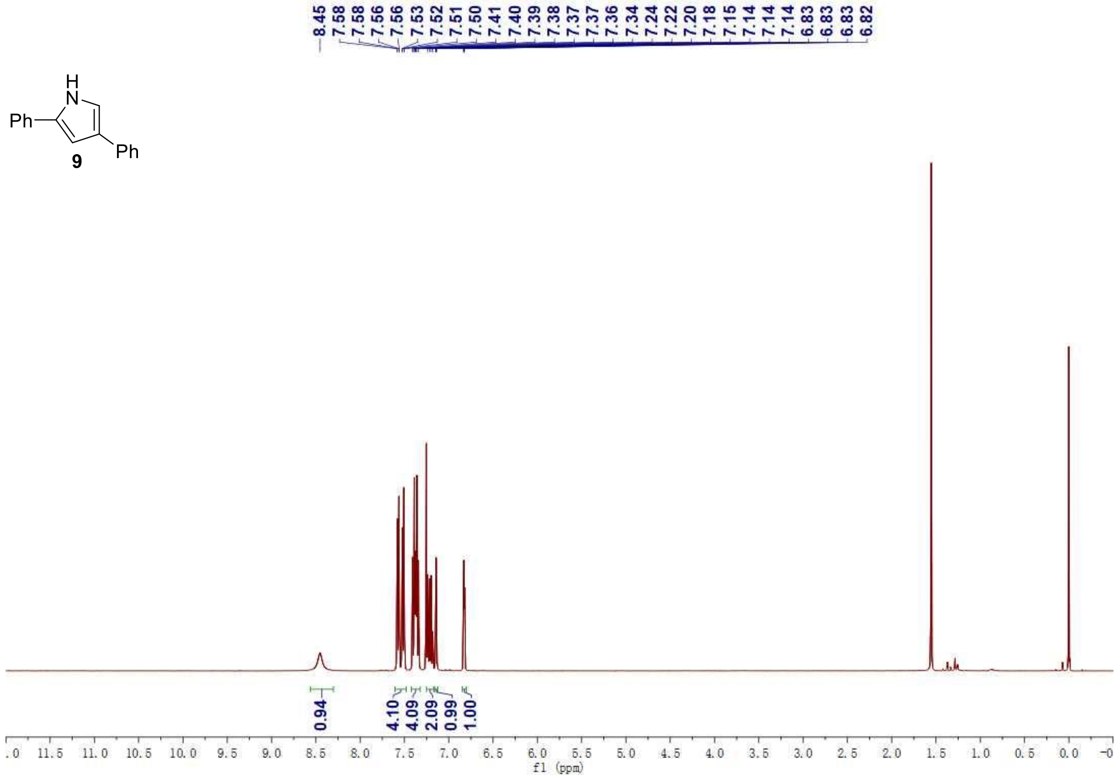

Supplementary Figure 123. ^{1}H NMR (400 MHz, $CDCl_3$) of **9**

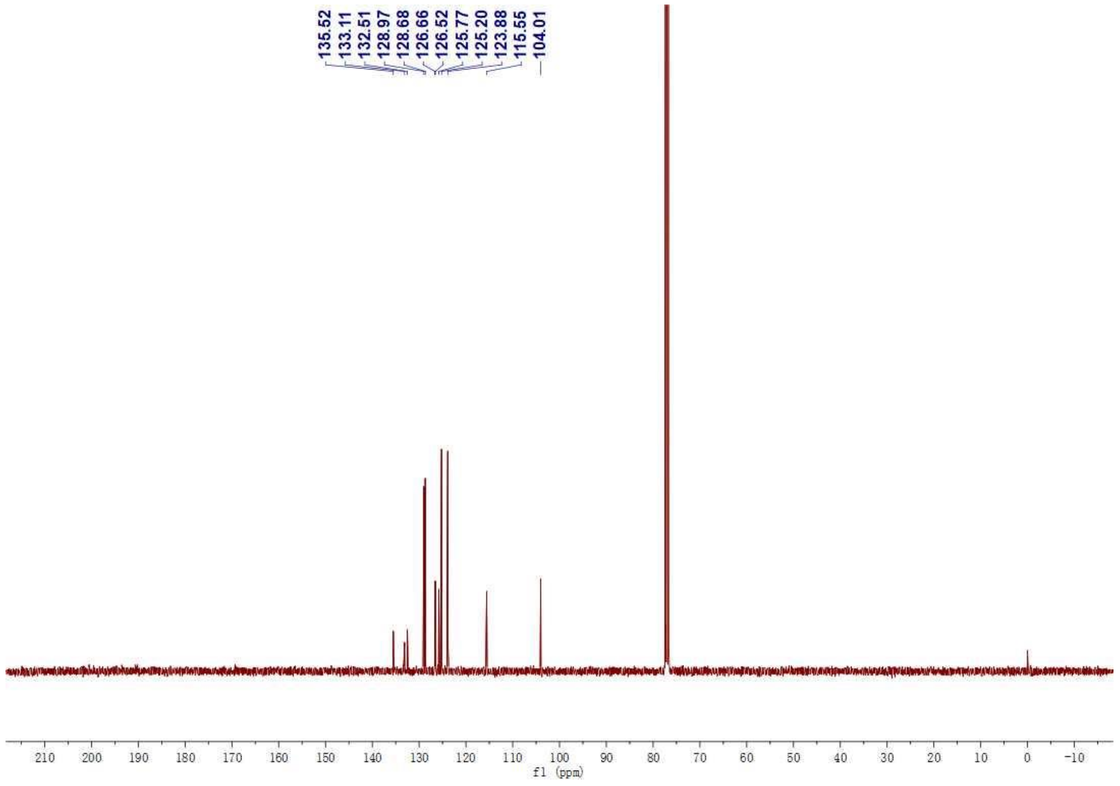

Supplementary Figure 124. ^{13}C NMR (101 MHz, $CDCl_3$) of **10**

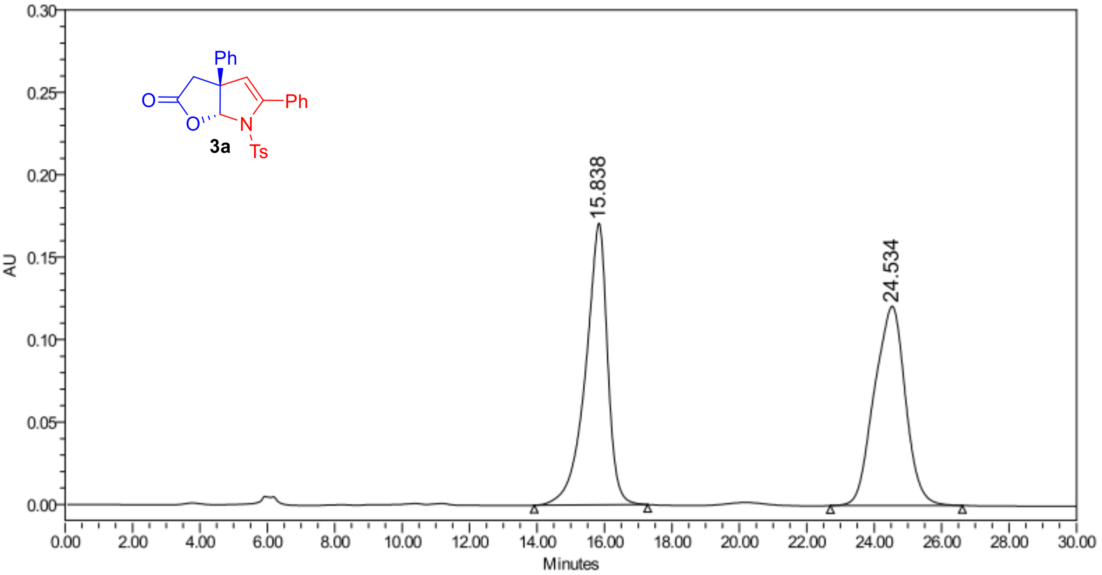

	RT	Area	% Area	Height
1	15.838	7734513	49.87	170835
2	24.534	7774075	50.13	120954

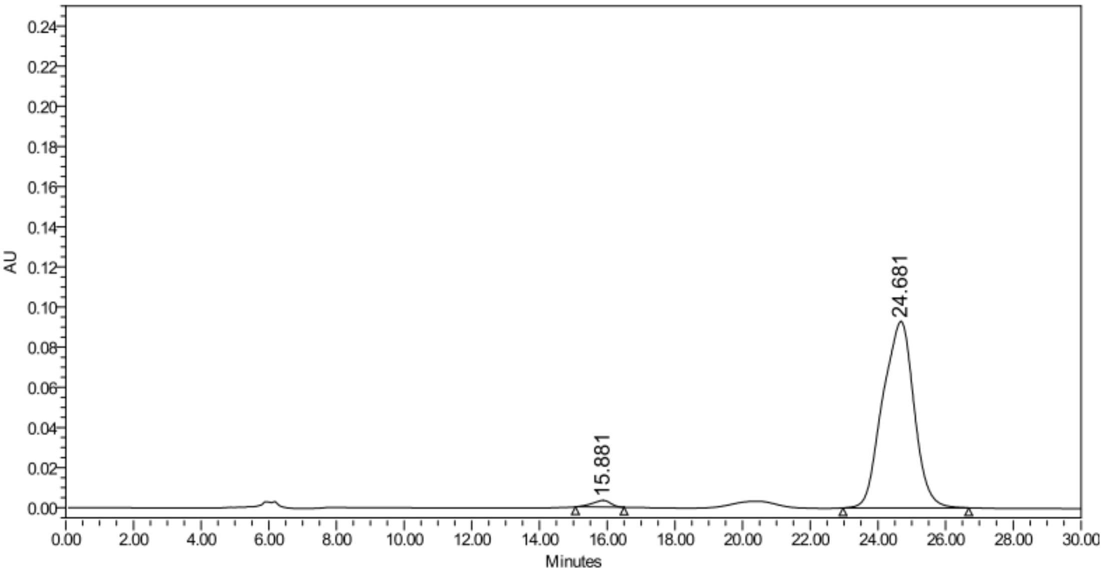

	RT	Area	% Area	Height
1	15.881	125101	2.05	3311
2	24.681	5985460	97.95	92935

Supplementary Figure 125. HPLC spectra of **3a**

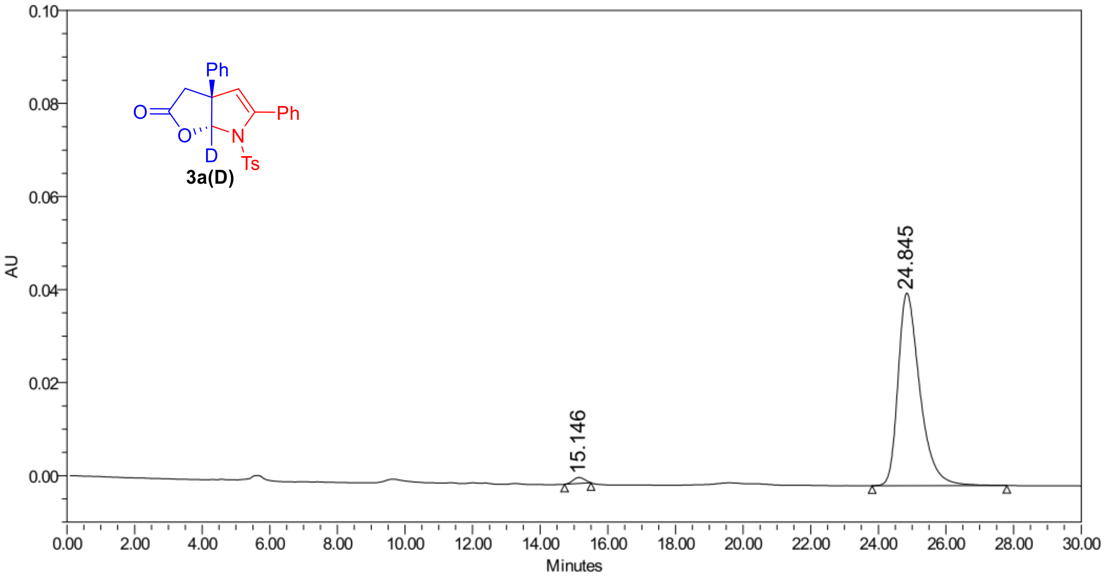

	RT	Area	% Area	Height
1	15.146	30742	1.63	1298
2	24.845	1856934	98.37	41377

Supplementary Figure 126. HPLC spectra of **3a(D)**

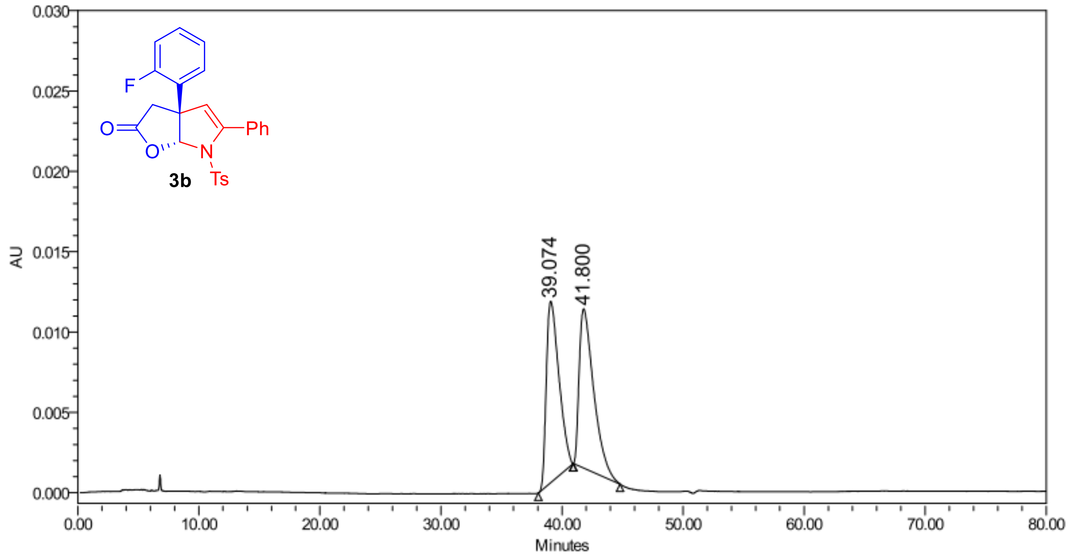

	RT	Area	% Area	Height
1	39.074	816886	49.66	11267
2	41.800	828020	50.34	9912

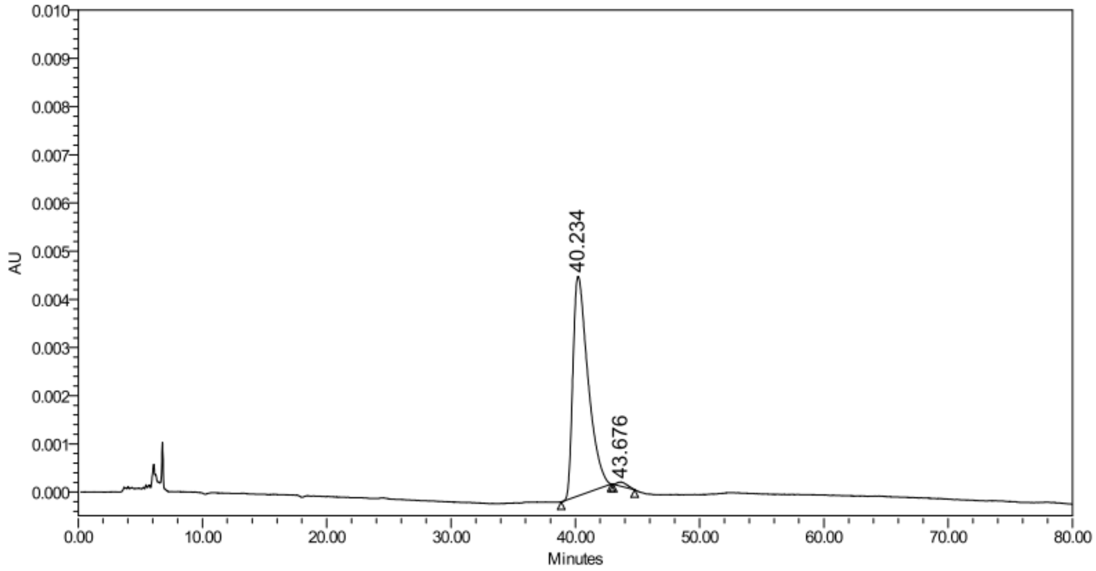

	RT	Area	% Area	Height
1	40.234	386989	98.75	4557
2	43.676	4905	1.25	86

Supplementary Figure 127. HPLC spectra of **3b**

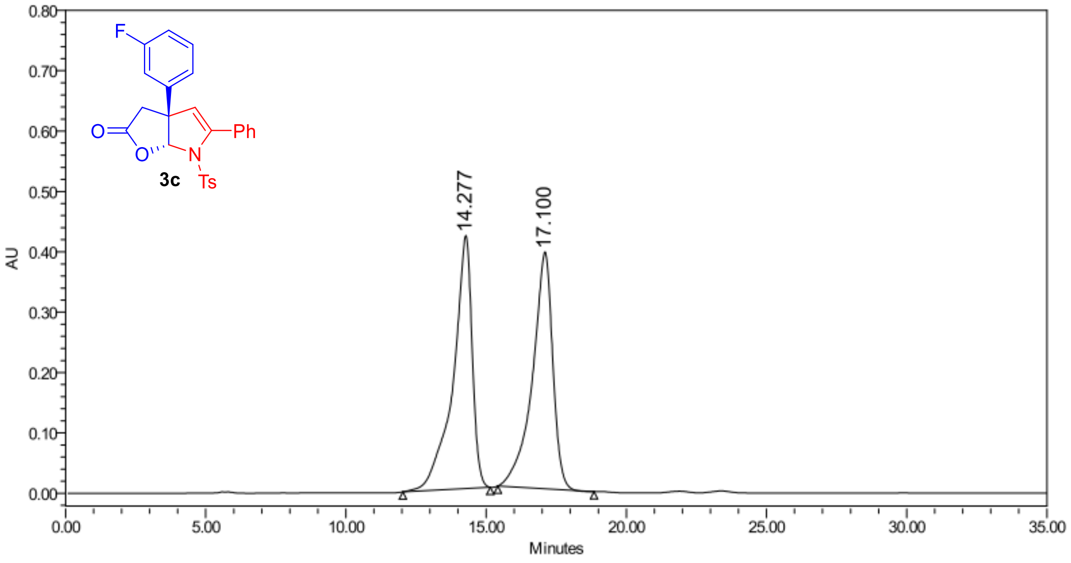

	RT	Area	% Area	Height
1	14.277	19730508	49.90	419373
2	17.100	19810923	50.10	392038

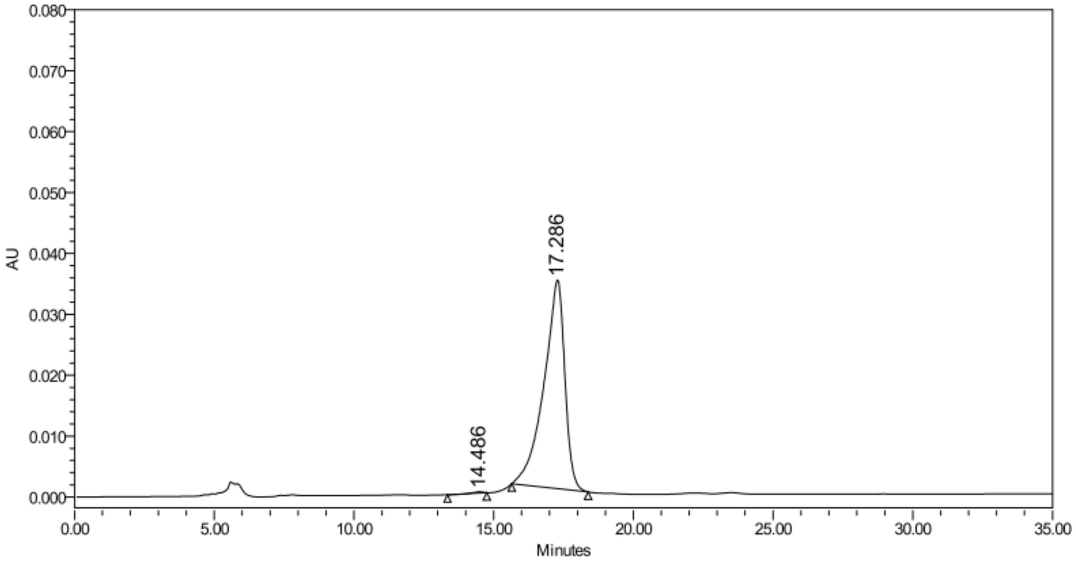

	RT	Area	% Area	Height
1	14.486	5803	0.32	212
2	17.286	1782750	99.68	34257

Supplementary Figure 128. HPLC spectra of **3c**

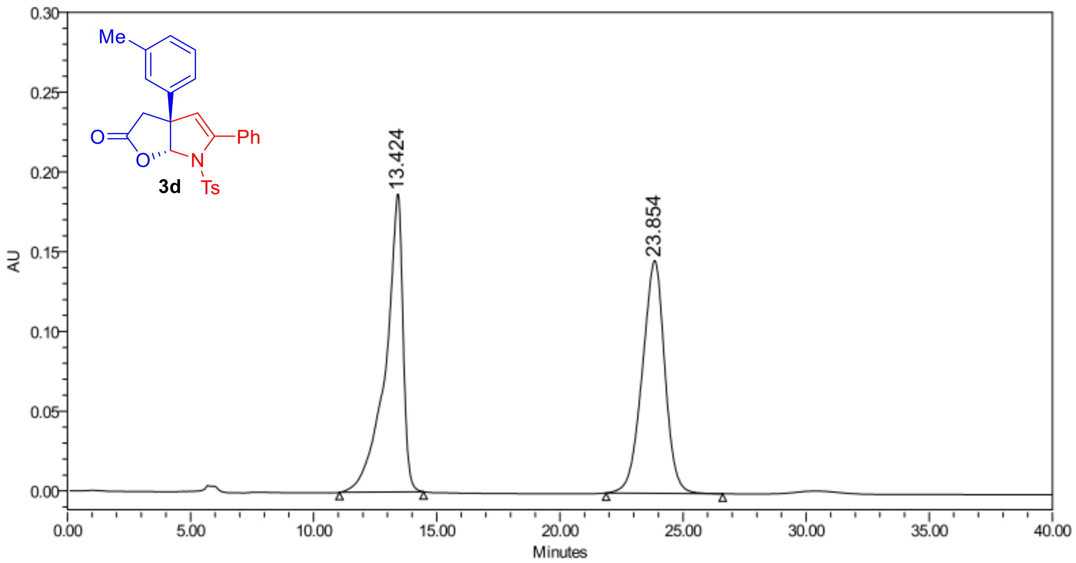

	RT	Area	% Area	Height
1	13.424	9205549	49.82	186823
2	23.854	9271890	50.18	145873

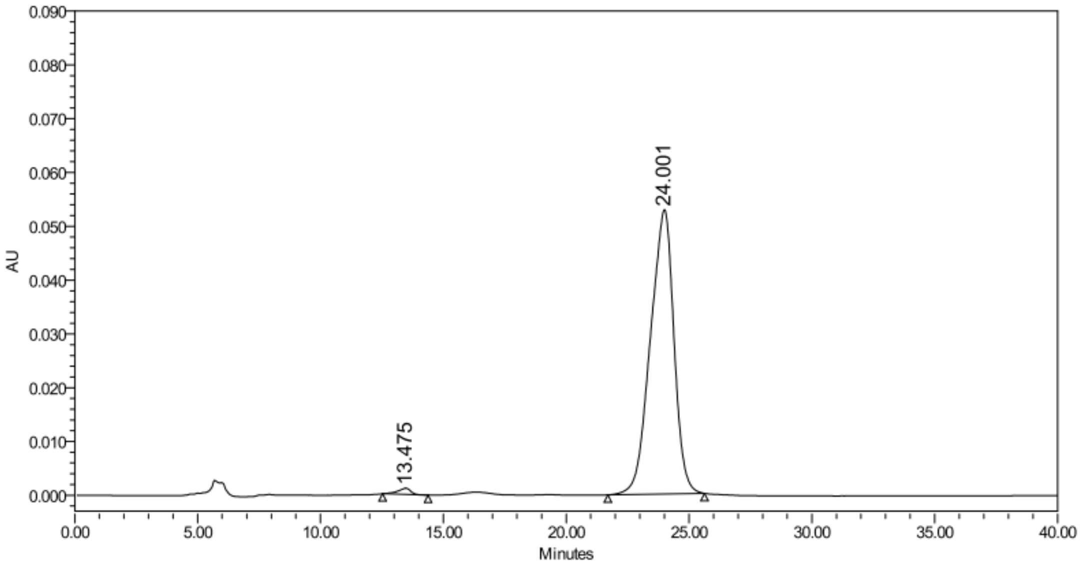

	RT	Area	% Area	Height
1	13.475	41915	1.18	1156
2	24.001	3514116	98.82	52839

Supplementary Figure 129. HPLC spectra of **3d**

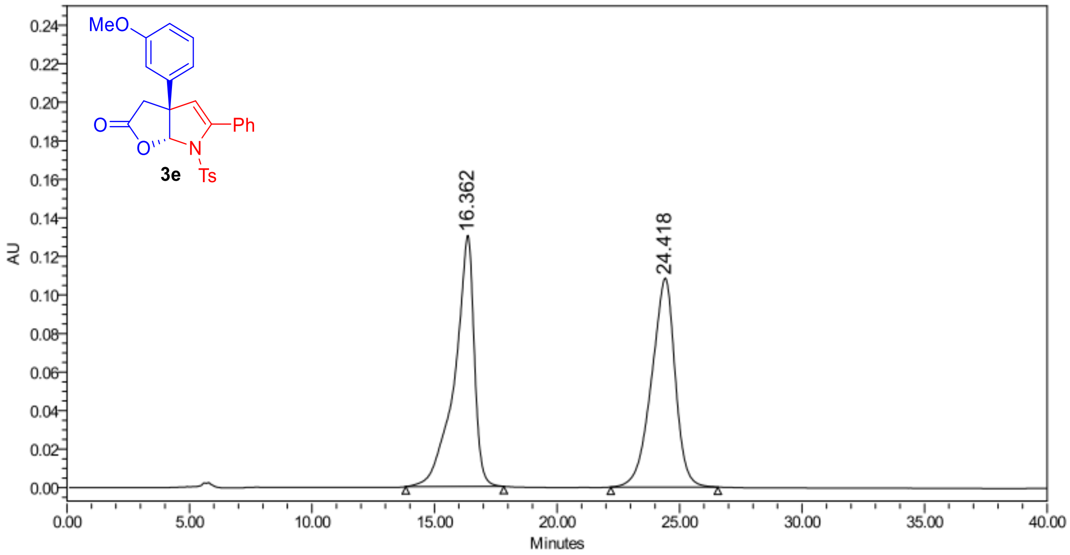

	RT	Area	% Area	Height
1	16.362	7243065	49.92	130372
2	24.418	7264843	50.08	108377

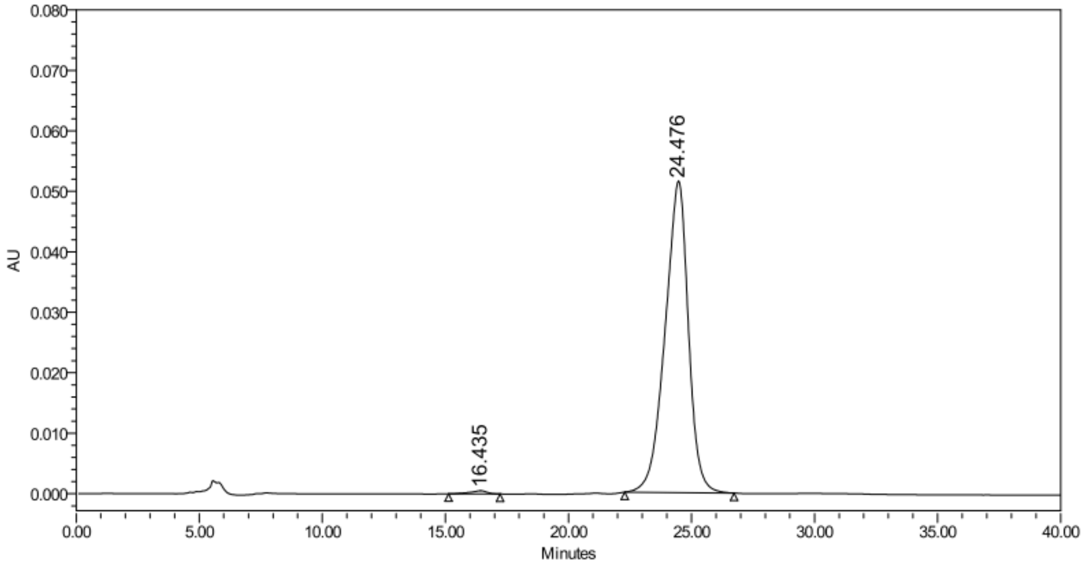

	RT	Area	% Area	Height
1	16.435	24809	0.73	516
2	24.476	3384306	99.27	51517

Supplementary Figure 130. HPLC spectra of **3e**

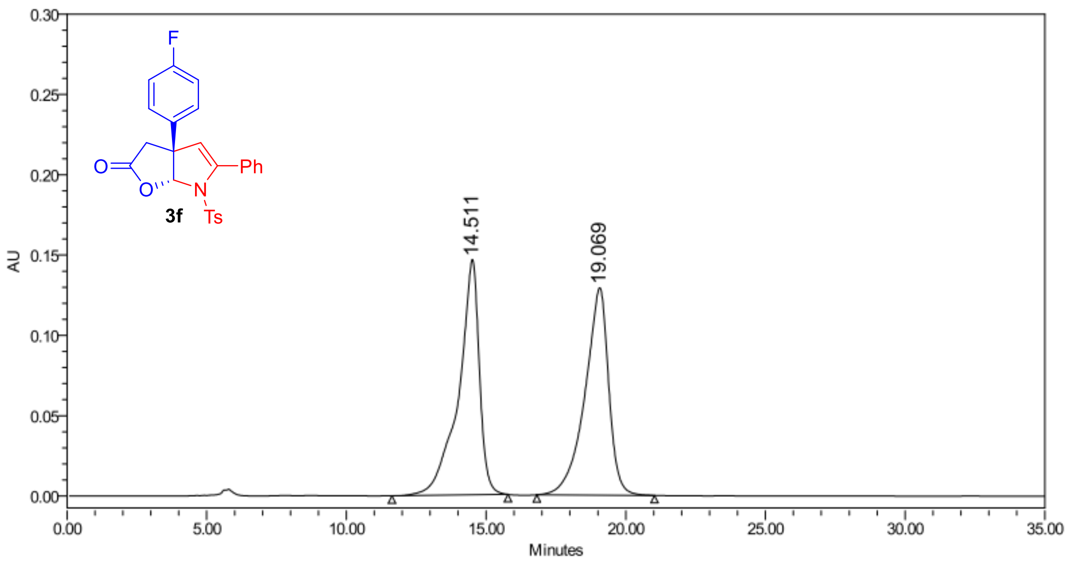

	RT	Area	% Area	Height
1	14.511	7592560	49.88	146480
2	19.069	7629687	50.12	129196

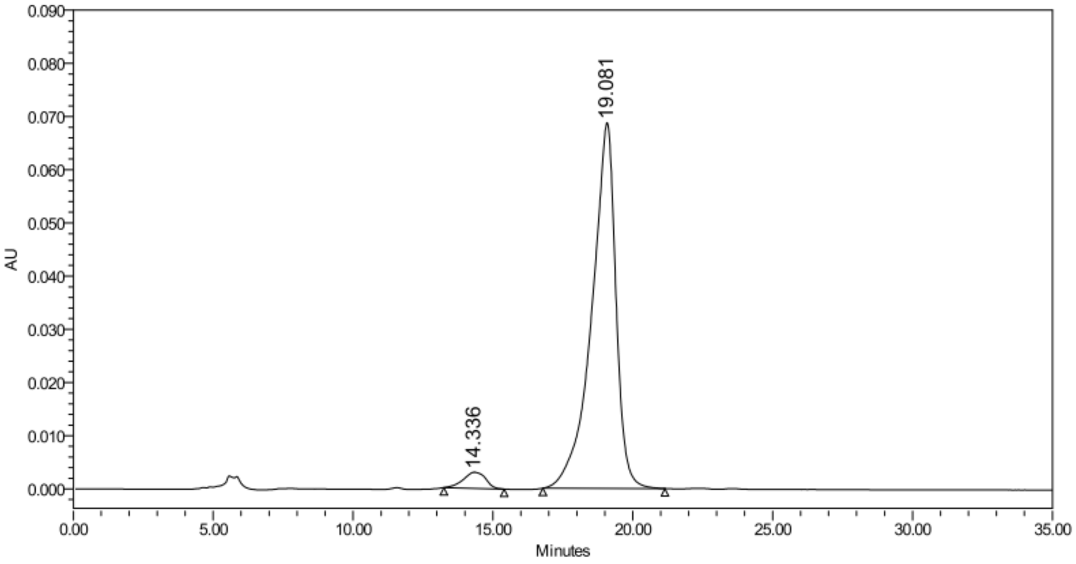

	RT	Area	% Area	Height
1	14.336	166949	3.89	3018
2	19.081	4128423	96.11	68770

Supplementary Figure 131. HPLC spectra of **3f**

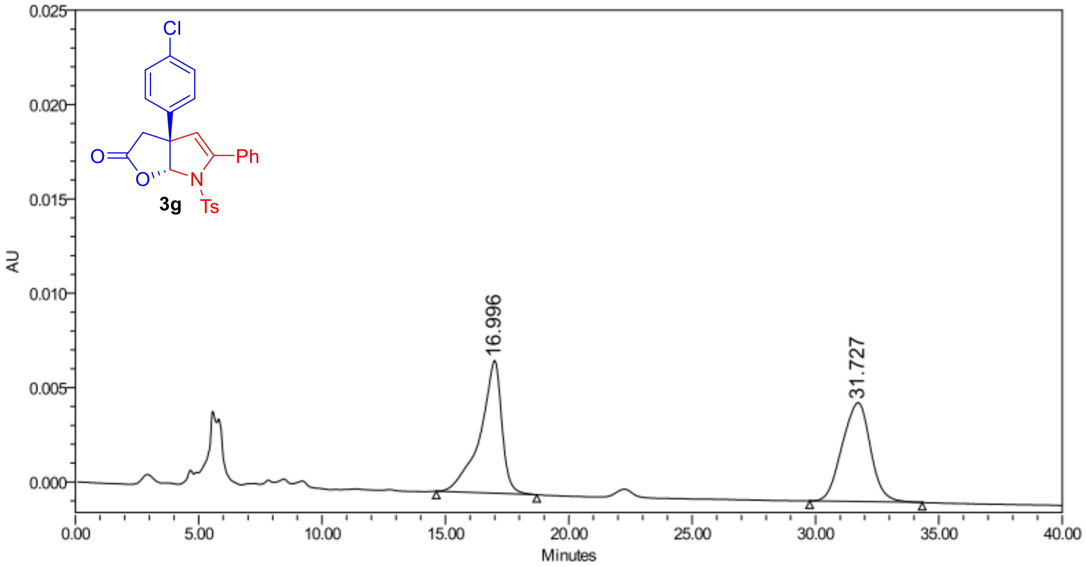

	RT	Area	% Area	Height
1	16.996	429615	50.65	7026
2	31.727	418629	49.35	5239

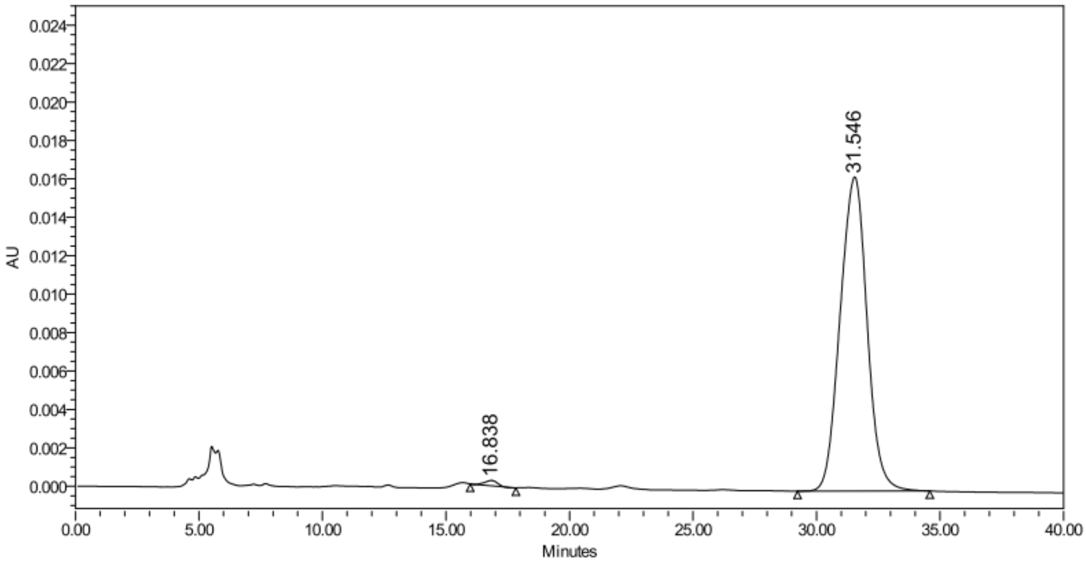

	RT	Area	% Area	Height
1	16.838	10316	0.83	278
2	31.546	1239371	99.17	16353

Supplementary Figure 132. HPLC spectra of **3g**

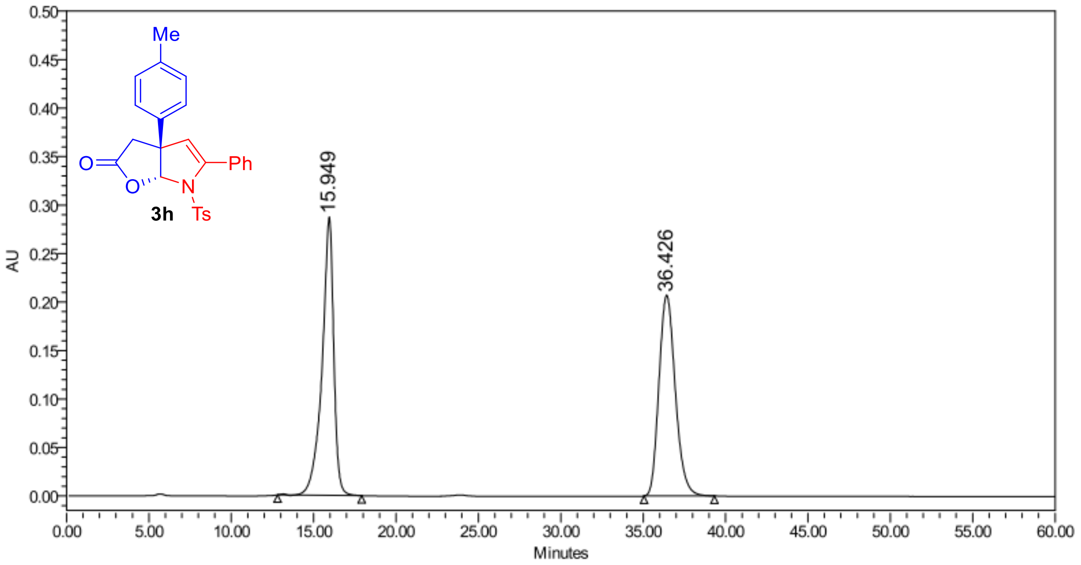

	RT	Area	% Area	Height
1	15.949	14170384	49.79	286886
2	36.426	14289352	50.21	206955

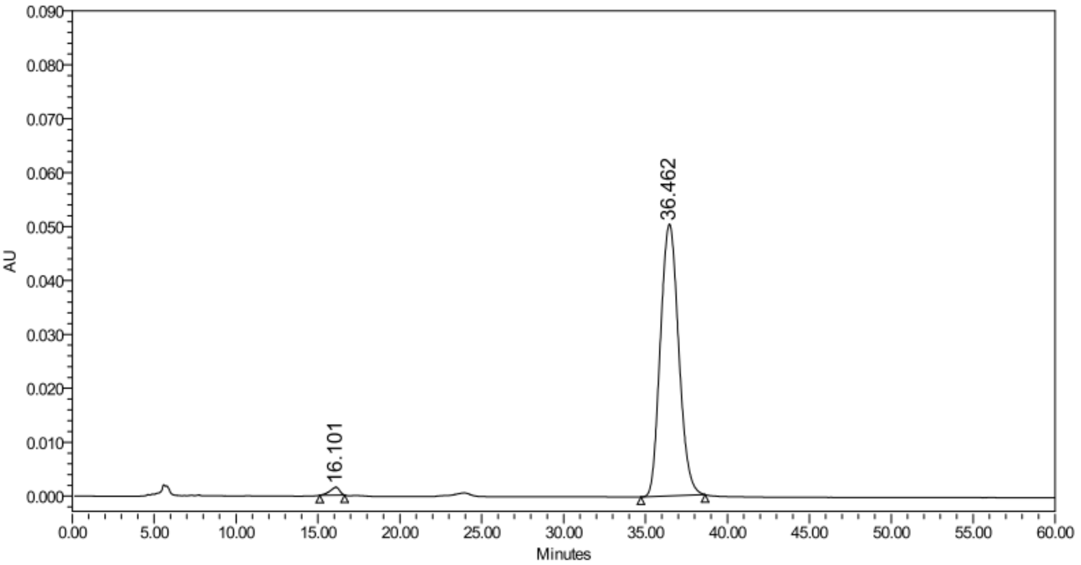

	RT	Area	% Area	Height
1	16.101	59766	1.51	1471
2	36.462	3909108	98.49	50411

Supplementary Figure 133. HPLC spectra of **3h**

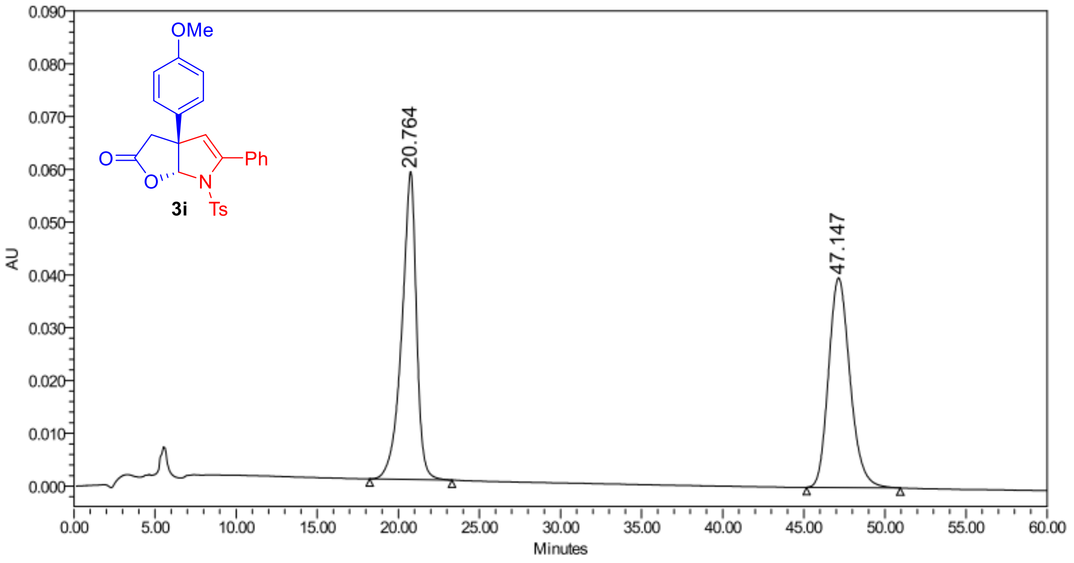

	RT	Area	% Area	Height
1	20.764	3673091	50.16	58320
2	47.147	3649515	49.84	39669

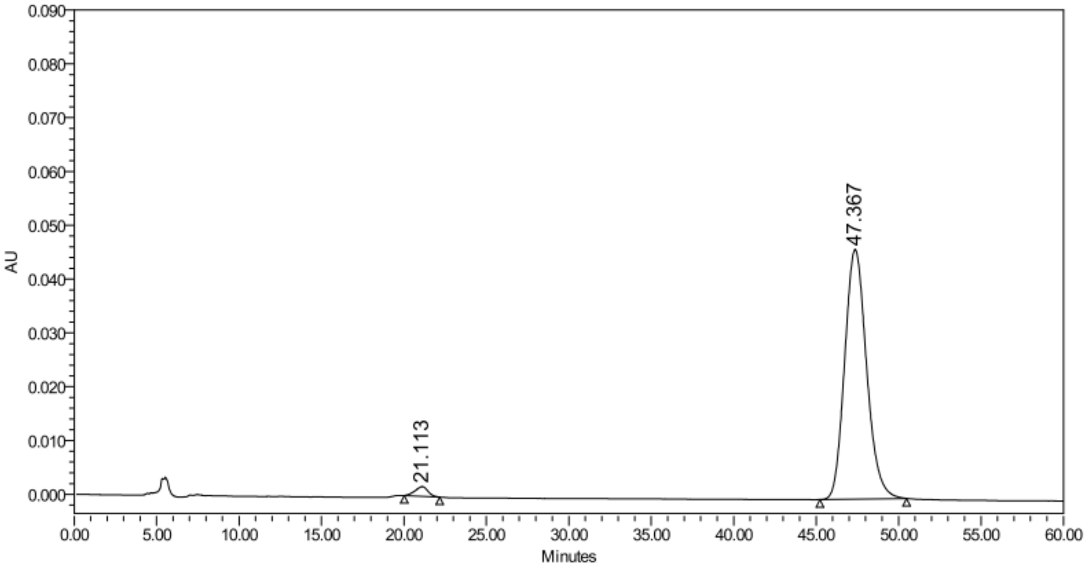

	RT	Area	% Area	Height
1	21.113	96574	2.20	1798
2	47.367	4284247	97.80	46416

Supplementary Figure 134. HPLC spectra of **3i**

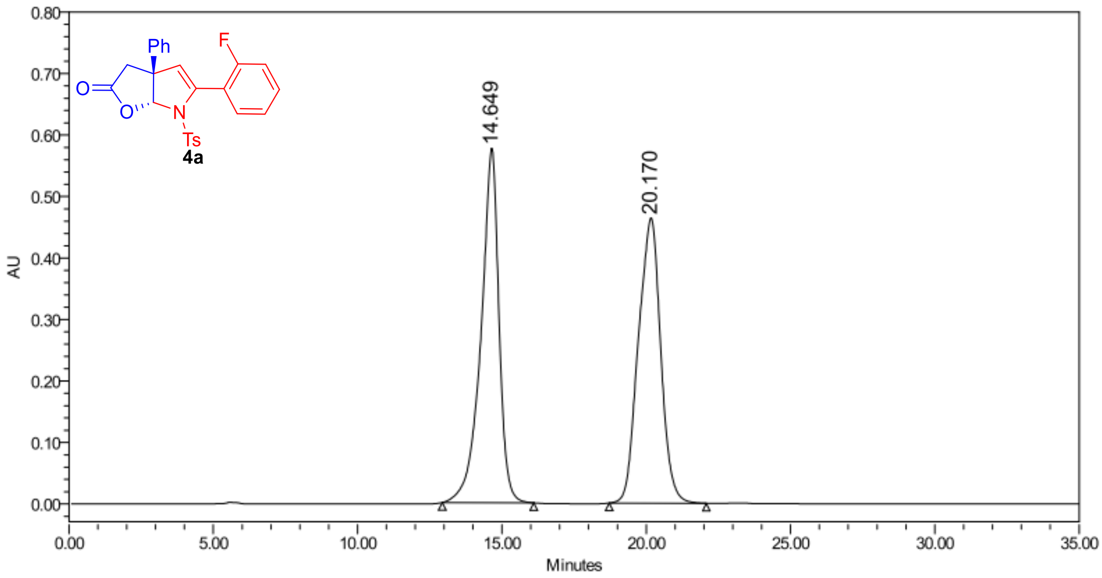

	RT	Area	% Area	Height
1	14.649	24984789	49.99	576428
2	20.170	24997457	50.01	463866

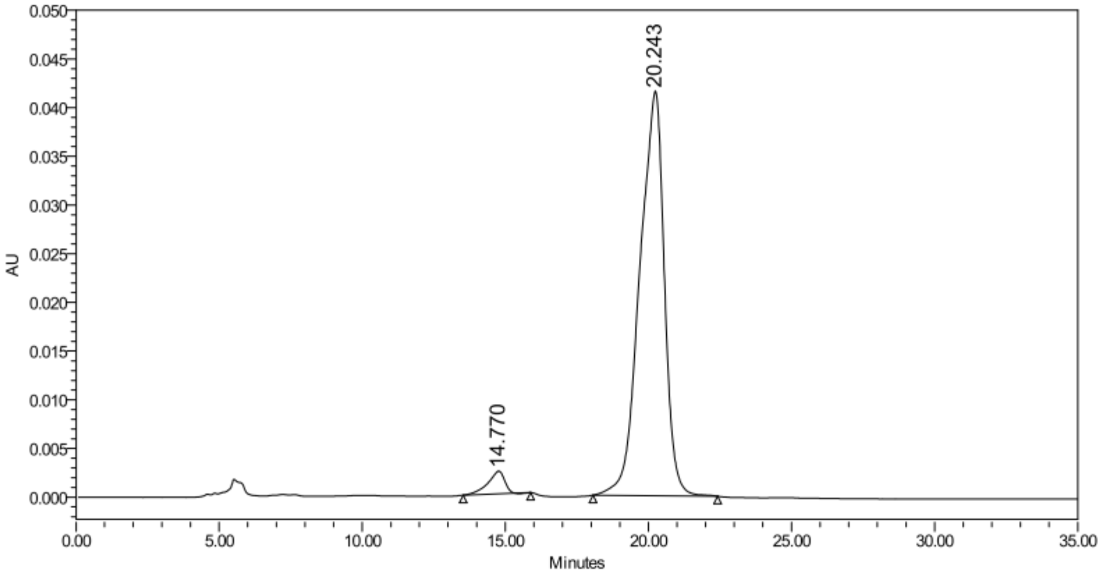

	RT	Area	% Area	Height
1	14.770	95663	3.72	2324
2	20.243	2477173	96.28	41539

Supplementary Figure 135. HPLC spectra of **4a**

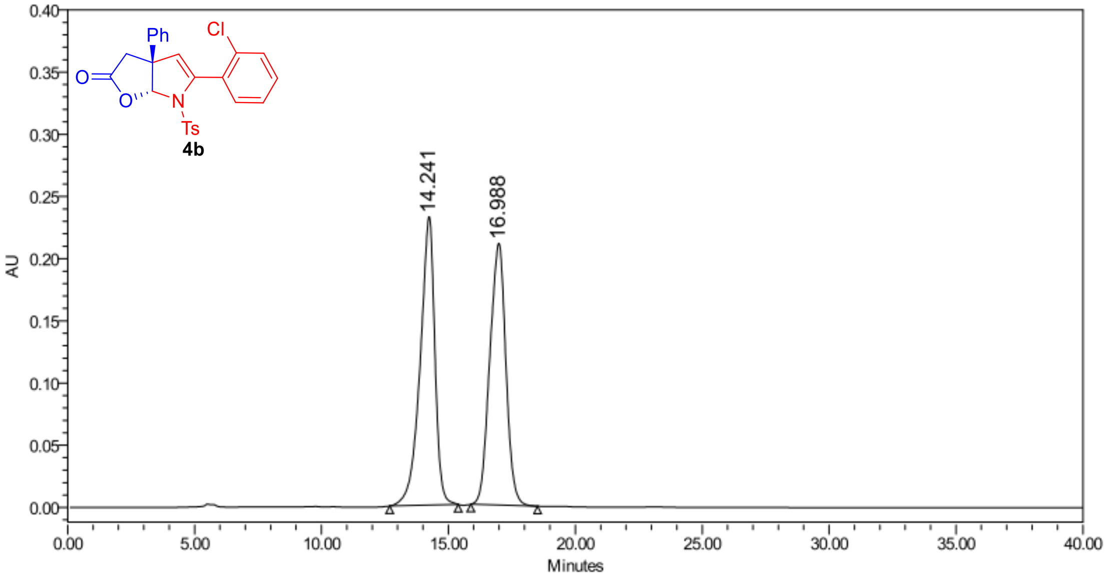

	RT	Area	% Area	Height
1	14.241	9337008	50.19	231946
2	16.988	9266489	49.81	210577

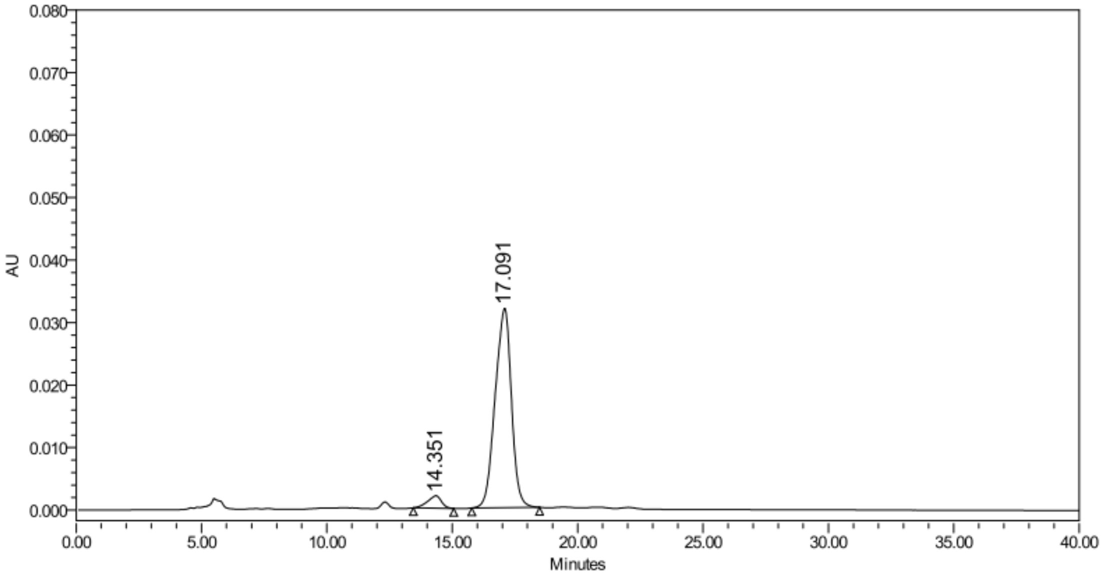

	RT	Area	% Area	Height
1	14.351	76353	5.04	1987
2	17.091	1439829	94.96	31923

Supplementary Figure 136. HPLC spectra of **4b**

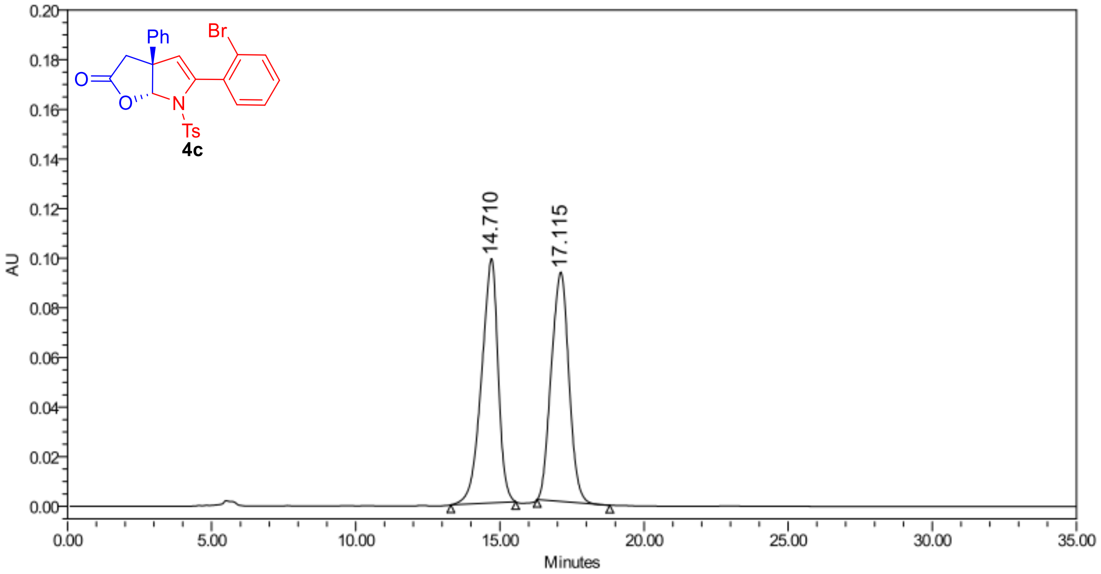

	RT	Area	% Area	Height
1	14.710	3943710	50.10	98510
2	17.115	3927355	49.90	92399

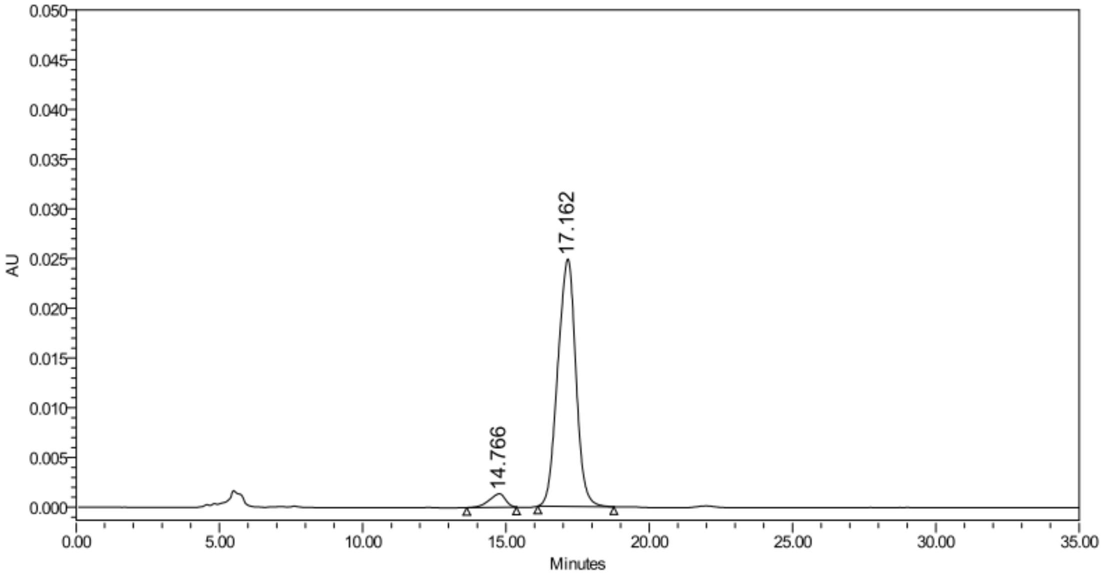

	RT	Area	% Area	Height
1	14.766	53616	4.67	1365
2	17.162	1093443	95.33	24897

Supplementary Figure 137. HPLC spectra of **4c**

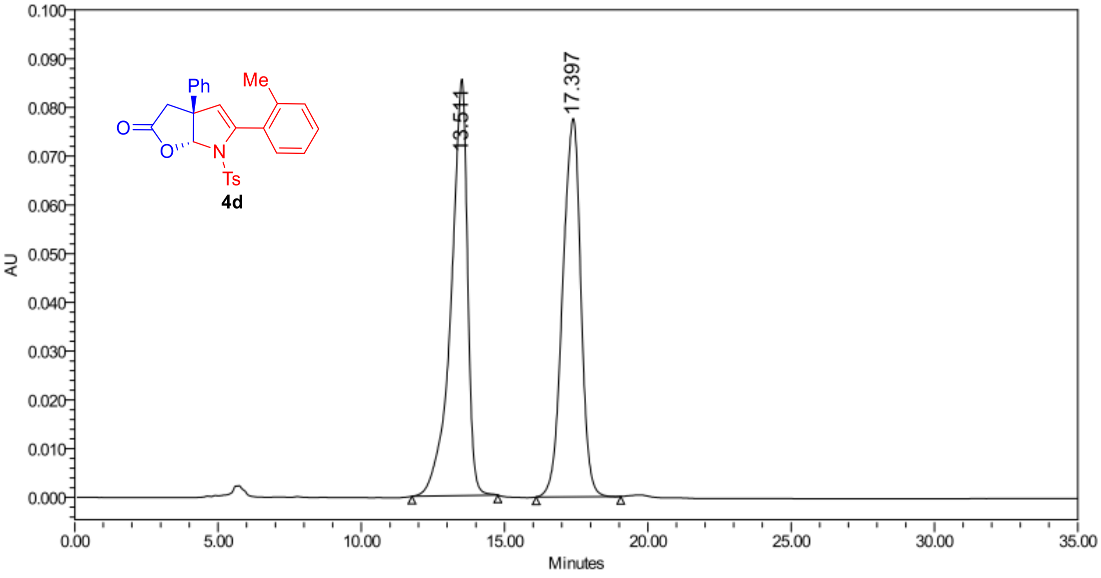

	RT	Area	% Area	Height
1	13.511	3420625	50.02	85437
2	17.397	3418264	49.98	77542

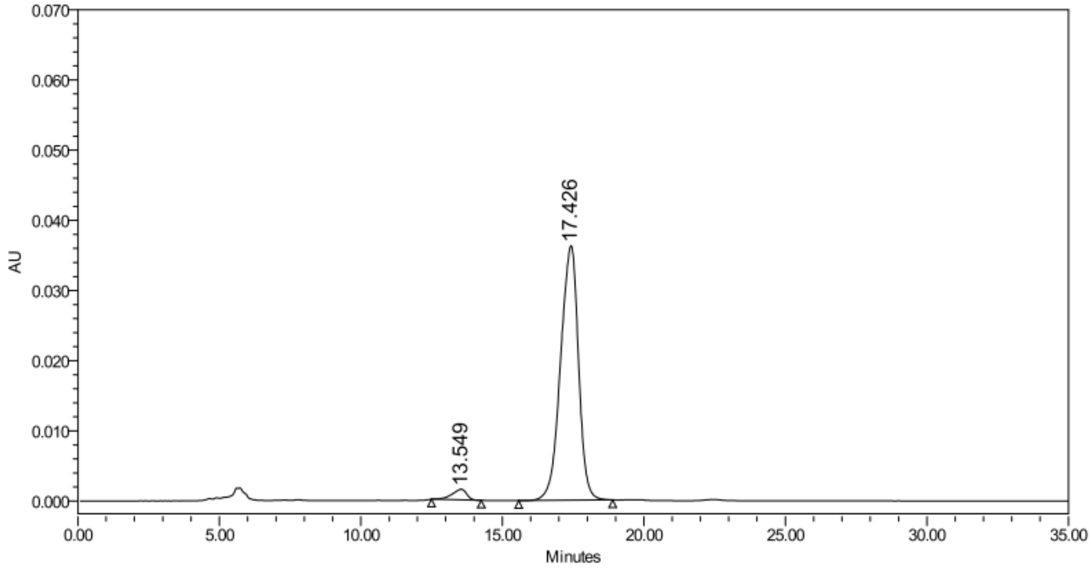

	RT	Area	% Area	Height
1	13.549	54873	3.31	1524
2	17.426	1602085	96.69	36281

Supplementary Figure 138. HPLC spectra of **4d**

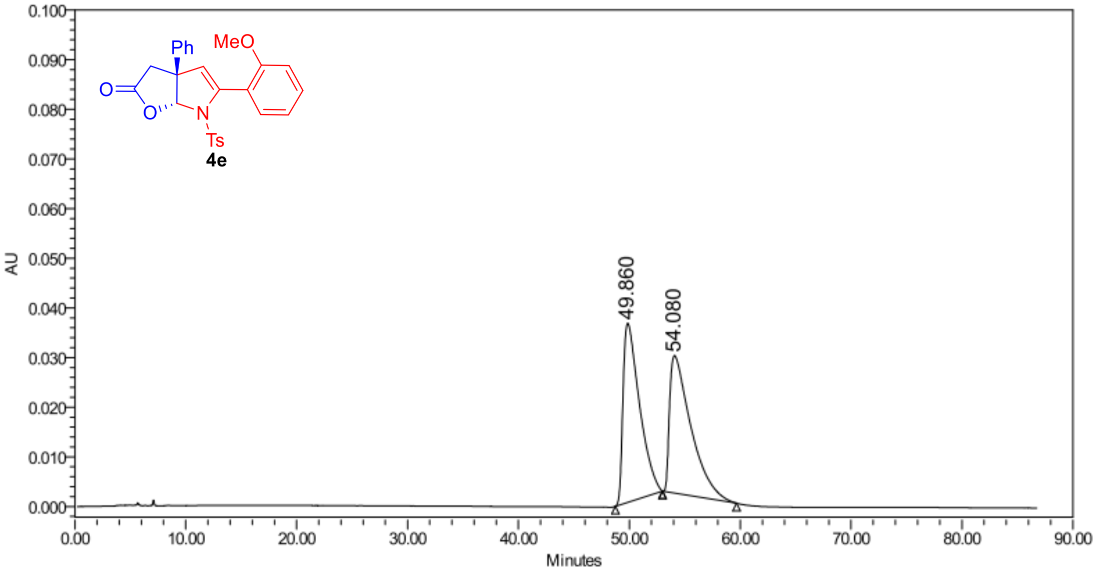

	RT	Area	% Area	Height
1	49.860	3729744	50.20	36058
2	54.080	3700153	49.80	27702

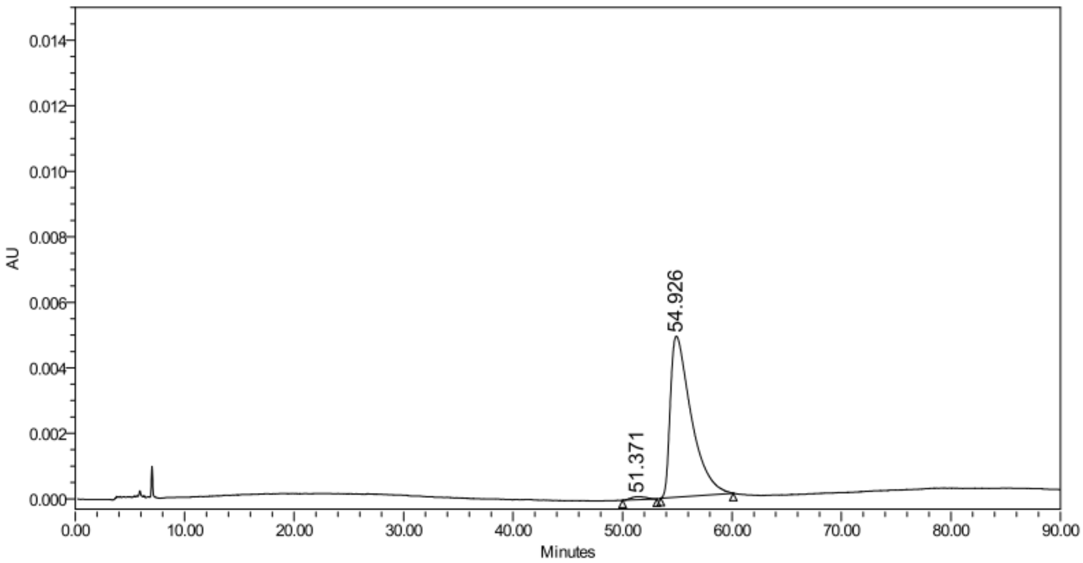

	RT	Area	% Area	Height
1	51.371	8877	1.34	94
2	54.926	653051	98.66	4918

Supplementary Figure 139. HPLC spectra of **4e**

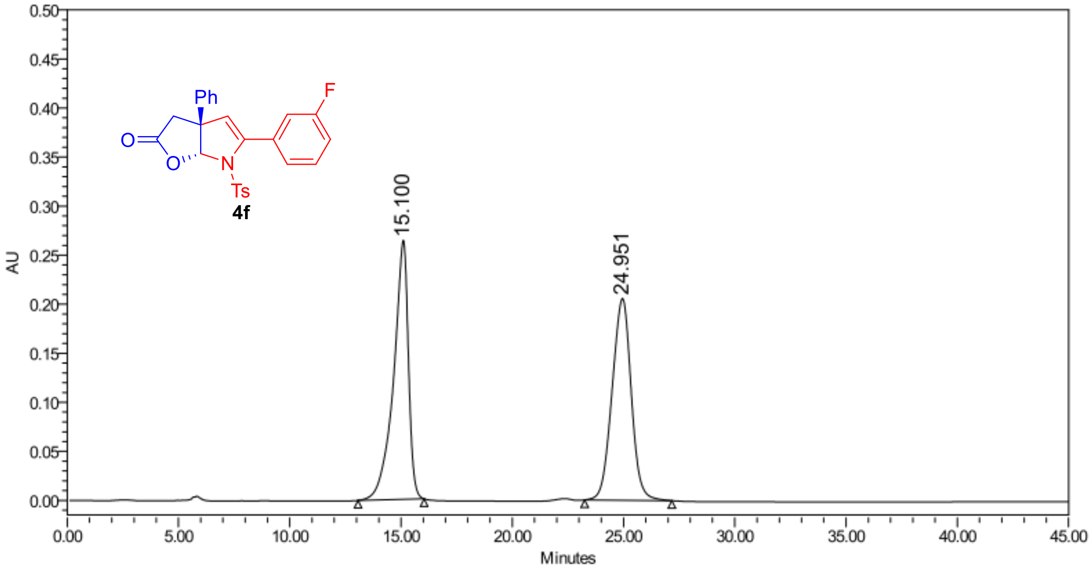

	RT	Area	% Area	Height
1	15.100	12236942	50.00	263816
2	24.951	12237005	50.00	205673

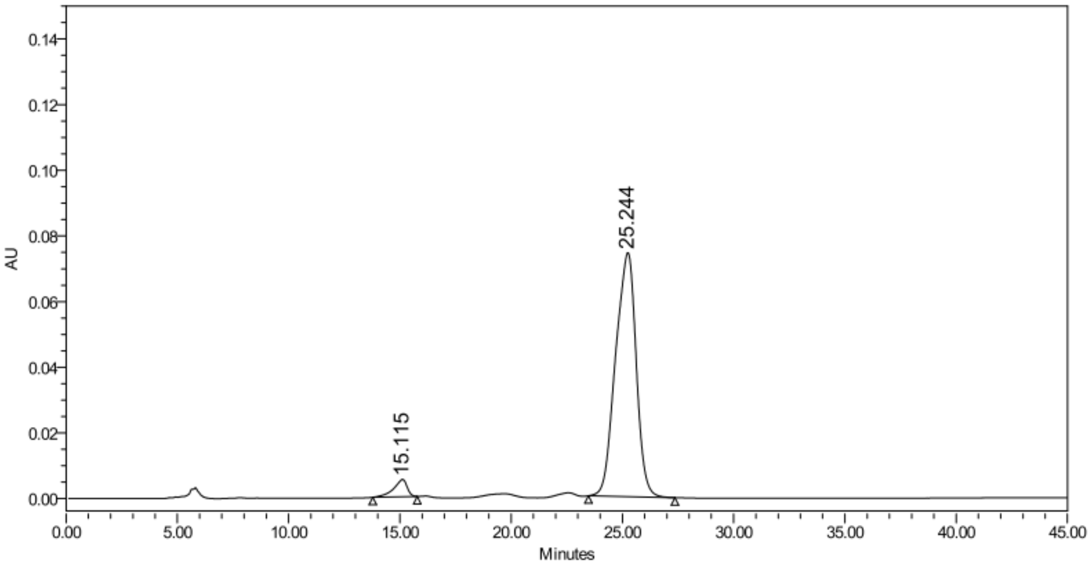

	RT	Area	% Area	Height
1	15.115	221367	4.43	5264
2	25.244	4773892	95.57	74341

Supplementary Figure 140. HPLC spectra of **4f**

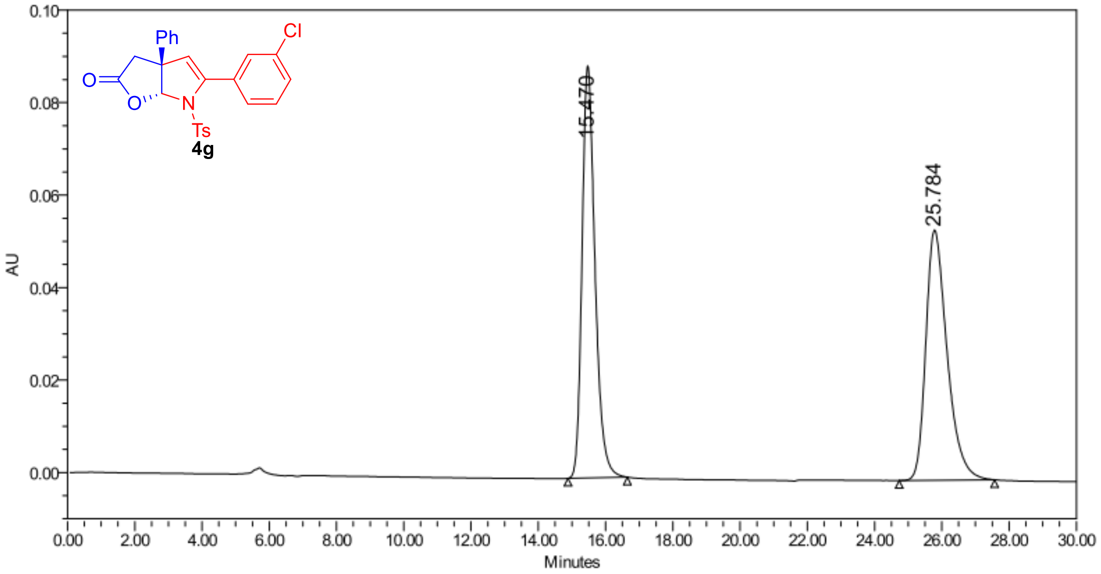

	RT	Area	% Area	Height
1	15.470	2383525	50.07	89104
2	25.784	2376607	49.93	54075

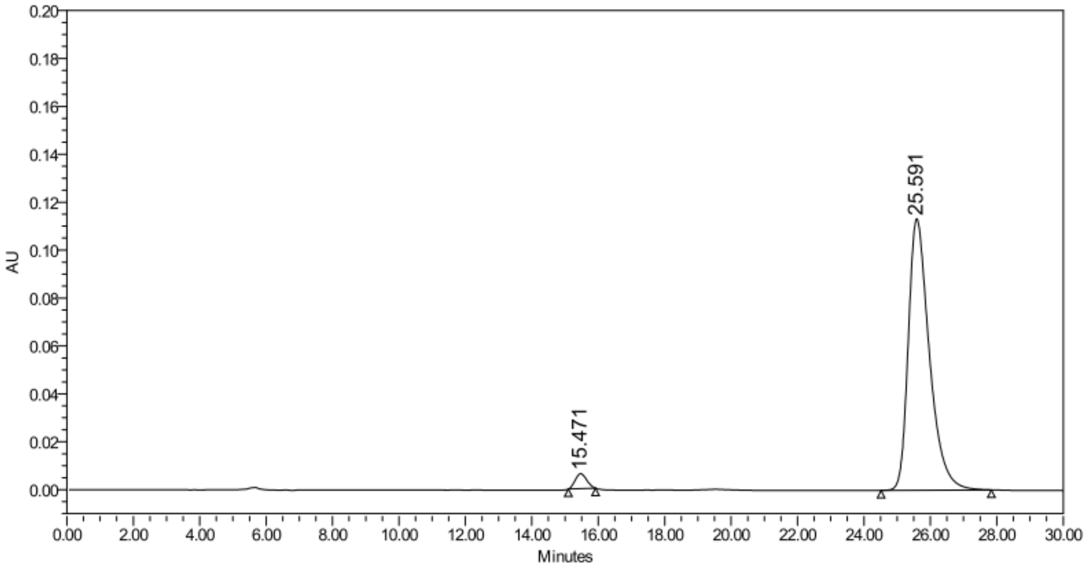

	RT	Area	% Area	Height
1	15.471	149269	2.91	6245
2	25.591	4976505	97.09	113247

Supplementary Figure 141. HPLC spectra of **4g**

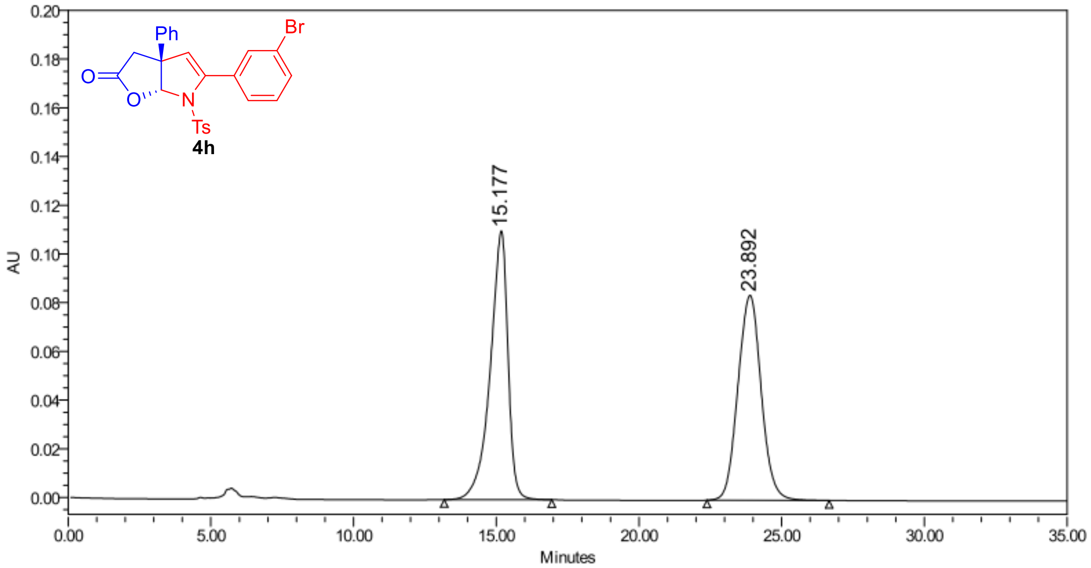

	RT	Area	% Area	Height
1	15.177	4764080	49.91	110369
2	23.892	4780399	50.09	84206

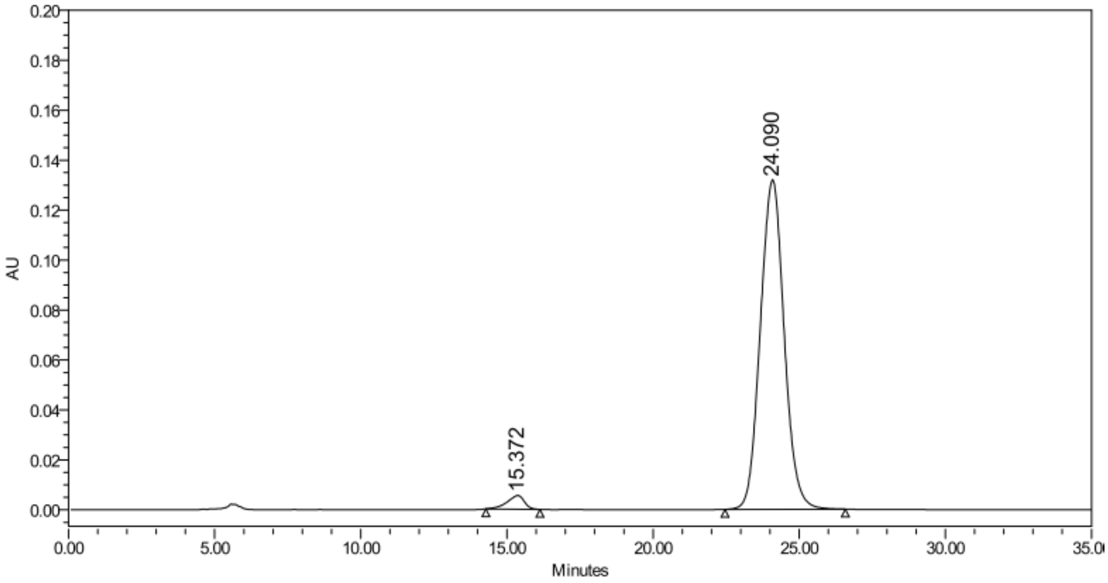

	RT	Area	% Area	Height
1	15.372	225813	2.93	5496
2	24.090	7480847	97.07	131908

Supplementary Figure 142. HPLC spectra of **4h**

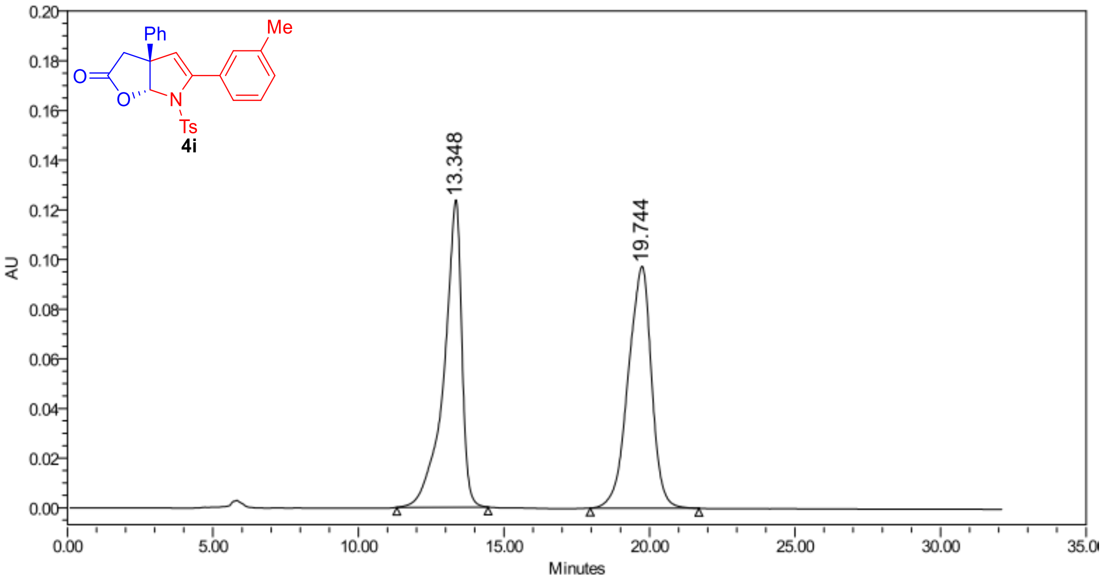

	RT	Area	% Area	Height
1	13.348	5264616	49.16	123756
2	19.744	5445452	50.84	97406

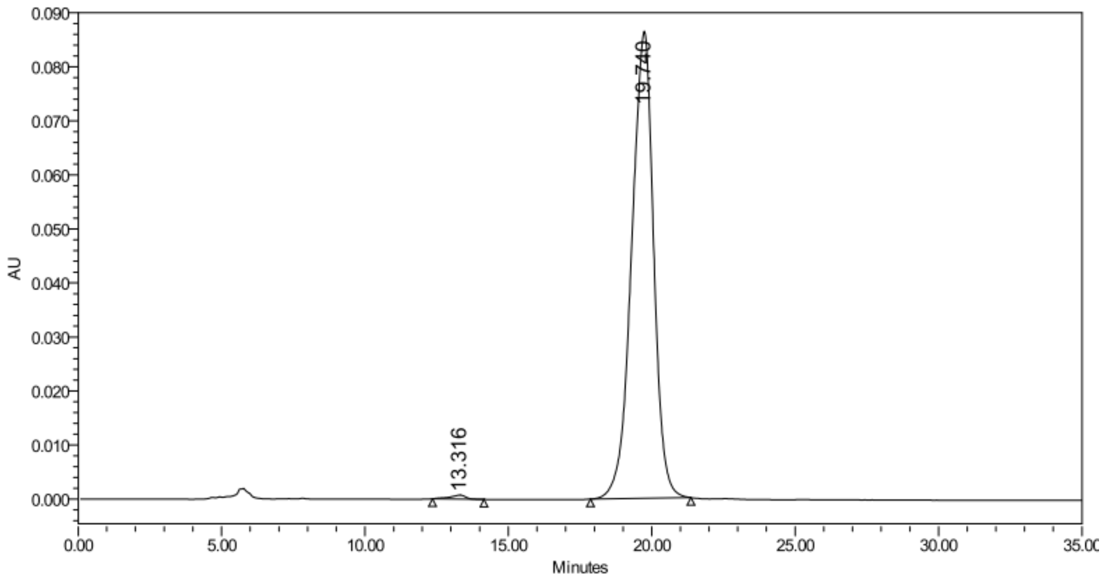

	RT	Area	% Area	Height
1	13.316	28203	0.61	733
2	19.740	4607930	99.39	86424

Supplementary Figure 143. HPLC spectra of **4i**

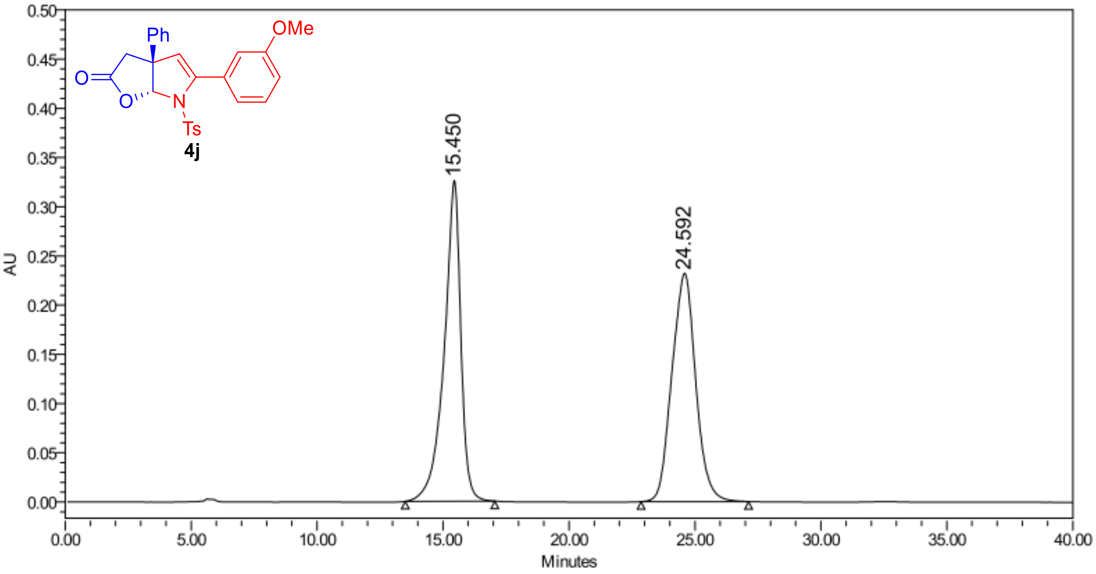

	RT	Area	% Area	Height
1	15.450	14984154	49.59	325739
2	24.592	15229458	50.41	231972

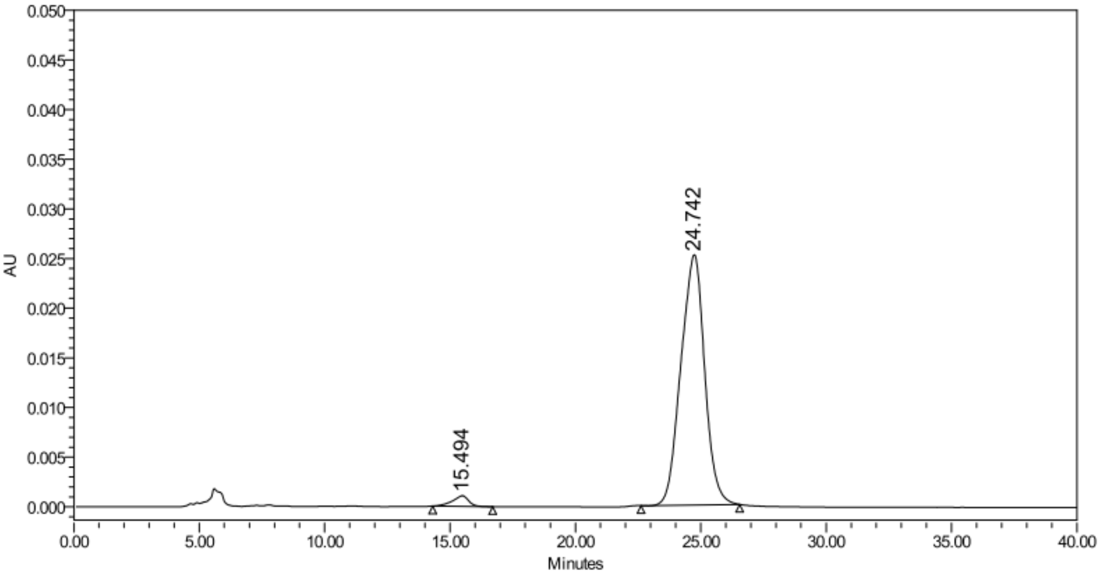

	RT	Area	% Area	Height
1	15.494	47581	2.78	1070
2	24.742	1662272	97.22	25211

Supplementary Figure 144. HPLC spectra of **4j**

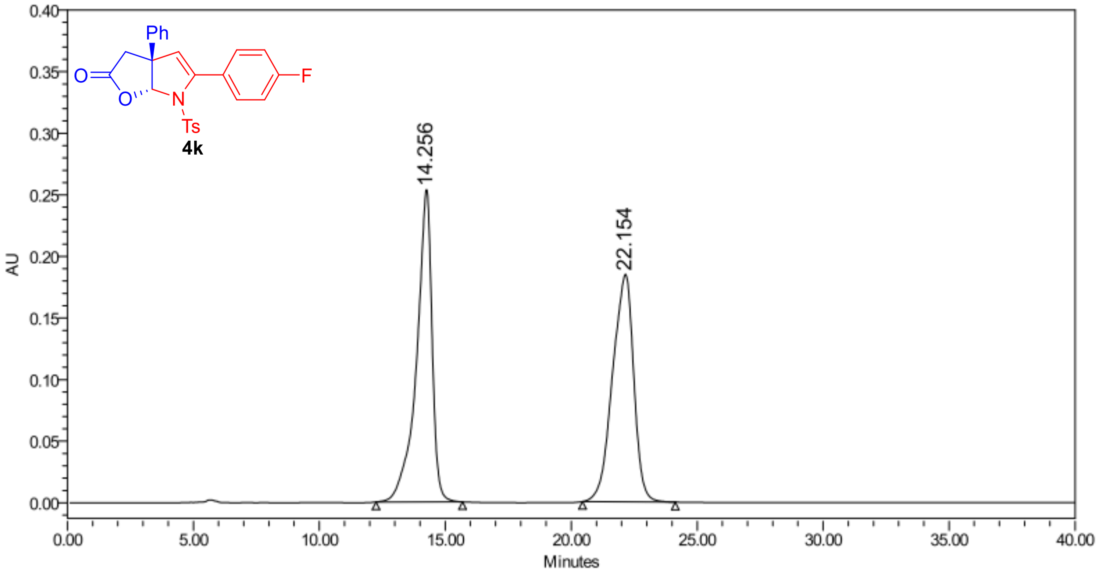

	RT	Area	% Area	Height
1	14.256	10843860	50.04	253576
2	22.154	10828550	49.96	184684

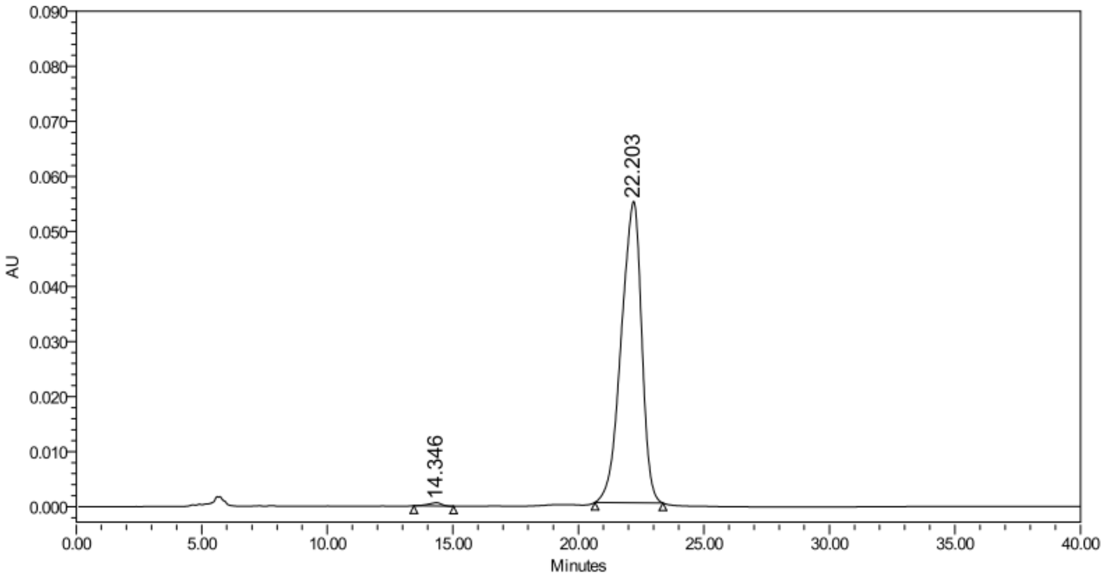

	RT	Area	% Area	Height
1	14.346	22424	0.72	605
2	22.203	3085130	99.28	54715

Supplementary Figure 145. HPLC spectra of **4k**

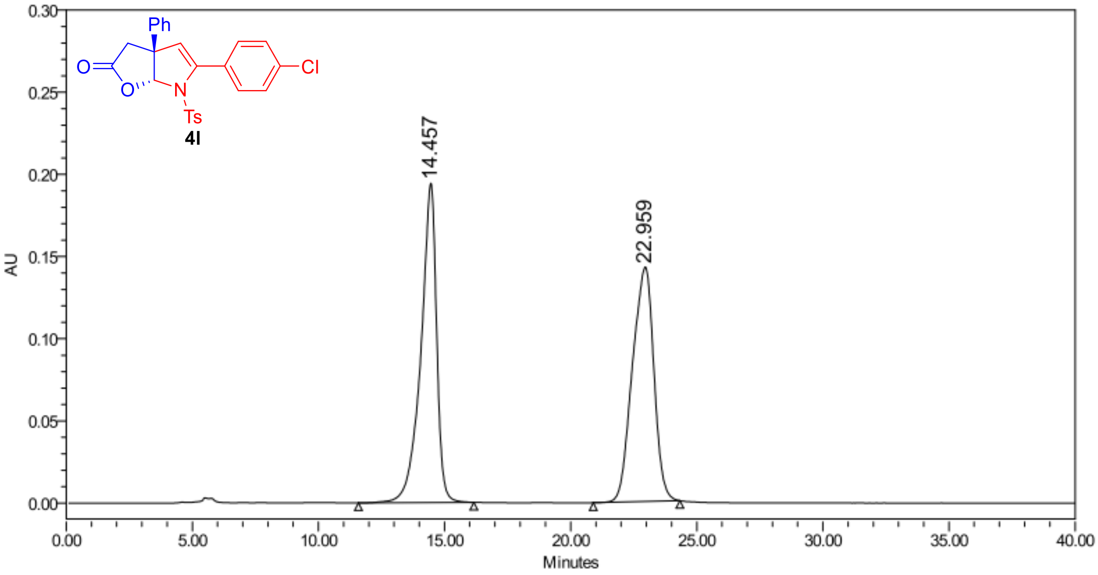

	RT	Area	% Area	Height
1	14.457	8438975	49.94	194461
2	22.959	8458434	50.06	142689

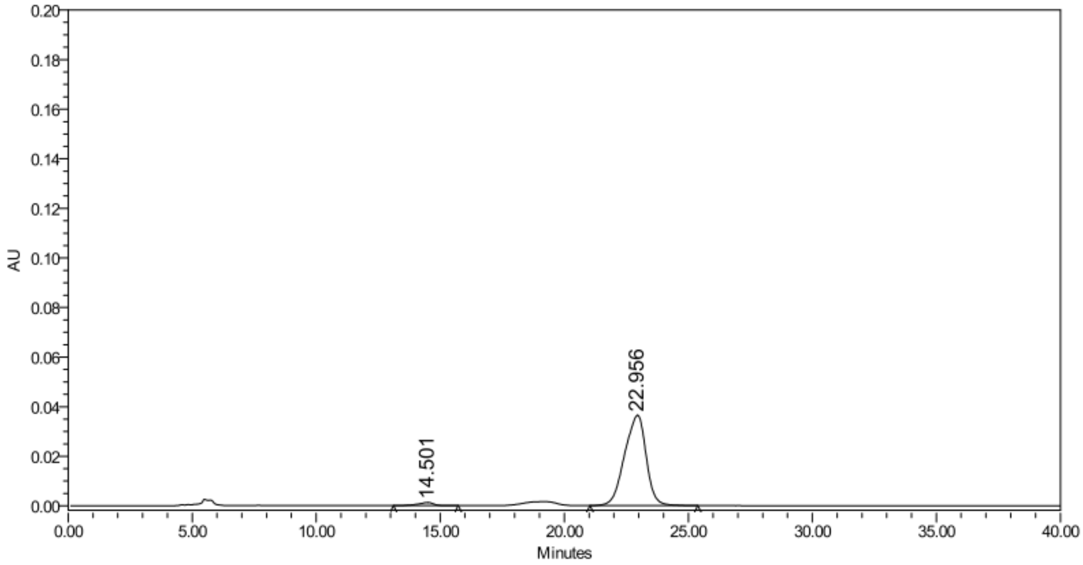

	RT	Area	% Area	Height
1	14.501	52913	2.35	1247
2	22.956	2202147	97.65	36432

Supplementary Figure 146. HPLC spectra of **4l**

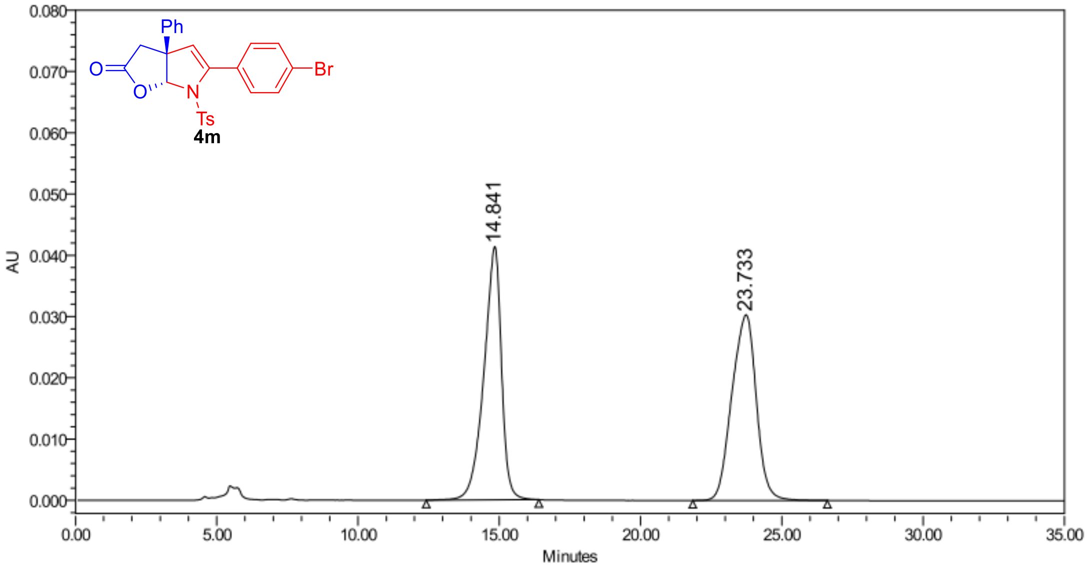

	RT	Area	% Area	Height
1	14.841	1784236	49.46	41342
2	23.733	1823003	50.54	30314

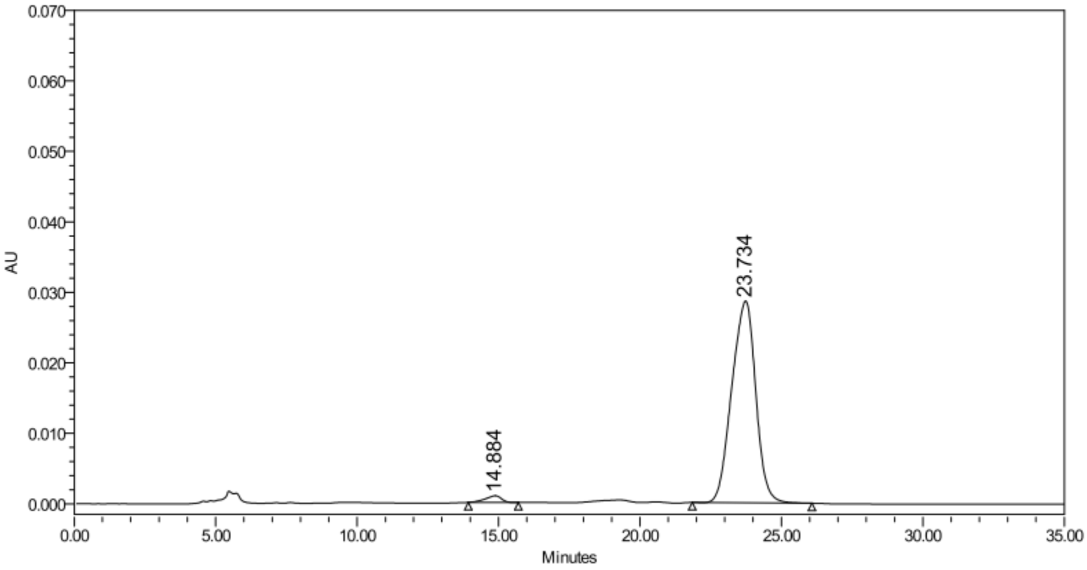

	RT	Area	% Area	Height
1	14.884	37132	2.15	946
2	23.734	1687759	97.85	28632

Supplementary Figure 147. HPLC spectra of **4m**

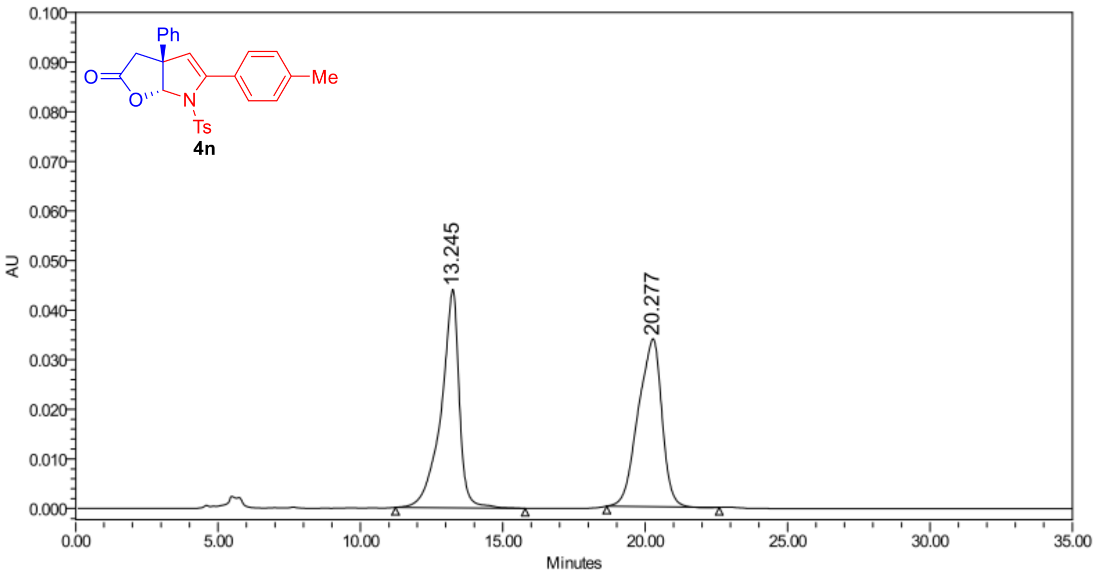

	RT	Area	% Area	Height
1	13.245	1942361	49.77	44006
2	20.277	1960426	50.23	33826

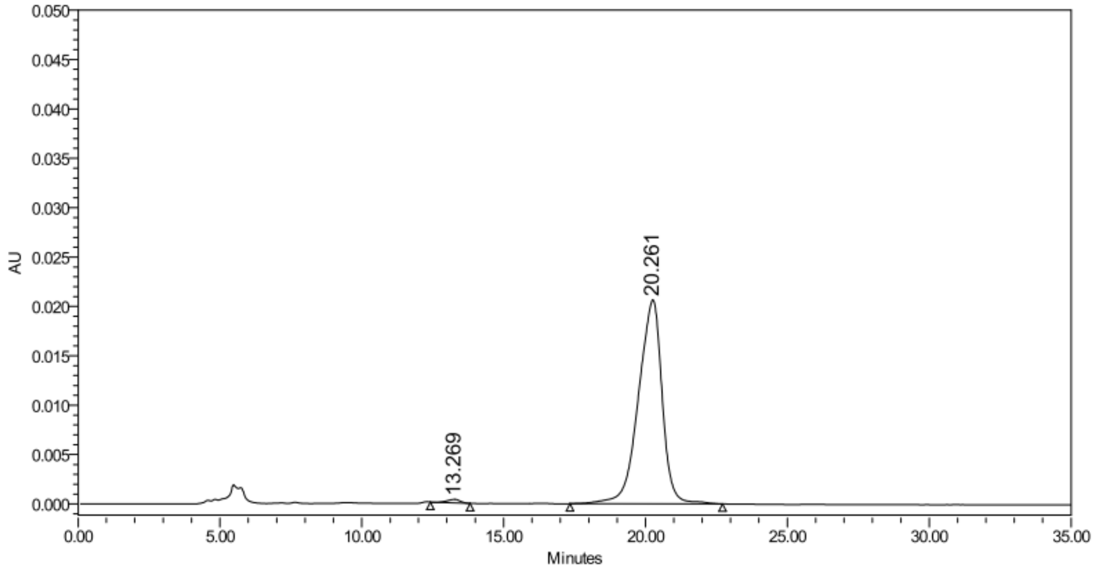

	RT	Area	% Area	Height
1	13.269	11357	0.94	356
2	20.261	1203169	99.06	20667

Supplementary Figure 148. HPLC spectra of **4n**

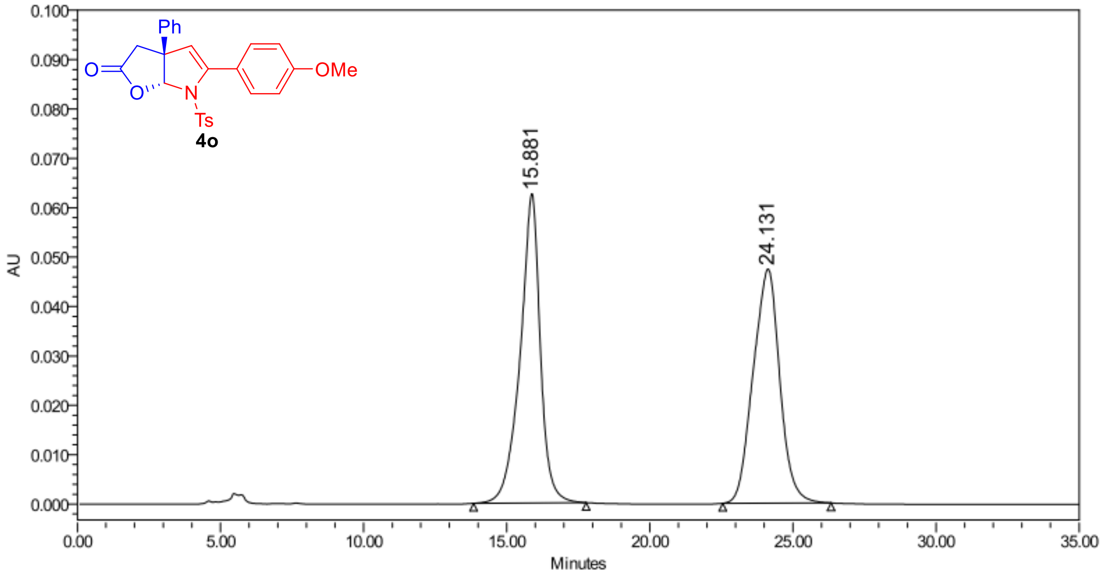

	RT	Area	% Area	Height
1	15.881	3065581	49.97	62605
2	24.131	3069559	50.03	47415

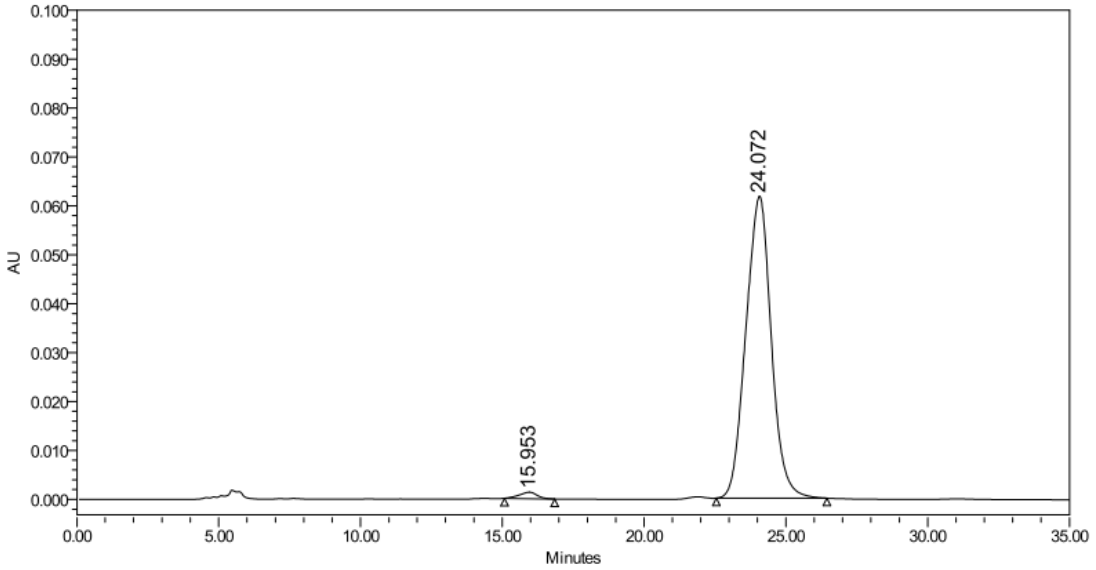

	RT	Area	% Area	Height
1	15.953	56766	1.45	1312
2	24.072	3849150	98.55	61727

Supplementary Figure 149. HPLC spectra of **4o**

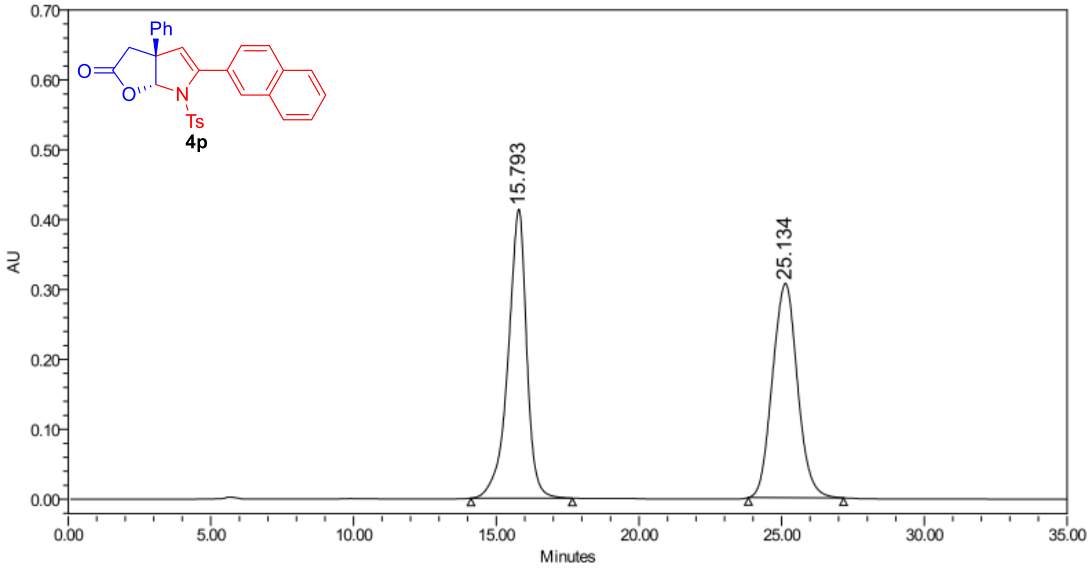

	RT	Area	% Area	Height
1	15.793	18795138	50.14	413658
2	25.134	18688269	49.86	306756

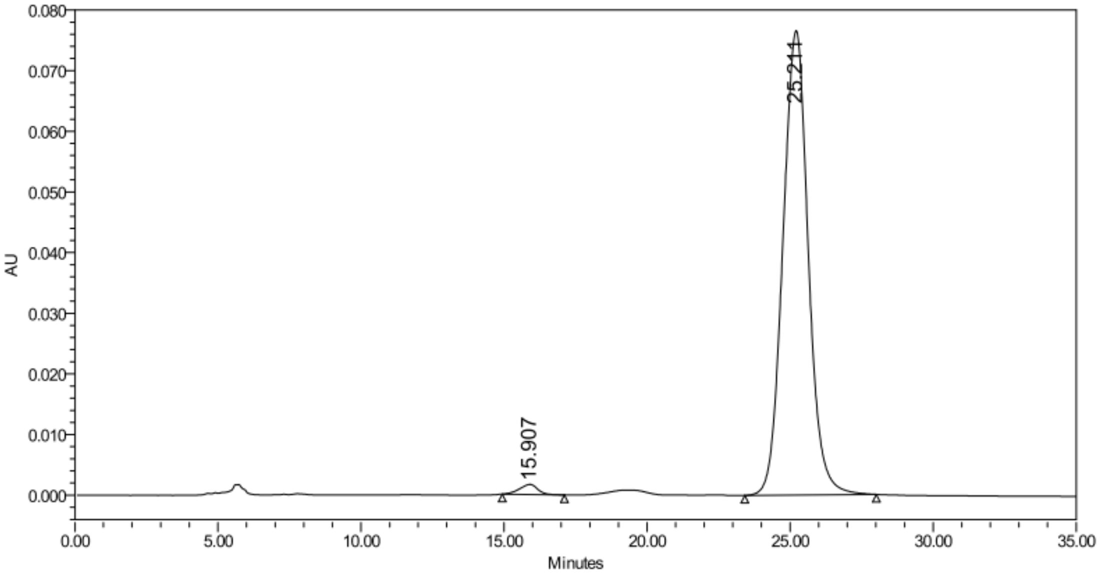

	RT	Area	% Area	Height
1	15.907	73599	1.54	1668
2	25.211	4705028	98.46	76588

Supplementary Figure 150. HPLC spectra of **4p**

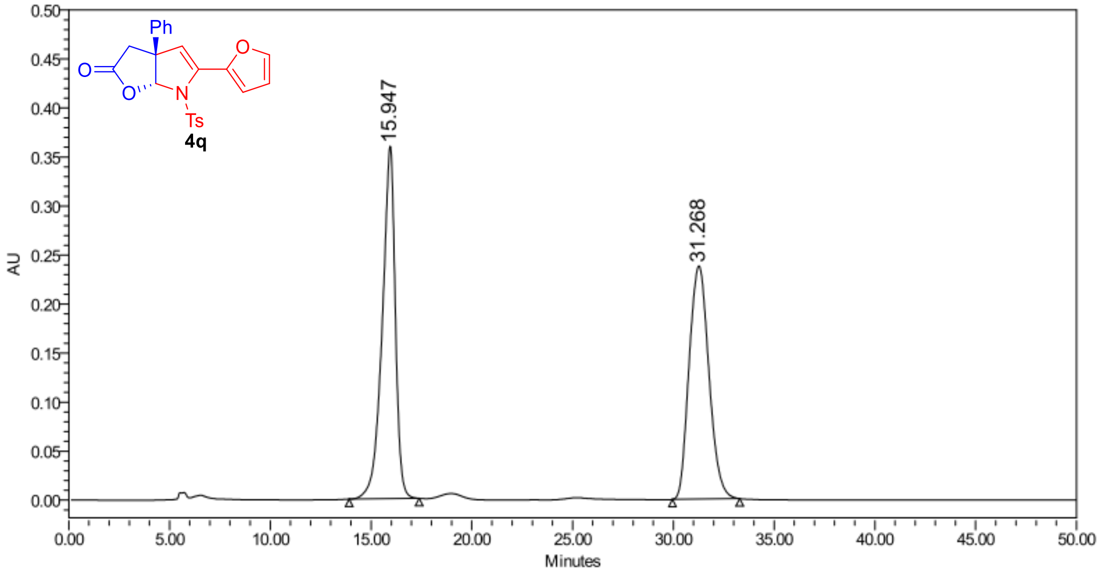

	RT	Area	% Area	Height
1	15.947	16236656	49.98	359369
2	31.268	16250308	50.02	237865

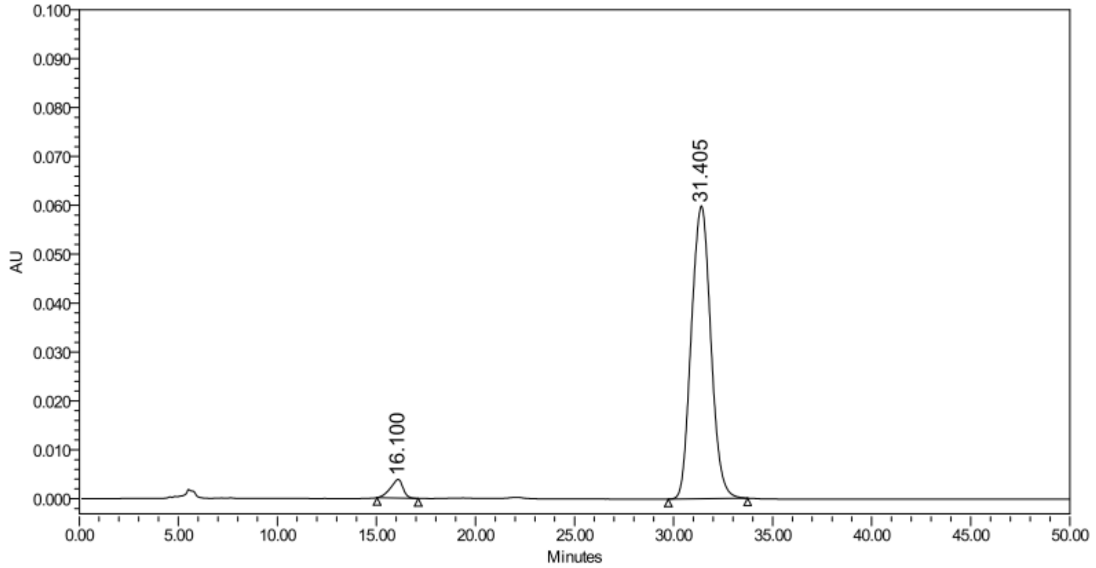

	RT	Area	% Area	Height
1	16.100	168484	3.95	3843
2	31.405	4101721	96.05	59879

Supplementary Figure 151. HPLC spectra of **4q**

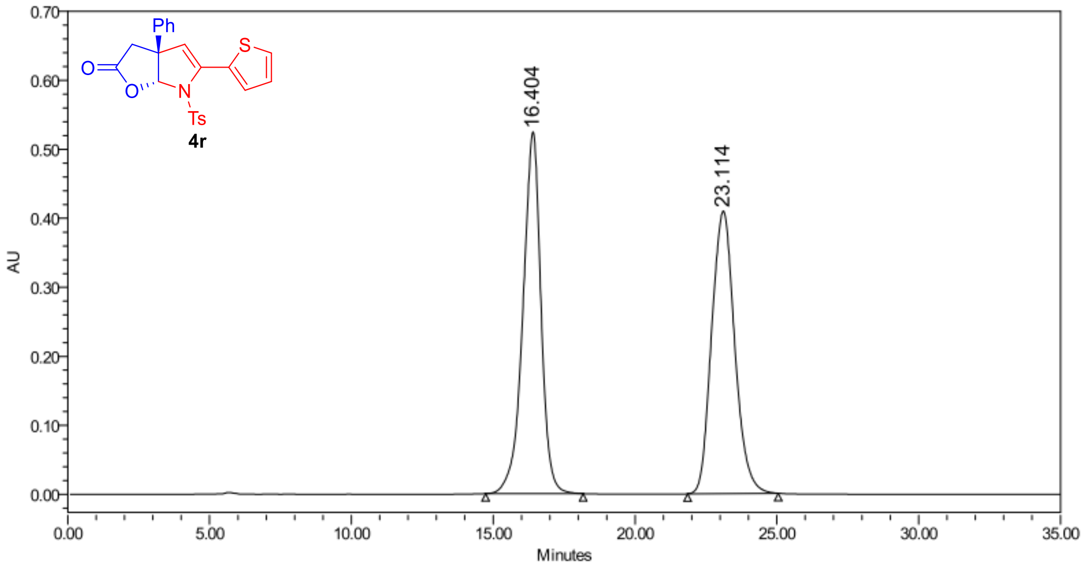

	RT	Area	% Area	Height
1	16.404	23172188	50.01	524162
2	23.114	23165254	49.99	409652

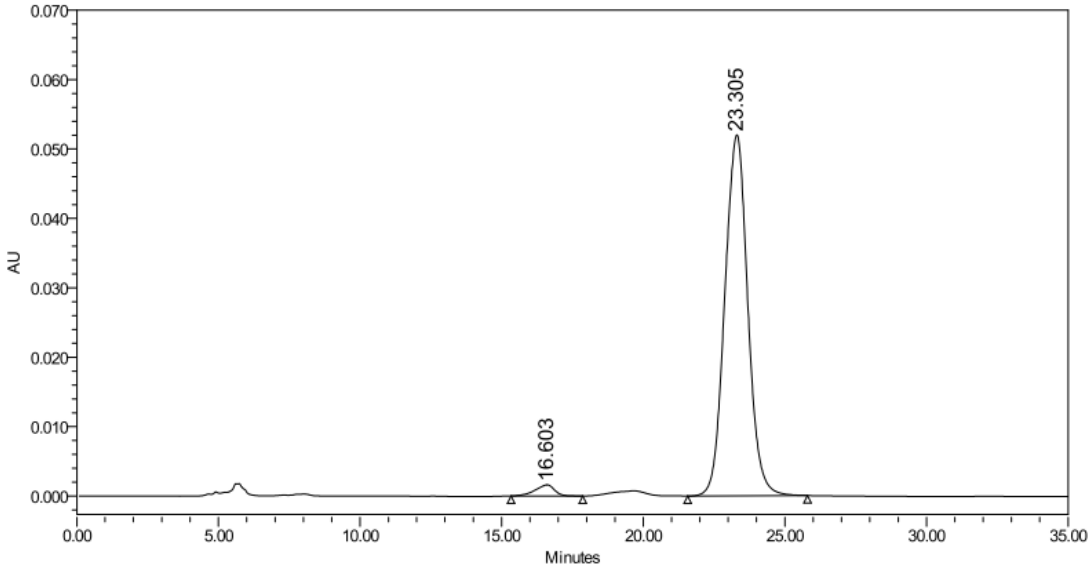

	RT	Area	% Area	Height
1	16.603	76307	2.54	1610
2	23.305	2929703	97.46	52026

Supplementary Figure 152. HPLC spectra of **4r**

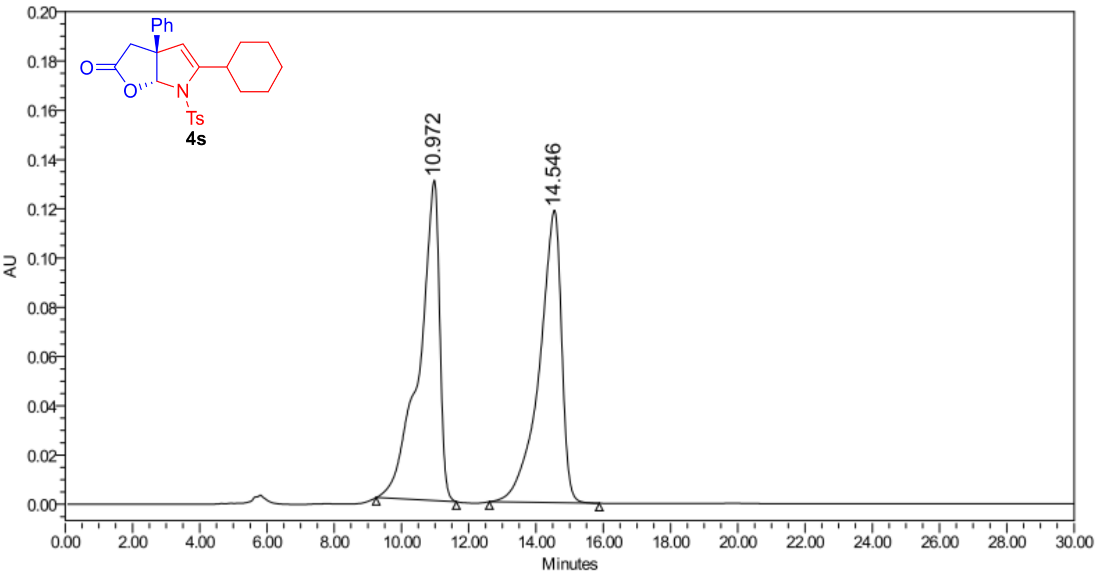

	RT	Area	% Area	Height
1	10.972	5441742	50.03	130062
2	14.546	5434257	49.97	118706

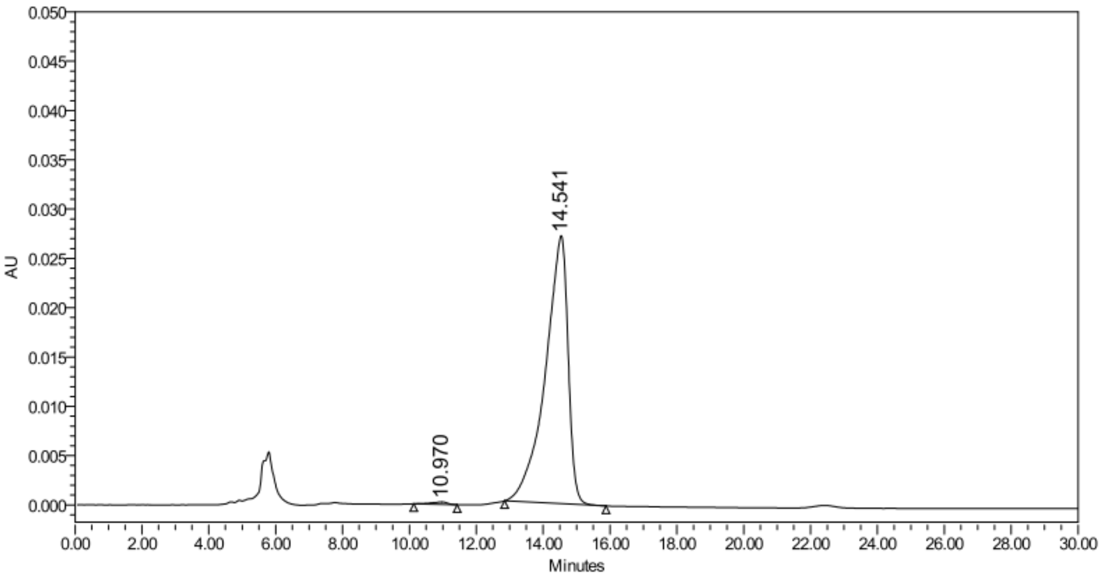

	RT	Area	% Area	Height
1	10.970	7886	0.61	241
2	14.541	1281183	99.39	27168

Supplementary Figure 153. HPLC spectra of **4s**

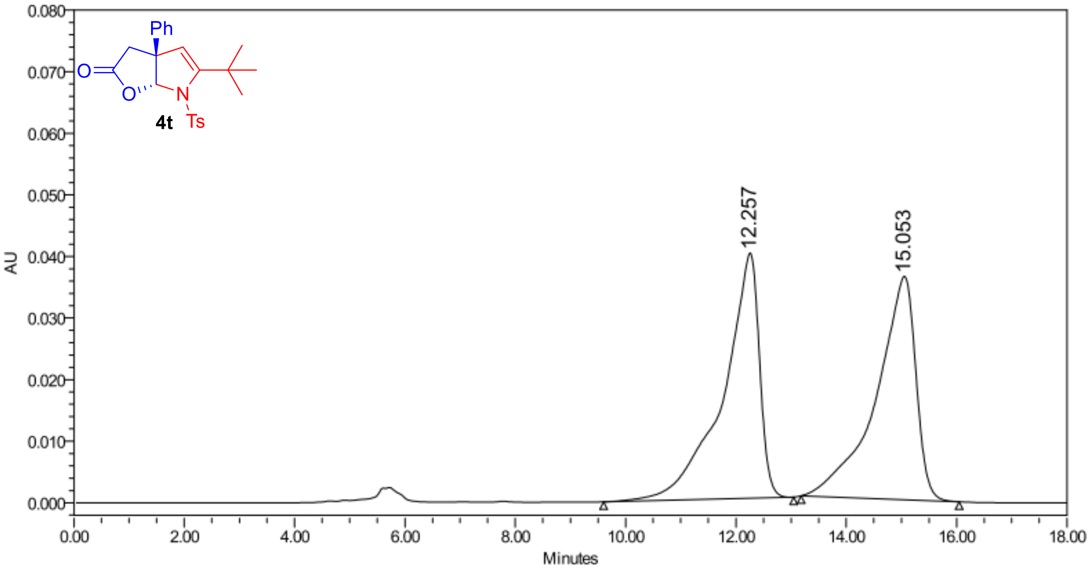

	RT	Area	% Area	Height
1	12.257	1814127	49.96	39817
2	15.053	1816936	50.04	36265

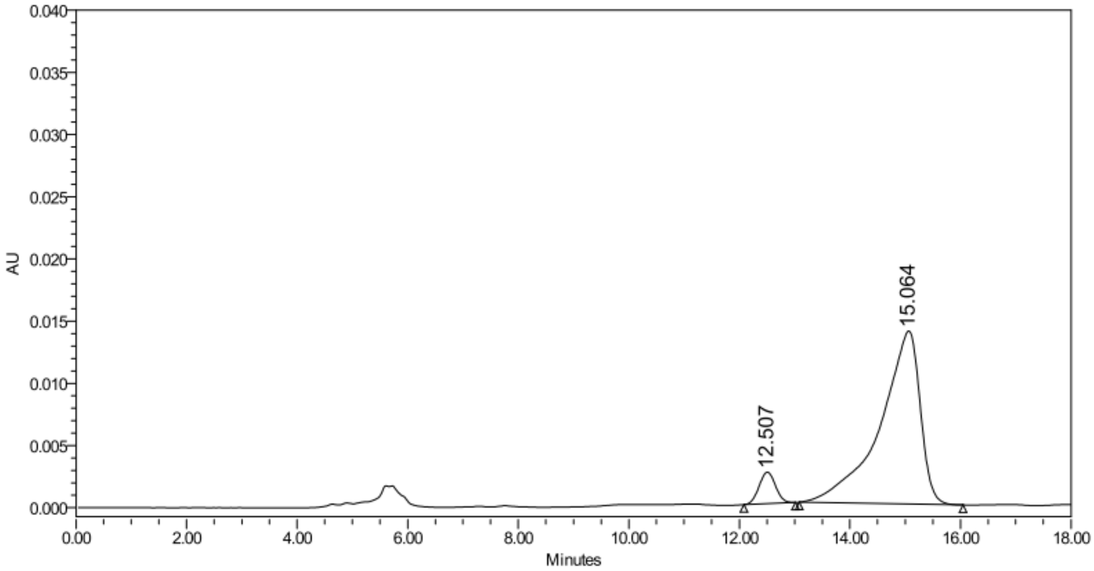

	RT	Area	% Area	Height
1	12.507	52489	6.98	2531
2	15.064	699815	93.02	13918

Supplementary Figure 154. HPLC spectra of **4t**

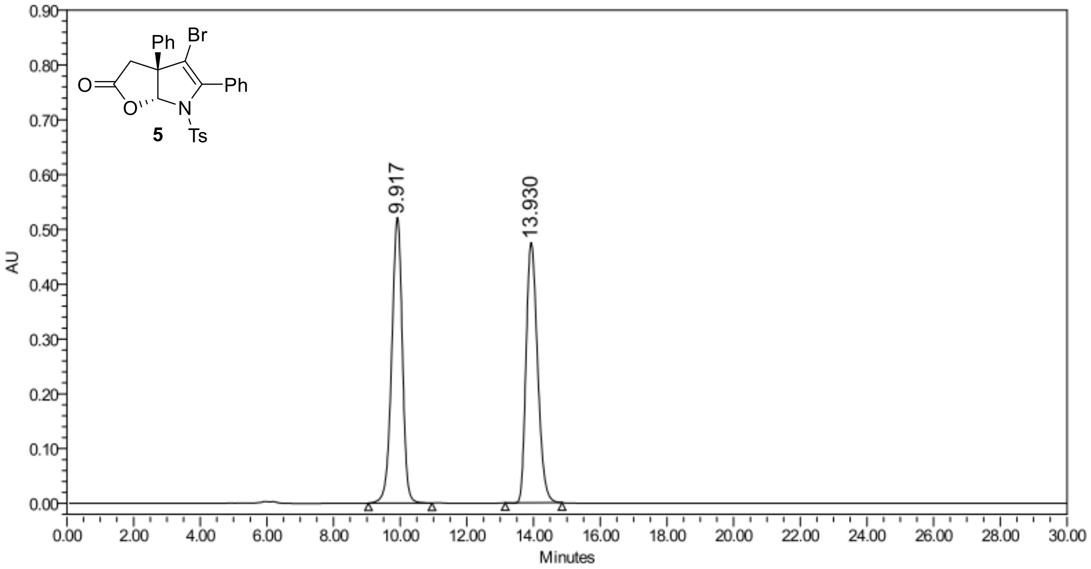

	RT	Area	% Area	Height
1	9.917	11529106	50.35	521059
2	13.930	11370526	49.65	474844

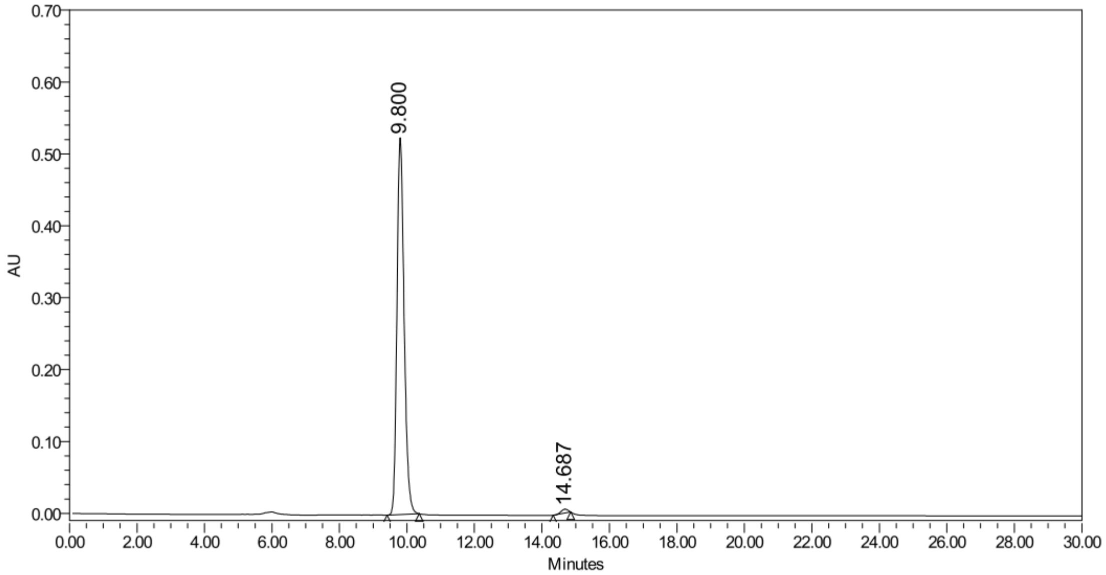

	RT	Area	% Area	Height
1	9.800	7748600	99.04	523828
2	14.687	74789	0.96	5256

Supplementary Figure 155. HPLC spectra of **5**

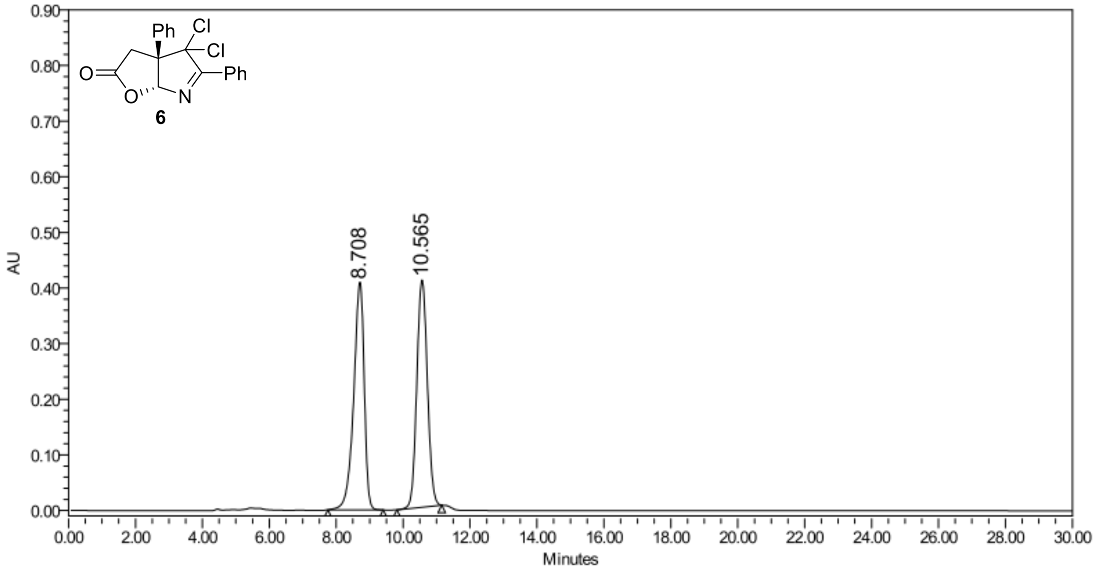

	RT	Area	% Area	Height
1	8.708	8959245	50.00	409515
2	10.565	8959486	50.00	408344

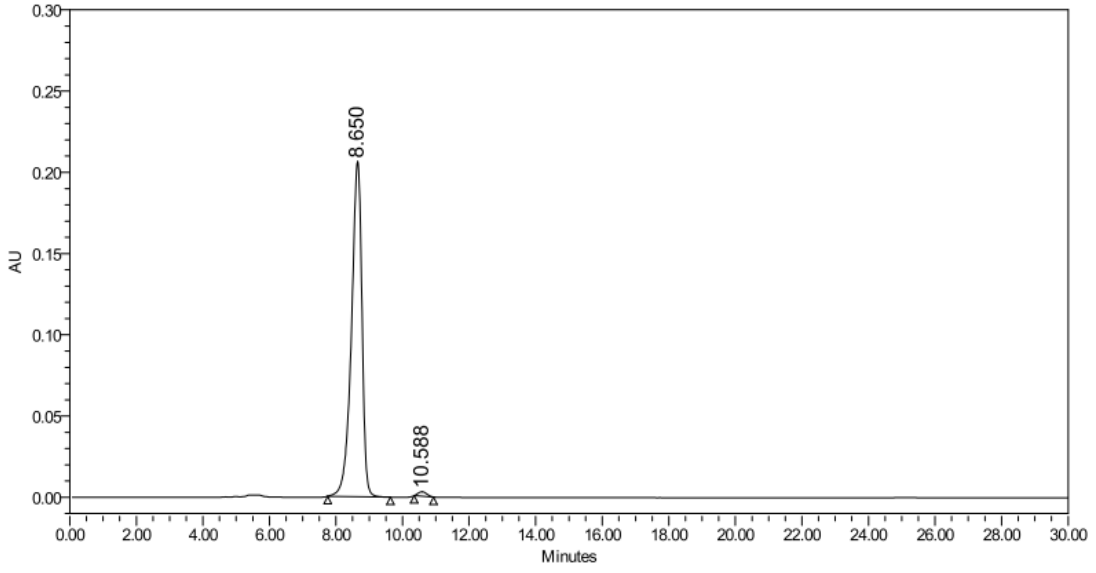

	RT	Area	% Area	Height
1	8.650	4521125	98.91	206263
2	10.588	49844	1.09	2757

Supplementary Figure 156. HPLC spectra of **6**

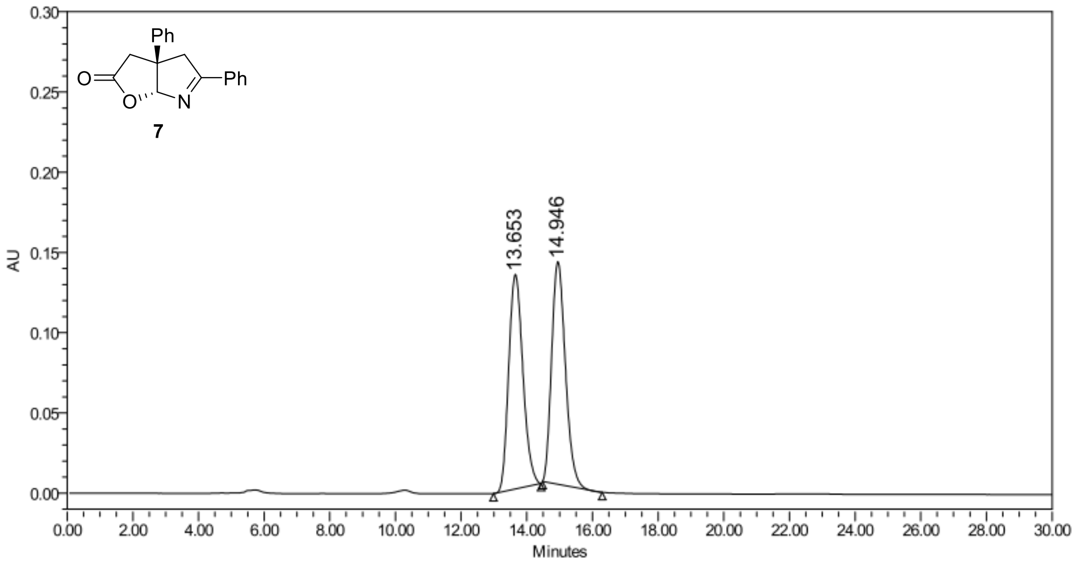

	RT	Area	% Area	Height
1	13.653	4026165	49.91	133567
2	14.946	4041459	50.09	138638

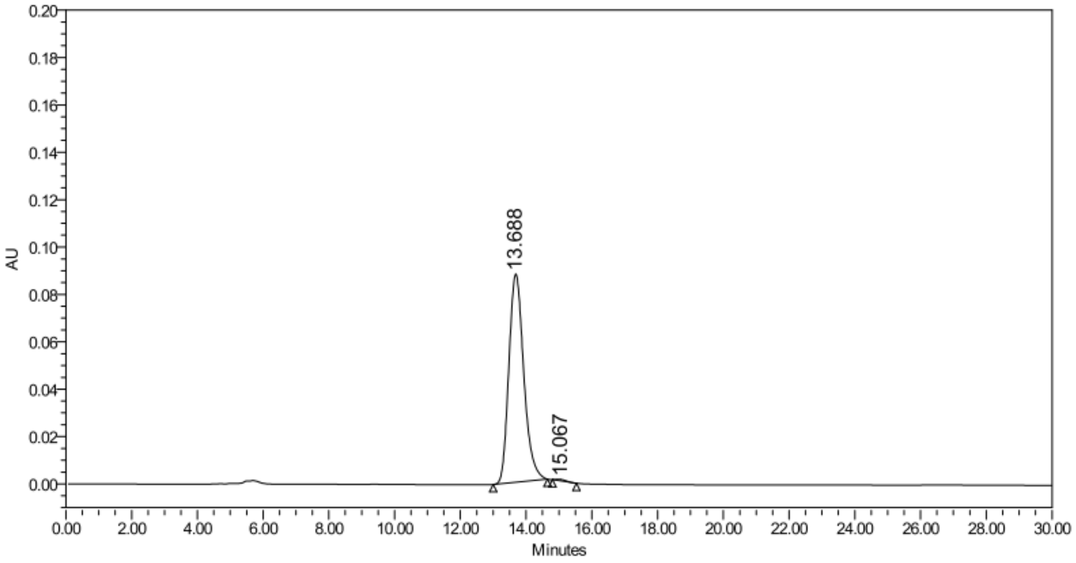

	RT	Area	% Area	Height
1	13.688	2773666	99.54	87913
2	15.067	12951	0.46	634

Supplementary Figure 157. HPLC spectra of **7**

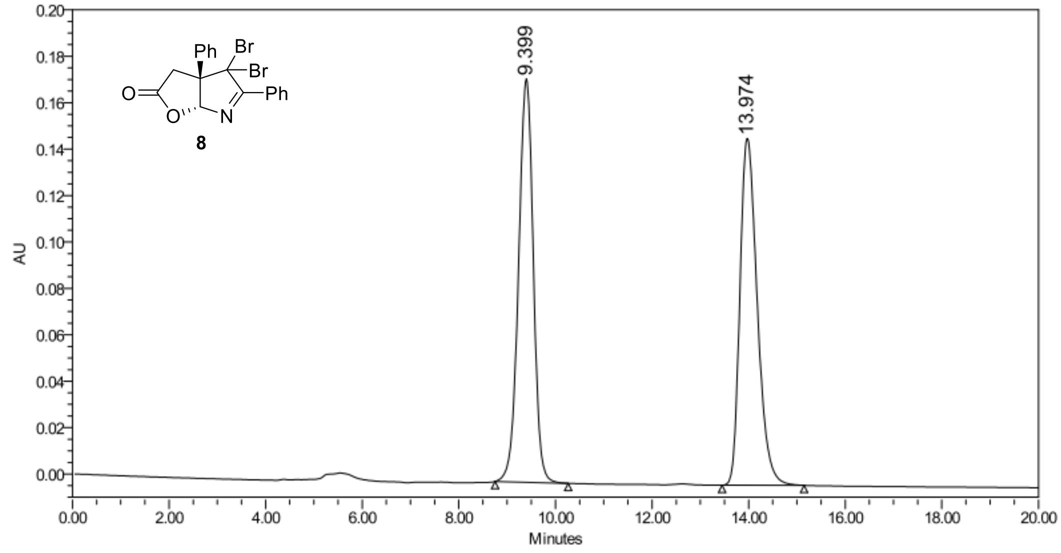

	RT	Area	% Area	Height
1	9.399	3691747	49.83	173747
2	13.974	3717321	50.17	149571

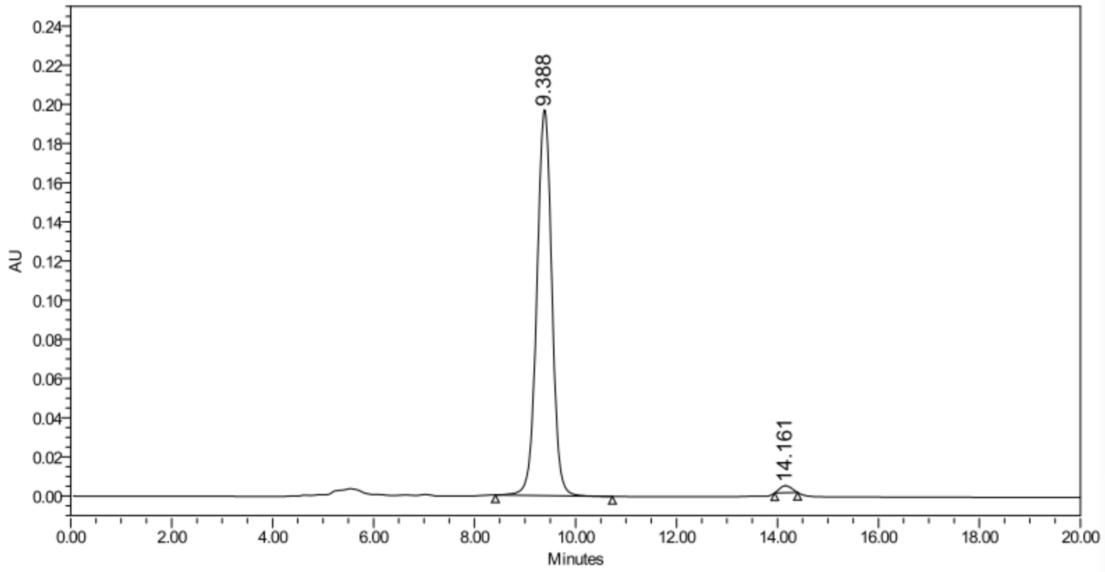

	RT	Area	% Area	Height
1	9.388	4071288	98.63	197137
2	14.161	56639	1.37	3527

Supplementary Figure 158. HPLC spectra of **8**

V. Supplementary References

1. Frisch, M. J.; Trucks, G. W.; Schlegel, H. B.; Scuseria, G. E.; Robb, M. A.; Cheeseman, J. R.; Scalmani, G.; Barone, V.; Mennucci, B.; Petersson, G. A.; Nakatsuji, H.; Caricato, M.; Li, X.; Hratchian, H. P.; Izmaylov, A. F.; Bloino, J.; Zheng, G.; Sonnenberg, J. L.; Hada, M.; Ehara, M.; Toyota, K.; Fukuda, R.; Hasegawa, J.; Ishida, M.; Nakajima, T.; Honda, Y.; Kitao, O.; Nakai, H.; Vreven, T.; Montgomery, J. A., Jr.; Peralta, J. E.; Ogliaro, F.; Bearpark, M.; Heyd, J. J.; Brothers, E.; Kudin, K. N.; Staroverov, V. N.; Keith, T.; Kobayashi, R.; Normand, J.; Raghavachari, K.; Rendell, A.; Burant, J. C.; Iyengar, S. S.; Tomasi, J.; Cossi, M.; Rega, N.; Millam, J. M.; Klene, M.; Knox, J. E.; Cross, J. B.; Bakken, V.; Adamo, C.; Jaramillo, J.; Gomperts, R.; Stratmann, R. E.; Yazyev, O.; Austin, A. J.; Cammi, R.; Pomelli, C.; Ochterski, J. W.; Martin, R. L.; Morokuma, K.; Krzewski, V. G., Voth, G. A.; Salvador, P.; Dannenberg, J. J.; Dapprich, S.; Daniels, A. D.; Farkas, O.; Foresman, J. B.; Ortiz, J. V.; Cioslowski, J.; Fox, D. J.Gaussian 16, rev. A.03.; Gaussian, Inc.: Wallingford, CT, (2016)
2. Lu, T. & Chen, Q. Shermo: A general code for calculating molecular thermodynamic properties, *Comput. Theor. Chem.* **1200**, 113249 (2021).
3. Scott, P Anthony. & Radom, Leo. Harmonic vibrational frequencies: an evaluation of hartree-fock, møller−plesset, quadratic configuration interaction, density functional theory, and semiempirical scale factors. *J. Phys. Chem.* **100**, 16502-16513 (1996).
4. Lu, T. & Chen, F. Multiwfn: a multifunctional wavefunction analyzer. *J. Comp. Chem.* **33**, 580-592 (2012).
5. Humphrey, W.; Dalke, A. & Schulten, K. VMD: visual molecular dynamics. *J. Mol. Graph.* **14**, 33-38 (1996).
6. Lu, T. & Chen, Q. Independent gradient model based on Hirshfeld partition: A new method for visual study of interactions in chemical systems. *J. Comp. Chem.*, **43**, 539-555 (2022)
7. CYLview20; Legault, C. Y. Université de Sherbrooke, 2020 (http://www.cylview.org)
