# Supplementary material for: Carbene-catalyzed chemoselective reaction of unsymmetric enedials for access to Furo[2,3-b]pyrroles
Source: Nat Commun. 2023 Jul 15;14:4243. doi: 10.1038/s41467-023-39988-z (PMC10349821; doi:10.1038/s41467-023-39988-z)
Supplement: Supplementary file 2 — Description of Additional Supplementary Files [file 41467_2023_39988_MOESM2_ESM.docx]

**Description of Additional Supplementary Files**

**File Name: Supplementary Data 1
Description:** Cartesian coordinates of all the optimized structures.
