# Supplementary material for: Carbene-catalyzed chemoselective reaction of unsymmetric enedials for access to Furo[2,3-b]pyrroles
Source: Nat Commun. 2023 Jul 15;14:4243. doi: 10.1038/s41467-023-39988-z (PMC10349821; doi:10.1038/s41467-023-39988-z)
Supplement: Supplementary file 3 — supplementary data 1 [file 41467_2023_39988_MOESM3_ESM.pdf]

## Cartesian coordinates of all the optimized structures

Calculated data.

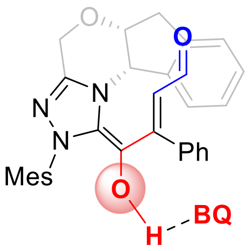


*α-Oxidant*
**SM a'**

Optimization: M062X-D3/def2SVP/IEFPCM($CH_3CN$)

E(RM062X-D3) = -1967.894366 a.u.

The lowest frequency = 15.03 $cm^{-1}$

Number of imaginary frequencies = 0

Single-point energy calculation:

RM062X-D3/def2TZVPP/IEFPCM(CH3CN) E(RM062X-D3) = -1970.092064 a.u.

Sum of electronic energy and thermal correction to G = -1969.5389729 a.u. (Shermo)

| | | | |
|---|---|---|---|
| O | - 1.3431766101 | 0.1230353592 | 0.4925068446 |
| O | - 2.8705258383 | - 3.1670254242 | 3.7663745011 |
| C | 0.7212267772 | - 2.8235750364 | 1.6003431542 |
| C | - 0.3206058291 | - 1.7586963296 | 1.5841263171 |
| C | - 1.8701939076 | - 3.0826775643 | 3.0621482160 |
| H | - 1.2271266808 | - 3.9818904955 | 2.9187609919 |
| C | 1.1485319305 | - 3.4146782695 | 0.4039028514 |
| H | 0.7390315893 | - 3.0574513374 | - 0.5436922113 |
| C | - 1.4666359246 | - 1.8950203168 | 2.3595108334 |
| H | - 2.1766447801 | - 1.0661149306 | 2.3889446503 |
| C | 1.2394115799 | - 3.2992147201 | 2.8138814760 |
| H | 0.9233344837 | - 2.8299077680 | 3.7474063049 |
| C | - 0.2190874364 | - 0.6604007342 | 0.6926461062 |
| C | 2.1614347256 | - 4.3419356263 | 2.8299923228 |
| H | 2.5647900387 | - 4.6932945713 | 3.7814873137 |
| C | 2.5749388404 | - 4.9311763558 | 1.6335441449 |
| H | 3.2949322487 | - 5.7511478822 | 1.6462572188 |
| C | 2.0647835564 | - 4.4661229927 | 0.4218738699 |
| H | 2.3818317800 | - 4.9233955939 | - 0.5170432394 |
| C | 2.5711229225 | 1.4554756025 | - 2.7644255565 |

| | | | |
|---|---|---|---|
| C | 2.2293113620 | 0.8456618487 | - 1.4347359945 |
| C | - 0.1877837527 | 0.9204539705 | - 1.9729451100 |
| C | 0.3192939669 | 1.1590283801 | - 3.4125966166 |
| H | 3.0065749870 | 0.6616898898 | - 3.3984135746 |
| H | 3.3228576367 | 2.2435982714 | - 2.6368374657 |
| H | - 0.8876449131 | 0.0709098993 | - 1.9652910672 |
| H | 0.5747024255 | 0.2189382512 | - 3.9297576352 |
| O | 1.4405047828 | 2.0210517409 | - 3.3680646961 |
| N | 3.0501394651 | 0.3783180826 | - 0.5571956854 |
| N | 0.9253267856 | 0.5905623993 | - 1.1005511859 |
| C | - 0.8259833568 | 1.9388150776 | - 4.0579682907 |
| H | - 1.5979635474 | 1.2434523332 | - 4.4249099573 |
| H | - 0.4679184211 | 2.5336785739 | - 4.9082378307 |
| C | - 0.9126308850 | 2.2242720579 | - 1.6868454749 |
| C | - 1.1370454341 | 2.8863446335 | - 0.4831873304 |
| C | - 1.3285024504 | 2.7775360101 | - 2.9045754210 |
| C | - 1.8196974322 | 4.1048218767 | - 0.5096577841 |
| H | - 0.7944655828 | 2.4543981675 | 0.4567815122 |
| C | - 2.0410943343 | 3.9722153284 | - 2.9258150963 |
| C | - 2.2881913859 | 4.6313483365 | - 1.7161531076 |
| H | - 2.3775843467 | 4.4042775513 | - 3.8706200104 |
| H | - 2.8366406390 | 5.5750182687 | - 1.7187776734 |
| C | 0.9324560610 | - 0.1575325997 | 0.0568641662 |
| N | 2.2552104351 | - 0.2142097991 | 0.3924328139 |
| C | 2.8483531793 | - 0.6241322452 | 1.6230672466 |
| C | 3.9020630721 | - 1.5526909417 | 1.5780682939 |
| C | 2.4139635051 | - 0.0441321112 | 2.8267770961 |
| C | 4.4815372021 | - 1.9365961993 | 2.7875958502 |
| C | 3.0162106023 | - 0.4833685302 | 4.0095873234 |
| C | 4.0387271366 | - 1.4345109896 | 4.0142105005 |
| H | 5.2943191231 | - 2.6675559741 | 2.7693038958 |
| H | 2.6857546803 | - 0.0476364398 | 4.9560010748 |
| C | 1.3635675544 | 1.0349233899 | 2.8789207080 |
| H | 1.5094000173 | 1.6545319912 | 3.7727536244 |
| H | 0.3495350528 | 0.6084587723 | 2.9205540052 |
| H | 1.4107732337 | 1.6866411223 | 1.9948022923 |
| C | 4.4224375487 | - 2.1047664602 | 0.2780458299 |
| H | 5.1505462091 | - 1.4132503526 | - 0.1713028731 |
| H | 3.6150944858 | - 2.2458544400 | - 0.4514839577 |

| | | | |
|---|---|---|---|
| H | 4.9146073692 | - 3.0711623516 | 0.4473029463 |
| C | 4.6367601672 | - 1.9254016513 | 5.3056592080 |
| H | 5.7121293471 | - 2.1210370667 | 5.1981349360 |
| H | 4.1603162898 | - 2.8696462763 | 5.6135335327 |
| H | 4.4903051032 | - 1.1995566186 | 6.1161080104 |
| H | - 2.0005294589 | 4.6429492461 | 0.4219991899 |
| H | - 2.0215584600 | - 0.3869857153 | 0.0137029614 |
| C | - 4.7945219834 | 1.7335359870 | - 3.6805884305 |
| C | - 4.1531891568 | 0.6210197418 | - 3.3016291540 |
| C | - 3.7178221828 | 0.4312647889 | - 1.8933857405 |
| C | - 4.0591142606 | 1.4852388855 | - 0.9088766758 |
| C | - 4.7168154912 | 2.5899646680 | - 1.2845342343 |
| H | - 5.1141376128 | 1.9062306509 | - 4.7099019311 |
| H | - 4.9755539842 | 3.3840530034 | - 0.5821642553 |
| O | - 3.0938253780 | - 0.5662638871 | -1.5686101642 |
| C | - 5.1170742648 | 2.8105523507 | - 2.7009864008 |
| O | - 5.6877466885 | 3.8218580188 | - 3.0470783200 |
| H | - 3.9100423785 | - 0.1863459176 | - 3.9949049463 |
| H | - 3.7340253071 | 1.3183689683 | 0.1199981096 |

Calculated data.

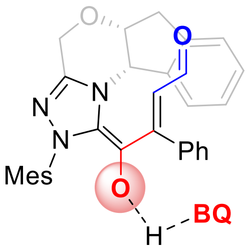


*α-Oxidant*

**BQ-a-TS**

Optimization: M062X-D3/def2SVP/IEFPCM($CH_3CN$)

E(RM062X-D3) = -1967.86035887 a.u.

The lowest frequency = -1188.9206 $cm^{-1}$

Number of imaginary frequencies = 1

Single-point energy calculation:

RM062X-D3/def2TZVPP/IEFPCM(CH3CN) E(RM062X-D3) = -1970.05672690 a.u.

Sum of electronic energy and thermal correction to G = -1969.5058403 a.u. (Shermo)

| | | | |
|---|---|---|---|
| O | - 3.1027746657 | 0.2291939760 | - 0.3449769057 |
| O | - 4.5040069109 | - 2.7211386067 | 3.2758941597 |
| C | - 1.0991018051 | - 2.6773107641 | 0.7805130129 |
| C | - 2.0964458957 | - 1.5780138500 | 0.8254706040 |
| C | - 3.5822659411 | - 2.7639758592 | 2.4815913022 |
| H | - 3.0270258209 | - 3.7136844194 | 2.3187937839 |
| C | - 0.7538661594 | - 3.2674480495 | - 0.4438879095 |
| H | - 1.1977343730 | - 2.8910916351 | - 1.3688576210 |
| C | - 3.1787521066 | - 1.6224674561 | 1.6710941178 |
| H | - 3.8515179395 | - 0.7621854926 | 1.7066128885 |
| C | - 0.5405356741 | - 3.1854205058 | 1.9629118861 |
| H | - 0.7818978964 | - 2.7102531284 | 2.9160772230 |
| C | - 2.0480016210 | - 0.4629687138 | - 0.1042093198 |
| C | 0.3400604635 | - 4.2627243979 | 1.9216141327 |
| H | 0.7763430605 | - 4.6406673006 | 2.8478093134 |
| C | 0.6738837727 | - 4.8484244285 | 0.6991304271 |
| H | 1.3640508294 | - 5.6929002125 | 0.6669720335 |
| C | 0.1246170320 | - 4.3493309112 | - 0.4820613344 |
| H | 0.3800027269 | - 4.8047693057 | - 1.4401553151 |
| C | 0.7284978606 | 1.4093974486 | - 3.7354508970 |
| C | 0.4004131830 | 0.8618966387 | - 2.3788611410 |
| C | - 2.0297638668 | 1.0666979954 | - 2.8303412147 |
| C | - 1.5648812286 | 1.3012551184 | - 4.2870878916 |

| | | | |
|---|---|---|---|
| H | 1.0494774078 | 0.5653778348 | - 4.3732286490 |
| H | 1.5597552028 | 2.1201201819 | - 3.6596251304 |
| H | - 2.7910138596 | 0.2717717679 | - 2.7995023484 |
| H | - 1.4113334599 | 0.3574288019 | - 4.8362831807 |
| O | - 0.3758924066 | 2.0698318943 | - 4.2815485437 |
| N | 1.2289777894 | 0.4364099980 | - 1.4782654206 |
| N | - 0.8971311755 | 0.6428099482 | - 2.0077484779 |
| C | - 2.6627860137 | 2.1947900415 | - 4.8603187879 |
| H | - 3.5067183290 | 1.5838639388 | - 5.2151783951 |
| H | - 2.2797712529 | 2.7852461217 | - 5.7028763502 |
| C | - 2.6391450946 | 2.4110356023 | - 2.4783100428 |
| C | - 2.8083594369 | 3.0289065705 | - 1.2444552926 |
| C | - 3.0545893205 | 3.0298961709 | - 3.6630870239 |
| C | - 3.4273031695 | 4.2810157909 | - 1.2070125729 |
| H | - 2.4800386010 | 2.5410036114 | - 0.3268803500 |
| C | - 3.6957842637 | 4.2635932161 | - 3.6204761007 |
| C | - 3.8809502618 | 4.8849391463 | - 2.3818046603 |
| H | - 4.0400229564 | 4.7437565034 | - 4.5382775113 |
| H | - 4.3747239610 | 5.8570172368 | - 2.3342176670 |
| C | - 0.8638221702 | - 0.0264046746 | - 0.8166670060 |
| N | 0.4508740599 | - 0.0956342346 | - 0.5019953926 |
| C | 1.0567183614 | - 0.5080803138 | 0.7265432582 |
| C | 2.0794052903 | - 1.4701642715 | 0.6696840152 |
| C | 0.6539335165 | 0.1013430325 | 1.9257104091 |
| C | 2.6563505681 | - 1.8634194695 | 1.8763588028 |
| C | 1.2563201285 | - 0.3481573597 | 3.1044797509 |
| C | 2.2436954951 | - 1.3357587929 | 3.1037659268 |
| H | 3.4428061676 | - 2.6221731479 | 1.8564499291 |
| H | 0.9546661880 | 0.1079186360 | 4.0505333258 |
| C | - 0.3508062734 | 1.2240340226 | 1.9820328833 |
| H | - 0.1392262704 | 1.8651411823 | 2.8468197780 |
| H | - 1.3788550799 | 0.8462212286 | 2.0934747269 |
| H | - 0.3167688512 | 1.8493658118 | 1.0788020547 |
| C | 2.5684260607 | - 2.0509743928 | - 0.6301700700 |
| H | 3.3162918174 | - 1.3881486442 | - 1.0899067130 |
| H | 1.7536233436 | - 2.1760320241 | - 1.3541865004 |
| H | 3.0305750493 | - 3.0305063288 | - 0.4538687542 |
| C | 2.8391418953 | - 1.8385761376 | 4.3907989390 |
| H | 3.9094728477 | - 2.0556586979 | 4.2758376341 |

| | | | |
|---|---|---|---|
| H | 2.3451399923 | - 2.7740357110 | 4.6975735000 |
| H | 2.7108276110 | - 1.1113804099 | 5.2027045313 |
| H | - 3.5647519690 | 4.7861084553 | - 0.2497734620 |
| H | - 4.1377902394 | - 0.3006017350 | - 0.9197587647 |
| C | - 6.1552245605 | 1.3883894801 | - 4.3649134828 |
| C | - 5.5865766069 | 0.3496320352 | - 3.6875228431 |
| C | - 5.4252099728 | 0.3985182053 | - 2.2651916954 |
| C | - 5.8586951378 | 1.5691653904 | - 1.5592270926 |
| C | - 6.4073094192 | 2.6228847007 | - 2.2260048260 |
| H | - 6.2948699629 | 1.3544699102 | - 5.4473062006 |
| H | - 6.7279864528 | 3.5239262014 | - 1.7001450941 |
| O | - 4.8919296259 | - 0.6136895963 | - 1.6431401117 |
| C | - 6.5860782180 | 2.6109255697 | - 3.6812633905 |
| O | - 7.0702912280 | 3.5691620035 | - 4.2915163048 |
| H | - 5.2505870526 | - 0.5542550400 | - 4.2007551392 |
| H | - 5.7196281630 | 1.5912945984 | - 0.4760484513 |

Calculated data.

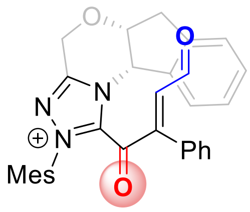


IM a'

Optimization: M062X-D3/def2SVP/IEFPCM($CH_3CN$)

E(RM062X-D3) = -1967.89044984 a.u.

The lowest frequency = 20.1709 $cm^{-1}$

Number of imaginary frequencies = 0

Single-point energy calculation:

RM062X-D3/def2TZVPP/IEFPCM(CH3CN) E(RM062X-D3) = -1970.09217375 a.u.

Sum of electronic energy and thermal correction to G = -1969.5379420 a.u. (Shermo)

| | | | |
|---|---|---|---|
| O | - 1.3502338491 | 0.7216991911 | 1.0378962309 |
| O | - 1.5759552358 | - 2.2693437053 | 5.0656276327 |
| C | 0.2164961944 | - 2.5034527352 | 1.1881596307 |
| C | - 0.3759178815 | - 1.3072306819 | 1.8234351968 |
| C | - 1.2056271372 | - 2.4080194491 | 3.9258600407 |
| H | - 1.0848457235 | - 3.4157477021 | 3.4751907457 |
| C | - 0.0172358456 | - 2.7345557072 | - 0.1766751687 |
| H | - 0.6278862128 | - 2.0321663363 | - 0.7494549188 |
| C | - 0.9513595143 | - 1.2459959485 | 3.0425799881 |
| H | - 1.3255936841 | - 0.2892541232 | 3.4193724382 |
| C | 0.9900883585 | - 3.4186804766 | 1.9169200140 |
| H | 1.2363986410 | - 3.2113105257 | 2.9606017495 |
| C | - 0.4102096002 | - 0.0303708269 | 1.0348603300 |
| C | 1.4858558024 | - 4.5641358436 | 1.2989995951 |
| H | 2.0906813794 | - 5.2677544120 | 1.8724442750 |
| C | 1.2228613735 | - 4.8024843960 | - 0.0512997684 |
| H | 1.6122844106 | - 5.7010256474 | - 0.5317151279 |
| C | 0.4777449388 | - 3.8827156253 | - 0.7909471648 |
| H | 0.2769010858 | - 4.0592722035 | -1.8483772006 |
| C | 2.2711579170 | 1.4651533807 | - 2.8899394837 |
| C | 1.9923789033 | 0.9977916626 | - 1.4949127688 |
| C | - 0.4428665115 | 1.4800221210 | - 1.7705993516 |
| C | - 0.0611422465 | 1.6701124682 | - 3.2596670625 |

| | | | |
|---|---|---|---|
| H | 2.4109576470 | 0.5692863696 | - 3.5227223210 |
| H | 3.1987042850 | 2.0492071497 | - 2.9063331550 |
| H | - 1.2767025393 | 0.7661374670 | - 1.6739107784 |
| H | - 0.0873499286 | 0.7198832240 | - 3.8183131824 |
| O | 1.2246651035 | 2.2661345392 | - 3.3464221493 |
| N | 2.8306476491 | 0.5135037829 | - 0.6212525692 |
| N | 0.7105222867 | 0.9365871838 | - 1.0272852089 |
| C | - 1.0543402962 | 2.7215033345 | - 3.7505666274 |
| H | - 1.9902762754 | 2.2506113337 | - 4.1000312944 |
| H | - 0.6129246681 | 3.2833512003 | - 4.5852896553 |
| C | - 0.8676237493 | 2.8798770142 | - 1.3661241327 |
| C | - 0.9196868465 | 3.4833300804 | - 0.1149471265 |
| C | - 1.2654849271 | 3.5676994409 | - 2.5198199608 |
| C | - 1.3974852431 | 4.7944918394 | - 0.0234375142 |
| H | - 0.6007579658 | 2.9555595539 | 0.7832148078 |
| C | - 1.7539368817 | 4.8668376234 | - 2.4229165903 |
| C | - 1.8206274502 | 5.4756042702 | - 1.1660308792 |
| H | - 2.0644370815 | 5.4089483073 | - 3.3179607754 |
| H | - 2.1936417740 | 6.4973406344 | - 1.0790797851 |
| C | 0.7747874262 | 0.3287949299 | 0.1698852519 |
| N | 2.0678989953 | 0.1034844455 | 0.4059446581 |
| C | 2.6672830455 | - 0.4071189435 | 1.6089468480 |
| C | 3.4932983740 | - 1.5398417643 | 1.5017247915 |
| C | 2.4285667775 | 0.2604946031 | 2.8174782207 |
| C | 4.0547613494 | - 2.0264482544 | 2.6781291044 |
| C | 3.0064191393 | - 0.2867157083 | 3.9683550764 |
| C | 3.8121736753 | - 1.4249719817 | 3.9210770127 |
| H | 4.6884351732 | - 2.9150507625 | 2.6271890158 |
| H | 2.8336989509 | 0.2106098899 | 4.9250390748 |
| C | 1.6178676536 | 1.5269420064 | 2.9177696918 |
| H | 1.9833804741 | 2.1325929165 | 3.7556723858 |
| H | 0.5555957232 | 1.3184541492 | 3.1212713203 |
| H | 1.6835114736 | 2.1397256986 | 2.0081122378 |
| C | 3.7407722079 | - 2.2202188356 | 0.1843648569 |
| H | 4.4318143818 | - 1.6332507256 | - 0.4368235870 |
| H | 2.8056010705 | - 2.3388928392 | - 0.3835277786 |
| H | 4.1740436955 | - 3.2139429438 | 0.3484847339 |
| C | 4.4188649498 | - 2.0032693342 | 5.1696209645 |
| H | 5.5099429753 | - 2.0884484335 | 5.0676188810 |

| | | | |
|---|---|---|---|
| H | 4.0287169859 | - 3.0153666432 | 5.3515716150 |
| H | 4.1962781708 | - 1.3843805084 | 6.0471519100 |
| H | - 1.4388189768 | 5.2854852419 | 0.9496325911 |
| H | - 3.0756223936 | - 0.9171496541 | 0.9764980696 |
| C | - 2.9419308768 | - 0.8894304173 | - 3.3595247437 |
| C | - 2.7867777261 | - 1.5243126973 | - 2.1305383337 |
| C | - 3.1388106146 | - 0.8786674423 | - 0.9401828988 |
| C | - 3.6693179160 | 0.4130346918 | - 1.0129696501 |
| C | - 3.8268463218 | 1.0511567853 | - 2.2414699949 |
| H | - 2.6608947859 | - 1.4089155488 | - 4.2795447883 |
| H | - 4.2332659823 | 2.0659438584 | - 2.2773652719 |
| O | - 2.9395118350 | - 1.5412059030 | 0.2519754445 |
| C | - 3.4836002549 | 0.4352872197 | - 3.4936667098 |
| O | - 3.6184448496 | 1.0195371728 | - 4.6159350801 |
| H | - 2.3931581550 | - 2.5432106390 | - 2.0792331527 |
| H | - 3.9419337069 | 0.9311265942 | - 0.0881557030 |

Calculated data.

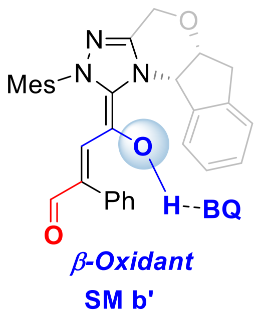


Optimization: M062X-D3/def2SVP/IEFPCM($CH_3CN$)

E(RM062X-D3) = -1967.90218113 a.u.

The lowest frequency = 21.6660 $cm^{-1}$

Number of imaginary frequencies = 0

Single-point energy calculation:

RM062X-D3/def2TZVPP/IEFPCM(CH3CN) E(RM062X-D3) = -1970.09705166 a.u.

Sum of electronic energy and thermal correction to G = -1969.5428419 a.u. (Shermo)

| | | | |
|---|---|---|---|
| O | 3.3584986723 | 3.6300289477 | 2.1241432309 |
| O | - 0.5972603492 | 1.4114147537 | - 0.7395146506 |
| C | 1.5536619546 | 3.4195808587 | - 0.1640194570 |
| C | 1.8020572818 | 2.3508144020 | 0.8313228266 |
| C | 0.1544264488 | 0.5947127706 | 0.0759366461 |
| C | 1.3438817164 | 4.7489372642 | 0.2392871394 |
| H | 1.3376855621 | 4.9838343809 | 1.3034842914 |
| C | 1.2466563120 | 1.0855946237 | 0.8193403840 |
| H | 1.6490683049 | 0.3957610602 | 1.5645019795 |
| C | 1.5576631099 | 3.1355488271 | - 1.5396534050 |
| H | 1.7325454191 | 2.1090814677 | - 1.8683674089 |
| C | 2.7660554771 | 2.5910265466 | 1.8979877422 |
| H | 2.9489779480 | 1.6943287704 | 2.5431646311 |
| C | 1.3425485691 | 4.1399430210 | - 2.4816197793 |
| H | 1.3465524202 | 3.8938977917 | - 3.5449287131 |
| C | 1.1281997350 | 5.4546157858 | - 2.0660341478 |
| H | 0.9605997483 | 6.2430880882 | - 2.8015139703 |
| C | 1.1318408262 | 5.7541910373 | - 0.7019980146 |
| H | 0.9619542671 | 6.7794293147 | - 0.3683605967 |
| C | - 2.6084569667 | - 3.4546740083 | - 0.4219649696 |
| C | - 1.4160882673 | - 2.6198468815 | - 0.0813993988 |
| C | - 2.3403877915 | - 0.7207873204 | - 1.3861073174 |
| C | - 3.5742231297 | - 1.6438321245 | - 1.5575577037 |

| | | | |
|---|---|---|---|
| H | - 3.3270221857 | - 3.3876675191 | 0.4162228598 |
| H | - 2.3077201952 | - 4.5005909216 | - 0.5473490771 |
| H | - 2.6611282556 | 0.2633035355 | - 1.0226238810 |
| H | - 4.3110895356 | - 1.5073690721 | - 0.7483592145 |
| O | - 3.1780144334 | - 2.9985319471 | - 1.6187805937 |
| N | - 0.3654862383 | - 2.9704414641 | 0.5755058487 |
| N | - 1.3961050840 | - 1.2870839603 | - 0.4203405439 |
| C | - 4.1086411802 | - 1.2692253713 | - 2.9396257046 |
| H | - 4.7854676334 | - 0.4042967918 | - 2.8636188320 |
| H | - 4.6684539907 | - 2.1072064531 | - 3.3750188567 |
| C | - 1.8176161670 | - 0.6104858541 | - 2.8060397528 |
| C | - 0.5539403632 | - 0.2541791121 | - 3.2647794139 |
| C | - 2.8525241762 | - 0.9099716231 | - 3.6978475603 |
| C | - 0.3333293378 | - 0.2057637406 | - 4.6442674559 |
| H | 0.2489794248 | - 0.0144834302 | - 2.5672617786 |
| C | - 2.6352086410 | - 0.8505351181 | - 5.0710744871 |
| C | - 1.3660450318 | - 0.4975049750 | - 5.5387476400 |
| H | - 3.4381866419 | - 1.0863601344 | - 5.7717912726 |
| H | - 1.1781724999 | - 0.4554936023 | - 6.6127326193 |
| C | - 0.2393448098 | - 0.7532744684 | 0.1078458859 |
| N | 0.3830958575 | - 1.8295405612 | 0.6906575780 |
| C | 1.7425144412 | - 1.9454713955 | 1.1226348618 |
| C | 2.0158736790 | - 2.3296700495 | 2.4421359893 |
| C | 2.7711603930 | - 1.7112983993 | 0.1883460307 |
| C | 3.3567664925 | - 2.3814639901 | 2.8413019409 |
| C | 4.0879897385 | - 1.7699250023 | 0.6406044875 |
| C | 4.4028042506 | - 2.0836674294 | 1.9684508347 |
| H | 3.5822219157 | - 2.6695015873 | 3.8712050170 |
| H | 4.8953929450 | - 1.5814865906 | - 0.0714383551 |
| C | 2.4844763831 | - 1.4042365599 | - 1.2564089921 |
| H | 1.5931286257 | - 1.9372896318 | - 1.6172384194 |
| H | 3.3383675660 | - 1.6946983352 | - 1.8805913889 |
| H | 2.3113945925 | - 0.3261984734 | - 1.4029575401 |
| C | 0.9419417314 | - 2.7343554737 | 3.4155485637 |
| H | 0.9726403187 | - 3.8252233273 | 3.5599437530 |
| H | - 0.0669381400 | - 2.4843049517 | 3.0718844287 |
| H | 1.1189343928 | - 2.2723314709 | 4.3968828947 |
| C | 5.8353539962 | - 2.1013707405 | 2.4299389819 |
| H | 6.4825915024 | - 2.5910023525 | 1.6894718434 |

| | | | |
|---|---|---|---|
| H | 5.9395080635 | - 2.6249552960 | 3.3886145865 |
| H | 6.2091613403 | - 1.0747428175 | 2.5629097726 |
| H | 0.6535545441 | 0.0635531280 | - 5.0233614242 |
| H | - 0.6714202720 | 2.2818716791 | - 0.3143264953 |
| C | - 1.2437011576 | - 0.0821472629 | 3.4907805173 |
| C | - 0.8835827477 | 1.1712571645 | 3.1787771899 |
| C | - 1.5654121548 | 1.9176918902 | 2.0891958394 |
| C | - 2.7651580359 | 1.2884585860 | 1.4790465996 |
| C | - 3.1277817211 | 0.0380930145 | 1.7939227819 |
| H | - 0.7502004163 | - 0.6502426833 | 4.2796754767 |
| H | - 4.0024121883 | - 0.4494571059 | 1.3582943495 |
| O | - 1.1711734367 | 3.0066439854 | 1.7136122978 |
| C | - 2.3020861424 | - 0.7831594929 | 2.7174889837 |
| O | - 2.4666714263 | - 1.9858999624 | 2.8019043401 |
| H | - 0.0711513761 | 1.6933751442 | 3.6882861119 |
| H | - 3.3226492690 | 1.9025831772 | 0.7683997014 |

Calculated data.

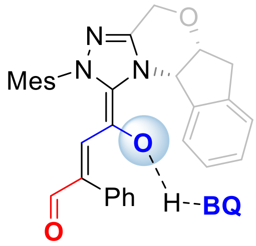


**BQ-b-TS**

Optimization: M062X-D3/def2SVP/IEFPCM($CH_3CN$)

E(RM062X-D3) = -1967.88376516 a.u.

The lowest frequency = -1342.1450 $cm^{-1}$

Number of imaginary frequencies = 1

Single-point energy calculation:

RM062X-D3/def2TZVPP/IEFPCM(CH3CN) E(RM062X-D3) = -1970.07640443 a.u.

Sum of electronic energy and thermal correction to G = -1969.5244844 a.u. (Shermo)

| | | | |
|---|---|---|---|
| O | 2.5636312523 | 2.8043034927 | 3.0356355033 |
| O | - 2.0591912932 | 1.2876012675 | 0.7738986537 |
| C | 0.3771074776 | 3.0270717929 | 1.1777218924 |
| C | 0.4961170803 | 1.9514977359 | 2.1912506708 |
| C | - 1.4646208563 | 0.4733224243 | 1.5823833667 |
| C | - 0.7177180019 | 3.9019569792 | 1.1739542473 |
| H | - 1.5143360780 | 3.7614422725 | 1.9065794407 |
| C | - 0.3136514770 | 0.8758100427 | 2.3720163896 |
| H | - 0.0431717431 | 0.2123354344 | 3.1952176708 |
| C | 1.4075539731 | 3.2187963721 | 0.2472018727 |
| H | 2.2709694552 | 2.5510293503 | 0.2563430229 |
| C | 1.6724085857 | 1.9929432897 | 3.0976705606 |
| H | 1.6887762004 | 1.1784759569 | 3.8619691788 |
| C | 1.3289188029 | 4.2475093101 | - 0.6906733087 |
| H | 2.1303656745 | 4.3784274124 | - 1.4193573560 |
| C | 0.2316218462 | 5.1092438758 | - 0.6938918147 |
| H | 0.1727522720 | 5.9176250913 | - 1.4243578769 |
| C | - 0.7883654746 | 4.9368041149 | 0.2437210452 |
| H | -1.6455264573 | 5.6119245139 | 0.2502805252 |
| C | - 4.2013401206 | - 3.6089873137 | 1.0982675220 |
| C | - 2.9935945207 | - 2.7843108159 | 1.4025318291 |
| C | - 3.9476268922 | - 0.8633807572 | 0.1428215405 |
| C | - 5.2043310309 | - 1.7624533324 | 0.0285463209 |

| | | | |
|---|---|---|---|
| H | - 4.8660667541 | - 3.5593882276 | 1.9822137840 |
| H | - 3.9136835077 | - 4.6532474845 | 0.9339309477 |
| H | - 4.2154200310 | 0.1270242302 | 0.5332459979 |
| H | - 5.8964093898 | - 1.6121971658 | 0.8725557785 |
| O | - 4.8286664068 | - 3.1235801386 | - 0.0552193804 |
| N | - 1.9313425238 | - 3.1205390403 | 2.0621821268 |
| N | 2.9754055697 | - 1.4590936593 | 1.0608462115 |
| C | - 5.7912733788 | - 1.3816242329 | - 1.3296066894 |
| H | - 6.4527567706 | - 0.5077727285 | - 1.2268228263 |
| H | - 6.3797121138 | - 2.2128124882 | - 1.7391369420 |
| C | - 3.4853975615 | - 0.7537205636 | - 1.2976226229 |
| C | - 2.2423569646 | - 0.4006919522 | - 1.8106055630 |
| C | - 4.5642032671 | - 1.0393165729 | - 2.1410071906 |
| C | - 2.0843374719 | - 0.3504421870 | - 3.1984532914 |
| H | - 1.4090173263 | - 0.1587386891 | - 1.1511055188 |
| C | - 4.4080807222 | - 0.9773884842 | - 3.5222123451 |
| C | - 3.1581765276 | - 0.6342687923 | - 4.0455348709 |
| H | - 5.2443468513 | - 1.2028145469 | - 4.1862949704 |
| H | - 3.0186332200 | - 0.5912657341 | - 5.1267659231 |
| C | - 1.8428474940 | - 0.9229687966 | 1.5928164990 |
| N | - 1.2095856950 | - 1.9756006509 | 2.1778547365 |
| C | 0.1688373258 | - 2.0634120876 | 2.5814407125 |
| C | 0.4846007220 | - 2.4757744608 | 3.8821169304 |
| C | 1.1604870787 | - 1.7490201456 | 1.6299982927 |
| C | 1.8355646866 | - 2.4626163300 | 4.2506780673 |
| C | 2.4870400429 | - 1.7505904657 | 2.0551571592 |
| C | 2.8449582147 | - 2.0799061961 | 3.3684571132 |
| H | 2.0974287608 | - 2.7669385870 | 5.2670149405 |
| H | 3.2678966084 | - 1.4989596647 | 1.3333555885 |
| C | 0.8387701902 | - 1.4155082463 | 0.1966146350 |
| H | - 0.0299197043 | - 1.9784689517 | - 0.1729036127 |
| H | 1.6967978008 | - 1.6584704687 | - 0.4419234291 |
| H | 0.6316416024 | - 0.3397919392 | 0.0710332599 |
| C | - 0.5366587576 | - 2.9647332900 | 4.8729183074 |
| H | - 0.3897731944 | - 4.0439398155 | 5.0326365784 |
| H | - 1.5730247756 | - 2.8177738804 | 4.5533489070 |
| H | - 0.3878271897 | - 2.4728838201 | 5.8445773885 |
| C | 4.2844583666 | - 2.0241600733 | 3.8020719314 |
| H | 4.9393167059 | - 2.4934258837 | 3.0551323641 |

| | | | |
|---|---|---|---|
| H | 4.4314419444 | - 2.5276959947 | 4.7655752404 |
| H | 4.6098578197 | - 0.9784285232 | 3.9112306039 |
| H | - 1.1146471912 | - 0.0842424623 | - 3.6209829699 |
| H | - 2.7517896453 | 1.9811296346 | 1.4889762969 |
| C | - 2.8375911610 | - 0.4547082437 | 4.7575182567 |
| C | - 2.6839637736 | 0.7664792907 | 4.1655876732 |
| C | - 3.5778514392 | 1.2177299007 | 3.1245126237 |
| C | - 4.7726169074 | 0.4421976664 | 2.8968342632 |
| C | - 4.9223757421 | - 0.7861771014 | 3.4547758566 |
| H | - 2.1546276979 | - 0.7931496975 | 5.5364511001 |
| H | - 5.8149856215 | - 1.3931992212 | 3.2845092191 |
| O | - 3.3768787662 | 2.3000598123 | 2.4399279283 |
| C | - 3.8784950679 | - 1.3740994330 | 4.3098571365 |
| O | - 3.9028766332 | - 2.5762110808 | 4.5981971934 |
| H | - 1.8803286852 | 1.4445390714 | 4.4595627900 |
| H | - 5.5339690430 | 0.8758120832 | 2.2438288092 |

Calculated data.

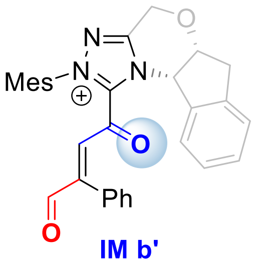


IM b'

Optimization: M062X-D3/def2SVP/IEFPCM(CH3CN)

E(RM062X-D3) = -1967.89592785 a.u.

The lowest frequency = 11.9214 cm$^{-1}$

Number of imaginary frequencies = 0

Single-point energy calculation:

RM062X-D3/def2TZVPP/IEFPCM(CH3CN) E(RM062X-D3) = -1970.09236908 a.u.

Sum of electronic energy and thermal correction to G = -1969.5373614 a.u. (Shermo)

| | | | |
|---|---|---|---|
| O | 4.0883733943 | 2.9178403618 | 1.7693350436 |
| O | - 0.4564066816 | 1.4581778967 | - 0.7753948264 |
| C | 2.0469543685 | 3.1588663134 | - 0.2775436996 |
| C | 2.0278734924 | 2.1606748997 | 0.8114820508 |
| C | 0.0860700697 | 0.7504393275 | 0.0436434872 |
| C | 0.9277643088 | 3.9677910675 | - 0.5206735193 |
| H | 0.0347323373 | 3.8261460833 | 0.0895910324 |
| C | 1.1398122398 | 1.1604738163 | 0.9948387185 |
| H | 1.2722258642 | 0.5163054415 | 1.8647828961 |
| C | 3.2075305055 | 3.3330970627 | - 1.0467744839 |
| H | 4.0839643385 | 2.7135080848 | - 0.8521298723 |
| C | 3.1346432039 | 2.1881495643 | 1.8252443195 |
| H | 3.0143433615 | 1.4496161516 | 2.6525921179 |
| C | 3.2346640832 | 4.2838867341 | - 2.0647238951 |
| H | 4.1339706072 | 4.4032702822 | - 2.6703924708 |
| C | 2.1170281231 | 5.0852415168 | - 2.3035024418 |
| H | 2.1434234199 | 5.8354593930 | - 3.0953557110 |
| C | 0.9673927449 | 4.9298366429 | - 1.5270422849 |
| H | 0.0952475725 | 5.5594713592 | - 1.7084901844 |
| C | - 2.6869997362 | -3.3421864314 | - 0.3245989678 |
| C | - 1.4484630494 | -2.5410915275 | - 0.0998457148 |
| C | - 2.4466059988 | -0.5978816136 | - 1.2883448733 |

| | | | |
|---|---|---|---|
| C | - 3.7238181395 | -1.4758968691 | - 1.3405061082 |
| H | - 3.2676050209 | - 3.2667759610 | 0.6153621049 |
| H | - 2.4326475564 | - 4.3920379991 | - 0.5071336460 |
| H | - 2.6692653395 | 0.3963115463 | - 0.8794453314 |
| H | - 4.3738226471 | - 1.3086048525 | - 0.4702547174 |
| O | - 3.3776569367 | - 2.8440600442 | - 1.4379087162 |
| N | - 0.3399215213 | - 2.8919381694 | 0.4864123206 |
| N | - 1.4324992746 | - 1.2151379667 | - 0.4224856896 |
| C | - 4.3621846928 | - 1.0857593352 | - 2.6729293189 |
| H | - 4.9973035053 | - 0.1959754690 | - 2.5436873338 |
| H | - 4.9880372898 | - 1.9041030715 | - 3.0516604795 |
| C | - 2.0429471077 | - 0.5160533015 | - 2.7491860241 |
| C | - 0.8115009133 | - 0.2188996726 | - 3.3224923952 |
| C | - 3.166000142 | - 0.7779591746 | - 3.5410773074 |
| C | - 0.7140653719 | - 0.1881144257 | - 4.7163421863 |
| H | 0.064691165 | - 0.0097163739 | - 2.7086302713 |
| C | - 3.0700402289 | - 0.7351478117 | - 4.9284084015 |
| C | - 1.8346674006 | - 0.4393074751 | - 5.5112894027 |
| H | - 3.9418442988 | - 0.9419292533 | - 5.5515272943 |
| H | - 1.7424436794 | - 0.4120439034 | - 6.5980497078 |
| C | - 0.2838108448 | - 0.7120487340 | 0.0638483532 |
| N | 0.3745544692 | - 1.7584677620 | 0.5860941649 |
| C | 1.7324920188 | - 1.7772909377 | 1.0557513274 |
| C | 2.0017199565 | - 2.2372849913 | 2.3530881939 |
| C | 2.7358468303 | - 1.3067216806 | 0.1872246690 |
| C | 3.3119396372 | - 2.0764460174 | 2.8182321108 |
| C | 4.0221912228 | - 1.1782875032 | 0.7061497878 |
| C | 4.3218960850 | - 1.5209738458 | 2.0304693478 |
| H | 3.5434336521 | - 2.4015335557 | 3.8351309550 |
| H | 4.8156289436 | - 0.8030433949 | 0.0552281322 |
| C | 2.4730547099 | - 0.9492096896 | - 1.2539589451 |
| H | 1.6636200184 | - 1.5519499472 | - 1.6883461492 |
| H | 3.3790728143 | - 1.1269010351 | - 1.8461897184 |
| H | 2.2206763704 | 0.1177873514 | - 1.3753847823 |
| C | 0.9896316393 | - 2.9376628172 | 3.2197859167 |
| H | 1.1625044885 | - 4.0231231373 | 3.1461129536 |
| H | - 0.0555850120 | - 2.7555205608 | 2.9424203274 |
| H | 1.1376532986 | - 2.6554867736 | 4.2708725654 |
| C | 5.7042826521 | - 1.2938563999 | 2.5769234344 |

| | | | |
|---|---|---|---|
| H | 6.4694817834 | - 1.6461753854 | 1.8718614699 |
| H | 5.8452051365 | - 1.8048931747 | 3.5371286705 |
| H | 5.8744521528 | - 0.2174890411 | 2.7335871432 |
| H | 0.2455580298 | 0.0351732795 | - 5.1840514239 |
| H | - 2.3688452427 | 3.0541885634 | 0.9748767905 |
| C | - 1.3104885832 | - 0.4649466018 | 3.2915807025 |
| C | - 1.1441884610 | 0.8683568357 | 2.9434251495 |
| C | - 2.0131849624 | 1.496799069 | 2.0387768514 |
| C | - 3.1417241189 | 0.7901379659 | 1.6065806141 |
| C | - 3.3191568040 | - 0.5435613282 | 1.9573773933 |
| H | - 0.6238366970 | - 0.922520245 | 4.0051570117 |
| H | - 4.2179161135 | - 1.0784531082 | 1.6414864869 |
| O | - 1.7482202823 | 2.7872876313 | 1.6642449484 |
| C | - 2.3548622781 | - 1.2830403725 | 2.7292230998 |
| O | - 2.4248739004 | - 2.5405311662 | 2.8839609036 |
| H | - 0.3301216378 | 1.4591862641 | 3.3701444915 |
| H | - 3.8897966440 | 1.2996381127 | 0.9896159591 |
