# Supplementary material for: Carbene-catalyzed chemoselective reaction of unsymmetric enedials for access to Furo[2,3-b]pyrroles
Source: Nat Commun. 2023 Jul 15;14:4243. doi: 10.1038/s41467-023-39988-z (PMC10349821; doi:10.1038/s41467-023-39988-z)
Supplement: Supplementary file 4 — Reporting Summary [file 41467_2023_39988_MOESM4_ESM.pdf]

nature portfolio

Corresponding author(s): Yonggui Robin Chi

Last updated by author(s): Jun 15, 2023

Reporting Summary

Nature Portfolio wishes to improve the reproducibility of the work that we publish. This form provides structure for consistency and transparency in reporting. For further information on Nature Portfolio policies, see our Editorial Policies and the Editorial Policy Checklist.

Statistics

For all statistical analyses, confirm that the following items are present in the figure legend, table legend, main text, or Methods section.

n/a	Confirmed	
☐	☒	The exact sample size (*n*) for each experimental group/condition, given as a discrete number and unit of measurement
☐	☒	A statement on whether measurements were taken from distinct samples or whether the same sample was measured repeatedly
☒	☐	The statistical test(s) used AND whether they are one- or two-sided<br>*Only common tests should be described solely by name; describe more complex techniques in the Methods section.*
☐	☒	A description of all covariates tested
☐	☒	A description of any assumptions or corrections, such as tests of normality and adjustment for multiple comparisons
☒	☐	A full description of the statistical parameters including central tendency (e.g. means) or other basic estimates (e.g. regression coefficient) AND variation (e.g. standard deviation) or associated estimates of uncertainty (e.g. confidence intervals)
☒	☐	For null hypothesis testing, the test statistic (e.g. *F*, *t*, *r*) with confidence intervals, effect sizes, degrees of freedom and *P* value noted<br>*Give P values as exact values whenever suitable.*
☒	☐	For Bayesian analysis, information on the choice of priors and Markov chain Monte Carlo settings
☐	☒	For hierarchical and complex designs, identification of the appropriate level for tests and full reporting of outcomes
☒	☐	Estimates of effect sizes (e.g. Cohen's *d*, Pearson's *r*), indicating how they were calculated

Our web collection on statistics for biologists contains articles on many of the points above.

Software and code

Policy information about availability of computer code

Data collection	Gaussian V 16.0, A03; VMD V1.93; Multiwfn 4.
Data analysis	Lu, T.; Chen, Q. Comput. Theor. Chem. 2020, 1200, 113249; Lu, T.; Chen, F. J. Comp. Chem. 2012, 33, 580-592 5. Humphrey, W.;Lu, T.; Chen, Q. J. Comp. Chem. 2022, 43, 539-555.

For manuscripts utilizing custom algorithms or software that are central to the research but not yet described in published literature, software must be made available to editors and reviewers. We strongly encourage code deposition in a community repository (e.g. GitHub). See the Nature Portfolio guidelines for submitting code & software for further information.

Data

Policy information about availability of data

All manuscripts must include a data availability statement. This statement should provide the following information, where applicable:

- Accession codes, unique identifiers, or web links for publicly available datasets
- A description of any restrictions on data availability
- For clinical datasets or third party data, please ensure that the statement adheres to our policy

The experimental method and data generated in this study are provided in the Supplementary Information file. The crystallographic data for structures of 1a, 3a, 5, 6 and 7 have been deposited in the Cambridge Crystallographic Data Centre under accession CCDC code 2156768, 2076667, 2156751, 2156752 and 2156755, respectively. Copies of the data can be obtained free of charge via www.ccdc.cam.ac.uk/data_request/cif.

Research involving human participants, their data, or biological material

Policy information about studies with human participants or human data. See also policy information about sex, gender (identity/presentation), and sexual orientation and race, ethnicity and racism.

Reporting on sex and gender	This experiment did not involve sex and gender.
Reporting on race, ethnicity, or other socially relevant groupings	This experiment did not involve race, ethnicity, or other socially relevant groupings.
Population characteristics	This experiment did not involve population characteristics.
Recruitment	This experiment did not involve Recruitment.
Ethics oversight	This experiment did not involve Ethics oversight.

Note that full information on the approval of the study protocol must also be provided in the manuscript.

Field-specific reporting

Please select the one below that is the best fit for your research. If you are not sure, read the appropriate sections before making your selection.

☒ Life sciences ☐ Behavioural & social sciences ☐ Ecological, evolutionary & environmental sciences

For a reference copy of the document with all sections, see nature.com/documents/nr-reporting-summary-flat.pdf

Life sciences study design

All studies must disclose on these points even when the disclosure is negative.

Sample size	No sample-size calculation was performed. As per international practice, investigators conducted three parallel experiments in each experimental group.
Data exclusions	This experiment did not exclude any data.
Replication	All attempts at replication were successful.
Randomization	Samples were random allocated into experimental groups.
Blinding	Investigators were blinded to group allocation during data collection and analysis.

Reporting for specific materials, systems and methods

We require information from authors about some types of materials, experimental systems and methods used in many studies. Here, indicate whether each material, system or method listed is relevant to your study. If you are not sure if a list item applies to your research, read the appropriate section before selecting a response.

Materials & experimental systems

n/a	Involved in the study	
☒	☐	Antibodies
☒	☐	Eukaryotic cell lines
☒	☐	Palaeontology and archaeology
☒	☐	Animals and other organisms
☒	☐	Clinical data
☒	☐	Dual use research of concern
☒	☐	Plants

Methods

n/a	Involved in the study	
☒	☐	ChIP-seq
☒	☐	Flow cytometry
☒	☐	MRI-based neuroimaging
